# Stannylenes and Germylenes Stabilized by Tetradentate Bis(amidine) Ligands with a Rigid Naphthalene Backbone †

**DOI:** 10.3390/molecules29020325

**Published:** 2024-01-09

**Authors:** Alejandra Acuña, Sonia Mallet-Ladeira, Jean-Marc Sotiropoulos, Eddy Maerten, Alan R. Cabrera, Antoine Baceiredo, Tsuyoshi Kato, René S. Rojas, David Madec

**Affiliations:** 1 Laboratoire Hétérochimie Fondamentale et Appliquée (UMR 5069), Université de Toulouse, CNRS, 118 Route de Narbonne, 31062 Toulouse, Cedex 09, France; azacuna@uc.cl (A.A.); eddy.maerten@univ-tlse3.fr (E.M.); jose-antoine.baceiredo@univ-tlse3.fr (A.B.); tsuyoshi.kato@univ-tlse3.fr (T.K.); 2 Departamento de Química Inorgánica, Facultad de Química y de Farmacia, Pontificia Universidad Católica de Chile, Vicuña Mackenna 4860, Santiago 7820436, Chile; arcabrer@uc.cl; 3 Institut de Chimie de Toulouse (UAR 2599), 118 Route de Narbonne, 31062 Toulouse, Cedex 09, France; sonia.ladeira@univ-tlse3.fr; 4 IPREM, UMR 5254, UPPA/CNRS Technopole Helioparc-2, av. Pdt P. Angot, 64053 Pau, Cedex 09, France; jean-marc.sotiro@univ-pau.fr

**Keywords:** amidine, ligand, metallylene

## Abstract

An unusual series of germylenes and stannylenes stabilized by new tetradentate bis(amidine) ligands RNC(R′)N-linker-NC(R′)NR with a rigid naphthalene backbone has been prepared by protonolysis reaction of Lappert’s metallylenes [M(HMDS)_2_] (M = Ge or Sn). Germylenes and stannylenes were fully characterized by NMR spectroscopy and X-ray diffraction analysis. DFT calculations have been performed to clarify the structural and electronic properties associated with tetradentate bis(amidine) ligands. Stannylene **L_1_Sn** shows reactivity through oxidation, oxidative addition, and transmetalation reactions, affording the corresponding gallium and aluminum derivatives.

## 1. Introduction

Amidines have been employed as ligands for numerous metallic and semi-metallic elements [1], and one of their best features is the ability to adjust both steric and electronic properties, leading to many substitution patterns of the CN_2_ framework [2,3,4]. In recent years, the trend has shifted to develop bulkier systems to stabilize a broader range of metal centers or to control the complex geometry via rigid coordination, as seen with bridged bis(amidine) ligands.

**Figure 1:**
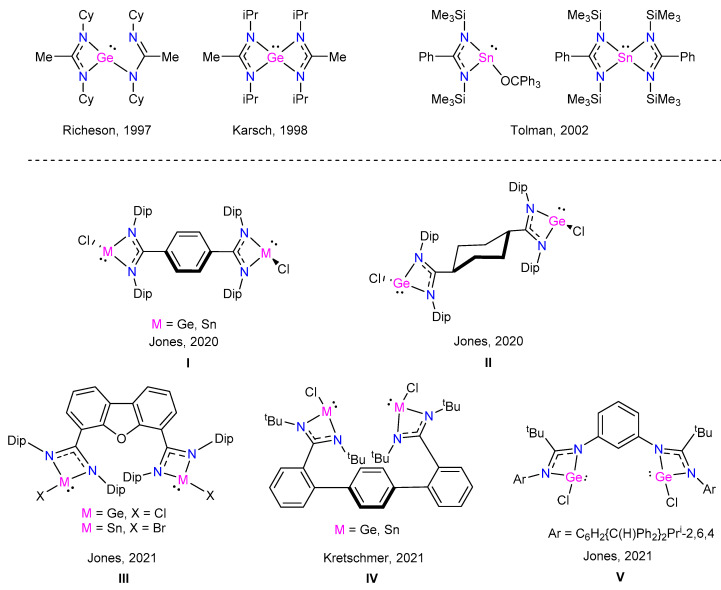
Examples of germylenes and stannylenes with bis(amidine) ligands [6,7,8,9,10,11].

Amidinate ligands have shown a remarkable ability to stabilize low-valent main group elements, such as tetrylenes, forming planar NCN-Ge^(II)^ and NCN-Sn^(II)^ four-membered rings [5]. The first bis(amidinato)germylene, reported by Richeson et al. in 1997, exhibited one chelating and one dangling amidinate ligand, with the three-coordinate germanium center adopting a trigonal–pyramidal geometry (Figure 1) [6]. Using the same synthetic method, Karsch et al. reported in 1998 a bis(amidinato)germylene with both amidinate ligands acting as chelates due to the less hindered substituents on the nitrogen atoms [7]. On the other hand, the first mono- and bis-(amidinato)stannylenes were reported by Tolman et al. in 2002, using a *N*-silylated benzamidinate ligand (Figure 1) [8].

More recently, the first tetrylenes with 1,4-phenylene (**I**) and 1,4-cyclohexylene (**II**) bridged bis(amidine) ligands were reported by Jones et al. in 2020 [9]. In 2021, the same group reported a bis-germylene and a bis-stannylene with a dibenzofurandiyl-linked Dipp-substituted bis(amidine) ligand (**III**) [10]. In the same year, Kretschmer et al. reported bis-germylenes and bis-stannylenes using a terphenyl-linked bis(amidine) ligand (**IV**) [11]. It is important to note that all these examples correspond to amidine fragments connected through the carbon atom, and only one bis-germylene with a bis(amidine) ligand nitrogen connected via a 1,3-phenylene bridge has been synthesized by Jones et al. (**V**) [10]. The main characteristic of bridged bis(amidine) ligands exhibited is that the amidine functionalities act as independent ligands, allowing the convenient preparation of stable bis-metallylenes. Moreover, there is no report of a bis(amidine) system acting as a tetradentate ligand with both amidine moieties coordinated to the same metal center. We therefore considered the hypothesis that such a bis(amidine) system could influence the stabilization and the reactivity of the corresponding tetrylenes.

Herein, we report the synthesis and structural characterization of novel stannylene and germylene compounds stabilized by naphthalene-linked bis(amidine) ligands, with the amidine functionalities connected to the naphthalene bridge through the nitrogen atoms. In addition, the reactivity of synthesized stannylenes was explored in oxidation, oxidative addition, and transmetalation reactions.

## 2. Results and Discussion

### 2.1. Synthesis of Bis(amidine) Ligands with a Rigid Naphthalene Backbone

**Scheme 1:**
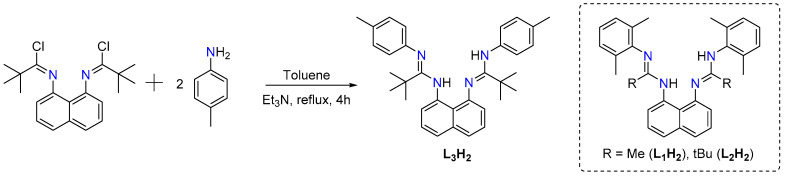
Synthesis of ligand **L_3_H_2_**.

Bis(amidine) ligands **L_1_H_2_** and **L_2_H_2_**, with a rigid planar 1,8-diaminonaphthalene linker, were prepared following the reported procedures [12,13], while the synthesis of **L_3_H_2_** was carried out through the reaction between one equivalent of *N*,*N*′-(naphthalene-1,8-diyl)bis(2,2-dimethylpropanimidoyl chloride) and two equivalents of *p*-tolylamine in dry toluene under reflux for 4 h in the presence of Et_3_N (Scheme 1). **L_3_H_2_** was isolated as yellow crystals in 63% yield and characterized by NMR spectroscopy. The ^1^H NMR spectrum shows two NH signals at 9.43 and 8.50 ppm, similar to those previously reported bis(amidine) ligands [13,14]. In addition, CH_3_ resonances of *p*-tolyl and *^t^*butyl fragments appear as singlets at 2.05 and 2.01 ppm and at 1.38 and 1.23 ppm, respectively. The ^13^C NMR spectrum also exhibits two different C=N groups at 161.3 and 160.7 ppm, confirming the non-symmetrical character of **L_3_H_2_** ligand in solution due to NH^…^N hydrogen bonds.

**Figure 2:**
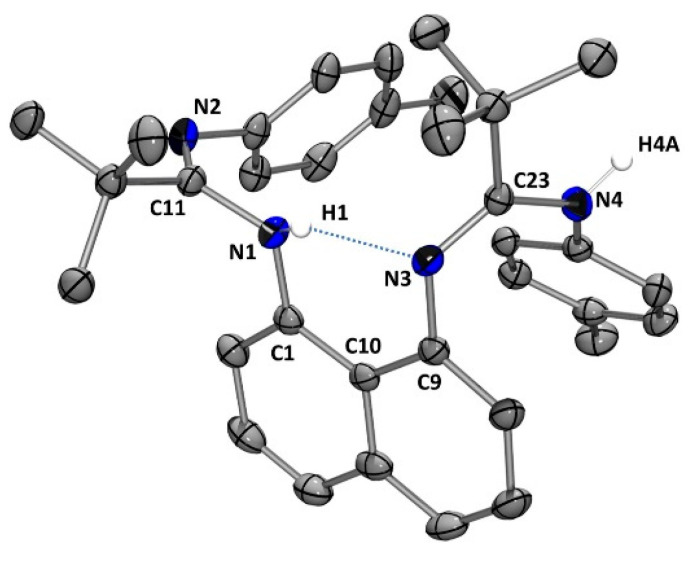
Molecular structure of **L_3_H_2_**. Thermal ellipsoids are represented with a 30% probability. Hydrogen atoms (except H1 and H4A) have been omitted for clarity. Selected bond distances [Å] and bond angles [deg]: N(1)-C(11) 1.421(5); N(2)-C(11) 1.272(5); N(3)-C(23) 1.286(5); N(4)-C(23) 1.373(5); N1-(H1)^…^N(3) 1.95(5); N(1)-C(11)-N(2) 122.5(3); N(3)-C(23)-N(4) 126.1(3).

The presence of an intramolecular hydrogen bond between the N-H group and the nitrogen atom of the amidine moiety [ N1-(H1)^…^N(3) 1.95(5) Å] is confirmed in the solid-state structure of ligand **L_3_H_2_** (Figure 2), in line with similar observations for ligands **L_1_H_2_** and **L_2_H_2_** [12,13].

### 2.2. Synthesis of Stannylenes L_1–3_Sn

**Scheme 2:**
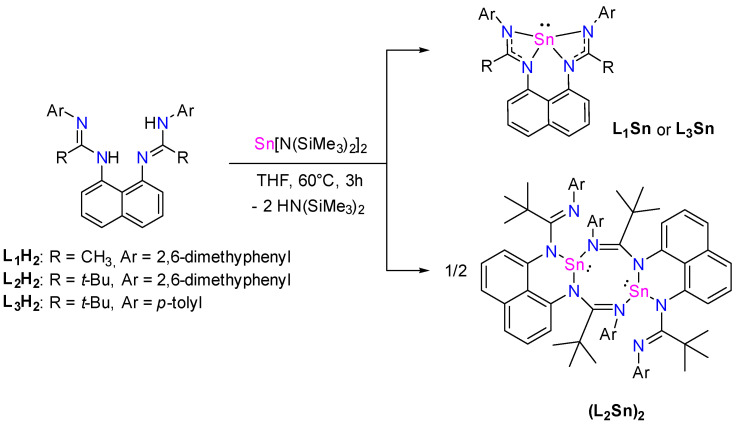
Synthesis of stannylene **L_1–3_Sn**.

The synthesis of stannylene **L_1–3_Sn** was carried out using protonolysis reactions due to the simplicity of the procedure [15,16,17]. Therefore, the corresponding bis(amidine) ligand **L_1–3_H_2_** reacted with one equivalent of Sn(HMDS)_2_ in dry THF at 60 °C for 3 h (Scheme 2). Stannylene **L_1_Sn** was isolated as colorless crystals from a pentane solution at −30 °C in 77% yield. The formation of **L_1_Sn** was confirmed by ^1^H, ^13^C, and ^119^Sn NMR spectroscopy and mass spectrometry. The ^1^H NMR spectrum shows a singlet at 1.97 ppm with an integration of 6 H, corresponding to the two amidine methyl groups, and a singlet at 2.18 ppm with an integration of 12 H, corresponding to the methyl groups of 2,6-dimethylphenyl fragments. This observed NMR pattern agrees with a symmetrical structure around the tin center. The ^13^C NMR spectrum shows a characteristic signal for the NCN fragment at 169.0 ppm, similar to those (amidinato)stannylenes previously reported [8,15,16,18]. In the ^119^Sn NMR spectrum, a resonance at −276.7 ppm confirms the tetracoordinated nature of the tin atom, comparable to that of reported homoleptic four-coordinated bis(amidinate) tin(II) [15]. **L_3_H_2_** also reacts with Sn(HMDS)_2_ in the same manner to give the corresponding tetracoordinated stannylene **L_3_Sn**, as shown by the NMR analysis. Indeed, the ^1^H NMR spectrum shows a singlet at 1.30 ppm that integrates for 18 H, corresponding to the amidine *^t^*butyl groups, and a singlet at 2.29 ppm that integrates for 6 H, corresponding to the *p*-tolyl methyl groups. The ^13^C NMR spectrum shows a characteristic signal for the NCN fragment at 177.5 ppm. The symmetrical tetracoordination of the tin center is also confirmed by a resonance at −254.9 ppm in the ^119^Sn NMR spectrum. In contrast, the use of the bulky bis(amidinate) ligand **L_2_H_2_** leads to the formation of a dimeric structure (**L_2_Sn**)**_2_**.

**Figure 3:**
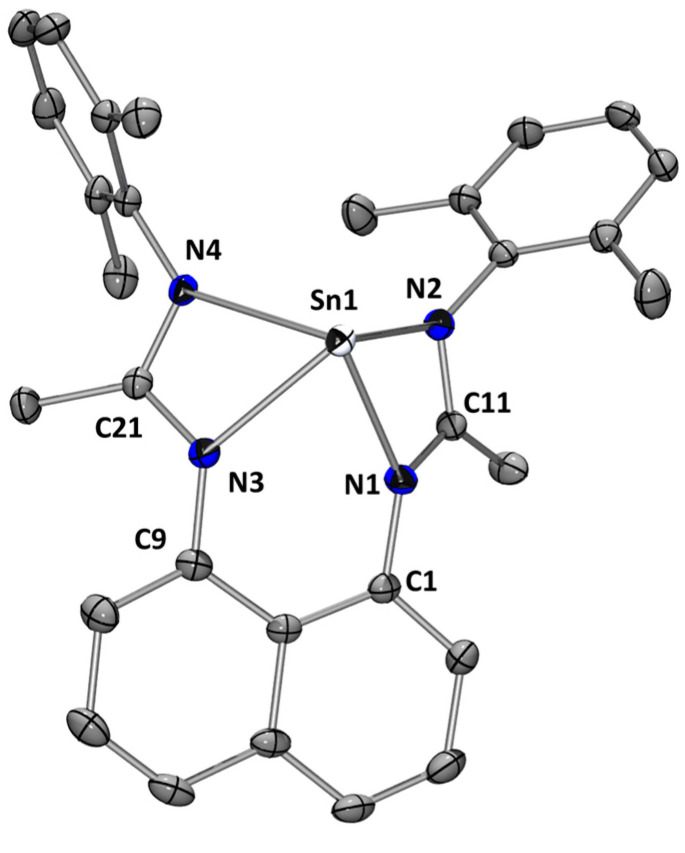
Molecular structure of **L_1_Sn**. Thermal ellipsoids are represented with a 30% probability. Hydrogen atoms have been omitted for clarity. Selected bond distances [Å] and bond angles [deg]: Sn(1)-N(1) 2.2435(17); Sn(1)-N(2) 2.3327(17); Sn(1)-N(3) 2.2232(17); Sn(1)-N(4) 2.3181(16); N(1)-C(11) 1.326(3); N(2)-C(11) 1.328(3); N(3)-C(21) 1.328(3); N(4)-C(21) 1.329(3); N(3)-Sn(1)-N(1) 72.57(6); N(3)-Sn(1)-N(4) 57.74(6); N(1)-Sn(1)-N(4) 107.35(6); N(3)-Sn(1)-N(2) 112.07(6); N(1)-Sn(1)-N(2) 57.38(6); N(4)-Sn(1)-N(2) 95.29(6); N(1)-C(11)-N(2) 111.85(17); N(3)-C(21)-N(4) 111.37(17).

Stannylene **L_1_Sn** was characterized in the solid state by single-crystal X-ray diffraction analysis (Figure 3). The molecular structure indicates a four-coordinate tin center with a distorted square-based pyramidal geometry. A few stannylenes with unbridged bis(amidine) ligands display a similar geometry [16]. The acute N1–Sn–N2 and N3–Sn–N4 angles of 57.38° and 57.74° are comparable to values previously recorded for Sn^(II)^ amidinate complexes [15,18].

**Figure 4:**
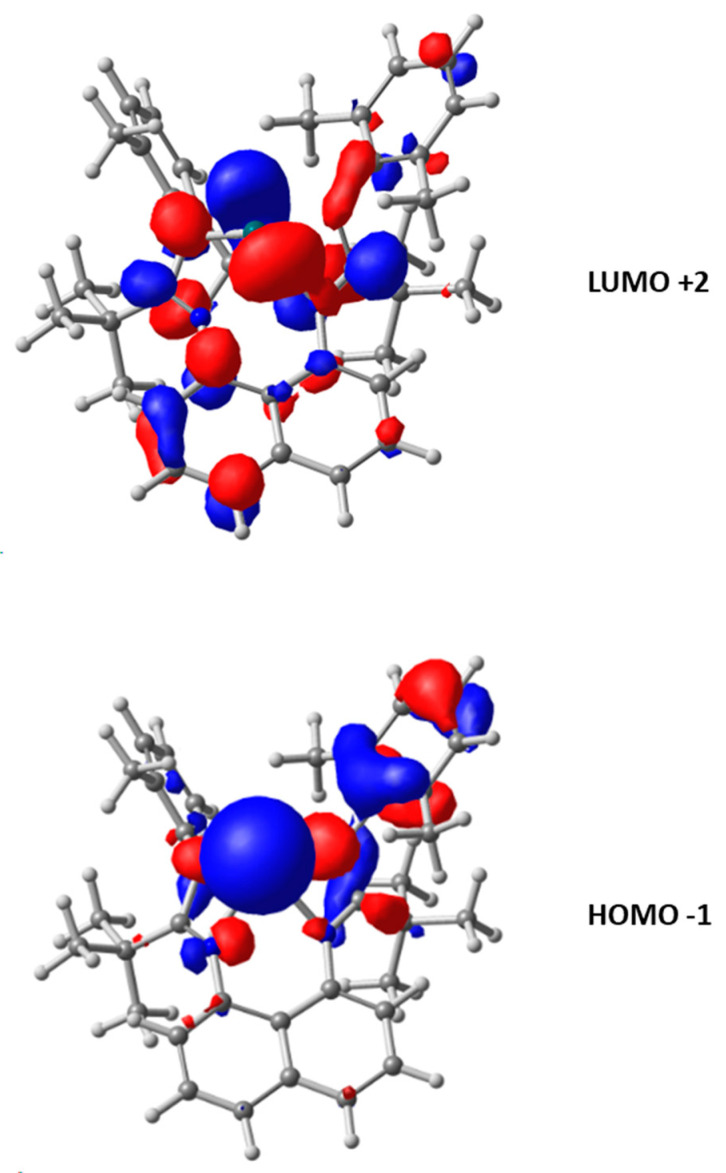
LUMO +2 (up) and HOMO −1 (down) for **L_1_Sn**.

The coordinates of the experimental solid-state structure are consistent with the calculated values in DFT (see Supplementary Materials for more information). Indeed, the modeling calculates values between 2.27 and 2.42 Å for the Sn–N bonds, probably due to steric repulsion, and angles at the tin atom of 56°. Moreover, the energy of molecular orbitals of this species (computed using DFT) shows an electrophilic character (the first vacant orbital on the Sn atom is computed at −0.60 eV) and a potential nucleophilicity, as the HOMO centered on the non-bonding pair of tin is rather accessible (−5.42 eV) (Figure 4).

The modeling demonstrates that this compound can be obtained from the bis(amidine) ligand through the addition of Sn(HMDS)_2_. Indeed, starting from the resulting complex, a prototropic shift can occur, leading to the facile formation of the corresponding RSnH (TS = 21.63 kcal/mol) (see Supplementary Materials for more information). Subsequently, the elimination of HMDS results in the obtaining of **L_1_Sn**, exhibiting a higher but consistent kinetic rate with the applied experimental conditions (60 °C/3 h).

**Figure 5:**
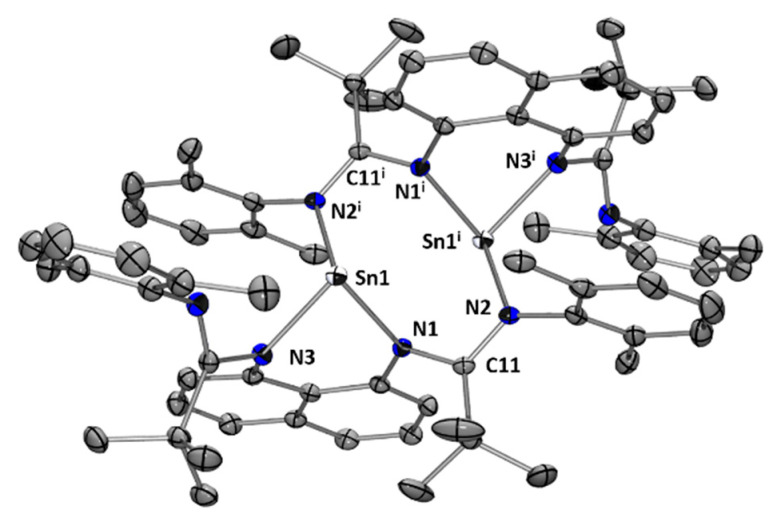
Molecular structure of (**L_2_Sn**)**_2_**. Thermal ellipsoids are represented with a 30% probability. Hydrogen atoms and solvent molecules have been omitted for clarity. Selected bond distances [Å] and bond angles [deg]: Sn(1)-N(1) 2.243(2); Sn(1)-N(2^i^) 2.327(2); Sn(1)-N(3) 2.144(3); C(11)-N(1) 1.378(4); C(11)-N(2) 1.309(4); N(1)-Sn(1)-N(3) 78.80(9); N(3)-Sn(1)-N(2^i^) 97.73(9); N(1)-Sn(1)-N(2^i^) 95.21(9); C(11)-N(1)-Sn(1) 112.43(18); N(1)-C(11)-N(2) 114.2(3).

Because of the poor solubility of stannylene dimer (**L_2_Sn**)**_2_** in common organic solvents, the NMR characterization cannot be obtained once crystallized. Nevertheless, the X-ray diffraction analysis shows an eight-membered cyclic structure with a Sn^…^Sn distance of 3.7311(5) Å (Figure 5). Each Sn center has a trigonal pyramidal geometry with a sum of angles around the Sn atom of 272°. Interestingly, the tin atoms are coordinated to both nitrogen atoms directly linked to the naphthalene core and, simultaneously, to one of the nitrogen atoms from another amidinate fragment. Unfortunately, providing theoretical arguments for the formation of dimer (**L_2_Sn**)**_2_** is challenging. Indeed, calculations reveal an energetically favorable reaction pathway (ΔG = −27.74 kcal/mol) to obtain the corresponding monomer. We can postulate that the presence of the 2,6-dimethylphenyl and *^t^*butyl groups in the ligand introduces significant steric hindrance, inhibiting the arrangement of a tetracoordinated tin center and thereby influencing the preferential formation of the dimer.

**Scheme 3:**
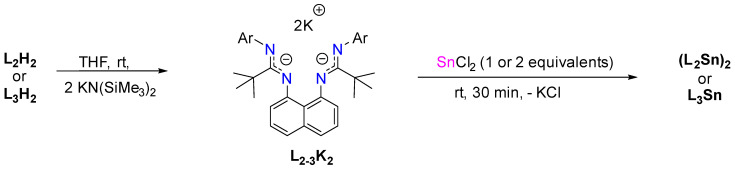
Synthetic routes to obtain **L_2-3_Sn** from deprotonated bis(amidine) ligands.

Stannylene **L_3_Sn** and dimer (**L_2_Sn**)**_2_** can also be prepared through the reaction of deprotonated bis(amidine) ligand (**L_2_K_2_** or **L_3_K_2_**) with one equivalent of SnCl_2_. The same products, (**L_2_Sn**)**_2_** and **L_3_Sn,** were also obtained using two equivalents of SnCl_2_, instead of the expected bis-stannylenes **L_2_Sn_2_** and **L_3_Sn_2_** (Scheme 3).

**Scheme 4:**
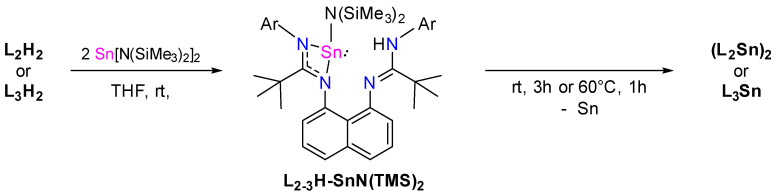
Synthetic routes to obtain **L_2-3_Sn** by protonolysis.

Other attempts to synthesize bis-stannylenes **L_2_Sn_2_** and **L_3_Sn_2_**, by reacting the corresponding ligand (**L_2_H_2_** or **L_3_H_2_**) with two equivalents of Sn(HMDS)_2_ (via protonolysis reactions), have also failed (Scheme 4). In both cases, at room temperature, the formation of an intermediate **LH-SnN**(**TMS**)**_2_** was first detected by ^1^H NMR spectroscopy. The subsequent heating at 60 °C for 1h affords the corresponding stannylenes (**L_2_Sn**)**_2_** or **L_3_Sn** (44% and 61% yield, respectively), accompanied by the formation of H-HMDS and a black solid of Sn^0^ as by-products.

### 2.3. Synthesis of Germylenes Stabilized by Bis(amidine) Ligands

**Scheme 5:**
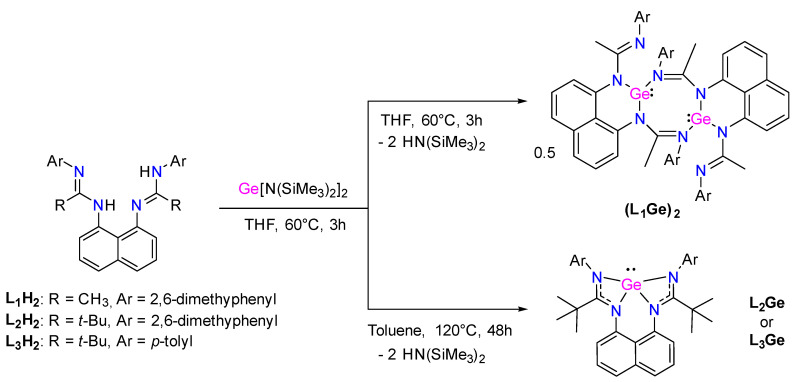
Synthesis of germylenes **L_1–3_Ge**.

**Figure 6:**
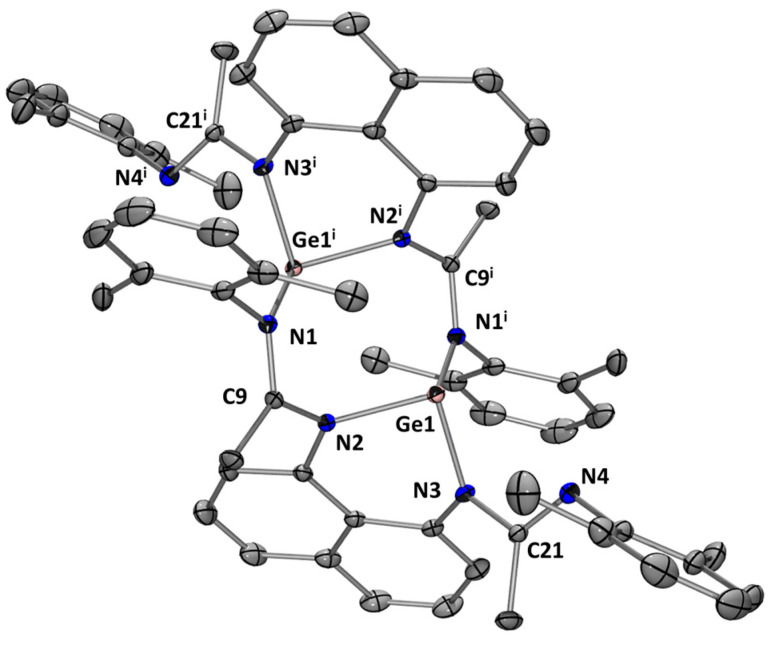
Molecular structure of (**L_1_Ge**)**_2_**. Thermal ellipsoids are represented with a 30% probability. Hydrogen atoms and solvent molecules have been omitted for clarity. Selected bond distances [Å] and bond angles [deg]: Ge(1)-N(1^i^) 2.0961(11); Ge(1)-N(2) 2.0048(10); Ge(1)-N(3) 1.9332(10); C(9)-N(1) 1.3074(16); C(9)-N(2) 1.3740(16); N(3)-Ge(1)-N(2) 87.48(4); N(3)-Ge(1)-N(1^i^) 95.69(4); N(2)-Ge(1)-N(1^i^) 96.79(4); N(1)-C(9)-N(2) 116.31(11); C(9)-N(2)-Ge(1) 112.38(8).

Following the same synthetic strategy, we have considered the reaction of bis(amidinato) ligands **L_1–3_H_2_** with Ge(HMDS)_2_. In the case of **L_1_H_2_**, only dimer (**L_1_Ge**)**_2_** was obtained as yellow crystals in 42% yield (Scheme 5). Because of poor solubility, it was only possible to characterize (**L_1_Ge**)**_2_** by an X-ray diffraction analysis. The eight-membered cyclic molecular structure of (**L_1_Ge**)**_2_** exhibits two trigonal pyramidal Ge centers with a Ge–Ge distance of 3.4984(3) Å (Figure 6).

**Figure 7:**
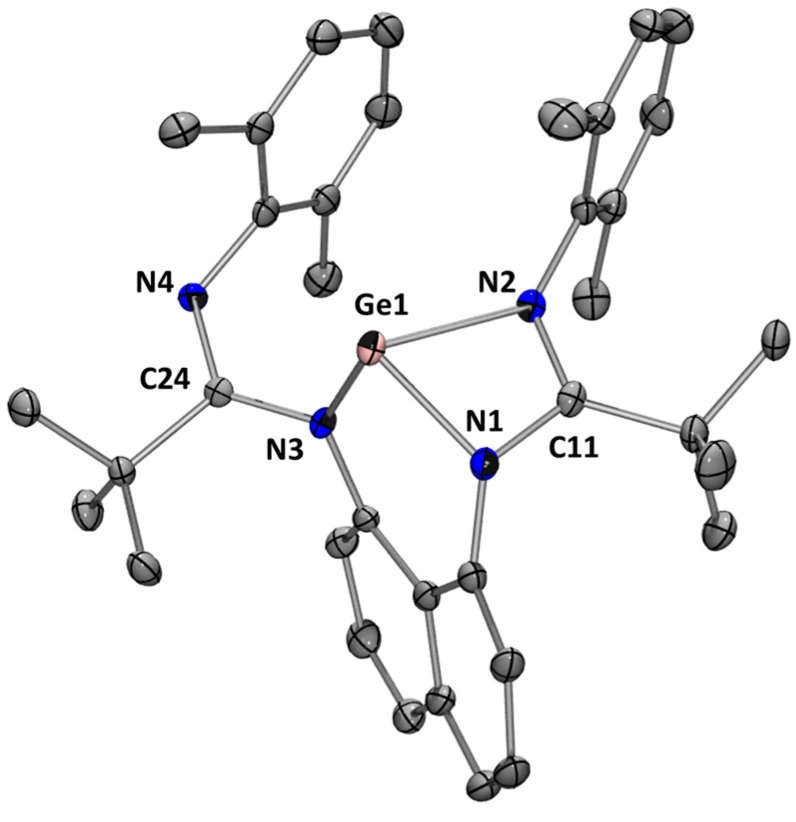
Molecular structure of **L_2_Ge**. Thermal ellipsoids are represented with a 30% probability. Hydrogen atoms have been omitted for clarity. Selected bond distances [Å] and bond angles [deg]: Ge(1)-N(1) 1.969(3); Ge(1)-N(2) 2.073(3); Ge(1)-N(3) 1.912(3); C(11)-N(2) 1.308(5); C(11)-N(1) 1.374(5); C(24)-N(4) 1.275(5); C(24)-N(3) 1.417(5); N(1)-Ge(1)-N(2) 64.89(13); N(1)-Ge(1)-N(3) 90.95(13); N(2)-Ge(1)-N(3) 102.00(13); N(1)-C(11)-N(2) 108.0(3); N(3)-C(24)-N(4) 124.0(3).

The reaction of **L_2_H_2_** with Ge(HMDS)_2_ afforded germylene **L_2_Ge** after heating at 110 °C for 48 h to complete the reaction (Scheme 5). Germylene **L_2_Ge** was isolated as yellow crystals from a pentane solution at −30 °C in 52% yield. The ^1^H NMR spectrum shows a singlet at 1.60 ppm with an integration of 18 H, corresponding to the *^t^*butyl groups, and a singlet at 2.18 ppm with an integration of 12 H, corresponding to the methyl groups of the 2,6-dimethylphenyl substituent. In addition, the ^13^C NMR spectrum shows the characteristic signal for the NCN fragment at 170.9 ppm. These data are in agreement with a symmetrical tetracoordinated germanium center in solution. However, in the solid state, the X-ray diffraction analysis of **L_2_Ge** shows a tri-coordinated germanium atom with a distorted trigonal pyramidal geometry. Here, the coordination is through the two nitrogen atoms of the naphthalene bridge and one nitrogen atom of amidine moiety, leaving the remaining nitrogen atom dangling (Figure 7). The N–C distances [1.275(5)–1.417(5) Å] indicate an electron delocalization within the NCN fragments, and due to the bulky nature of the *^t^*butyl substituents, they are in opposite directions to each other, distorting the naphthalene ring.

### 2.4. Reactivity of Stannylene L_1_Sn

Several tests of reactivity were carried out with stannylene **L_1_Sn**, which is easy to obtain in good yields. We first considered the activation of small molecules such as ethylene, NH_3_, and CO_2_, but without any success because of no reactions, despite the multiple conditions evaluated (temperature, time, and pressure).

**Scheme 6:**
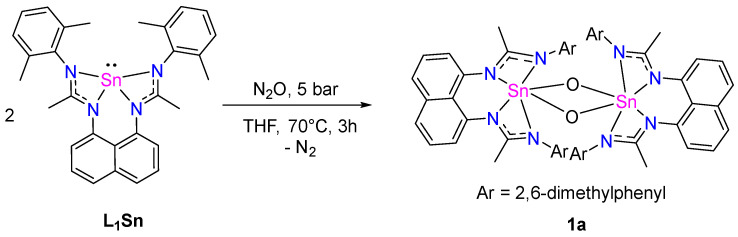
Synthesis of **1a**.

**Figure 8:**
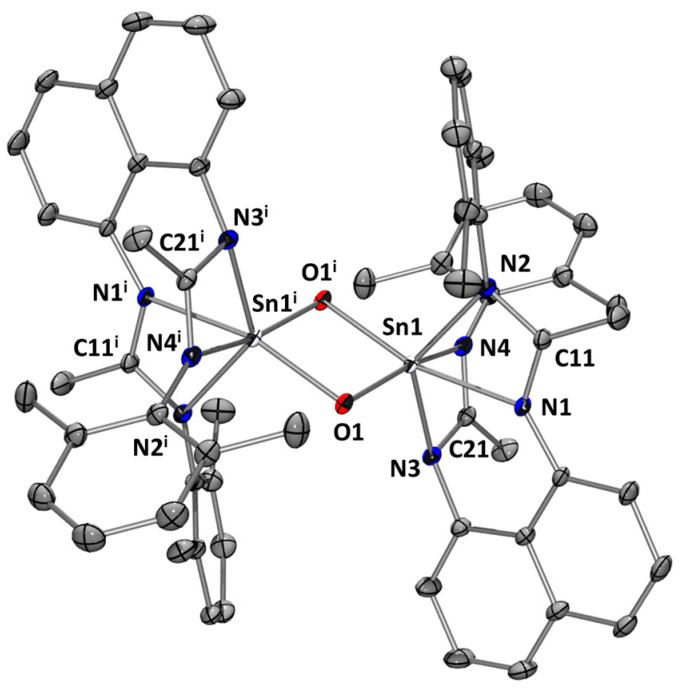
Molecular structure of **1a**. Thermal ellipsoids are represented with a 30% probability. Hydrogens and solvent molecules have been omitted for clarity. Selected bond distances [Å] and bond angles [deg]: Sn(1)-N(1) 2.2236(10); Sn(1)-N(2) 2.1644(11); Sn(1)-N(3) 2.1423(10); Sn(1)-N(4) 2.2742(10); Sn(1)-O(1) 2.0079(9); Sn(1)-O(1^i^) 1.9996(8); Sn(1)-Sn(1^i^) 2.97787(19); C(11)-N(1) 1.3285(16); C(11)-N(2) 1.3415(16); C(21)-N(4) 1.3113(17); C(21)-N(3) 1.3439(17); O(1)-Sn(1)-O(1^i^) 84.01(4); Sn(1)-O(1)-Sn(1^i^) 95.98(4); N(1)-C(11)-N(2) 110.50(11); N(3)-C(21)-N(4) 112.95(11).

However, when exposed to N_2_O (five bars) in THF solution, **L_1_Sn** slowly reacts (3 h at 70 °C) to give a dimeric amidinate-stannoxane **1a**, which was isolated as colorless crystals in 46% yield (Scheme 6). Compound **1a** is moderately soluble in THF, and the ^1^H NMR signals tend to be broadened. Therefore, the coupling of signals in the aromatic region cannot be clearly seen, but the integration is as expected, with two broad singlets at 2.07 and 1.85 ppm that integrate for 12 H, corresponding to the methyl groups of the 2,6-dimethylphenyl fragment. In addition, a resonance at 1.76 ppm, integrating for 12 H and corresponding to the methyl groups of the amidine moieties, was observed. The characteristic signal of the carbon amidinate fragments in the ^13^C NMR spectrum resonates at 168.7 ppm. In addition, a ^119^Sn chemical shift at −494.5 ppm was observed, consistent with reported stannoxane dimers [19]. The crystal structure of **1a** shows a dinuclear species with a central planar Sn_2_O_2_ ring with two hexacoordinated tin atoms in a distorted pseudo-octahedral geometry (Figure 8). The O–Sn distances (~2.000 Å), Sn–Sn contact (2.9779(2) Å), and O–Sn–O angles (84.01°) are in the range of other dimeric tin amidinate-stannoxanes reported [19].

**Scheme 7:**
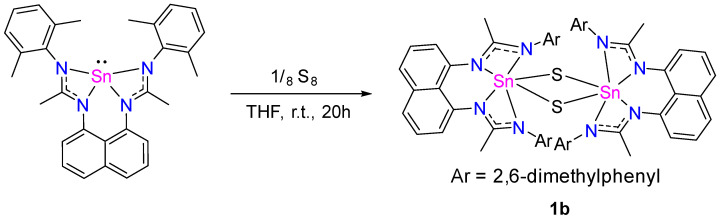
Synthetic route to obtain **1b**.

**Figure 9:**
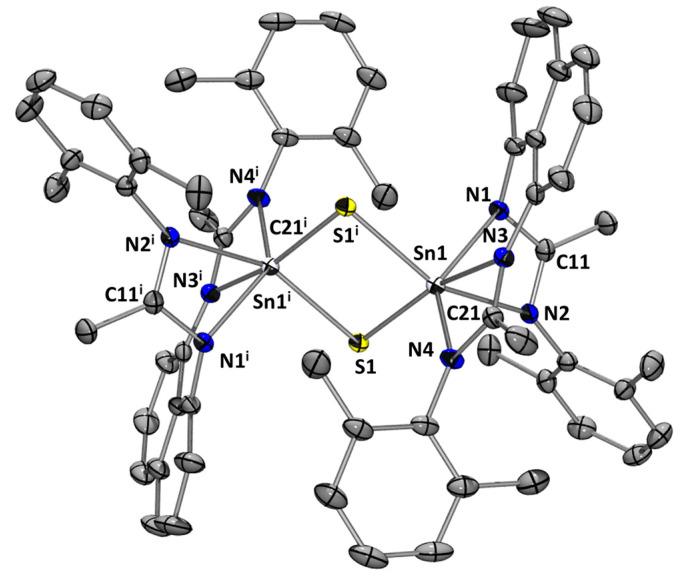
Molecular structure of **1b**. Thermal ellipsoids are represented with a 30% probability. Hydrogen atoms and solvent molecules have been omitted for clarity. The asymmetric unit contains two independent but very similar molecules; data for only one molecule are discussed. Selected bond distances [Å] and bond angles [deg]: Sn(1)-N(1) 2.130(4); Sn(1)-N(2) 2.374(3); Sn(1)-N(3) 2.240(3); Sn(1)-N(4) 2.198(4); Sn(1)-S(1) 2.4172(14); Sn(1)-S(1^i^) 2.4585(13); C(11)-N(1) 1.345(6); C(11)-N(2) 1.315(5); C(21)-N(3) 1.332(5); C(21)-N(4) 1.328(5); Sn(1)-S(1)-Sn(1^i^) 87.92(4); S(1)-Sn(1)-S(1^i^) 92.08(4); N(1)-C(11)-N(2) 112.8(4); N(3)-C(21)-N(4) 110.9(4).

Usually, stannylenes readily react with chalcogenides [6,20,21,22,23,24,25], and therefore we have investigated the reaction of **L_1_Sn** with elemental sulfur (Scheme 7). After the reaction mixture had been stirred at room temperature for 4 h, ^1^H, and ^119^Sn NMR spectroscopy showed a mixture of new signals and starting stannylene. In the ^119^Sn NMR spectrum, next to the resonance of **L_1_Sn**, a new signal appears at −371 ppm, which evolves to a resonance singlet at −648.0 ppm after 24 h, corresponding to dimer **1b**. After purification, **1b** was isolated as pale-yellow crystals from a THF solution at −30 °C in 51% yield. The dimeric structure of **1b** has been determined by an X-ray diffraction analysis, revealing a central planar Sn_2_S_2_ ring with the two tin centers in a distorted pseudo-octahedral geometry (Figure 9). The S–Sn distances are ~2.400 Å and Sn–Sn contact is 3.3847(10) Å [25], longer than that observed for the di-oxygen analog dimer **1a** due to the size of the sulfur atom.

In addition, compound **1b** was characterized by NMR spectroscopy. The ^1^H NMR spectrum exhibits broadened signals. In the ^13^C NMR spectrum, the characteristic signal of the NCN fragment appears at 167.4 ppm. In the ^119^Sn NMR spectrum, a very high field chemical shift at −648.0 ppm is observed, a resonance signal that is more high-field shifted than those of previously reported Sn_2_S_2_-bridged dimeric complexes [26,27]. 

**Scheme 8:**
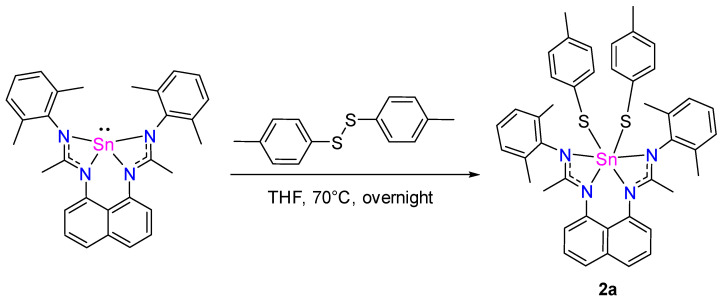
Synthesis of **2a**.

**Figure 10:**
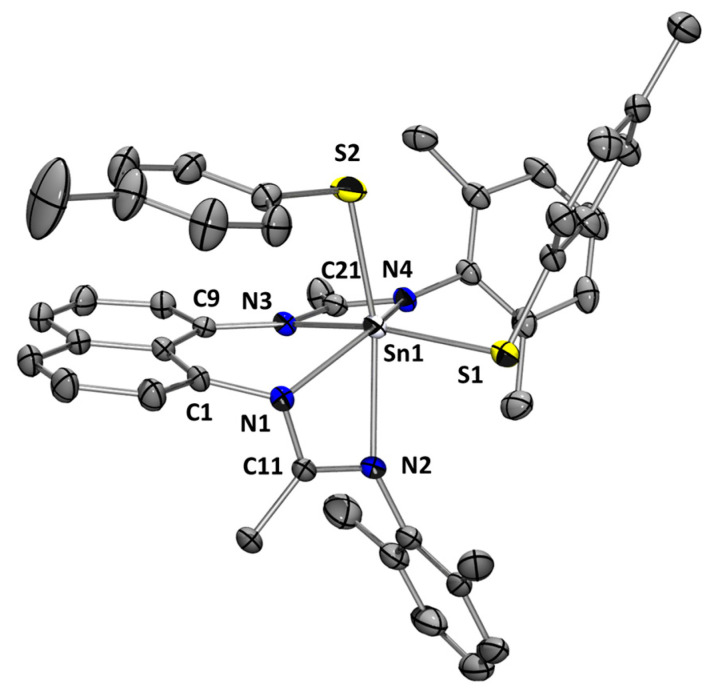
Molecular structure of **2a**. Thermal ellipsoids are represented with a 30% probability. Hydrogen atoms have been omitted for clarity. Selected bond distances [Å] and bond angles [deg]: Sn(1)-N(1) 2.132(3); Sn(1)-N(2) 2.411(3); Sn(1)-N(3) 2.230(3); Sn(1)-N(4) 2.180(3); Sn(1)-S(1) 2.4297(15); Sn(1)-S(2) 2.4483(16); C(11)-N(1) 1.361(4); C(11)-N(2) 1.301(4); C(21)-N(3) 1.334(4); C(21)-N(4) 1.337(4); N(1)-Sn(1)-N(4) 132.96(11); N(1)-Sn(1)-N(3) 78.01(11); N(3)-Sn(1)-N(4) 59.72(10); N(1)-Sn(1)-N(2) 58.27(11); N(2)-Sn(1)-N(4) 97.20(12); N(2)-Sn(1)-N(3) 86.71(11); N(1)-Sn(1)-S(1) 111.48(9); N(4)-Sn(1)-S(1) 102.83(8); N(3)-Sn(1)-S(1) 158.14(7); N(2)-Sn(1)-S(1) 82.44(9); N(1)-Sn(1)-S(2) 105.90(9); N(4)-Sn(1)-S(2) 97.26(9); N(3)-Sn(1)-S(2) 94.06(9); N(2)-Sn(1)-S(2) 163.67(8); S(1)-Sn(1)-S(2) 101.67(7); N(2)-C(11)-N(1) 113.5(3); N(3)-C(21)-N(4) 110.6(3).

**L_1_Sn** reacts with *p*-tolyldisulfide in THF at 70 °C overnight via an oxidative addition reaction with a tin insertion into the S–S bond (Scheme 8) to give a hexacoordinate Sn^(IV)^ species **2a** [22]. This molecule presents a characteristic ^119^Sn chemical shift at −456.7 ppm, consistent with a hexacoordinated tin center [24]. In addition, the X-ray diffraction analysis of **2a** reveals a monomeric Sn^(IV)^ species in a distorted octahedral coordination sphere that included the four nitrogen atoms of the two chelating amidinates and the two *p*-tolyl sulfur fragments (Figure 10). The equivalence of C–N bond lengths within NCN frameworks and their magnitudes (1.301 to 1.361 Å) indicate that the π electrons within the ligands are delocalized.

**Scheme 9:**
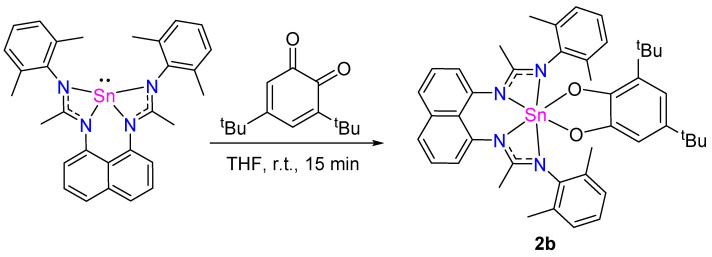
Synthesis of **2b**.

3,5-di-*tert*-butyl-ortho-quinone also reacts with **L_1_Sn** to afford the corresponding [4 + 1]-cycloadduct **2b** (Scheme 9). The ^1^H NMR spectrum reveals the characteristic signals of the quinone group in the aromatic region, doublets at 6.57 and 6.39 ppm (*J*_HH_ = 2.4 Hz), and two singlets at 1.06 and 1.16 ppm, corresponding to the CH_3_ groups of *^t^*butyl in the aliphatic area. Also, the signals corresponding to the amidine CH_3_ groups’ moieties integrating for 6 H as a singlet at 2.00 ppm and to the CH_3_ groups of 2,6-dimethylphenyl moieties integrating for 12 H as a singlet at 2.08 ppm, respectively, are observed. The ^119^Sn NMR spectrum shows a singlet at −512.2 ppm, in agreement with the cycloadduct structure. The mass analysis (DCI/CH_4_) spectrum exhibits a peak at 786.29, which corresponds to [M]^+^ of compound **2b**, evidencing its formation.

**Scheme 10:**
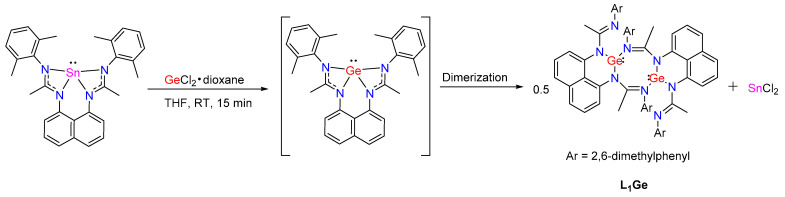
Synthesis of **L_1_Ge** by transmetalation reaction.

As reported by So et al., an amidinatogermylene can act as a good ligand to form a germylene→ECl_2_-type adduct with GeCl_2_ and SnCl_2_ [28]. In contrast, **L_1_Sn** reacts with GeCl_2_(dioxane) in THF at room temperature via a transmetalation reaction to give a germylene dimer (**L_1_Ge**)**_2_** (Scheme 10). The ^119^Sn NMR spectrum shows a resonance at −214.0 ppm, in agreement with the formation of SnCl_2_ [29]. 

**Scheme 11:**
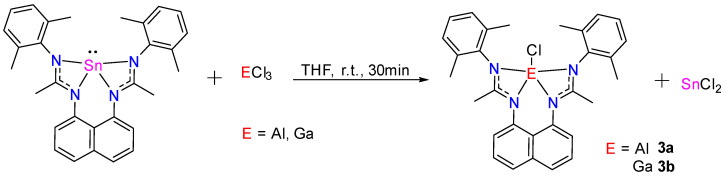
Synthesis of **3a** and **3b** via transmetalation reactions.

**Figure 11:**
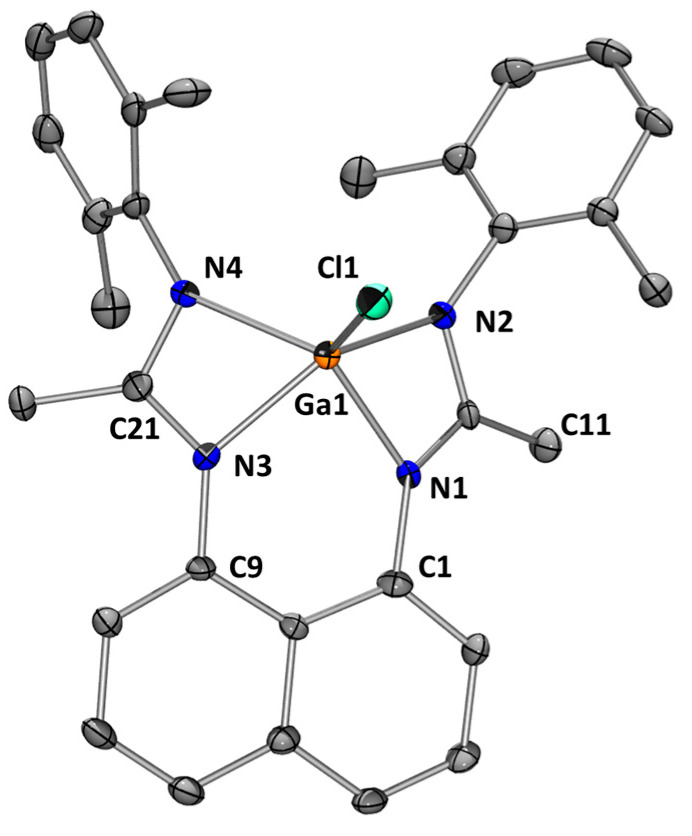
Molecular structure of **3b**. Thermal ellipsoids are represented with a 30% probability. Hydrogens have been omitted for clarity. Selected bond distances [Å] and bond angles [deg]: Ga(1)-N(1) 1.984(4); Ga(1)-N(2) 2.017(4); Ga(1)-N(3) 1.981(4); Ga(1)-N(4) 2.051(4); Ga(1)-Cl(1) 2.1828(14); N(1)-Ga(1)-N(3) 85.32(15); C(11)-N(1) 1.320(5); C(11)-N(2) 1.345(6); C(21)-N(3) 1.327(6); C(21)-N(4) 1.342(6); N(2)-Ga(1)-N(3) 139.06(16); N(1)-Ga(1)-N(2) 65.43(16); N(3)-Ga(1)-N(4) 65.69(15); N(1)-Ga(1)-N(4) 130.88(15); N(2)-Ga(1)-N(4) 112.06(16); N(3)-Ga(1)-Cl(1) 110.32(12); N(1)-Ga(1)-Cl(1) 115.30(11); N(2)-Ga(1)-Cl(1) 107.98(12); N(4)-Ga(1)-Cl(1) 111.79(12); N(1)-C(11)-N(2) 108.5(4); N(3)-C(21)-N(4) 110.1(4).

Transmetalation reactions were also observed with AlCl_3_ and GaCl_3_, affording the corresponding Al^(III)^ **3a** and Ga^(III)^-chlorides **3b**, and in both cases, forming one equivalent of SnCl_2_ (Scheme 11). The modeling confirms an exergonic reaction (−18.92 kcal/mol) during this “metal” exchange process. **L_1_Sn** reacts with AlCl_3_ quickly in THF at room temperature for 30 min to give **3a**, which was isolated as a white solid in 83% yield. The ^1^H NMR spectrum shows, in the aromatic region, the expected resonances and integrals, and in the aliphatic area, two singlets at 2.33 and 1.90 ppm, integrating for 12 H, corresponding to the methyl groups of 2,6-dimethylphenyl fragments and a singlet at 2.13 ppm (6 H) related to the -CH_3_ of the amidine groups. In the ^13^C NMR spectrum, the characteristic NCN signal is observed at 176.4 ppm. The symmetry of the ^1^H and ^13^C NMR spectra are in agreement with a pentacoordinated aluminum complex. Using the same experimental conditions, **L_1_Sn** also reacts with GaCl_3_ to give Ga^(III)^-chloride **3b**, which was isolated as colorless crystals from a pentane solution at −30 °C in 88% yield. Gallium chloride **3b** was fully characterized by NMR spectroscopy (very similar to that of **3a**) and by X-ray diffraction analysis. The molecular structure of **3b** (Figure 11) shows a pentacoordinated gallium center with a distorted square-based pyramidal geometry. The Ga–Cl bond distance of 2.183 Å is in the range expected for such compounds [30].

## 3. Materials and Methods

### 3.1. General Comments

All manipulations were performed under an inert argon or nitrogen atmosphere using standard Schlenk-line and glovebox techniques. Dry, oxygen-free solvents were employed. Reagents were obtained from commercial suppliers unless otherwise stated. *N*-(2,6-dimethylphenyl)acetamide [31], *N*-(2,6-dimethylphenyl)acetimidoyl chloride [29], *N*,*N*′-(naphthalene-1,8-diyl)bis(2,2-dimethylpropanamide) [13], *N*,*N*′(naphthalene-1,8-diyl)bis(2,2-dimethylpropanimidoyl chloride) [13], L_1_ [12], and L_2_ [13] were synthesized following reported procedures. The Lappert’s germanium(II) and tin(II) derivatives were prepared according to the literature procedures [32]. The 1D and 2D NMR spectra were recorded with the following spectrometers for ^1^H, ^13^C, and ^119^Sn: Bruker Avance II 300 MHz, Avance III HD 400 MHz, and Avance I and II 500 MHz spectrometers. The chemical shift has been counted positively versus the low field and expressed in part per million (ppm). The mass spectrometric analysis was performed using three techniques: direct chemical ionization (DCI-CH_4_) methods recorded on a GCT Premier Waters mass spectrometer (Headquarters, MN, USA); electrospray ionization (ESI, Los Angeles, CA, USA) recorded on a Waters Xevo G2 Q-TOF mass spectrometer; and a Maldi micro-MX micro–mass in a pyrene matrix (ratio of product/matrix 1/100). Melting points were measured with capillary Electrothermal Stuart SMP40 apparatus, and samples were prepared in the glovebox before the analysis. FT-IR spectra were measured on a ThermoNicolet 6700 (Waltham, MA, USA) Nexus and recovered in a solid state (KBr). Single-crystal X-ray data were collected at a low temperature (193(2)K) on a Bruker APEX II Quazar (Billerica, MA, USA) diffractometer equipped with a 30W air-cooled microfocus source [(**L_2_Sn**)**_2_, L_2_Ge, 2a** and **3b**] or on a Bruker D8 VENTURE diffractometer equipped with a PHOTON III detector [**L_3_H_2_, L_1_Sn**, (**L_1_Ge**)**_2_, 1a** and **1b**] using MoKα radiation (λ = 0.71037 Å). The structures were solved by the intrinsic phasing method (SHELXT) [33] and refined by the full-matrix least-squares method on F2 [34]. All non-H atoms were refined with anisotropic displacement parameters and all the hydrogen atoms were refined isotropically at calculated positions using a riding model. For **2a**, some solvent molecules were highly disordered and difficult to model correctly. Therefore, the SQUEEZE function of PLATON [35] was used to eliminate the contribution of the electron density of those solvent molecules from the intensity data. Calculations were performed with the Gaussian 16 suite of programs [36] using the density functional method B3LYP with dispersion (D3) [37,38,39,40]. Tin atoms were treated with a Stuttgart–Dresden pseudopotential in combination with its adapted basis set [41]. All other atoms have been described with a 6–31G(d,p) basis set. Geometry optimizations were carried out without any symmetry restrictions. Frequency calculations were undertaken to confirm the nature of the stationary points, yielding one imaginary frequency for transition states (TS) and all of them were positive for minima. The connectivity of the transition states and their adjacent minima was confirmed by intrinsic reaction coordinate (IRC) calculations [42,43].

### 3.2. Synthesis

**Synthesis of L_3_H_2_.** *p*-toluidine (591 mg, 5.6 mmol) was added to a solution of *N*,*N*′-(naphthalene-1,8-diyl)bis(2,2-dimethylpropanimidoyl chloride) (1 g, 2.8 mmol) in dry toluene (50 mL). Then, Et_3_N (559 mg, 5.6 mmol) was added to the mixture. The reaction was stirred for 4 h under reflux. All volatiles were removed under reduced pressure. The solid residue was taken up in 40 mL of Et_2_O and washed with 25 mL of a saturated solution of Na_2_CO_3_. Then, the organic layer was washed with water (3 × 30 mL) and dried over Na_2_SO_4_. After that, the solvent was removed, and the crude product was recrystallized with CH_2_Cl_2_/pentane (1:2). Yellow crystals were obtained (63% yield). **Melting point**: 116–122 °C. **^1^H NMR** (DMSO-d_6_, 500 MHz): δ 9.43 (s, 1H, NH); 8.50 (s, 1H, NH); 7.26 (d, *J*_HH_ = 6.5 Hz, 1H, aryl); 6.94 (d, *J*_HH_ = 7.9 Hz, 1H, aryl); 6.88–6.83 (m, 3H, aryl); 6.80 (m, 3H, aryl); 6.66 (d, *J*_HH_ = 8.1 Hz, 2H, aryl); 6.58–6.51 (m, 2H, aryl); 6.32–6.20 (m, 2H, aryl); 2.05 (s, 3H, CH_3_); 2.01 (s, 3H, CH_3_); 1.38 (s, 9H, C(CH_3_)_3_); 1.23 (s, 9H, C(CH_3_)_3_). **^13^C{^1^H} NMR** (DMSO-d_6_, 125 MHz): δ 161.3 (C=N); 160.7 (C=N); 147.2 (aryl, C); 146.5 (aryl, C); 138.6 (aryl, C); 137.2 (aryl, C); 135.6 (aryl, C); 131.2 (aryl, C); 130.4 (aryl, C); 128.7 (aryl, CH); 128.0 (aryl, CH); 127.7 (aryl, CH); 124.8 (aryl, CH); 121.4 (aryl, CH); 120.9 (aryl, CH); 119.8 (aryl, CH); 116.5 (aryl, C); 114.8 (aryl, CH); 28.8 (C(CH_3_)_3_); 28.3 (C(CH_3_)_3_); 20.3 (CH_3_). **IR** (KBr, cm^−1^): 3373 (˅NH); 1623 (˅C=N). **ESI** *m*/*z*: 504.33 ([M]^+^).

#### 3.2.1. General Synthetic Procedure of Metallylenes

THF (5 mL) was added to a mixture of one equiv. of MCl_2_ (M = Sn or Ge) and two equiv. of K[N(SiMe_3_)_2_]_2_ in a 20 mL vial inside the glovebox. The mixture was stirred for 30 min at room temperature. After this time, a white precipitate was obtained. The mixture was filtered, and the solution was added to one equiv. of the corresponding **L_1–3_H_2_**, stirring at 60 °C for 3 h. Then, all the volatiles were removed and the residual solid was washed with pentane (3 × 5 mL).

**L_1_Sn**. Colorless crystals were obtained by recrystallization from pentane at −30 °C (77% yield). **Melting point**: 202 °C (decomposition). **^1^H NMR** (THF-d_8_, 500 MHz): δ 7.42 (dd, *J*_HH_ = 8.2, 1.1 Hz, 2H, C_10_H_6_); 7.31–7.27 (m, 2H, C_10_H_6_); 7.07 (dd, *J*_HH_ = 7.5, 1.1 Hz, 2H, C_10_H_6_); 6.96 (d, *J*_HH_ = 7.4 Hz, 4H, C_6_H_3_); 6.83 (t, *J*_HH_ = 7.5 Hz, 2H, C_6_H_3_); 2.18 (s, 12H, CH_3_); 1.97 (s, 6H, CH_3_). **^13^C{^1^H} NMR** (THF-d_8_, 125 MHz): δ 169.0 (NCN); 145.3 (C_6_H_3ipso_); 144.5 (C_10_H_6ipso_); 138.6 (C_10_H_6ipso_); 134.4 (C_6_H_3ipso_); 128.8 (C_6_H_3_); 126.4 (C_10_H_6_); 124.8 (C_6_H_3_); 123.4 (C_10_H_6_); 119.8 (C_10_H_6_); 20.0 (CH_3_); 17.9 (CH_3_). **^119^Sn{^1^H} NMR** (THF-d_8_, 186 MHz): δ −276.7. **ESI** *m*/*z*: 565.15 ([M]^+^); 449.27 ([M − Sn]^+^).

(**L_2_Sn**)**_2_**. Yellow crystals (44% yield). **Melting point**: 243 °C (decomposition). **MS** (Maldi-TOF) *m*/*z*: 649.3 ([M/2, monomer]^+^). Meaningful solution state spectroscopic data for the compound could not be obtained for the compound as it shows negligible solubility in normal non-coordinating deuterated solvents once crystallized.

**L_3_Sn**. Yellow solid (61% yield). **Melting point**: 189 °C (decomposition). **^1^H NMR** (THF-d_8_, 400 MHz): δ 7.38 (d, *J*_HH_ = 7.5 Hz, 2H, C_10_H_6_); 7.24–7.19 (m, 2H, C_10_H_6_); 7.09 (d, *J*_HH_ = 7.1 Hz, 2H, C_10_H_6_); 7.05 (d, *J*_HH_ = 8.0 Hz, 4H, C_6_H_4_); 6.97 (d, *J*_HH_ = 8.2 Hz, 4H, C_6_H_4_); 2.29 (s, 6H, CH_3_); 1.30 (s, 18H, C(CH_3_)_3_). **^13^C{^1^H} NMR** (THF-d_8_, 125 MHz): δ 177.5 (NCN); 146.7 (C_6_H_4ipso_); 145.7 (C_10_H_6ipso_); 138.4 (C_10_H_6ipso_); 133.0 (C_6_H_4ipso_); 130.4 (C_6_H_4_); 126.3 (C_10_H_6_); 124.9 (C_6_H_4_); 123.4 (C_10_H_6_); 121.3 (C_10_H_6_); 43.6 (*C*(CH_3_)_3_); 31.8 (C(*C*H_3_)_3_); 21.0 (CH_3_). **^119^Sn{^1^H} NMR** (THF-d_8_, 186 MHz): δ −254.9. **MS** (Maldi-TOF) *m*/*z*: 621.2 ([M]^+^); 503.4 ([M–Sn]^+^).

(**L_1_Ge**)**_2_.** Yellow crystals (42% yield). **Melting point**: 259 °C (decomposition). **MS** (Maldi-TOF) *m*/*z*: 519.14 ([M/2, monomer]^+^). Meaningful solution state spectroscopic data for the compound could not be obtained for the compound as it shows negligible solubility in normal non-coordinating deuterated solvents once crystallized.

**L_2_Ge.** Toluene was used instead of THF as solvent. Yellow crystals were obtained by recrystallization from pentane at −30 °C. (52% yield). **Melting point**: 199 °C (decomposition). **^1^H NMR** (C_6_D_6_, 300 MHz): δ 6.98–6.91 (m, 2H, C_10_H_6_); 6.89–6.81 (m, 2H, C_10_H_6_); 6.62 (d, *J*_HH_ = 7.7 Hz, 4H, C_6_H_3_); 6.47–6.40 (m, 2H, C_6_H_3_); 6.33 (d, *J*_HH_ = 6.3 Hz, 2H. C_10_H_6_); 2.18 (s, 12H, CH_3_); 1.60 (s, 18H, C(CH_3_)_3_). **^13^C{^1^H} NMR** (C_6_D_6_, 125 MHz): δ 170.9 (NCN); 151.8 (C_6_H_3ipso_); 148.8 (C_10_H_6ipso_); 139.7 (C_10_H_6ipso_); 138.5 (C_6_H_3ipso_); 128.3 (C_6_H_3_); 126.3 (C_10_H_6_); 120.4 (C_6_H_3_); 114.9 (C_10_H_6_); 108.8 (C_10_H_6_); 41.9 (C(CH_3_)_3_); 30.6 (C(CH_3_)_3_); 19.7 (CH_3_).

**Synthesis of 1a.** In a J. Young valve NMR tube, a THF (0.4 mL) solution of **L_1_Sn** (30 mg, 0.053 mmol) was exposed to 5 bar of N_2_O. The reaction was monitored by NMR and proceeded quantitatively after 3 h at 70 °C. Colorless crystals were observed and separated from the solution by filtration. Then, the crystals were washed with pentane (3 × 2 mL). Colorless crystals were obtained (46% yield). **Melting point**: 204 °C. **^1^H NMR** (THF-d_8_, 500 MHz): δ 7.42 (d, *J*_HH_ = 6.4 Hz, 4H, C_10_H_6_); 7.26 (t, *J*_HH_ = 7.8 Hz; 4H, C_10_H_6_); 7.13 (d, *J*_HH_ = 6.8 Hz, 4H, C_10_H_6_), 7.01 (s, 4H, C_6_H_3_); 6.92–6.83 (m, 8H, C_6_H_3_); 2.07 (s, 12H, CH_3_); 1.85 (s, 12H, CH_3_); 1.76 (s, 12H, CH_3_). **^13^C{^1^H} NMR** (THF-d_8_, 125 MHz): δ 168.7 (NCN); 143.6 (C_6_H_3ipso_); 142.7 (C_10_H_6ipso_); 138.5 (C_10_H_6ipso_); 137.0 (C_10_H_6ipso_); 135.3 (C_6_H_3ipso_); 128.7 (C_6_H_3_); 128.3 (C_6_H_3_); 126.3 (C_10_H_6_); 125.7 (C_6_H_3_); 124.8 (C_10_H_6_); 120.6 (C_10_H_6_); 19.8 (CH_3_); 19.0 (CH_3_); 15.8 (CH_3_). **^119^Sn{^1^H} NMR** (THF-d_8_, 186 MHz): δ −494.5. **MS** (Maldi-TOF) *m*/*z*: 1161.26 ([M]^+^); 583.13 ([M/2, monomer]^+^).

#### 3.2.2. General Reactivity Evaluation Procedure

THF was added to a mixture of one equiv. of **L_1_Sn** and one equiv. of S_8_, *p*-tolyldisulfide, 3,5-di-tert-butyl-*o*-benzoquinone, AlCl_3_, or GaCl_3_, accordingly. The mixture was stirred for the corresponding time and temperature. Then, the solvent was removed and the residual solid was washed with pentane (3 × 0.5 mL).

**1b**. Stirred overnight at room temperature. Pale-yellow crystals were obtained by recrystallization from THF at −30 °C (51% yield). **Melting point**: 229 °C (decomposition). **^1^H NMR** (THF-d_8_, 300 MHz): δ 7.40 (d, *J*_HH_ = 7.5 Hz, 4H, C_10_H_6_); 7.22 (t, *J*_HH_ = 7.8 Hz, 4H, C_10_H_6_); 7.11–7.02 (m, 4H, C_10_H_6_); 6.93 (bs, 12H, C_6_H_3_); 2.11 (br s, 24H, CH_3_); 1.75 (s, 12H, CH_3_). **^13^C{^1^H} NMR** (THF-d_8_, 125 MHz): δ 167.4 (NCN); 142.7 (C_10_H_6ipso_); 138.2 (C_10_H_6ipso_); 135.9 (C_6_H_3ipso_); 128.8 (C_6_H_3_); 126.4 (C_10_H_6_); 125.7 (C_6_H_3_); 124.8 (C_10_H_6_); 121.5 (C_10_H_6_); 19.7 (CH_3_); 16.4 (CH_3_)). **^119^Sn{^1^H} NMR** (THF-d_8_, 186 MHz): δ −648.0. **MS** (Maldi-TOF) m/z: 1193.10 ([M]^+^); 597.04 ([M/2, monomer]^+^).

**2a.** Stirred overnight at 70 °C. Pale-brown crystals (51% yield). **Melting point**: 199 °C. **^1^H NMR** (THF-d_8_, 500 MHz): δ 7.40 (dd, *J*_HH_ = 8.1, 1.1 Hz, 2H, C_10_H_6_); 7.25–7.21 (m, 2H, C_10_H_6_); 7.17 (dd, *J*_HH_ = 7.5, 1.2 Hz, 2H, C_10_H_6_); 7.05–6.96 (m, 6H, C_6_H_3_); 6.63 (d, *J*_HH_ = 8.1 Hz, 4H, C_6_H_4_); 6.44 (d, *J*_HH_ = 7.9 Hz, 4H, C_6_H_4_); 2.19 (s, 12H, CH_3_); 2.09 (s, Sn satellite: *J*_HSn_ = 7.3 Hz, 6H, CH_3_); 1.79 (s, Sn satellite: *J*_HSn_ = 2.4 Hz, 6H, C_H3_). **^13^C{^1^H} NMR** (THF-d_8_, 125 MHz): δ 168.8 (NCN); 143.8 (Sn satellite: *J*_CSn_ = 5.2 Hz, C_10_H_6ipso_); 143.3 (Sn satellite: *J*_CSn_ = 7.2 Hz, C_6_H_3_); 138.4 (Sn satellite: *J*_CSn_ = 3.3 Hz, C_10_H_6ipso_); 136.1 (Sn satellite: *J*_CSn_ = 12.8 Hz, C_6_H_4_); 135.6 (Sn satellite: *J*_CSn_ = 3.1 Hz, C_6_H_3_); 135.5 (C_6_H_4ipso_); 131.4 (C_6_H_4ipso_); 129.2 (C_6_H_3_); 129.0 (Sn satellite: *J*_CSn_ = 6.6 Hz, C_6_H_4_); 127.6 (C_10_H_6ipso_); 126.4 (C_10_H_6_); 126.4 (C_6_H_3_); 125.2 (C_10_H_6_); 121.1 (Sn satellite: *J*_CSn_ = 11.2 Hz, C_10_H_6_); 21.1 (CH_3_); 19.9 (CH_3_); 16.4 (Sn satellite: *J*_CSn_ = 21.40 Hz, CH_3_). **^119^Sn{^1^H} NMR** (THF-d_8_, 186 MHz): δ −456.7. **MS** (Maldi-TOF) *m*/*z*: 687.13 ([M − S(tolyl)]^+^); 565.05 ([M − 2 S(tolyl)]^+^).

**2b.** Stirred for 15 min at room temperature. White solid (96% yield). **Melting point**: 240 °C (decomposition). **^1^H NMR** (THF-d_8_, 500 MHz): δ 7.65–7.62 (m, 2H, C_10_H_6_); 7.44–7.39 (m, 4H, C_10_H_6_); 6.98–6.92 (m, 6H, C_6_H_3_); 6.57 (d, *J*_HH_ = 2.4 Hz, 1H, C_6_H_2_); 6.39 (d, *J*_HH_ = 2.4 Hz, 1H, C_6_H_2_); 2.08 (s, 12H, CH_3_); 2.00 (s, Sn satellite: *J*_HSn_ = 3.5 Hz, 6H, CH_3_); 1.16 (s, 9H, C(CH_3_)_3_); 1.06 (s, 9H, C(CH_3_)_3_). **^13^C{^1^H} NMR** (THF-d_8_, 125 MHz): δ 171.6 (NCN); 151.3 (Sn satellite: *J*_CSn_ = 5.1 Hz, C_6_H_2ipso_); 147.1 (Sn satellite: *J*_CSn_ = 3.3 Hz, C_6_H_2ipso_); 142.0 (Sn satellite: *J*_CSn_ = 3.9 Hz, C_10_H_6ipso_); 140.9 (Sn satellite: *J*_CSn_ = 7.5 Hz, C_6_H_3ipso_); 138.7 (C_6_H_2ipso_); 138.5 (C_10_H_6ipso_); 136.1 (C_6_H_3ipso_); 134.1 (C_6_H_2ipso_); 129.1 (C_6_H_3_); 127.1 (C_6_H_3_); 126.7 (C_10_H_6_); 126.1 (C_10_H_6ipso_); 126.0 (C_10_H_6_); 121.2 (Sn satellite: *J*_CSn_ = 11.6 Hz, C_10_H_6_); 111.5 (C_6_H_2_); 110.3 (Sn satellite: *J*_CSn_ = 40.2 Hz, C_6_H_2_); 35.3 (*C*(CH_3_)_3_); 34.9 (*C*(CH_3_)_3_); 32.4 (C(*C*H_3_)_3_); 30.2 (C(*C*H_3_)_3_); 19.1 (CH_3_); 15.5 (Sn satellite: *J*_CSn_ = 33.7 Hz, CH_3_). **^119^Sn{^1^H} NMR** (THF-d_8_, 186 MHz): δ −512.2. **ESI** *m*/*z*: 786.29 ([M]^+^).

**3a.** Stirred for 30 min at room temperature. White solid (85% yield). **Melting point**: 124 °C. **^1^H NMR** (THF-d_8_, 500 MHz): δ 7.46 (dd, *J*_HH_ = 8.3, 1.1 Hz, 2H, C_10_H_6_); 7.35–7.31 (m, 2H, C_10_H_6_); 7.24 (dd, *J*_HH_ = 7.5, 1.2 Hz, 2H, C_10_H_6_); 7.02–6.90 (m, 6H, C_6_H_3_); 2.33 (s, 6H, CH_3_); 2.13 (s, 6H, CH_3_); 1.90 (s, 6H, CH_3_). **^13^C{^1^H} NMR** (THF-d_8_, 125 MHz): δ 176.4 (NCN); 141.9 (C_10_H_6ipso_); 141.8 (C_10_H_6ipso_); 140.6 (C_6_H_3ipso_); 138.4 (C_10_H_6ipso_); 135.8 (C_6_H_3ipso_); 135.1 (C_6_H_3ipso_); 129.4 (C_6_H_3_); 128.8 (C_6_H_3_); 126.6 (C_10_H_6_); 126.2 (C_6_H_3_); 124.1 (C_10_H_6_); 123.9 (C_10_H_6ipso_); 117.8 (C_10_H_6_); 19.9 (CH_3_); 19.3 (CH_3_); 15.6 (CH_3_). **^27^Al{^1^H} NMR** (THF-d_8_, 130 MHz): δ 69.4.

**3b.** Stirred for 30 min at room temperature. Colorless crystals were obtained from recrystallization with pentane at −30 °C (88% yield). **Melting point**: 209 °C (decomposition). **^1^H NMR** (THF-d_8_, 500 MHz): δ 7.46 (dd, *J*_HH_ = 8.3, 1.1 Hz, 2H, C_10_H_6_); 7.34–7.30 (m, 2H, C_10_H_6_); 7.20 (dd, *J*_HH_ = 7.5, 1.2 Hz, 2H, C_10_H_6_); 7.03–6.90 (m, 6H, C_6_H_3_); 2.34 (s, 6H, CH_3_); 2.12 (s, 6H, CH_3_); 1.98 (s, 6H, CH_3_). **^13^C{^1^H} NMR** (THF-d_8_, 125 MHz): δ 172.3 (NCN); 142.9 (C_10_H_6ipso_); 141.9 (C_6_H_3ipso_); 138.6 (C_10_H_6ipso_); 136.1 (C_6_H_3ipso_); 135.5 (C_6_H_3ipso_); 128.8 (C_6_H_3_); 126.4 (C_10_H_6_); 126.4 (C_6_H_3_); 124.3 (C_10_H_6_); 123.1 (C_10_H_6ipso_); 118.3 (C_10_H_6_); 19.8 (CH_3_); 15.1 (CH_3_). **MS** (Maldi-TOF) *m*/*z*: 551.06 ([M]^+^); 517.04 ([M − Cl]^+^); 449.10 ([M − GaCl]^+^).

### 3.3. X-ray Data

CCDC-2303009 (**L_3_H_2_**), CCDC-2266345 (**L_1_Sn**), CCDC-2266346 [(**L_2_Sn**)**_2_**], CCDC-2266347 [(**L_1_Ge**)**_2_**], CCDC-2266348 (**L_2_Ge**), CCDC-2266349 (**1a**), CCDC-2266350 (**1b**), CCDC-2266351 (**2a**), and CCDC-2266352 (**3b**) contain the supplementary crystallographic data for this paper. These data can be obtained free of charge from The Cambridge Crystallographic Data Centre via www.ccdc.cam.ac.uk/data_request/cif.

## 4. Conclusions

Three tetradentate bis(amidine) ligands RNC(R′)N-linker-NC(R′)NR with a rigid naphthalene linker **L_1–3_** were successfully used for the stabilization of metallylenes. The corresponding stannylenes and germylenes (**LSn** and **LGe**) have been prepared by protonolysis reaction of Lappert’s metallylenes [M(HMDS)_2_] (M = Ge or Sn) and were fully characterized by NMR spectroscopy and X-ray diffraction analysis. Structures in the solid state show either a monomer or a dimer depending on the different substituents of the amidine moiety, demonstrating that the nature of the bis(amidine) system can influence the stabilization and the reactivity of the corresponding tetrylenes. DFT calculations have been performed in order to define the electronic properties associated with tetradentate ligands. The reactivity of stannylene **L_1_Sn** was explored in oxidation, oxidative addition, and transmetalation reactions to form in particular the corresponding gallium and aluminum derivatives.

## Data Availability

The data presented in this study are available in article and Supplementary Materials.

## Supplementary Materials

The following supporting information can be downloaded at: https://www.mdpi.com/article/10.3390/molecules29020325/s1, NMR spectra, crystal structure refinements, and computational investigations (PDF).
