# Supplementary material for: Stannylenes and Germylenes Stabilized by Tetradentate Bis(amidine) Ligands with a Rigid Naphthalene Backbone
Source: Molecules. 2024 Jan 9;29(2):325. doi: 10.3390/molecules29020325 (PMC10819778; doi:10.3390/molecules29020325)
Supplement: Supplementary file 1 [file molecules-29-00325-s001.zip › molecules-2805737-supplementary.pdf]

Supporting Information

Table of contents

General information. 3
^{1}H NMR spectrum of compound L_3H_2 (DMSO-d_6, 500 MHz) 5
$^{13}C\{^{1}H\}$ NMR spectrum of compound L_3H_2 (DMSO-d_6, 125 MHz) 6
^{1}H NMR spectrum of compound L_1Sn (THF-d_8, 500 MHz) 7
$^{13}C\{^{1}H\}$ NMR spectrum of compound L_1Sn (THF-d_8, 125 MHz) 8
$^{119}Sn\{^{1}H\}$ NMR spectrum of compound L_1Sn (THF-d_8, 186 MHz) 9
^{1}H NMR spectrum of compound L_3Sn (THF-d_8, 400 MHz) 10
$^{13}C\{^{1}H\}$ NMR spectrum of compound L_3Sn (THF-d_8, 125 MHz) 11
$^{119}Sn\{^{1}H\}$ NMR spectrum of compound L_3Sn (THF-d_8, 186 MHz) 12
^{1}H NMR spectrum of compound L_2Ge (C_6D_6, 300 MHz) 13
$^{13}C\{^{1}H\}$ NMR spectrum of compound L_2Ge (C_6D_6, 125 MHz) 14
^{1}H NMR spectrum of compound 1a (THF-d_8, 500 MHz) 15
$^{13}C\{^{1}H\}$ NMR spectrum of compound 1a (THF-d_8, 125 MHz) 16
$^{119}Sn\{^{1}H\}$ NMR spectrum of compound 1a (THF-d_8, 186 MHz) 17
^{1}H NMR spectrum of compound 1b (THF-d_8, 300 MHz) 18
$^{13}C\{^{1}H\}$ NMR spectrum of compound 1b (THF-d_8, 125 MHz) 19
$^{119}Sn\{^{1}H\}$ NMR spectrum of compound 1b (THF-d_8, 186 MHz) 20
^{1}H NMR spectrum of compound 2a (THF-d_8, 500 MHz) 21
$^{13}C\{^{1}H\}$ NMR spectrum of compound 2a (THF-d_8, 125 MHz) 22
$^{119}Sn\{^{1}H\}$ NMR spectrum of compound 2a (THF-d_8, 186 MHz) 23
^{1}H NMR spectrum of compound 2b (THF-d_8, 500 MHz) 24
$^{13}C\{^{1}H\}$ NMR spectrum of compound 2b (THF-d_8, 125 MHz) 25
$^{119}Sn\{^{1}H\}$ NMR spectrum of compound 2a (THF-d_8, 186 MHz) 26
^{1}H NMR spectrum of compound 3a (THF-d_8, 500 MHz) 27
$^{13}C\{^{1}H\}$ NMR spectrum of compound 3a (THF-d_8, 125 MHz) 28
$^{27}Al\{^{1}H\}$ NMR spectrum of compound 3a (THF-d_8, 130 MHz) 29
^{1}H NMR spectrum of compound 3b (THF-d_8, 500 MHz) 30
$^{13}C\{^{1}H\}$ NMR spectrum of compound 3b (THF-d_8, 125 MHz) 31
Crystal data and structure refinement of compound L_3H_2 32

Crystal data and structure refinement of compound L_1Sn 41
Crystal data and structure refinement of compound $(L_2Sn)_2$ 49
Crystal data and structure refinement of compound $(L_1Ge)_2$ 64
Crystal data and structure refinement of compound L_2Ge 76
Crystal data and structure refinement of compound 1a 85
Crystal data and structure refinement of compound 1b 95
Crystal data and structure refinement of compound 2a 111
Crystal data and structure refinement of compound 3b 123
Calculations 132

General information.

All manipulations were performed under an inert argon or nitrogen atmosphere using standard Schlenk-line and glovebox techniques. Dry, oxygen-free solvents were employed. Reagents were obtained from commercial suppliers unless otherwise stated. *N*-(2,6-dimethylphenyl)acetamide,[1] *N*-(2,6-dimethylphenyl)acetimidoyl chloride,[1] *N*,*N*'-(naphthalene-1,8-diyl)bis(2,2-dimethylpropanamide),[2] *N*,*N*'(naphthalene-1,8-diyl)bis(2,2-dimethylpropanimidoyl chloride),[2] L_1[3], and L_2[2] were synthesized following reported procedures. The Lappert germanium(II) and tin (II) derivatives were prepared according to literature procedures.[4] 1D and 2D NMR spectra were recorded with the following spectrometers for ^{1}H, ^{13}C and ^{119}Sn: Bruker Avance II 300MHz, Avance III HD 400 MHz, and Avance I and II 500 MHz spectrometers. The chemical shift has been counted positively verse the low field and expressed in part per million (ppm). The mass spectrometric analysis was done using three techniques, direct chemical ionization (DCI-CH_4) methods and recorded on a GCT Premier Waters mass spectrometer; electrospray ionization (ESI), recorded on a Waters Xevo G2 Q-TOF mass spectrometer; and a Maldi micro-MX micro–Mass in a pyrene matrix (ratio product/matrix:1/100). Melting points were measured with a capillary Electrothermal Stuart SMP40 apparatus, and samples were prepared in the glovebox before the analysis. FT-IR spectra were measured on a ThermoNicolet 6700, Nexus and recovered in solid state (KBr). Single-crystal X-ray data were collected at low temperature (193(2)K) on a Bruker APEX II Quazar diffractometer equipped with a 30W air-cooled microfocus source [**(L2Sn)2, L_2Ge, 2a** and **3b**] or on a Bruker D8 VENTURE diffractometer equipped with a PHOTON III detector [**L_3H_2**, **L_1Sn**, **(L_1Ge)$_2$, 1a** and **1b**], using MoKα radiation (λ= 0.71037 Å). The structure were solved by intrinsic phasing method (SHELXT)[5] and refined by full-matrix least- squares method on F2.[6] All non-H atoms were refined with anisotropic displacement parameters and all the hydrogen atoms were refined isotropically at calculated positions using a riding model. For **2a**, some solvent molecules were highly disordered and difficult to model correctly. Therefore the SQUEEZE function of PLATON[7] was used to eliminate the contribution of the electron density of those solvent molecules from the intensity data.

CCDC-2303009 (**L_3H_2**), CCDC-2266345 (**L_1Sn**), CCDC-2266346 [**(L_2Sn)$_2$**], CCDC-2266347 [**(L_1Ge)$_2$**], CCDC-2266348 (**L_2Ge**), CCDC-2266349 (**1a**), CCDC-2266350 (**1b**) CCDC-2266351 (**2a**) and CCDC-2266352 (**3b**) contain the supplementary

crystallographic data for this paper. These data can be obtained free of charge from The Cambridge Crystallographic Data Centre via www.ccdc.cam.ac.uk/data_request/cif.

(1) Boeré, R. T.; Klassen, V.; Wolmershäuser, G. Synthesis of Some Very Bulky N,N′-Disubstituted Amidines and Initial Studies of Their Coordination Chemistry†. *J. Chem. Soc. Dalton Trans.* **1998**, No. 24, 4147–4154. https://doi.org/10.1039/A805548C.

(2) Yakovenko, M. V.; Cherkasov, A. V.; Fukin, G. K.; Cui, D.; Trifonov, A. A. Lanthanide Complexes Coordinated by a Dianionic Bis(Amidinate) Ligand with a Rigid Naphthalene Linker. *Eur. J. Inorg. Chem.* **2010**, *2010* (21), 3290–3298. https://doi.org/10.1002/ejic.201000330

(3) Saltarini, S.; Villegas-Escobar, N.; Martínez, J.; Daniliuc, C. G.; Matute, R. A.; Gade, L. H.; Rojas, R. S. Toward a Neutral Single-Component Amidinate Iodide Aluminum Catalyst for the CO2 Fixation into Cyclic Carbonates. *Inorg. Chem.* **2021**, *60* (2), 1172–1182. https://doi.org/10.1021/acs.inorgchem.0c03290

(4) Davidson, P.; Harris, D.; Lappert, M. Subvalent Group 4B metal alkyls and amides. Part I. The synthesis and physical properties of kinetically stable bis[bis(trimethysilyl)methyl]-germanium(II), -tin(II), and -lead(II). *J. Chem. Soc., Chem. Dalton Trans.* **1976**, 2268-2274**.** https://doi.org/10.1039/DT9760002268

(5) Sheldrick, G. M. SHELXT - Integrated space-group and crystal-structure determination*, Acta Crystallogr. Sect. A,* **2015**, *71*, 3-8. https://doi.org/10.1107/S2053273314026370

(6) Sheldrick, G. M. Crystal structure refinement with SHELXL. *Acta Crystallogr. Sect. C*, **2015**, *71*, 3-8. https://doi.org/10.1107/S2053229614024218

(7) Van der Sluis, P.; Spek, A. L. BYPASS: an effective method for the refinement of crystal structures containing disordered solvent regions. *Acta Cryst., Sect. A*, **1990**, 46, 194-201. https://doi.org/10.1107/S0108767389011189

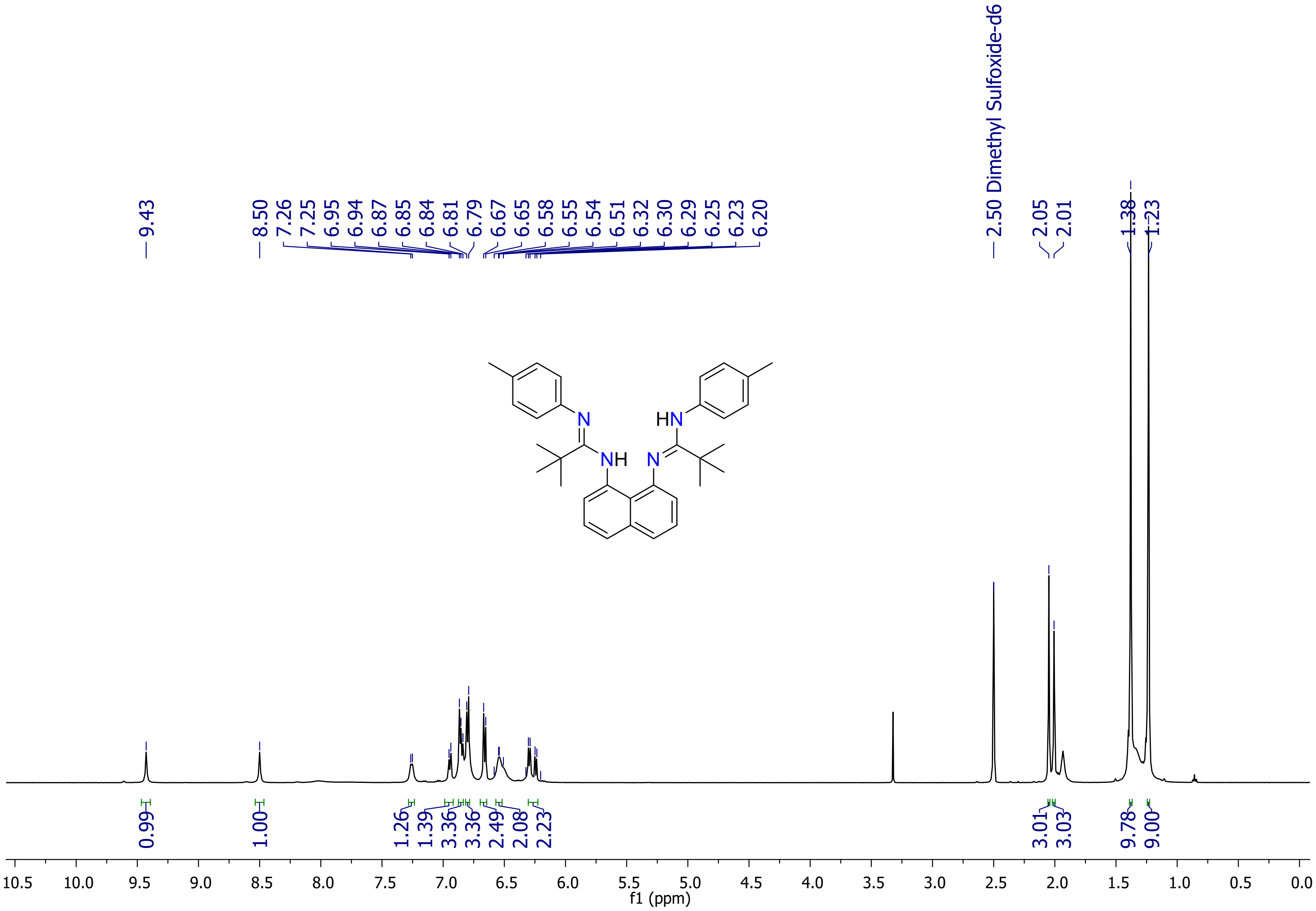

^{1}H NMR spectrum of compound $\mathbf{L_3H_2}$ (DMSO-d_6, 500 MHz)

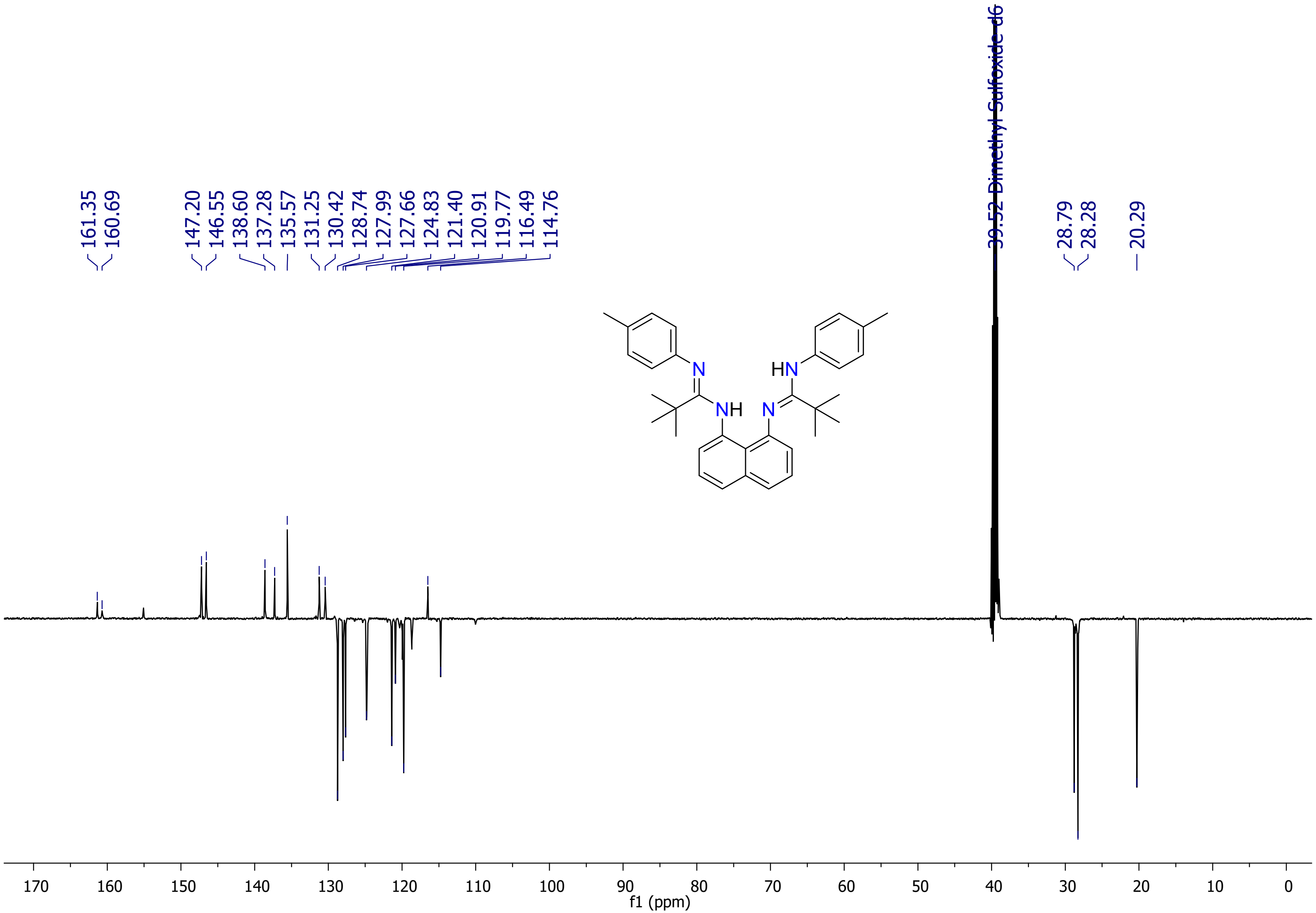

$^{13}C\{^{1}H\}$ NMR spectrum of compound $\mathbf{L_3H_2}$ (DMSO-d_6, 125 MHz)

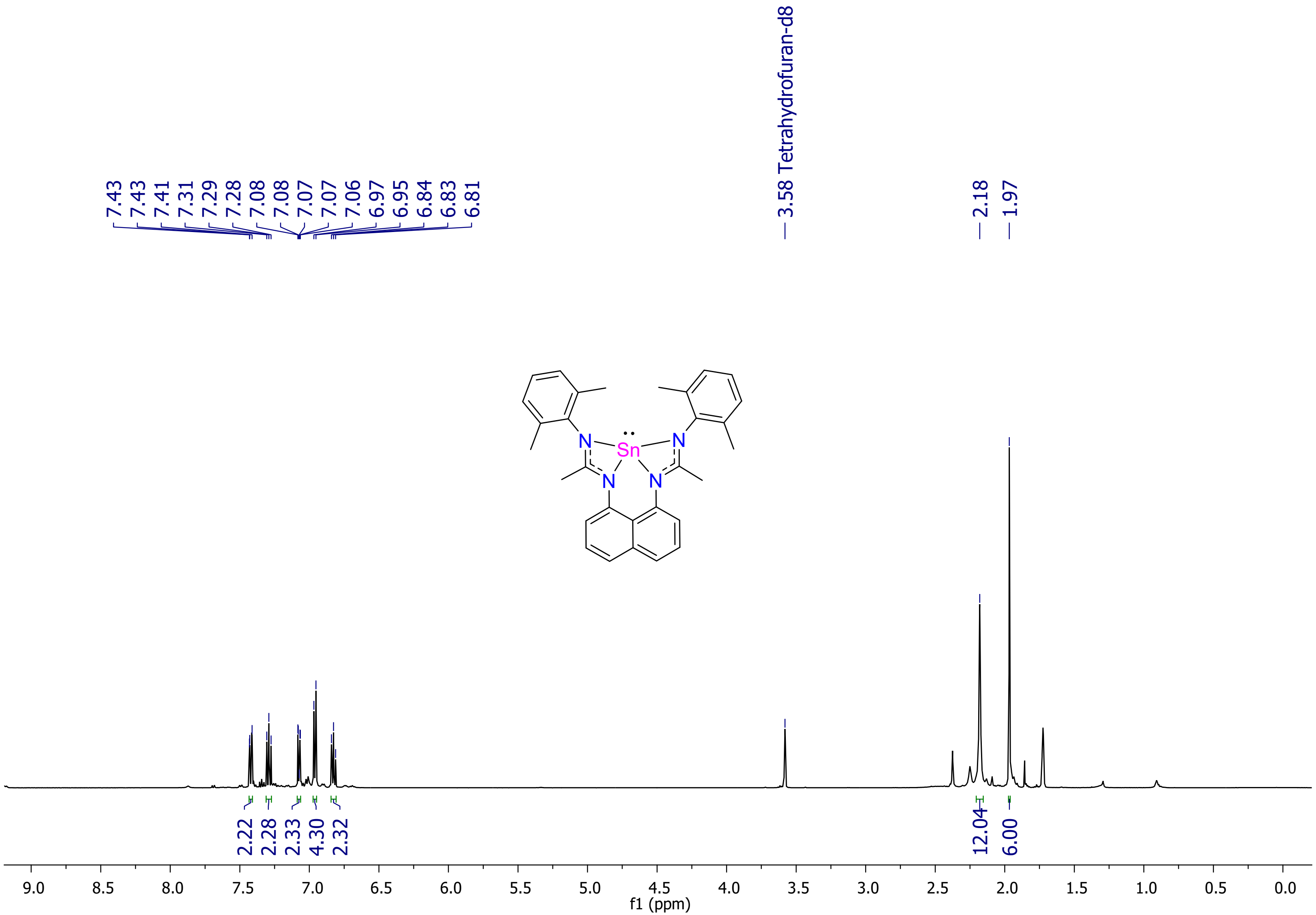

^{1}H NMR spectrum of compound $\mathbf{L_1Sn}$ (THF-d_8, 500 MHz)

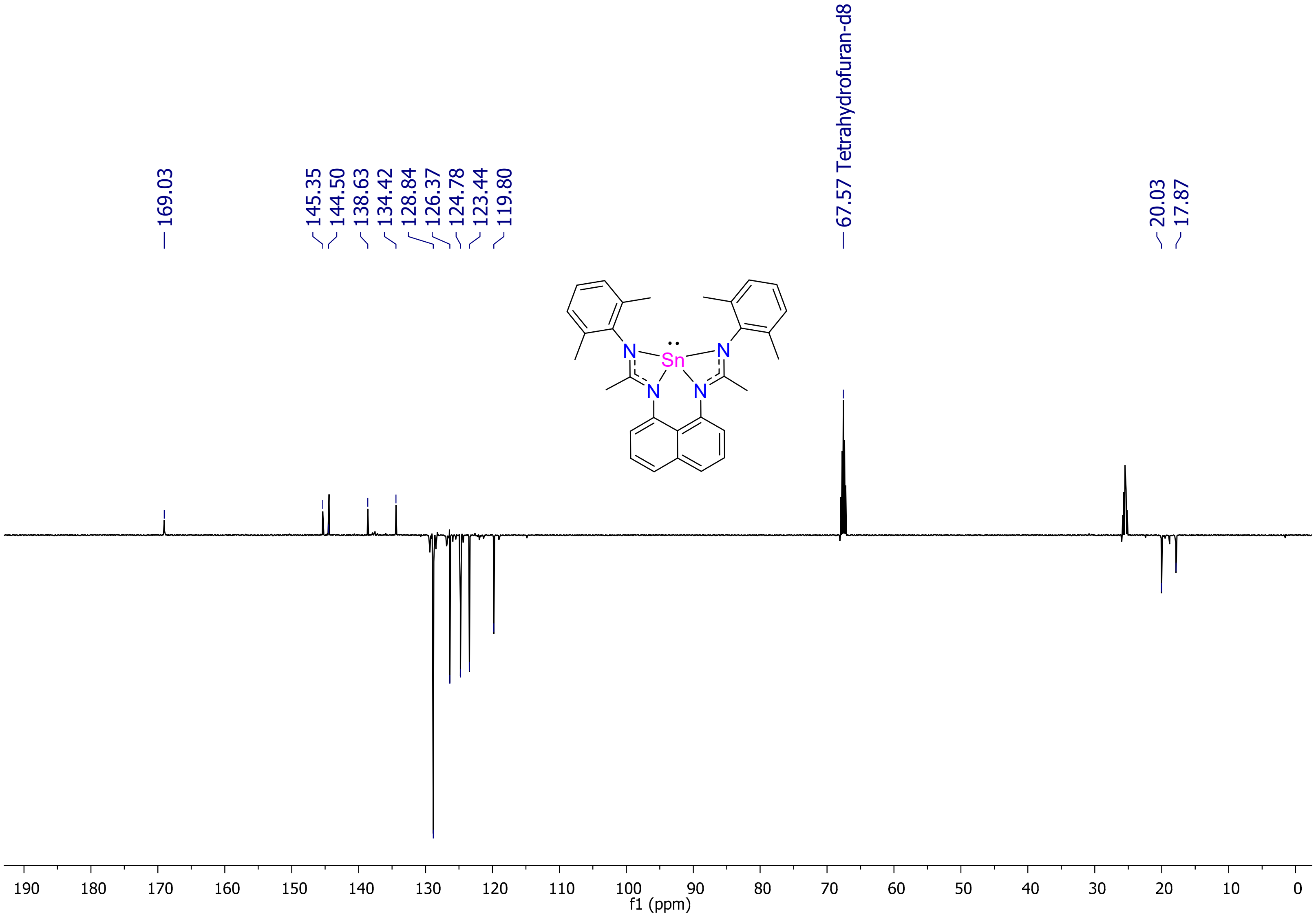

$^{13}C\{^{1}H\}$ NMR spectrum of compound **L_1Sn** (THF-d_8, 125 MHz)

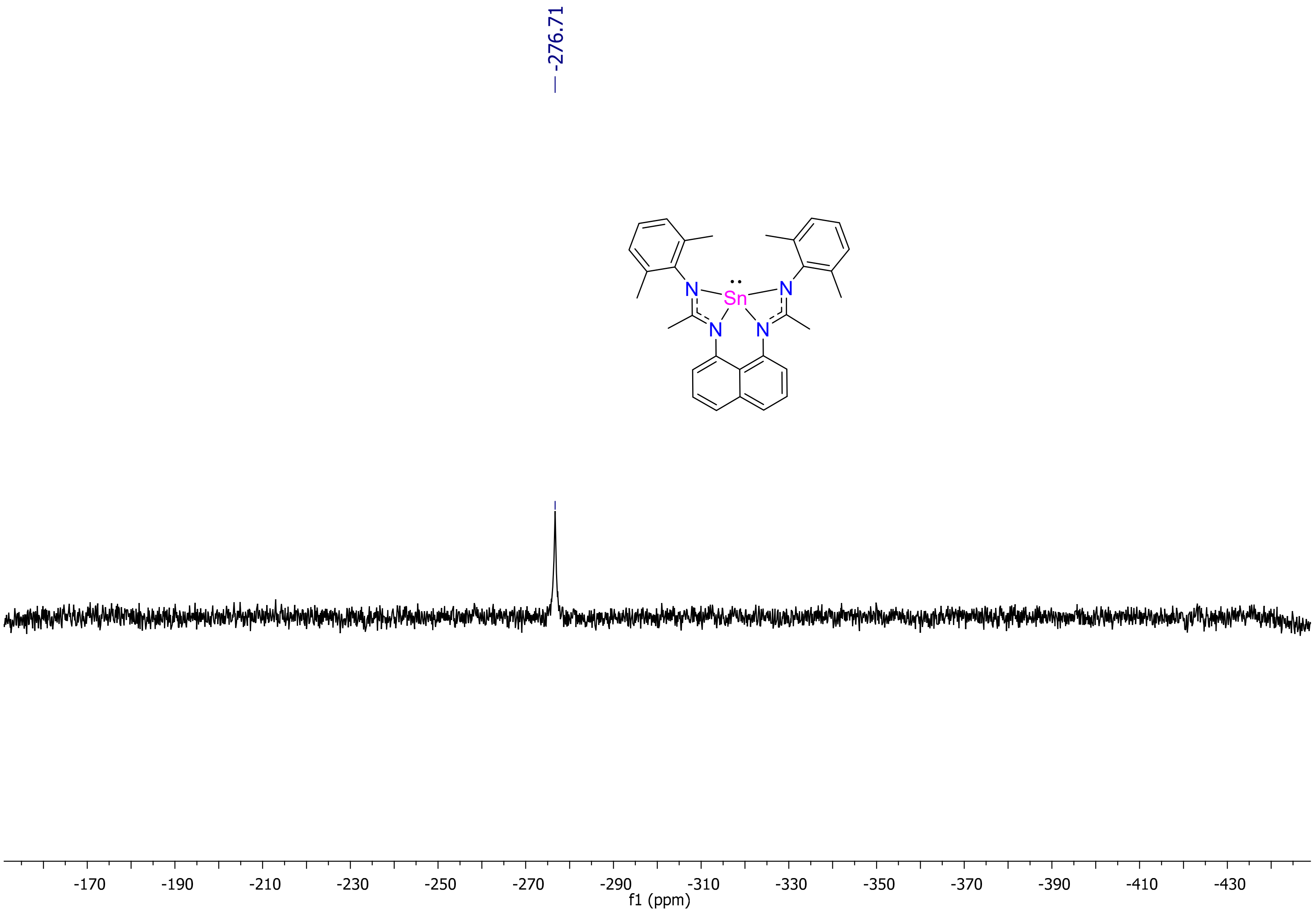

$^{119}Sn\{^1H\}$ NMR spectrum of compound $\mathbf{L_1Sn}$ (THF-d_8, 186 MHz)

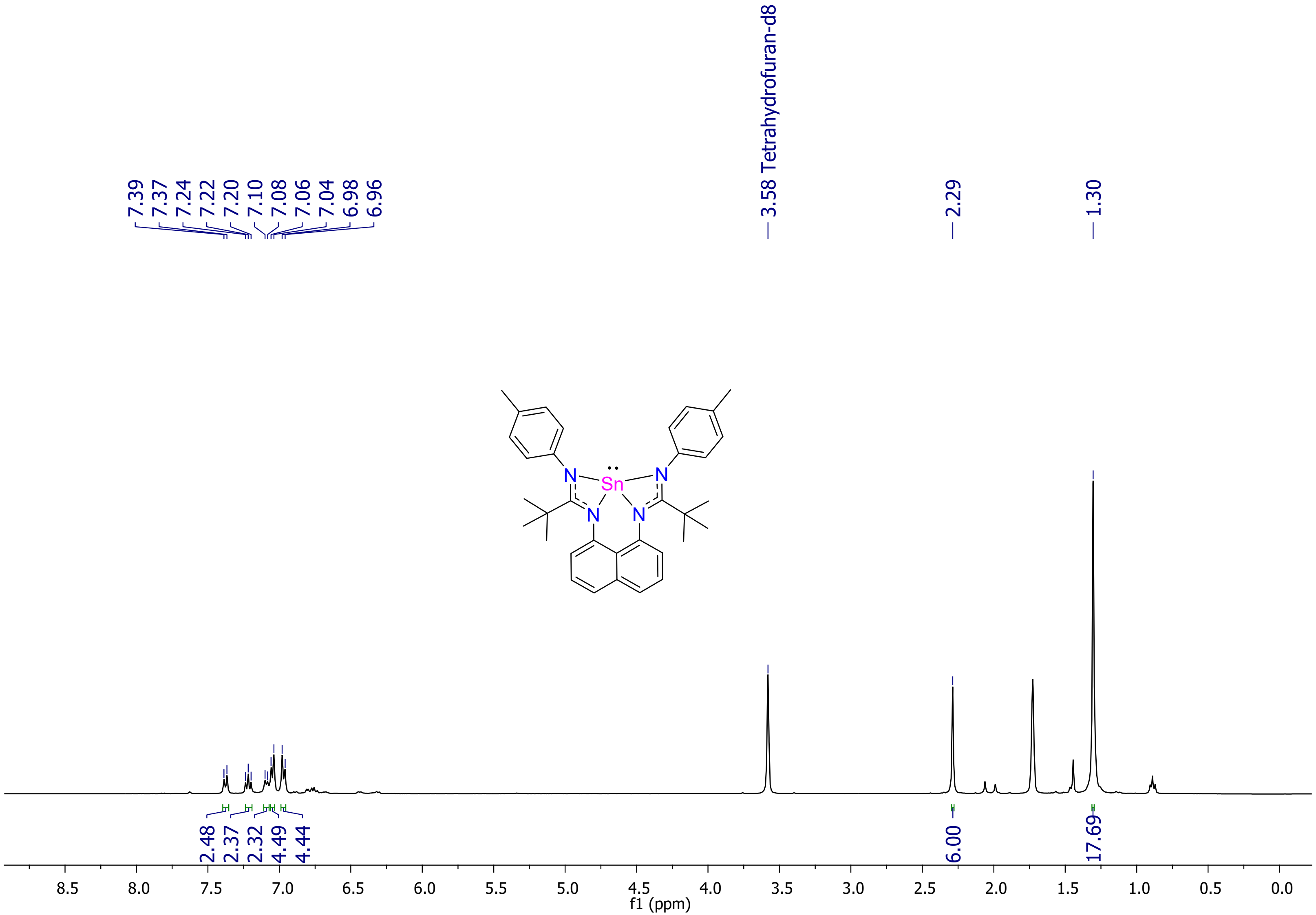

^{1}H NMR spectrum of compound **L_3Sn** (THF-d_8, 400 MHz)

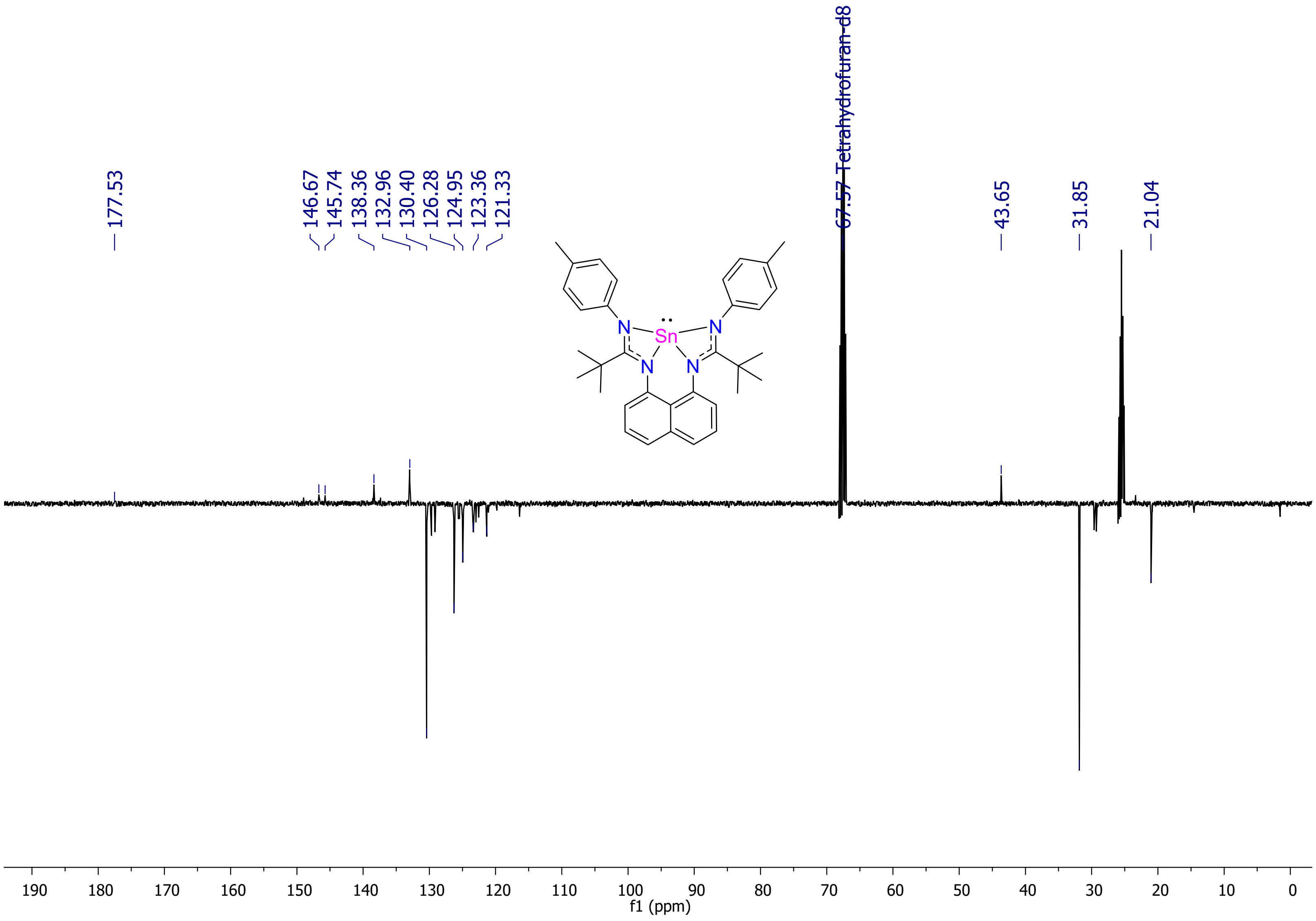

$^{13}C\{^{1}H\}$ NMR spectrum of compound **L_3Sn** (THF-d_8, 125 MHz)

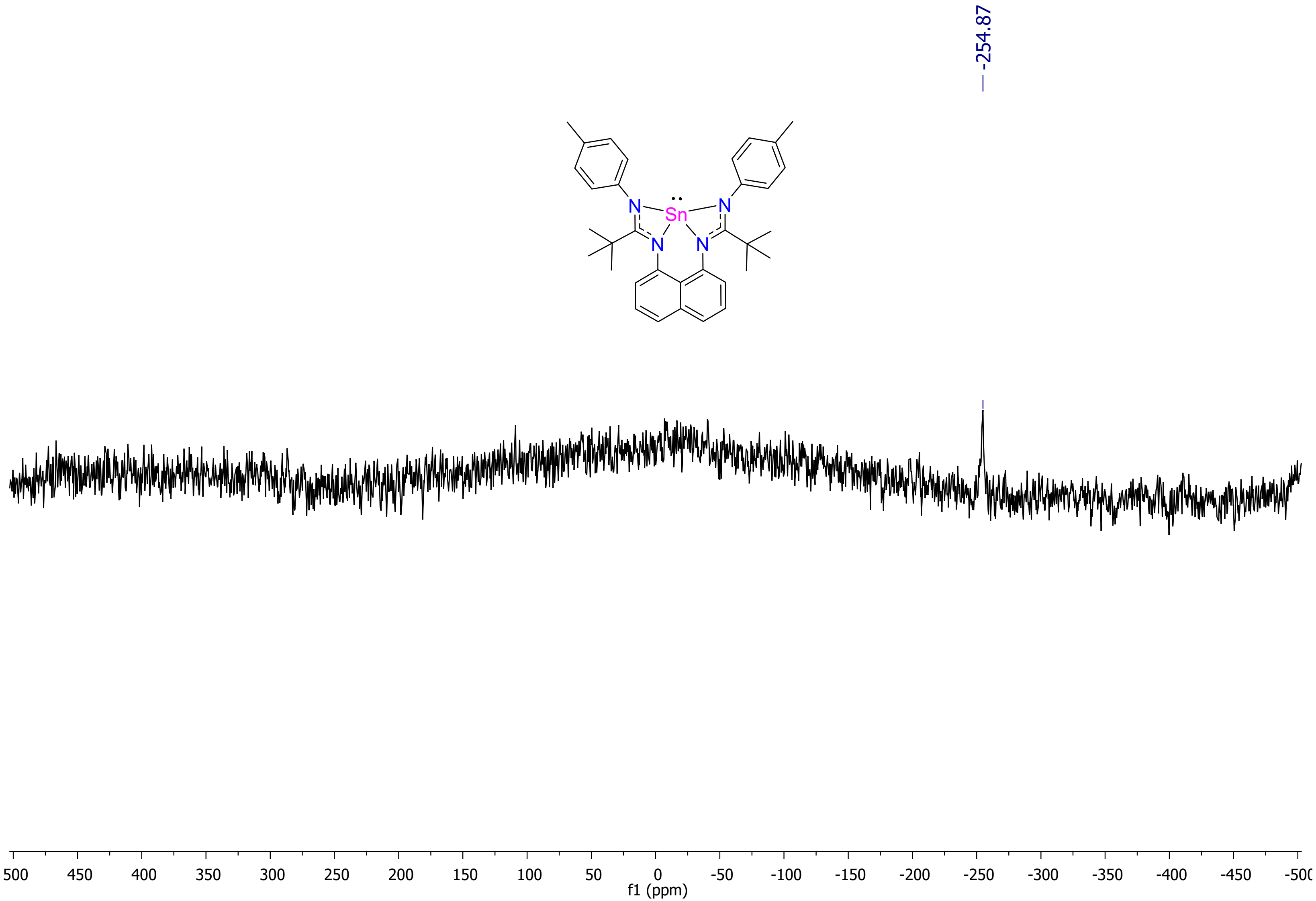

$^{119}Sn\{^{1}H\}$ NMR spectrum of compound **L_3Sn** (THF-d_8, 186 MHz)

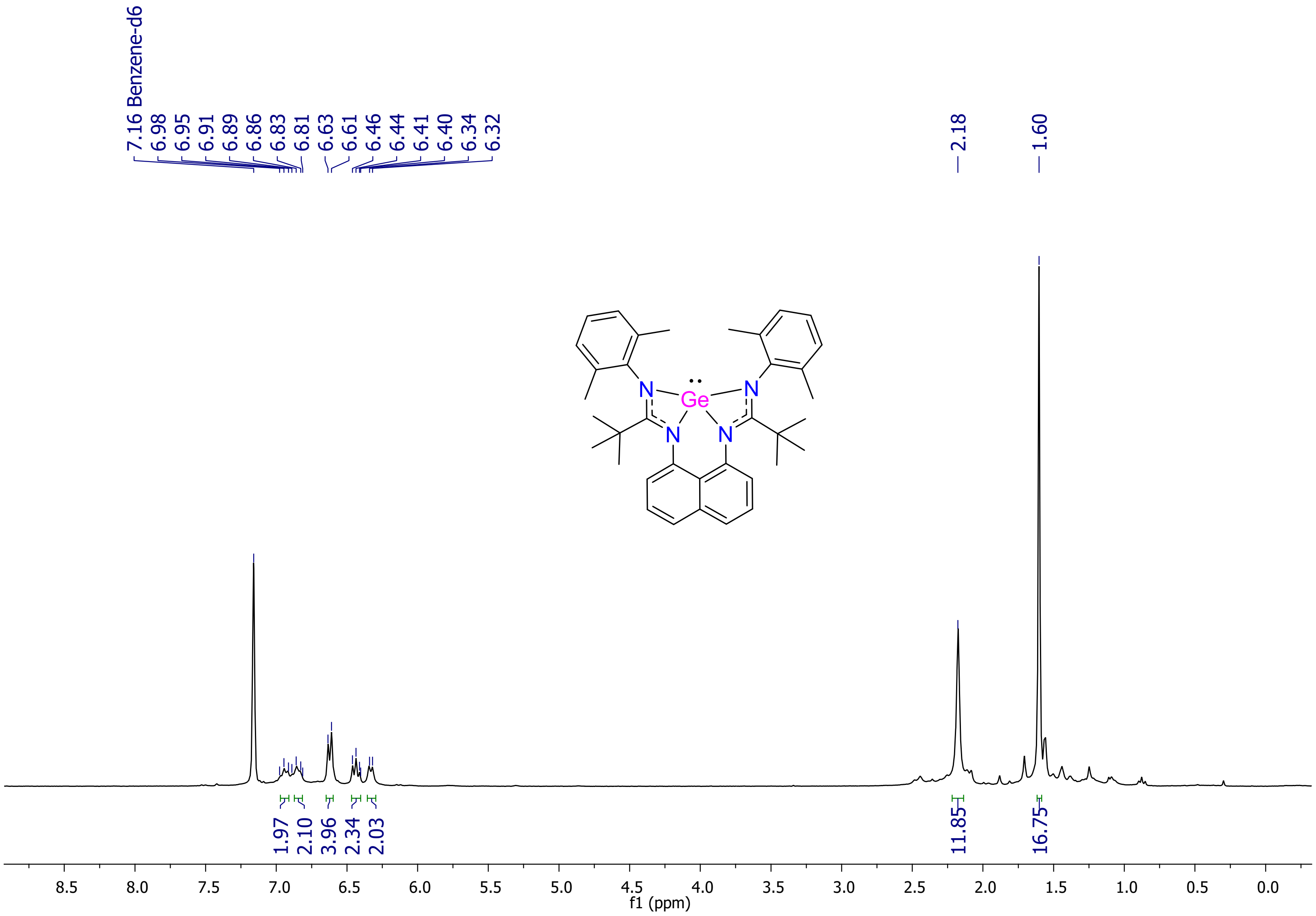

^{1}H NMR spectrum of compound **L_2Ge** (C_6D_6, 300 MHz)

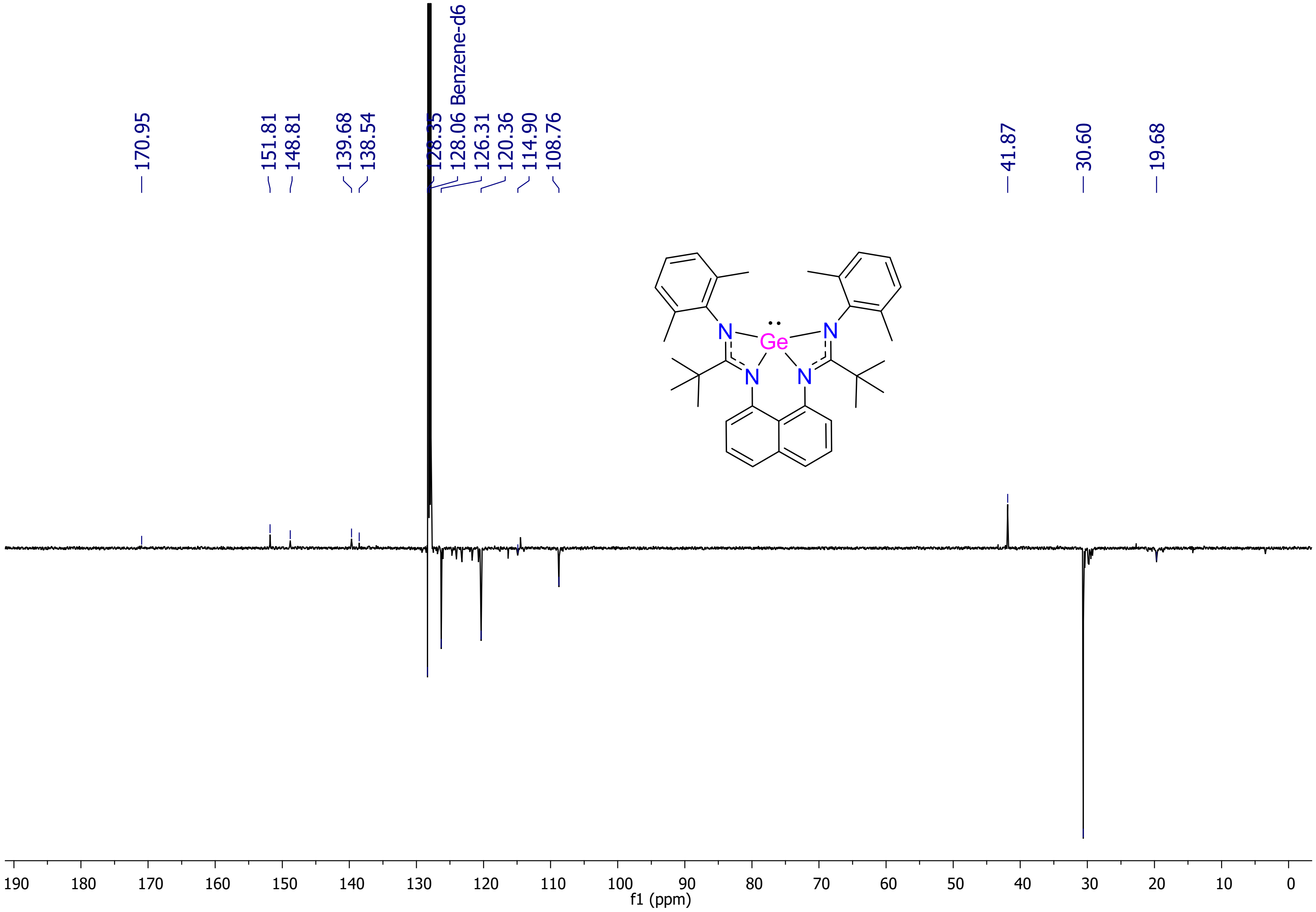

$^{13}C\{^{1}H\}$ NMR spectrum of compound $\mathbf{L_2Ge}$ (C_6D_6, 125 MHz)

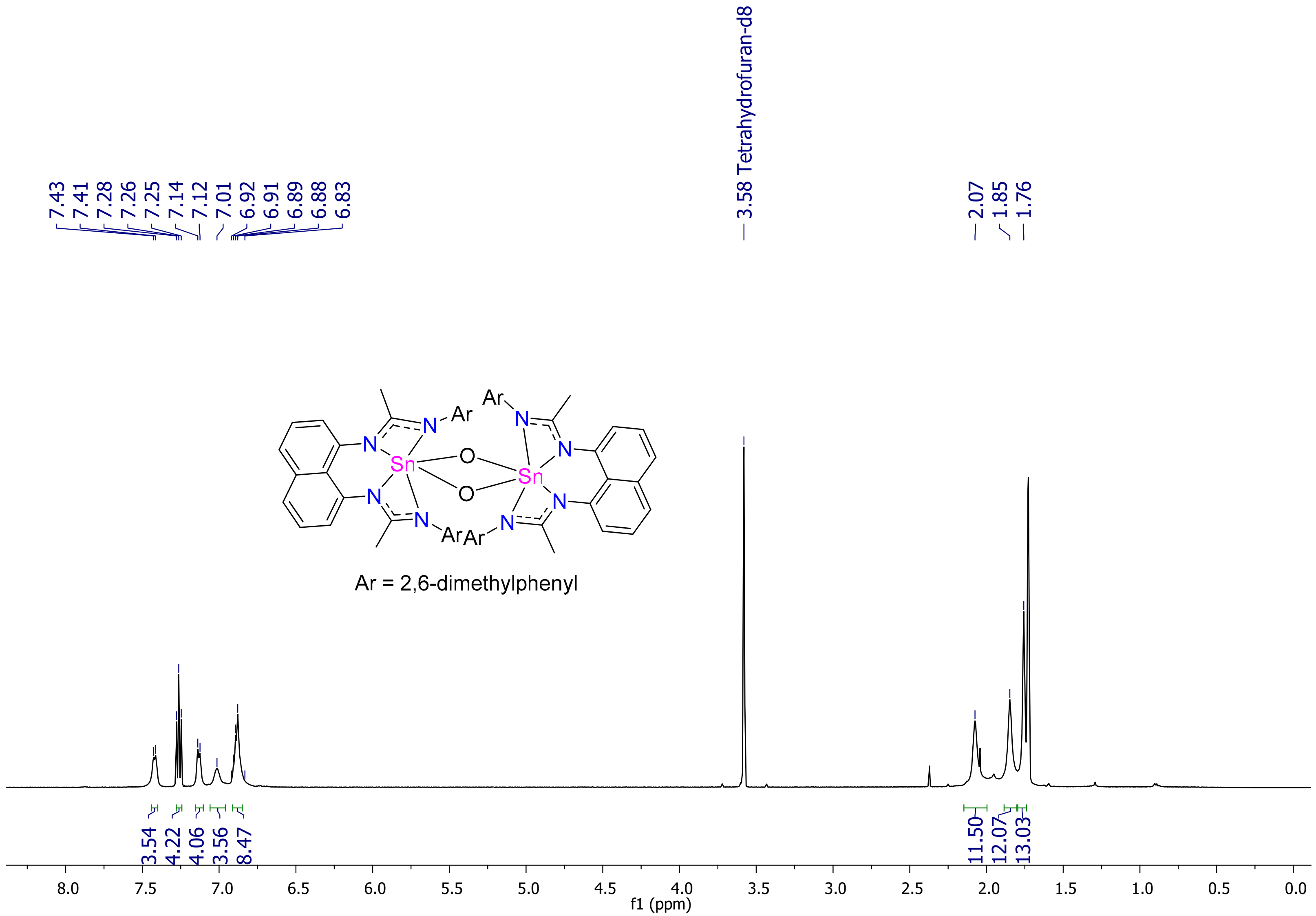

^{1}H NMR spectrum of compound **1a** (THF-d_8, 500 MHz)

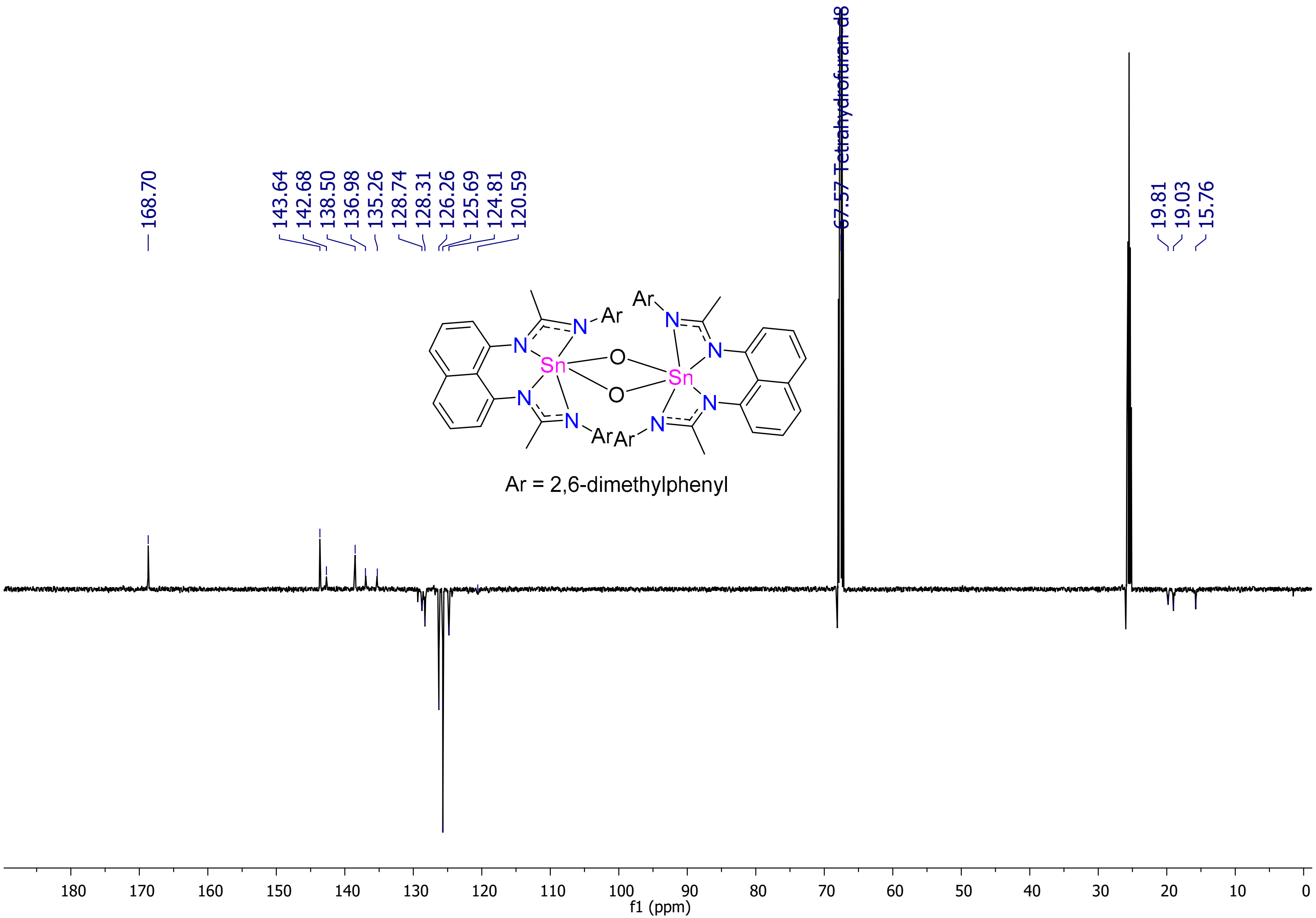

$^{13}C\{^{1}H\}$ NMR spectrum of compound **1a** (THF-d_8, 125 MHz)

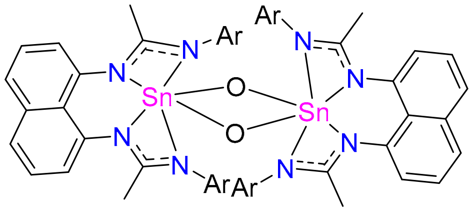

Ar = 2,6-dimethylphenyl

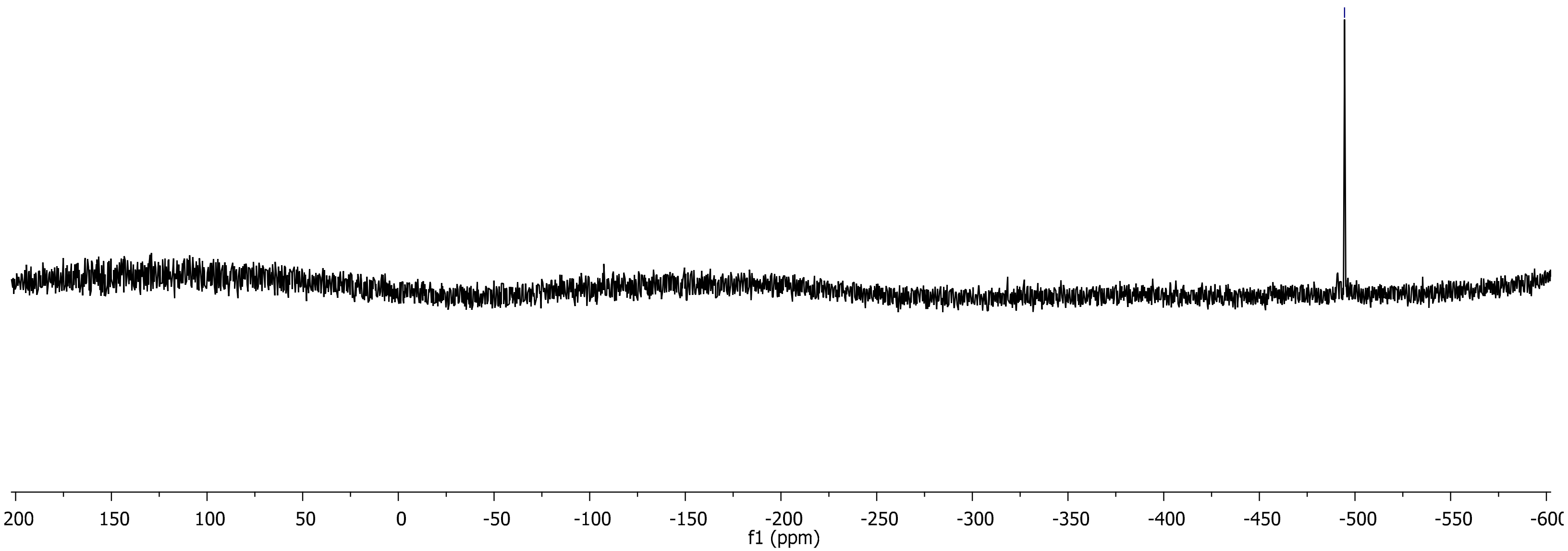

$^{119}Sn\{^{1}H\}$ NMR spectrum of compound **1a** (THF-d_8, 186 MHz)

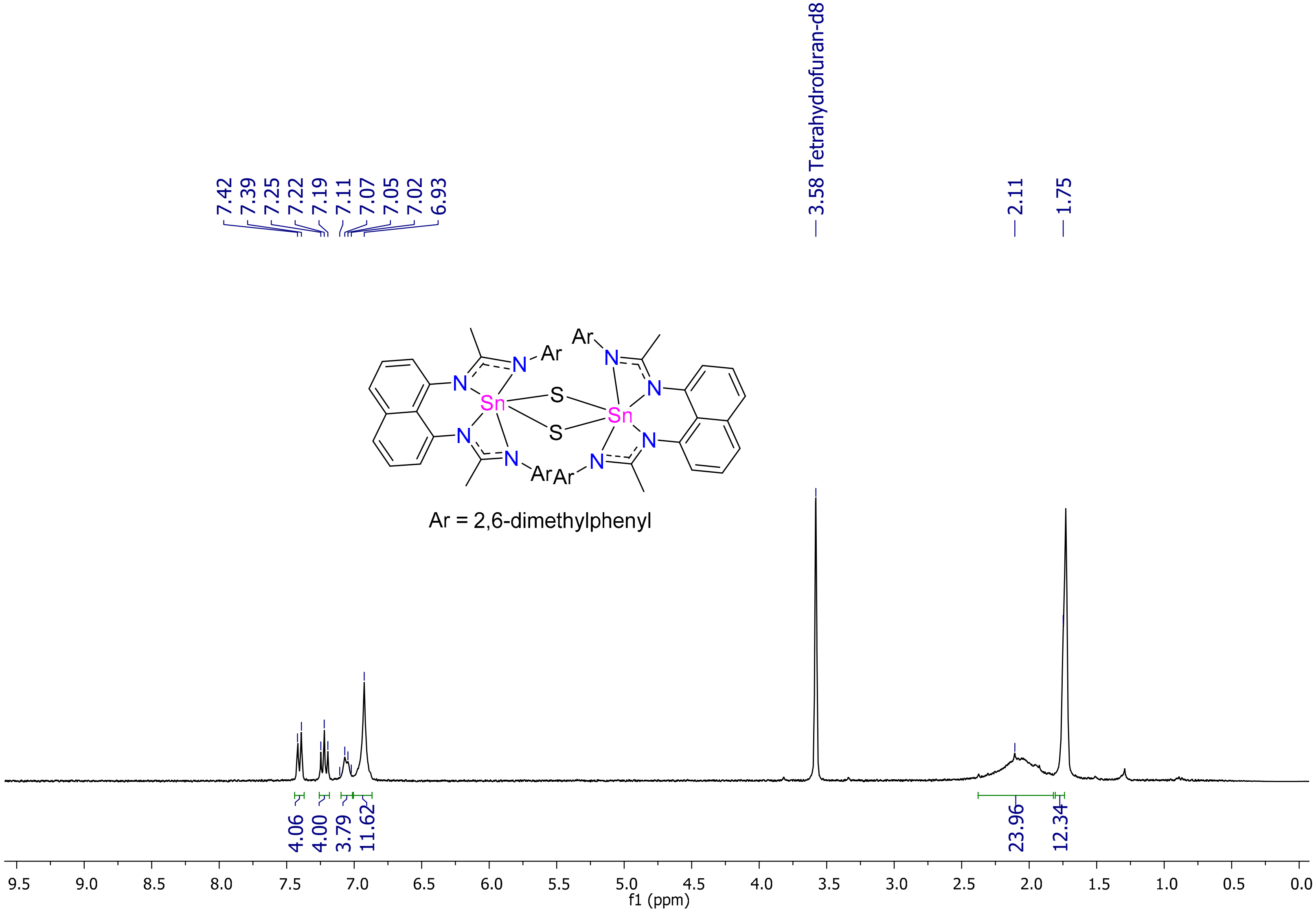

^{1}H NMR spectrum of compound **1b** (THF-d_8, 300 MHz)

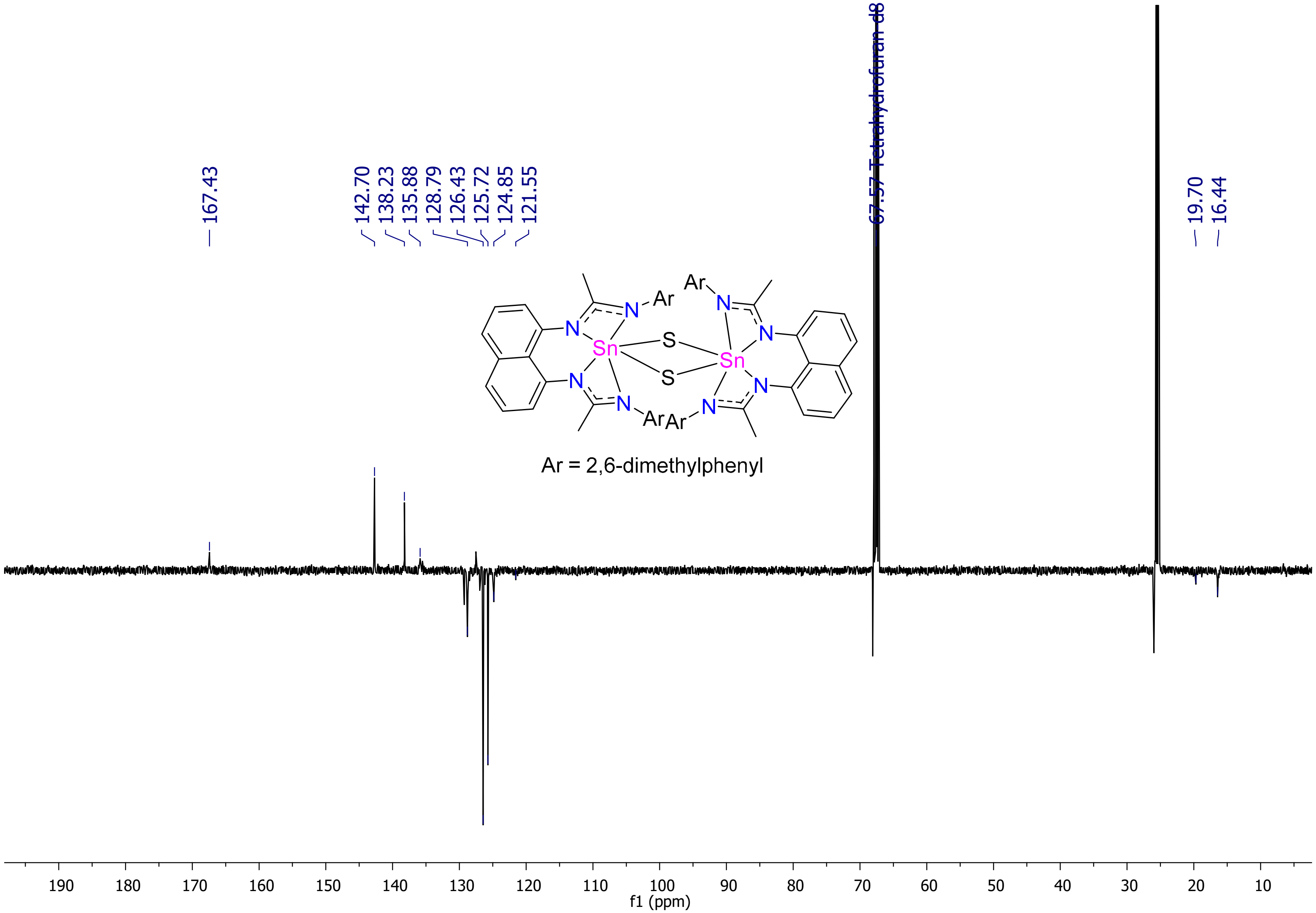

$^{13}C\{^{1}H\}$ NMR spectrum of compound **1b** (THF-d_8, 125 MHz)

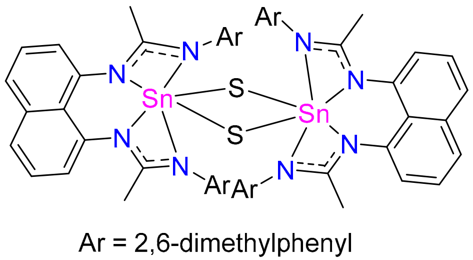

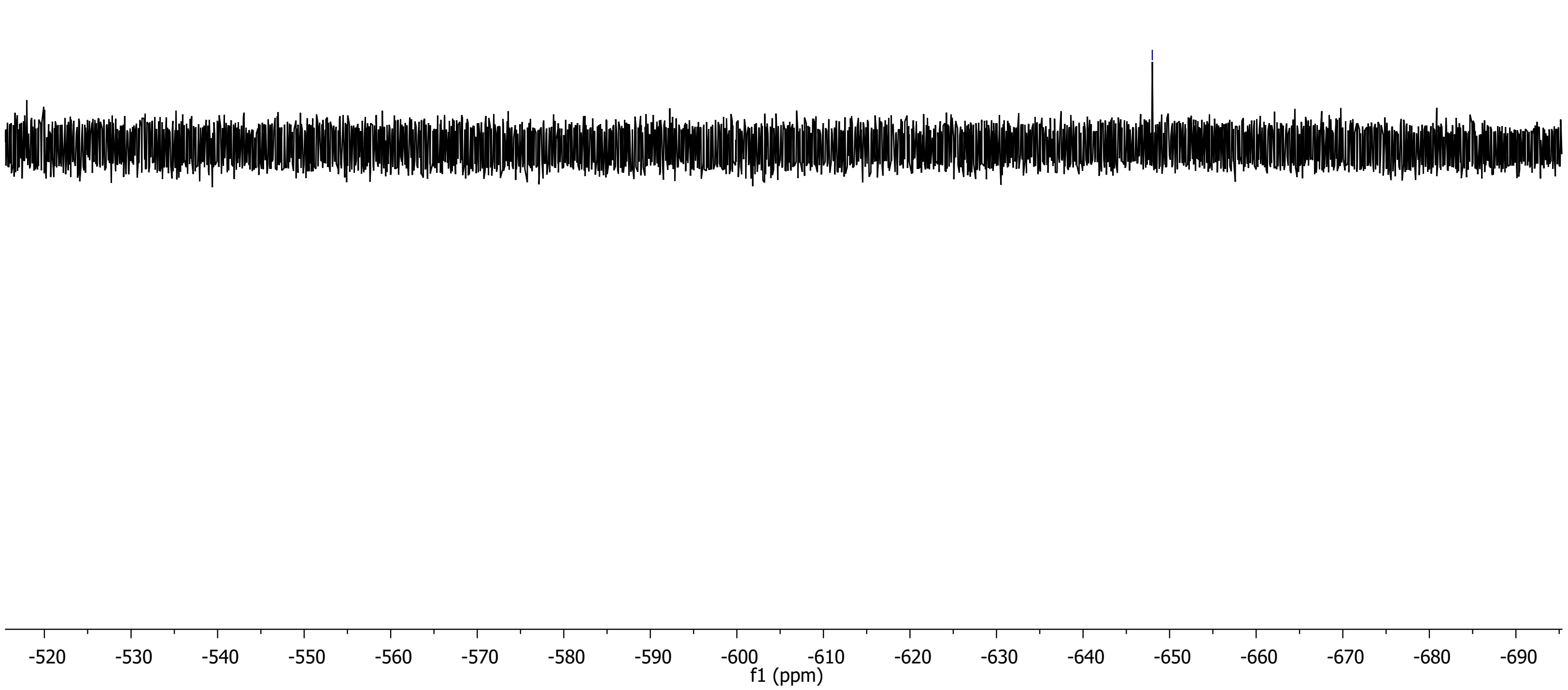

$^{119}Sn\{^{1}H\}$ NMR spectrum of compound **1b** (THF-d_8, 186 MHz)

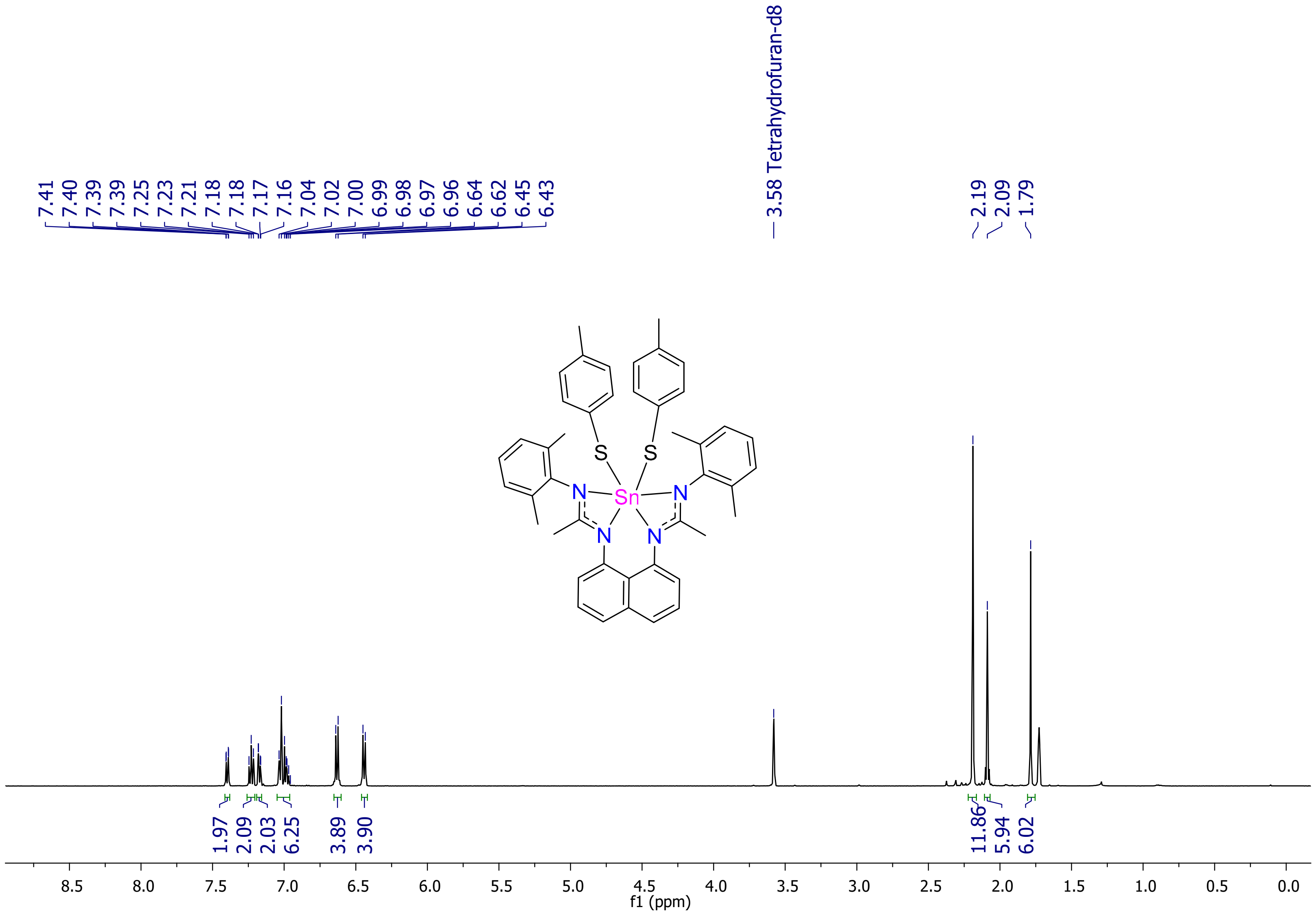

^{1}H NMR spectrum of compound **2a** (THF-d_8, 500 MHz)

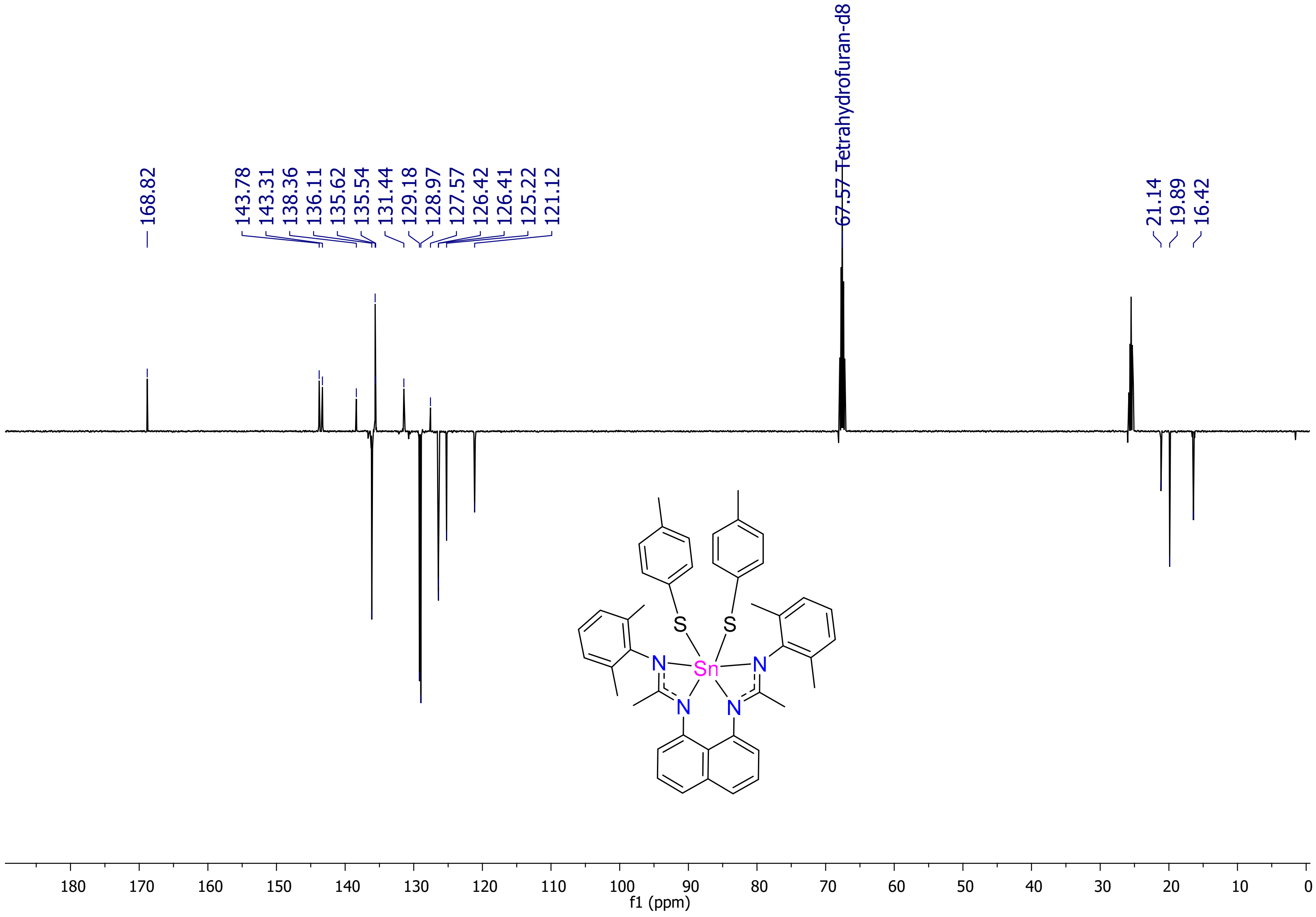

$^{13}C\{^{1}H\}$ NMR spectrum of compound **2a** (THF-d_8, 125 MHz)

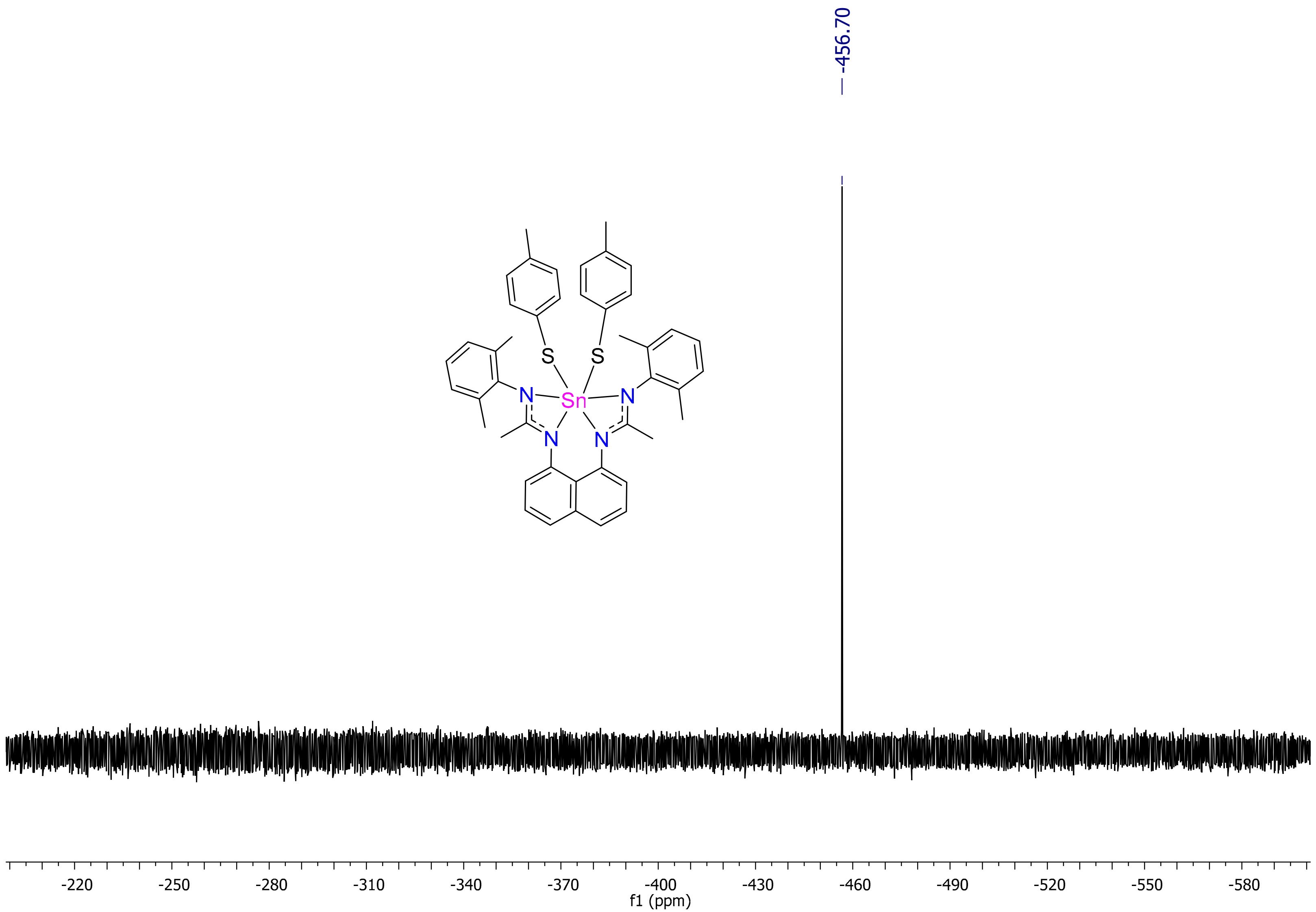

$^{119}Sn\{^1H\}$ NMR spectrum of compound **2a** (THF-d_8, 186 MHz)

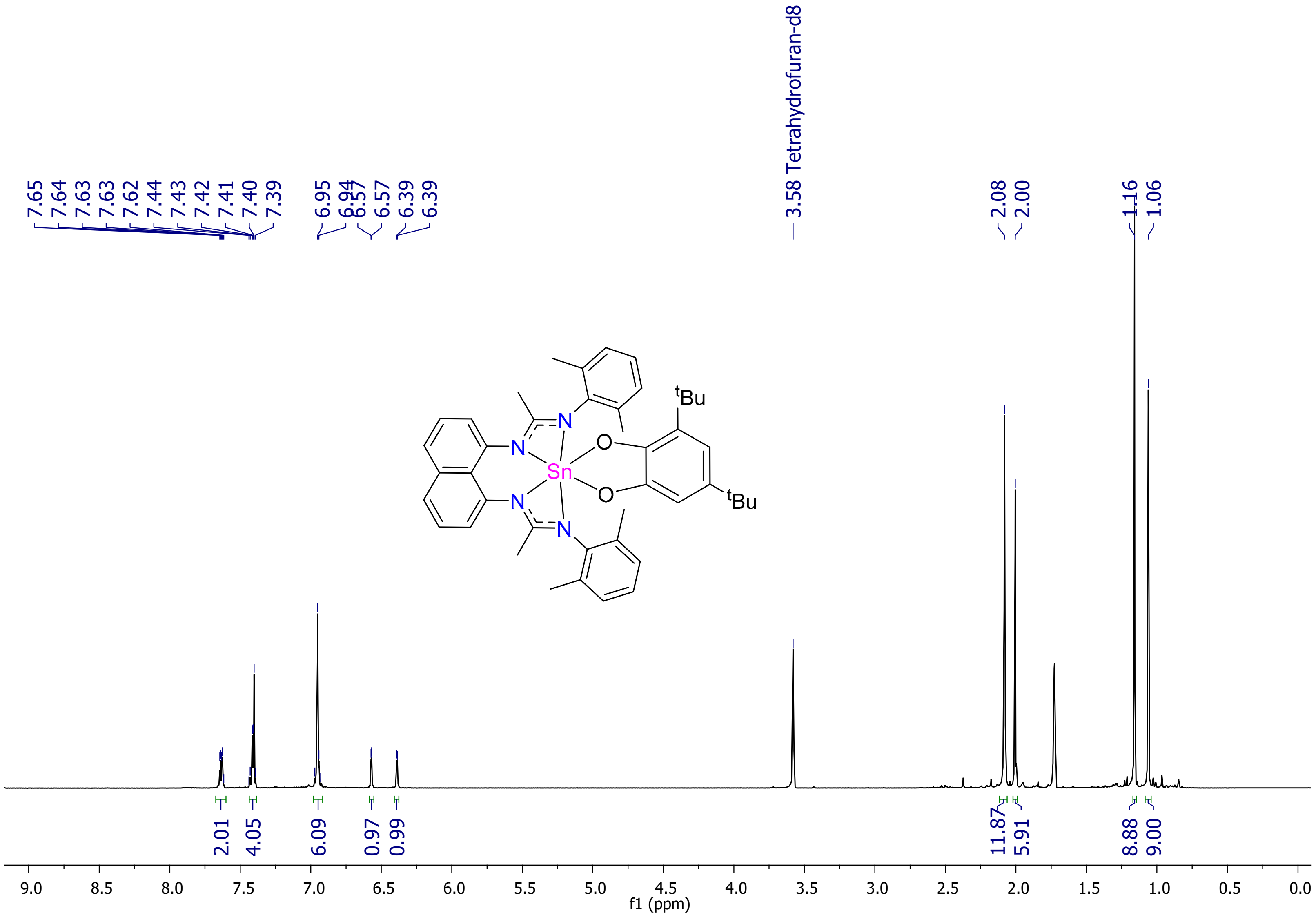

^{1}H NMR spectrum of compound **2b** (THF-d_8, 500 MHz)

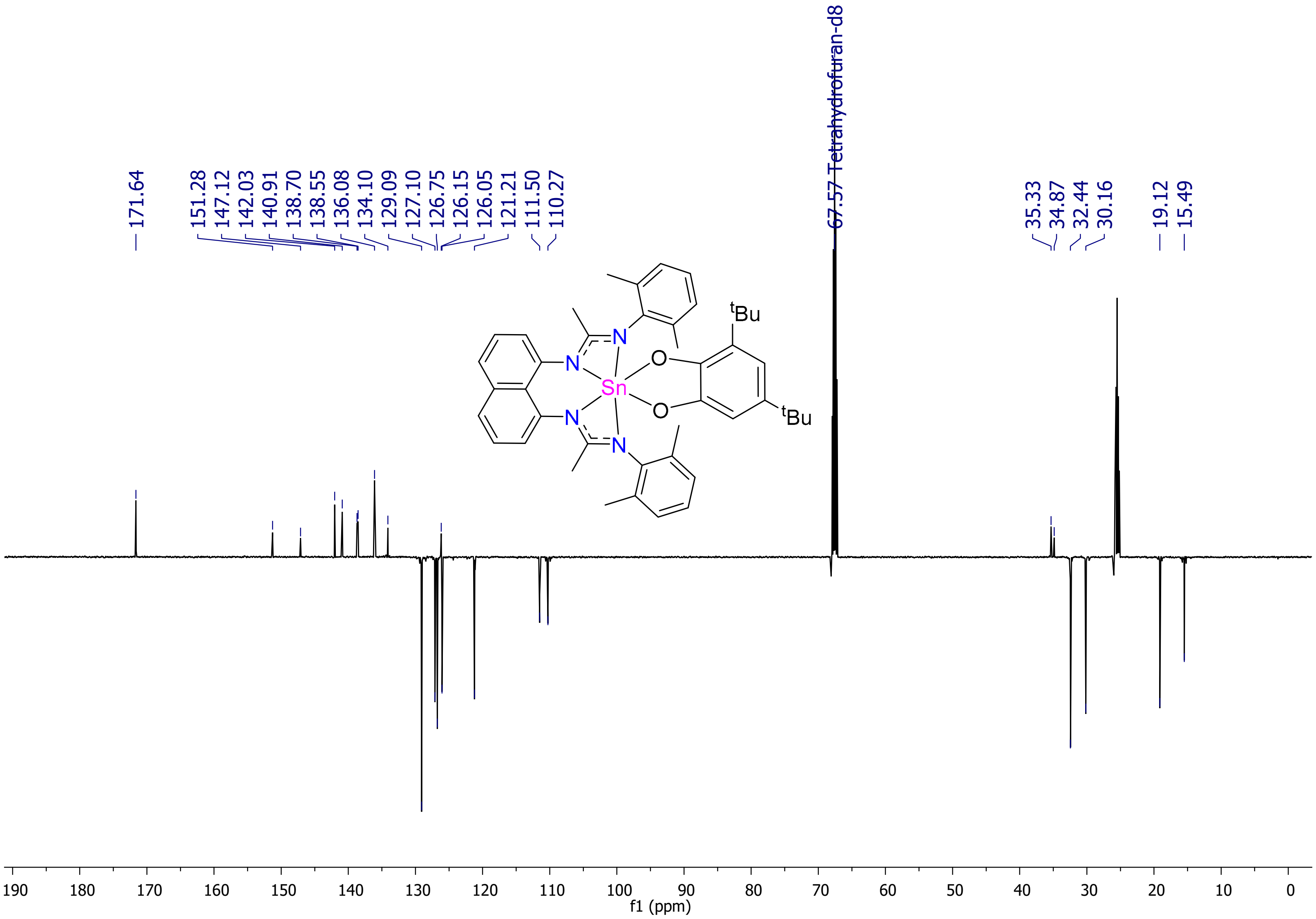

$^{13}C\{^{1}H\}$ NMR spectrum of compound **2b** (THF-d_8, 125 MHz)

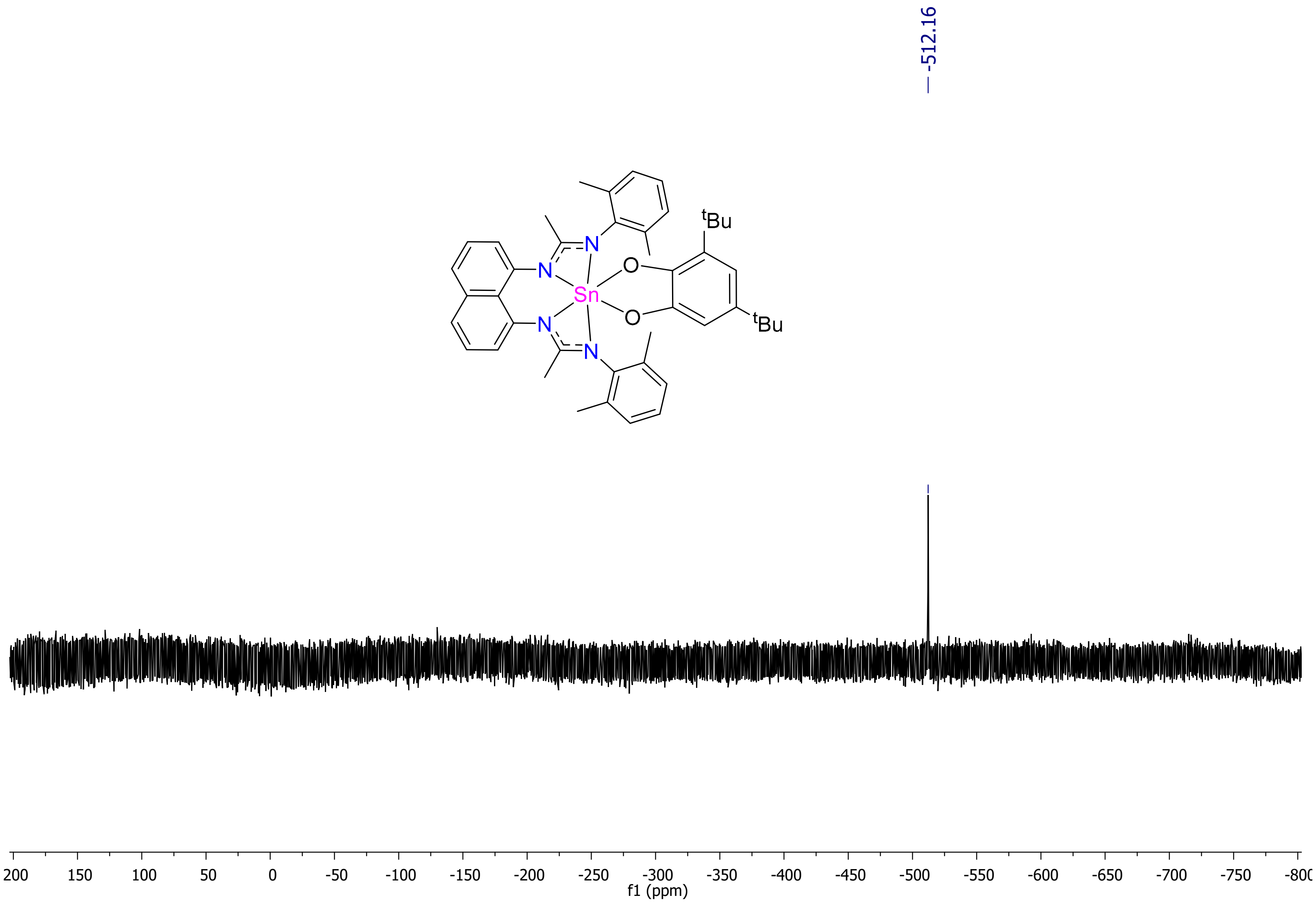

^{119}Sn{^{1}H} NMR spectrum of compound **2b** (THF-d_8, 186 MHz)

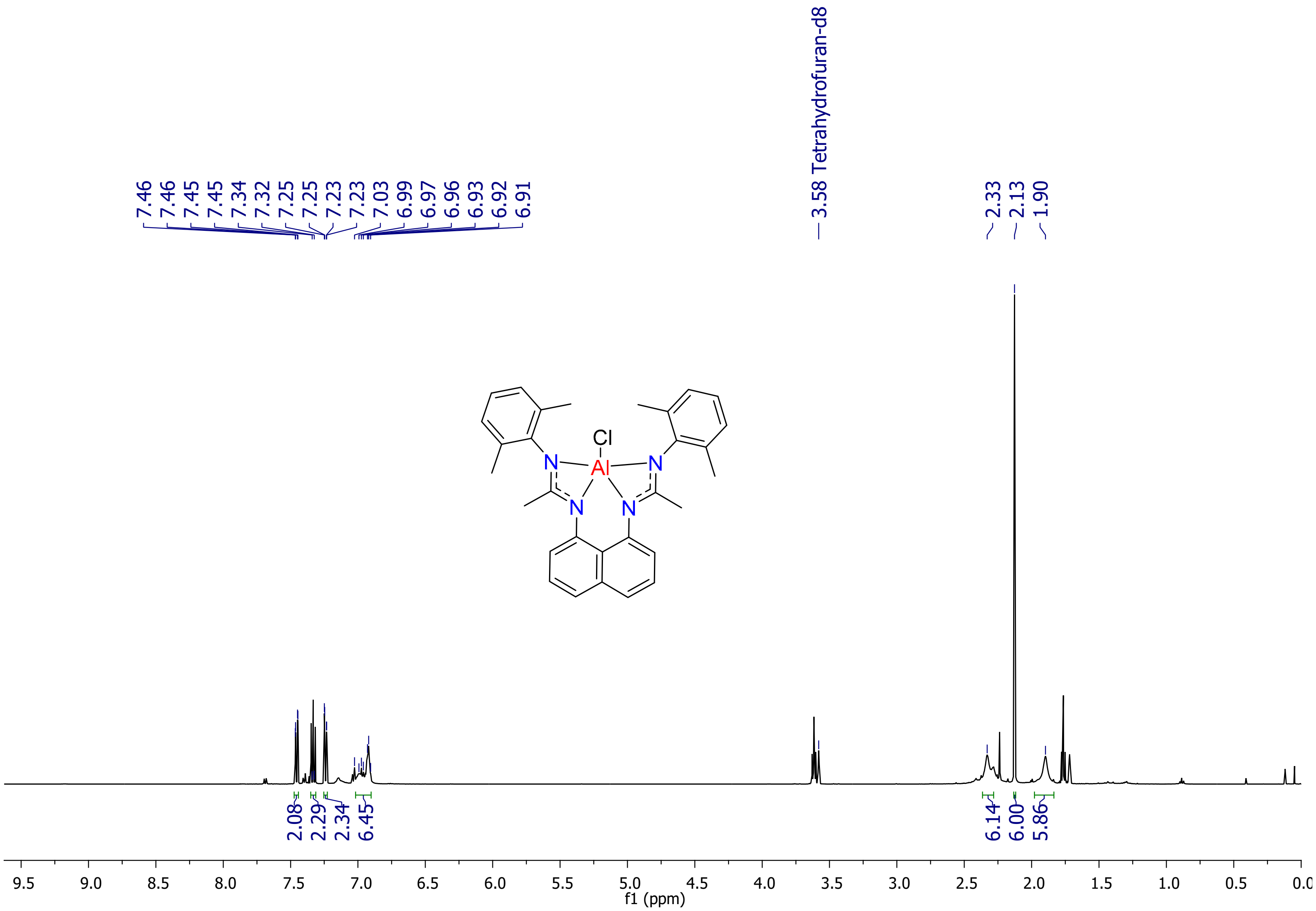

^{1}H NMR spectrum of compound **3a** (THF-d_8, 500 MHz)

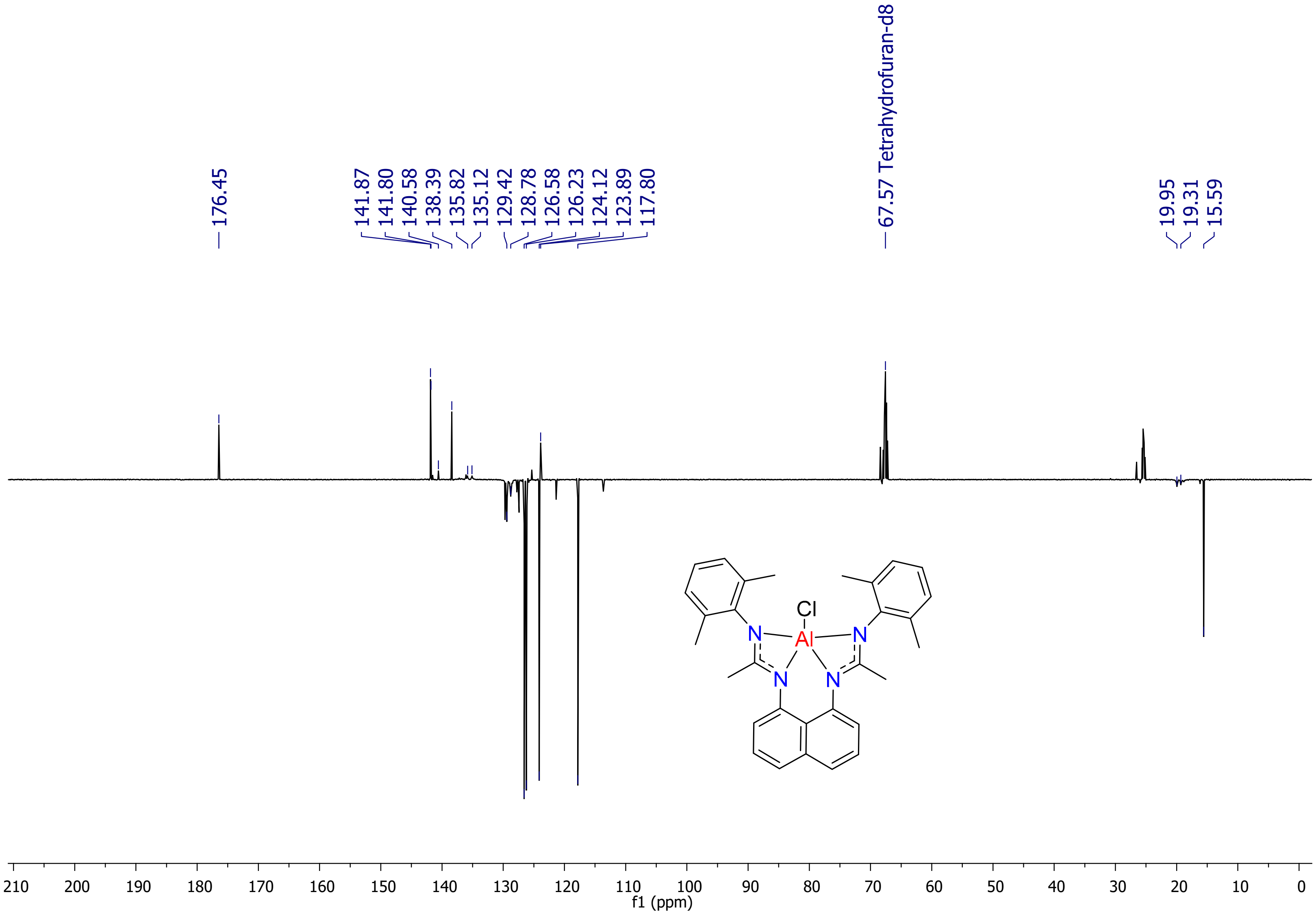

$^{13}C\{^{1}H\}$ NMR spectrum of compound **3a** (THF-d_8, 125 MHz)

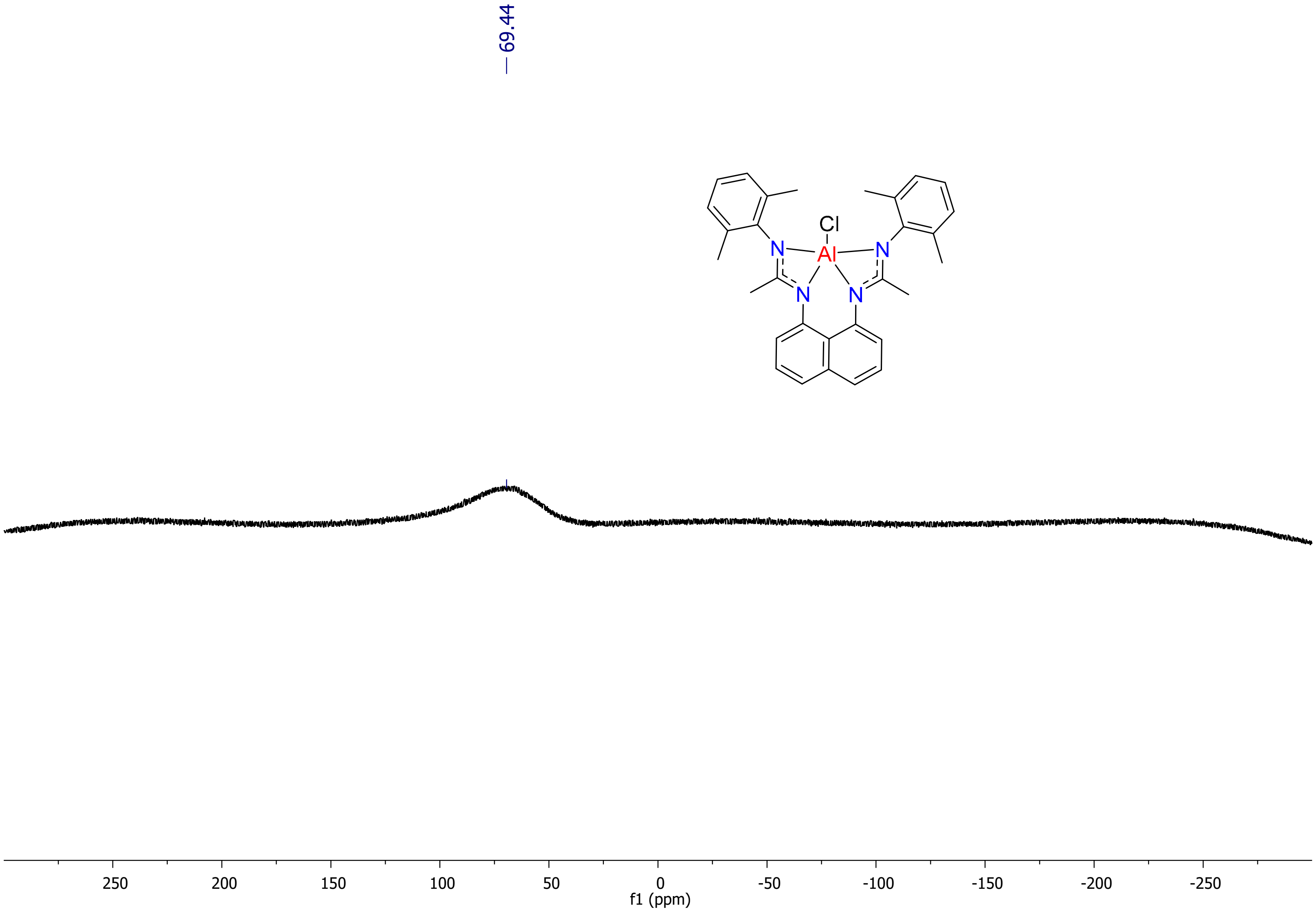

$^{27}Al\{^{1}H\}$ NMR spectrum of compound **3a** (THF-d_8, 130 MHz)

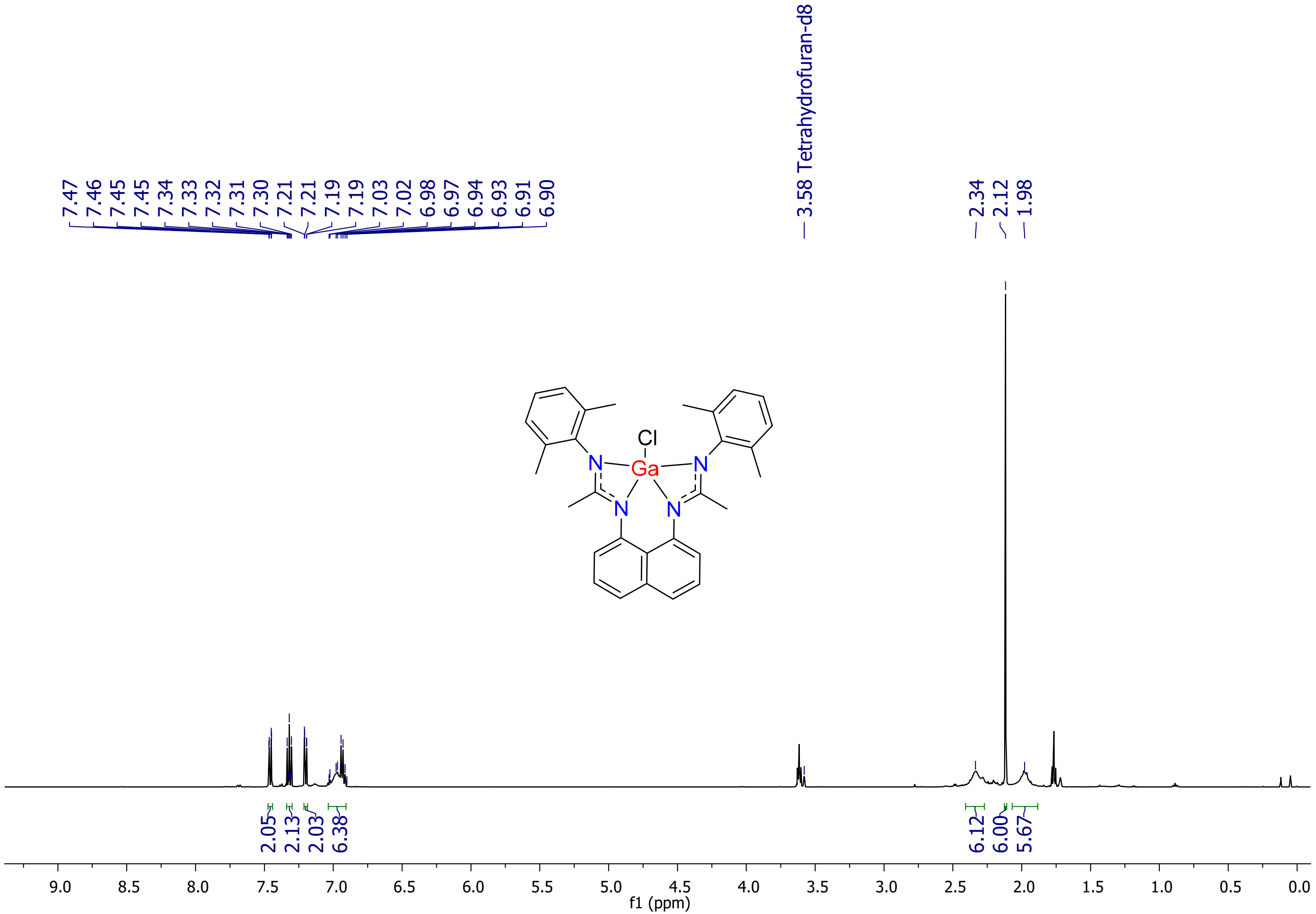

^{1}H NMR spectrum of compound **3b** (THF-d_8, 500 MHz)

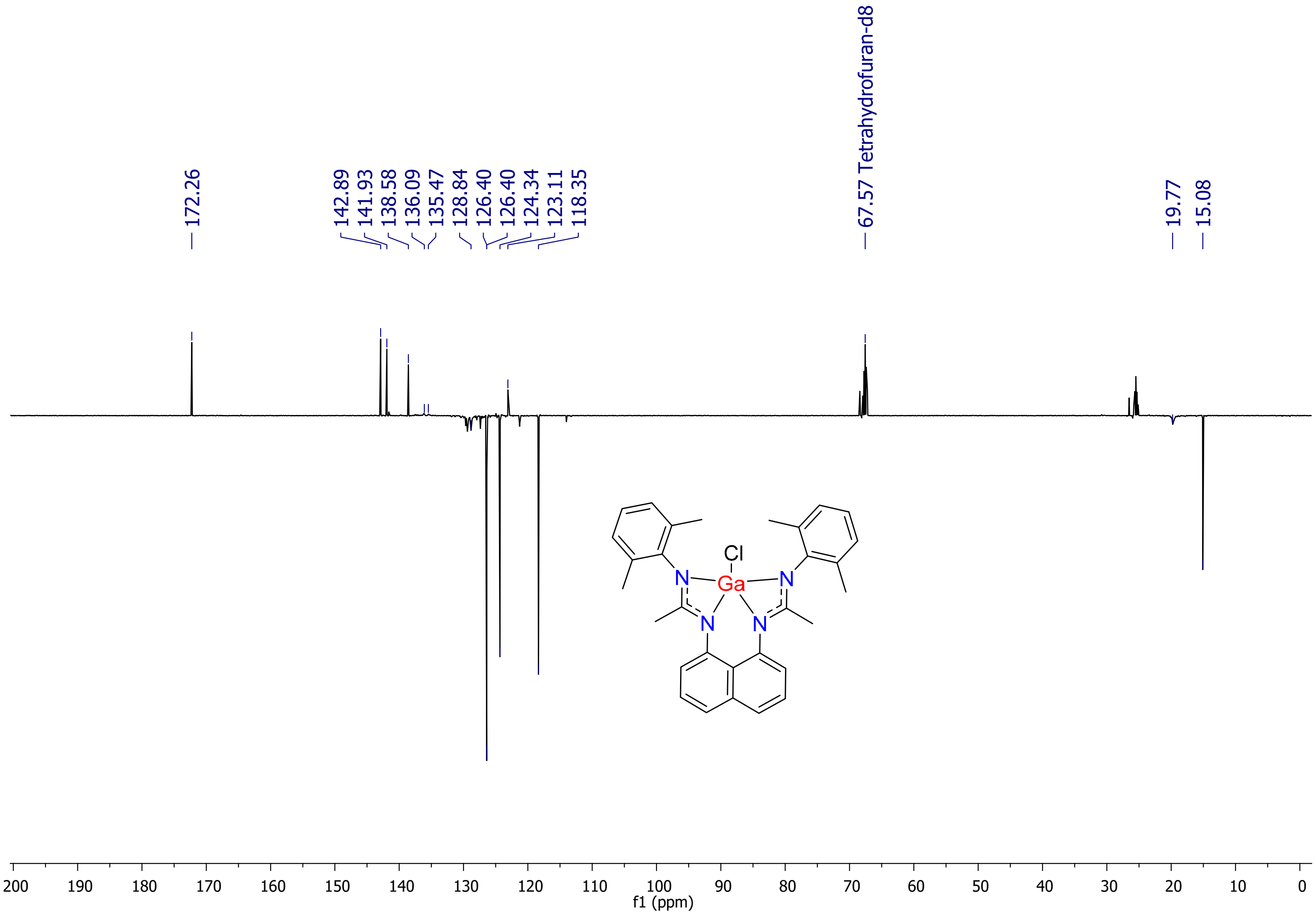

$^{13}C\{^{1}H\}$ NMR spectrum of compound **3b** (THF-d_8, 125 MHz)

Crystal data and structure refinement of compound L_3H_2

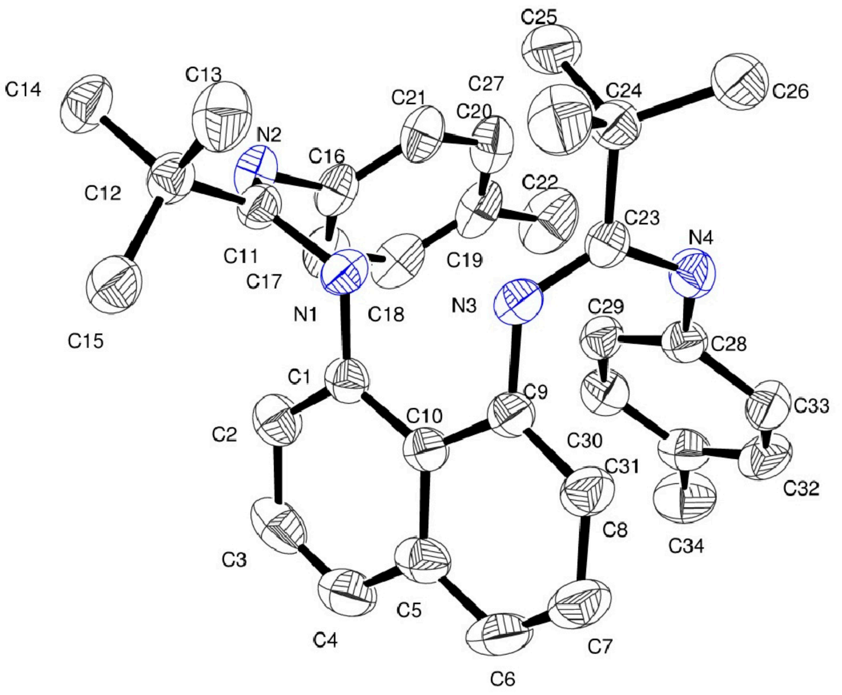

Figure S1. Asymmetric Unit

Table S1. Crystal data and structure refinement for R104.

Identification code	R104
Empirical formula	C34 H40 N4
Formula weight	504.70
Temperature	193(2) K
Wavelength	0.71073 A
Crystal system, space group	Orthorhombic, P 21 21 21
Unit cell dimensions	a = 10.4214(4) A alpha = 90 deg.<br>b = 16.1580(7) A beta = 90 deg.<br>c = 17.5675(8) A gamma = 90 deg.
Volume	2958.2(2) A^3
Z, Calculated density	4, 1.133 Mg/m^3
Absorption coefficient	0.067 mm^-1

F(000)	1088
Crystal size	0.180 x 0.160 x 0.100 mm
Theta range for data collection	2.639 to 25.376 deg.
Limiting indices	-12<=h<=12, -18<=k<=19, -21<=l<=21
Reflections collected / unique	45826 / 5433 [R(int) = 0.1088]
Completeness to theta = 25.242	99.8 %
Refinement method	Full-matrix least-squares on F^2
Data / restraints / parameters	5433 / 0 / 359
Goodness-of-fit on F^2	1.030
Final R indices [I>2sigma(I)]	R1 = 0.0510, wR2 = 0.1244
R indices (all data)	R1 = 0.0756, wR2 = 0.1404
Largest diff. peak and hole	0.762 and -0.156 e.A^-3

Table S2. Atomic coordinates (x 10^4) and equivalent isotropic displacement parameters (A^2 x 10^3) for R104.
U(eq) is defined as one third of the trace of the orthogonalized Uij tensor.

	x	y	z	U(eq)
C(1)	8472(3)	3199(2)	2197(2)	35(1)
C(2)	9235(4)	3678(3)	2654(2)	44(1)
C(3)	10477(4)	3409(3)	2863(3)	54(1)
C(4)	10944(4)	2678(3)	2617(3)	53(1)
C(5)	10202(4)	2163(3)	2141(2)	45(1)
C(6)	10697(4)	1404(3)	1870(3)	55(1)
C(7)	10001(4)	909(3)	1406(3)	55(1)
C(8)	8753(4)	1127(2)	1200(2)	43(1)
C(9)	8213(3)	1856(2)	1457(2)	35(1)
C(10)	8935(3)	2410(2)	1926(2)	34(1)
C(11)	6840(4)	4297(2)	2095(2)	36(1)
C(12)	6932(4)	4882(2)	1420(2)	39(1)
C(13)	6144(5)	4531(3)	759(2)	59(1)
C(14)	6417(4)	5738(3)	1612(3)	52(1)
C(15)	8333(4)	4955(3)	1176(3)	52(1)
C(16)	6200(4)	3962(2)	3331(2)	40(1)
C(17)	6953(4)	3963(3)	3975(2)	47(1)
C(18)	6691(5)	3425(3)	4572(2)	54(1)
C(19)	5651(5)	2901(3)	4557(2)	53(1)
C(20)	4872(5)	2921(3)	3916(3)	53(1)
C(21)	5146(5)	3436(2)	3314(2)	46(1)
C(22)	5392(6)	2320(3)	5212(3)	80(2)
C(23)	5993(3)	1598(2)	1292(2)	32(1)
C(24)	4758(4)	1812(2)	863(2)	38(1)
C(25)	4060(4)	2476(3)	1336(3)	56(1)
C(26)	3865(4)	1068(3)	774(2)	48(1)
C(27)	5077(5)	2145(3)	71(3)	59(1)
C(28)	6650(3)	812(2)	2451(2)	34(1)
C(29)	6898(3)	1451(2)	2950(2)	36(1)
C(30)	7495(4)	1292(3)	3637(2)	43(1)
C(31)	7850(4)	501(3)	3853(2)	43(1)
C(32)	7610(4)	-132(3)	3347(3)	49(1)
C(33)	7035(4)	16(2)	2651(2)	42(1)
C(34)	8437(5)	340(3)	4628(3)	65(1)
N(1)	7260(3)	3472(2)	1969(2)	36(1)
N(2)	6394(3)	4539(2)	2732(2)	40(1)
N(3)	6974(3)	2068(2)	1191(2)	34(1)
N(4)	5926(3)	932(2)	1777(2)	39(1)

Table S3. Bond lengths [A] and angles [deg] for R104.

C(1)-C(2)	1.371(5)
C(1)-N(1)	1.397(5)
C(1)-C(10)	1.443(5)
C(2)-C(3)	1.414(6)
C(2)-H(2)	0.9500
C(3)-C(4)	1.349(7)
C(3)-H(3)	0.9500
C(4)-C(5)	1.409(6)
C(4)-H(4)	0.9500
C(5)-C(6)	1.414(6)
C(5)-C(10)	1.431(5)
C(6)-C(7)	1.352(7)
C(6)-H(6)	0.9500
C(7)-C(8)	1.395(6)
C(7)-H(7)	0.9500
C(8)-C(9)	1.381(5)
C(8)-H(8)	0.9500
C(9)-N(3)	1.416(5)
C(9)-C(10)	1.430(5)
C(11)-N(2)	1.272(5)
C(11)-N(1)	1.421(5)
C(11)-C(12)	1.519(5)
C(12)-C(14)	1.521(5)
C(12)-C(15)	1.526(6)
C(12)-C(13)	1.531(6)
C(13)-H(13A)	0.9800
C(13)-H(13B)	0.9800
C(13)-H(13C)	0.9800
C(14)-H(14A)	0.9800
C(14)-H(14B)	0.9800
C(14)-H(14C)	0.9800
C(15)-H(15A)	0.9800
C(15)-H(15B)	0.9800
C(15)-H(15C)	0.9800
C(16)-C(17)	1.378(6)
C(16)-C(21)	1.389(6)
C(16)-N(2)	1.421(5)
C(17)-C(18)	1.389(6)
C(17)-H(17)	0.9500
C(18)-C(19)	1.376(7)
C(18)-H(18)	0.9500
C(19)-C(20)	1.389(7)
C(19)-C(22)	1.509(6)
C(20)-C(21)	1.376(6)
C(20)-H(20)	0.9500
C(21)-H(21)	0.9500
C(22)-H(22A)	0.9800
C(22)-H(22B)	0.9800
C(22)-H(22C)	0.9800
C(23)-N(3)	1.286(5)
C(23)-N(4)	1.373(5)

C(23)-C(24)	1.531(5)
C(24)-C(27)	1.528(6)
C(24)-C(26)	1.529(5)
C(24)-C(25)	1.540(6)
C(25)-H(25A)	0.9800
C(25)-H(25B)	0.9800
C(25)-H(25C)	0.9800
C(26)-H(26A)	0.9800
C(26)-H(26B)	0.9800
C(26)-H(26C)	0.9800
C(27)-H(27A)	0.9800
C(27)-H(27B)	0.9800
C(27)-H(27C)	0.9800
C(28)-C(29)	1.380(5)
C(28)-C(33)	1.392(5)
C(28)-N(4)	1.419(5)
C(29)-C(30)	1.382(5)
C(29)-H(29)	0.9500
C(30)-C(31)	1.383(6)
C(30)-H(30)	0.9500
C(31)-C(32)	1.378(6)
C(31)-C(34)	1.515(6)
C(32)-C(33)	1.383(6)
C(32)-H(32)	0.9500
C(33)-H(33)	0.9500
C(34)-H(34A)	0.9800
C(34)-H(34B)	0.9800
C(34)-H(34C)	0.9800
N(1)-H(1)	0.89(5)
N(4)-H(4A)	0.89(5)

C(2)-C(1)-N(1)	120.9(4)
C(2)-C(1)-C(10)	119.9(3)
N(1)-C(1)-C(10)	119.1(3)
C(1)-C(2)-C(3)	120.6(4)
C(1)-C(2)-H(2)	119.7
C(3)-C(2)-H(2)	119.7
C(4)-C(3)-C(2)	121.1(4)
C(4)-C(3)-H(3)	119.5
C(2)-C(3)-H(3)	119.5
C(3)-C(4)-C(5)	120.6(4)
C(3)-C(4)-H(4)	119.7
C(5)-C(4)-H(4)	119.7
C(4)-C(5)-C(6)	120.8(4)
C(4)-C(5)-C(10)	119.9(4)
C(6)-C(5)-C(10)	119.3(4)
C(7)-C(6)-C(5)	121.4(4)
C(7)-C(6)-H(6)	119.3
C(5)-C(6)-H(6)	119.3
C(6)-C(7)-C(8)	120.5(4)
C(6)-C(7)-H(7)	119.8
C(8)-C(7)-H(7)	119.8
C(9)-C(8)-C(7)	120.7(4)
C(9)-C(8)-H(8)	119.7

C(7)-C(8)-H(8)	119.7
C(8)-C(9)-N(3)	118.0(3)
C(8)-C(9)-C(10)	120.5(3)
N(3)-C(9)-C(10)	121.2(3)
C(9)-C(10)-C(5)	117.6(3)
C(9)-C(10)-C(1)	124.5(3)
C(5)-C(10)-C(1)	117.8(4)
N(2)-C(11)-N(1)	122.5(3)
N(2)-C(11)-C(12)	121.2(3)
N(1)-C(11)-C(12)	116.2(3)
C(11)-C(12)-C(14)	111.7(3)
C(11)-C(12)-C(15)	109.1(3)
C(14)-C(12)-C(15)	109.2(3)
C(11)-C(12)-C(13)	109.2(3)
C(14)-C(12)-C(13)	108.4(4)
C(15)-C(12)-C(13)	109.2(4)
C(12)-C(13)-H(13A)	109.5
C(12)-C(13)-H(13B)	109.5
H(13A)-C(13)-H(13B)	109.5
C(12)-C(13)-H(13C)	109.5
H(13A)-C(13)-H(13C)	109.5
H(13B)-C(13)-H(13C)	109.5
C(12)-C(14)-H(14A)	109.5
C(12)-C(14)-H(14B)	109.5
H(14A)-C(14)-H(14B)	109.5
C(12)-C(14)-H(14C)	109.5
H(14A)-C(14)-H(14C)	109.5
H(14B)-C(14)-H(14C)	109.5
C(12)-C(15)-H(15A)	109.5
C(12)-C(15)-H(15B)	109.5
H(15A)-C(15)-H(15B)	109.5
C(12)-C(15)-H(15C)	109.5
H(15A)-C(15)-H(15C)	109.5
H(15B)-C(15)-H(15C)	109.5
C(17)-C(16)-C(21)	118.0(4)
C(17)-C(16)-N(2)	121.8(4)
C(21)-C(16)-N(2)	119.9(4)
C(16)-C(17)-C(18)	120.5(4)
C(16)-C(17)-H(17)	119.7
C(18)-C(17)-H(17)	119.7
C(19)-C(18)-C(17)	121.7(4)
C(19)-C(18)-H(18)	119.1
C(17)-C(18)-H(18)	119.1
C(18)-C(19)-C(20)	117.5(4)
C(18)-C(19)-C(22)	120.6(5)
C(20)-C(19)-C(22)	121.9(5)
C(21)-C(20)-C(19)	121.0(4)
C(21)-C(20)-H(20)	119.5
C(19)-C(20)-H(20)	119.5
C(20)-C(21)-C(16)	121.2(4)
C(20)-C(21)-H(21)	119.4
C(16)-C(21)-H(21)	119.4
C(19)-C(22)-H(22A)	109.5
C(19)-C(22)-H(22B)	109.5

H(22A)-C(22)-H(22B)	109.5
C(19)-C(22)-H(22C)	109.5
H(22A)-C(22)-H(22C)	109.5
H(22B)-C(22)-H(22C)	109.5
N(3)-C(23)-N(4)	126.1(3)
N(3)-C(23)-C(24)	117.8(3)
N(4)-C(23)-C(24)	116.1(3)
C(27)-C(24)-C(26)	108.4(3)
C(27)-C(24)-C(23)	110.2(3)
C(26)-C(24)-C(23)	112.6(3)
C(27)-C(24)-C(25)	110.4(4)
C(26)-C(24)-C(25)	108.4(3)
C(23)-C(24)-C(25)	106.8(3)
C(24)-C(25)-H(25A)	109.5
C(24)-C(25)-H(25B)	109.5
H(25A)-C(25)-H(25B)	109.5
C(24)-C(25)-H(25C)	109.5
H(25A)-C(25)-H(25C)	109.5
H(25B)-C(25)-H(25C)	109.5
C(24)-C(26)-H(26A)	109.5
C(24)-C(26)-H(26B)	109.5
H(26A)-C(26)-H(26B)	109.5
C(24)-C(26)-H(26C)	109.5
H(26A)-C(26)-H(26C)	109.5
H(26B)-C(26)-H(26C)	109.5
C(24)-C(27)-H(27A)	109.5
C(24)-C(27)-H(27B)	109.5
H(27A)-C(27)-H(27B)	109.5
C(24)-C(27)-H(27C)	109.5
H(27A)-C(27)-H(27C)	109.5
H(27B)-C(27)-H(27C)	109.5
C(29)-C(28)-C(33)	118.6(3)
C(29)-C(28)-N(4)	121.8(3)
C(33)-C(28)-N(4)	119.4(3)
C(28)-C(29)-C(30)	120.0(3)
C(28)-C(29)-H(29)	120.0
C(30)-C(29)-H(29)	120.0
C(29)-C(30)-C(31)	122.2(4)
C(29)-C(30)-H(30)	118.9
C(31)-C(30)-H(30)	118.9
C(32)-C(31)-C(30)	117.4(4)
C(32)-C(31)-C(34)	121.7(4)
C(30)-C(31)-C(34)	120.9(4)
C(31)-C(32)-C(33)	121.5(4)
C(31)-C(32)-H(32)	119.3
C(33)-C(32)-H(32)	119.3
C(32)-C(33)-C(28)	120.4(4)
C(32)-C(33)-H(33)	119.8
C(28)-C(33)-H(33)	119.8
C(31)-C(34)-H(34A)	109.5
C(31)-C(34)-H(34B)	109.5
H(34A)-C(34)-H(34B)	109.5
C(31)-C(34)-H(34C)	109.5
H(34A)-C(34)-H(34C)	109.5

H(34B)-C(34)-H(34C)	109.5
C(1)-N(1)-C(11)	122.0(3)
C(1)-N(1)-H(1)	111(3)
C(11)-N(1)-H(1)	119(3)
C(11)-N(2)-C(16)	120.1(3)
C(23)-N(3)-C(9)	122.5(3)
C(23)-N(4)-C(28)	126.8(3)
C(23)-N(4)-H(4A)	115(3)
C(28)-N(4)-H(4A)	115(3)

Symmetry transformations used to generate equivalent atoms:

Table S4. Anisotropic displacement parameters (A^2 x 10^3) for R104. The anisotropic displacement factor exponent takes the form: -2 pi^2 [h^2 a*^2 U11 + ... + 2 h k a* b* U12]

	U11	U22	U33	U23	U13	U12
C(1)	34(2)	37(2)	32(2)	7(2)	3(2)	-3(2)
C(2)	44(2)	47(2)	39(2)	3(2)	0(2)	-6(2)
C(3)	42(2)	73(3)	46(2)	9(2)	-7(2)	-18(2)
C(4)	36(2)	67(3)	56(3)	16(2)	0(2)	1(2)
C(5)	35(2)	56(3)	42(2)	16(2)	6(2)	2(2)
C(6)	39(2)	63(3)	62(3)	19(2)	9(2)	21(2)
C(7)	49(3)	50(3)	64(3)	4(2)	19(2)	17(2)
C(8)	41(2)	40(2)	49(2)	3(2)	12(2)	6(2)
C(9)	33(2)	36(2)	35(2)	6(2)	8(2)	2(2)
C(10)	32(2)	37(2)	32(2)	11(2)	8(2)	1(2)
C(11)	38(2)	30(2)	39(2)	-1(2)	-1(2)	2(2)
C(12)	44(2)	35(2)	38(2)	5(2)	7(2)	4(2)
C(13)	76(3)	56(3)	44(2)	14(2)	-3(2)	-4(2)
C(14)	61(3)	41(2)	55(3)	15(2)	18(2)	12(2)
C(15)	54(3)	48(2)	54(3)	12(2)	18(2)	7(2)
C(16)	60(3)	30(2)	31(2)	-5(2)	5(2)	5(2)
C(17)	59(3)	43(2)	38(2)	-4(2)	-1(2)	1(2)
C(18)	79(3)	50(2)	31(2)	-1(2)	2(2)	16(3)
C(19)	83(3)	38(2)	40(2)	4(2)	17(2)	11(2)
C(20)	68(3)	38(2)	53(3)	3(2)	10(2)	-4(2)
C(21)	67(3)	33(2)	39(2)	-3(2)	1(2)	2(2)
C(22)	110(5)	72(4)	58(3)	23(3)	19(3)	11(3)
C(23)	37(2)	29(2)	30(2)	-2(2)	1(2)	2(2)
C(24)	38(2)	37(2)	39(2)	-4(2)	-9(2)	1(2)
C(25)	44(2)	48(2)	74(3)	-16(2)	-13(2)	8(2)
C(26)	48(2)	48(2)	48(2)	-6(2)	-11(2)	-1(2)
C(27)	71(3)	59(3)	47(3)	15(2)	-18(2)	-5(2)
C(28)	31(2)	35(2)	35(2)	5(2)	2(2)	2(2)
C(29)	37(2)	32(2)	39(2)	7(2)	7(2)	2(2)
C(30)	42(2)	47(2)	38(2)	-1(2)	4(2)	0(2)
C(31)	36(2)	53(2)	41(2)	8(2)	1(2)	4(2)
C(32)	49(2)	44(2)	54(3)	16(2)	0(2)	12(2)
C(33)	46(2)	32(2)	49(2)	1(2)	2(2)	1(2)
C(34)	61(3)	79(3)	54(3)	11(3)	-8(2)	12(3)
N(1)	40(2)	32(2)	38(2)	-2(1)	-4(1)	6(1)
N(2)	56(2)	31(2)	34(2)	1(1)	2(2)	1(1)
N(3)	36(2)	32(2)	35(2)	2(1)	2(1)	6(1)
N(4)	40(2)	35(2)	41(2)	6(1)	-3(2)	-2(2)

Crystal data and structure refinement of compound L_1Sn

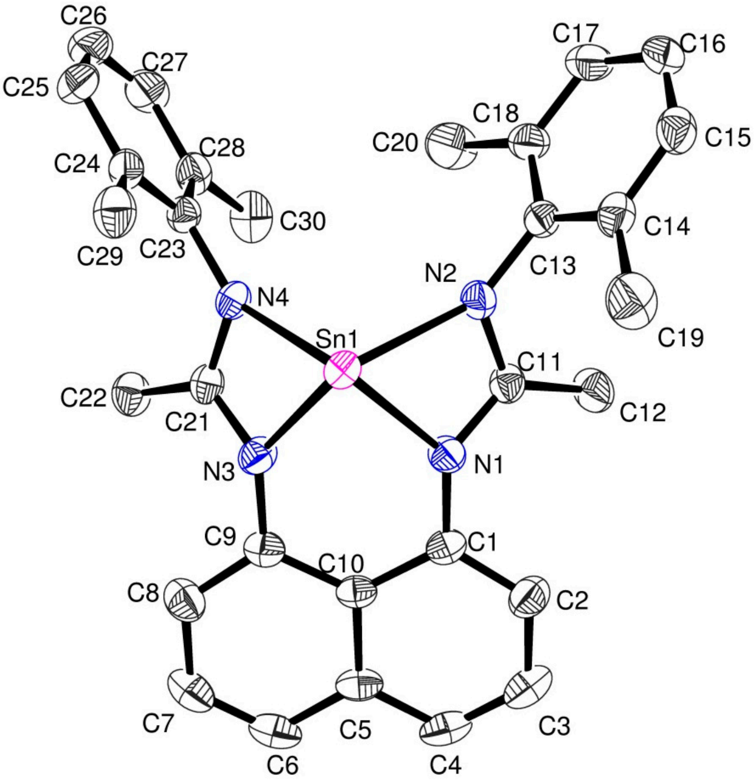

Figure S2. Asymmetric Unit

Table S5. Crystal data and structure refinement for R29.

Identification code	R29
Empirical formula	C30 H30 N4 Sn
Formula weight	565.29
Temperature	193(2) K
Wavelength	0.71073 A
Crystal system, space group	Monoclinic, C 2/c
Unit cell dimensions	a = 20.6812(9) A alpha = 90 deg.<br>b = 12.6563(5) A beta = 96.409(2) deg.<br>c = 20.1577(8) A gamma = 90 deg.
Volume	5243.3(4) A^3

Z, Calculated density	8, 1.432 Mg/m^3
Absorption coefficient	1.000 mm^-1
F(000)	2304
Crystal size	0.120 x 0.100 x 0.080 mm
Theta range for data collection	2.677 to 35.008 deg.
Limiting indices	-33<=h<=33, -20<=k<=19, -31<=l<=32
Reflections collected / unique	95894 / 11564 [R(int) = 0.0699]
Completeness to theta = 25.242	99.9 %
Refinement method	Full-matrix least-squares on F^2
Data / restraints / parameters	11564 / 0 / 322
Goodness-of-fit on F^2	1.029
Final R indices [I>2sigma(I)]	R1 = 0.0403, wR2 = 0.0747
R indices (all data)	R1 = 0.0784, wR2 = 0.0867
Largest diff. peak and hole	0.588 and -0.596 e.A^-3

Table S6. Atomic coordinates (x 10^4) and equivalent isotropic displacement parameters (A^2 x 10^3) for R29.
U(eq) is defined as one third of the trace of the orthogonalized Uij tensor.

	x	y	z	U(eq)
Sn(1)	7150(1)	5482(1)	5806(1)	25(1)
N(4)	6571(1)	4738(1)	4870(1)	26(1)
C(6)	5812(1)	9560(2)	5074(1)	40(1)
N(2)	6556(1)	4643(1)	6572(1)	29(1)
C(13)	6597(1)	3738(2)	6994(1)	28(1)
C(14)	7039(1)	3723(2)	7579(1)	33(1)
C(5)	5874(1)	8979(2)	5682(1)	32(1)
N(1)	6434(1)	6341(1)	6368(1)	29(1)
C(12)	5794(1)	5558(2)	7243(1)	41(1)
C(15)	7088(1)	2815(2)	7967(1)	38(1)
C(7)	5987(1)	9129(2)	4508(1)	43(1)
C(22)	5955(1)	5806(2)	3973(1)	36(1)
N(3)	6518(1)	6438(1)	5071(1)	30(1)
C(4)	5690(1)	9446(2)	6268(1)	40(1)
C(16)	6706(1)	1946(2)	7796(1)	43(1)
C(10)	6121(1)	7917(2)	5698(1)	26(1)
C(25)	7039(1)	2465(2)	3872(1)	42(1)
C(18)	6225(1)	2845(2)	6807(1)	34(1)
C(24)	7087(1)	3392(2)	4248(1)	31(1)
C(9)	6281(1)	7478(2)	5077(1)	29(1)
C(21)	6347(1)	5667(2)	4640(1)	27(1)
C(19)	7462(1)	4663(2)	7776(1)	52(1)
C(29)	7736(1)	3935(2)	4402(1)	40(1)
C(11)	6272(1)	5533(2)	6734(1)	29(1)
C(17)	6282(1)	1959(2)	7220(1)	43(1)
C(8)	6228(1)	8093(2)	4507(1)	38(1)
C(3)	5764(1)	8922(2)	6856(1)	46(1)
C(28)	5926(1)	3267(2)	4352(1)	33(1)
C(26)	6453(2)	1946(2)	3740(1)	48(1)
C(2)	6022(1)	7892(2)	6893(1)	38(1)
C(23)	6524(1)	3800(2)	4482(1)	26(1)
C(1)	6179(1)	7367(2)	6331(1)	28(1)
C(27)	5905(1)	2334(2)	3978(1)	43(1)
C(20)	5777(1)	2848(2)	6164(1)	52(1)
C(30)	5309(1)	3669(2)	4599(1)	43(1)

Table S7. Bond lengths [A] and angles [deg] for R29.

Sn(1)-N(3)	2.2232(17)
Sn(1)-N(1)	2.2435(17)
Sn(1)-N(4)	2.3181(16)
Sn(1)-N(2)	2.3327(17)
N(4)-C(21)	1.329(3)
N(4)-C(23)	1.420(2)
C(6)-C(7)	1.350(4)
C(6)-C(5)	1.422(3)
C(6)-H(6)	0.9500
N(2)-C(11)	1.328(3)
N(2)-C(13)	1.423(3)
C(13)-C(18)	1.395(3)
C(13)-C(14)	1.409(3)
C(14)-C(15)	1.387(3)
C(14)-C(19)	1.505(3)
C(5)-C(4)	1.410(3)
C(5)-C(10)	1.437(3)
N(1)-C(11)	1.326(3)
N(1)-C(1)	1.400(3)
C(12)-C(11)	1.503(3)
C(12)-H(12A)	0.9800
C(12)-H(12B)	0.9800
C(12)-H(12C)	0.9800
C(15)-C(16)	1.375(4)
C(15)-H(15)	0.9500
C(7)-C(8)	1.403(3)
C(7)-H(7)	0.9500
C(22)-C(21)	1.500(3)
C(22)-H(22A)	0.9800
C(22)-H(22B)	0.9800
C(22)-H(22C)	0.9800
N(3)-C(21)	1.328(3)
N(3)-C(9)	1.405(3)
C(4)-C(3)	1.352(4)
C(4)-H(4)	0.9500
C(16)-C(17)	1.375(4)
C(16)-H(16)	0.9500
C(10)-C(9)	1.442(3)
C(10)-C(1)	1.447(3)
C(25)-C(26)	1.379(4)
C(25)-C(24)	1.394(3)
C(25)-H(25)	0.9500
C(18)-C(17)	1.392(3)
C(18)-C(20)	1.507(3)
C(24)-C(23)	1.404(3)
C(24)-C(29)	1.509(3)
C(9)-C(8)	1.380(3)
C(19)-H(19A)	0.9800
C(19)-H(19B)	0.9800
C(19)-H(19C)	0.9800
C(29)-H(29A)	0.9800

C(29)-H(29B)	0.9800
C(29)-H(29C)	0.9800
C(17)-H(17)	0.9500
C(8)-H(8)	0.9500
C(3)-C(2)	1.408(3)
C(3)-H(3)	0.9500
C(28)-C(27)	1.399(3)
C(28)-C(23)	1.406(3)
C(28)-C(30)	1.509(3)
C(26)-C(27)	1.369(4)
C(26)-H(26)	0.9500
C(2)-C(1)	1.383(3)
C(2)-H(2)	0.9500
C(27)-H(27)	0.9500
C(20)-H(20A)	0.9800
C(20)-H(20B)	0.9800
C(20)-H(20C)	0.9800
C(30)-H(30A)	0.9800
C(30)-H(30B)	0.9800
C(30)-H(30C)	0.9800
N(3)-Sn(1)-N(1)	72.57(6)
N(3)-Sn(1)-N(4)	57.74(6)
N(1)-Sn(1)-N(4)	107.35(6)
N(3)-Sn(1)-N(2)	112.07(6)
N(1)-Sn(1)-N(2)	57.38(6)
N(4)-Sn(1)-N(2)	95.29(6)
C(21)-N(4)-C(23)	123.17(17)
C(21)-N(4)-Sn(1)	93.11(12)
C(23)-N(4)-Sn(1)	141.84(13)
C(7)-C(6)-C(5)	120.8(2)
C(7)-C(6)-H(6)	119.6
C(5)-C(6)-H(6)	119.6
C(11)-N(2)-C(13)	122.30(17)
C(11)-N(2)-Sn(1)	93.22(12)
C(13)-N(2)-Sn(1)	139.58(13)
C(18)-C(13)-C(14)	120.13(19)
C(18)-C(13)-N(2)	119.73(18)
C(14)-C(13)-N(2)	120.04(19)
C(15)-C(14)-C(13)	118.9(2)
C(15)-C(14)-C(19)	120.2(2)
C(13)-C(14)-C(19)	120.9(2)
C(4)-C(5)-C(6)	119.9(2)
C(4)-C(5)-C(10)	120.1(2)
C(6)-C(5)-C(10)	120.0(2)
C(11)-N(1)-C(1)	128.94(17)
C(11)-N(1)-Sn(1)	97.36(12)
C(1)-N(1)-Sn(1)	133.51(13)
C(11)-C(12)-H(12A)	109.5
C(11)-C(12)-H(12B)	109.5
H(12A)-C(12)-H(12B)	109.5
C(11)-C(12)-H(12C)	109.5
H(12A)-C(12)-H(12C)	109.5
H(12B)-C(12)-H(12C)	109.5

C(16)-C(15)-C(14)	121.1(2)
C(16)-C(15)-H(15)	119.4
C(14)-C(15)-H(15)	119.4
C(6)-C(7)-C(8)	120.5(2)
C(6)-C(7)-H(7)	119.8
C(8)-C(7)-H(7)	119.8
C(21)-C(22)-H(22A)	109.5
C(21)-C(22)-H(22B)	109.5
H(22A)-C(22)-H(22B)	109.5
C(21)-C(22)-H(22C)	109.5
H(22A)-C(22)-H(22C)	109.5
H(22B)-C(22)-H(22C)	109.5
C(21)-N(3)-C(9)	128.79(18)
C(21)-N(3)-Sn(1)	97.47(13)
C(9)-N(3)-Sn(1)	133.17(14)
C(3)-C(4)-C(5)	120.9(2)
C(3)-C(4)-H(4)	119.5
C(5)-C(4)-H(4)	119.5
C(17)-C(16)-C(15)	119.8(2)
C(17)-C(16)-H(16)	120.1
C(15)-C(16)-H(16)	120.1
C(5)-C(10)-C(9)	117.16(19)
C(5)-C(10)-C(1)	117.60(19)
C(9)-C(10)-C(1)	125.23(18)
C(26)-C(25)-C(24)	120.9(2)
C(26)-C(25)-H(25)	119.6
C(24)-C(25)-H(25)	119.6
C(17)-C(18)-C(13)	118.9(2)
C(17)-C(18)-C(20)	121.3(2)
C(13)-C(18)-C(20)	119.9(2)
C(25)-C(24)-C(23)	118.7(2)
C(25)-C(24)-C(29)	120.2(2)
C(23)-C(24)-C(29)	121.06(19)
C(8)-C(9)-N(3)	121.1(2)
C(8)-C(9)-C(10)	120.02(19)
N(3)-C(9)-C(10)	118.82(18)
N(3)-C(21)-N(4)	111.37(17)
N(3)-C(21)-C(22)	125.31(19)
N(4)-C(21)-C(22)	123.32(19)
C(14)-C(19)-H(19A)	109.5
C(14)-C(19)-H(19B)	109.5
H(19A)-C(19)-H(19B)	109.5
C(14)-C(19)-H(19C)	109.5
H(19A)-C(19)-H(19C)	109.5
H(19B)-C(19)-H(19C)	109.5
C(24)-C(29)-H(29A)	109.5
C(24)-C(29)-H(29B)	109.5
H(29A)-C(29)-H(29B)	109.5
C(24)-C(29)-H(29C)	109.5
H(29A)-C(29)-H(29C)	109.5
H(29B)-C(29)-H(29C)	109.5
N(1)-C(11)-N(2)	111.85(17)
N(1)-C(11)-C(12)	126.14(19)
N(2)-C(11)-C(12)	121.89(19)

C(16)-C(17)-C(18)	121.2(2)
C(16)-C(17)-H(17)	119.4
C(18)-C(17)-H(17)	119.4
C(9)-C(8)-C(7)	121.4(2)
C(9)-C(8)-H(8)	119.3
C(7)-C(8)-H(8)	119.3
C(4)-C(3)-C(2)	120.4(2)
C(4)-C(3)-H(3)	119.8
C(2)-C(3)-H(3)	119.8
C(27)-C(28)-C(23)	118.6(2)
C(27)-C(28)-C(30)	119.1(2)
C(23)-C(28)-C(30)	122.3(2)
C(27)-C(26)-C(25)	120.4(2)
C(27)-C(26)-H(26)	119.8
C(25)-C(26)-H(26)	119.8
C(1)-C(2)-C(3)	121.5(2)
C(1)-C(2)-H(2)	119.2
C(3)-C(2)-H(2)	119.2
C(24)-C(23)-C(28)	120.44(19)
C(24)-C(23)-N(4)	118.90(18)
C(28)-C(23)-N(4)	120.63(18)
C(2)-C(1)-N(1)	121.5(2)
C(2)-C(1)-C(10)	119.24(19)
N(1)-C(1)-C(10)	119.12(18)
C(26)-C(27)-C(28)	120.9(2)
C(26)-C(27)-H(27)	119.5
C(28)-C(27)-H(27)	119.5
C(18)-C(20)-H(20A)	109.5
C(18)-C(20)-H(20B)	109.5
H(20A)-C(20)-H(20B)	109.5
C(18)-C(20)-H(20C)	109.5
H(20A)-C(20)-H(20C)	109.5
H(20B)-C(20)-H(20C)	109.5
C(28)-C(30)-H(30A)	109.5
C(28)-C(30)-H(30B)	109.5
H(30A)-C(30)-H(30B)	109.5
C(28)-C(30)-H(30C)	109.5
H(30A)-C(30)-H(30C)	109.5
H(30B)-C(30)-H(30C)	109.5

Symmetry transformations used to generate equivalent atoms:

Table S8. Anisotropic displacement parameters (A^2 x 10^3) for R29.
The anisotropic displacement factor exponent takes the form:
-2 pi^2 [h^2 a*^2 U11 + ... + 2 h k a* b* U12]

	U11	U22	U33	U23	U13	U12
Sn(1)	20(1)	28(1)	26(1)	-4(1)	1(1)	-1(1)
N(4)	24(1)	30(1)	24(1)	-4(1)	1(1)	0(1)
C(6)	33(1)	25(1)	61(2)	9(1)	2(1)	1(1)
N(2)	32(1)	30(1)	27(1)	2(1)	7(1)	2(1)
C(13)	29(1)	28(1)	27(1)	1(1)	7(1)	5(1)
C(14)	32(1)	40(1)	29(1)	-2(1)	6(1)	3(1)
C(5)	25(1)	23(1)	47(1)	-3(1)	0(1)	-2(1)
N(1)	28(1)	28(1)	30(1)	2(1)	6(1)	3(1)
C(12)	43(1)	42(1)	43(1)	8(1)	22(1)	10(1)
C(15)	40(1)	46(1)	29(1)	2(1)	1(1)	13(1)
C(7)	41(1)	38(1)	51(2)	17(1)	7(1)	1(1)
C(22)	35(1)	42(1)	29(1)	2(1)	-2(1)	2(1)
N(3)	32(1)	30(1)	29(1)	-4(1)	1(1)	5(1)
C(4)	38(1)	26(1)	56(1)	-12(1)	-2(1)	4(1)
C(16)	60(2)	33(1)	37(1)	7(1)	8(1)	16(1)
C(10)	21(1)	23(1)	35(1)	-2(1)	2(1)	-4(1)
C(25)	57(2)	36(1)	35(1)	-5(1)	10(1)	13(1)
C(18)	38(1)	28(1)	35(1)	0(1)	0(1)	2(1)
C(24)	36(1)	33(1)	25(1)	2(1)	6(1)	4(1)
C(9)	24(1)	27(1)	37(1)	1(1)	4(1)	-1(1)
C(21)	22(1)	34(1)	26(1)	-2(1)	5(1)	2(1)
C(19)	50(2)	59(2)	43(1)	0(1)	-9(1)	-14(1)
C(29)	32(1)	51(2)	39(1)	0(1)	11(1)	2(1)
C(11)	27(1)	32(1)	27(1)	1(1)	5(1)	4(1)
C(17)	58(2)	26(1)	45(1)	0(1)	0(1)	0(1)
C(8)	37(1)	39(1)	40(1)	8(1)	10(1)	2(1)
C(3)	48(1)	41(1)	48(1)	-19(1)	3(1)	3(1)
C(28)	35(1)	38(1)	25(1)	-1(1)	0(1)	-10(1)
C(26)	77(2)	32(1)	33(1)	-6(1)	-2(1)	-1(1)
C(2)	43(1)	37(1)	33(1)	-7(1)	1(1)	2(1)
C(23)	27(1)	28(1)	22(1)	-1(1)	1(1)	-1(1)
C(1)	24(1)	27(1)	33(1)	-4(1)	0(1)	-1(1)
C(27)	55(2)	37(1)	35(1)	-2(1)	-6(1)	-16(1)
C(20)	56(2)	45(1)	50(2)	1(1)	-16(1)	-6(1)
C(30)	27(1)	61(2)	41(1)	-1(1)	1(1)	-8(1)

Crystal data and structure refinement of compound $(L_2Sn)_2$

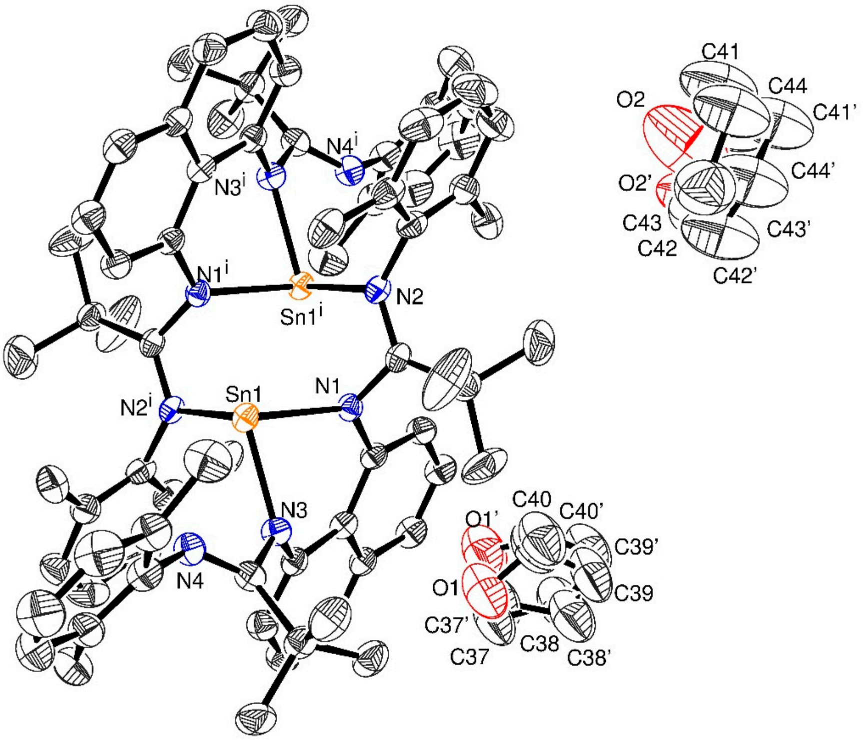

Figure S3. Asymmetric Unit (Symmetry code (i) : 2-x,1-y,1-z)

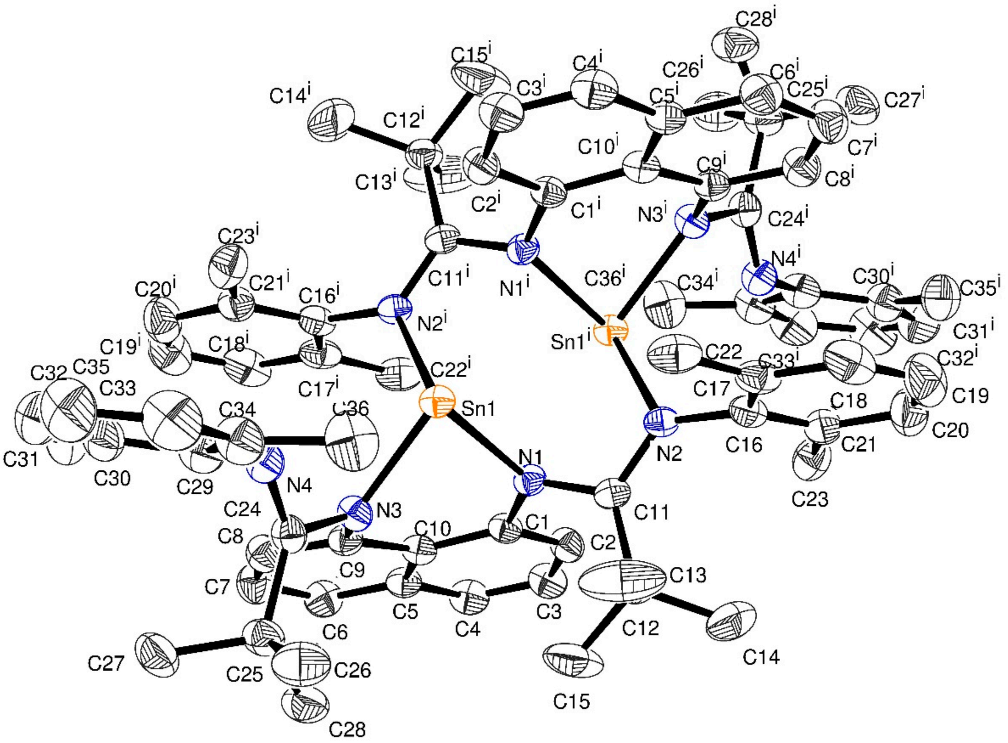

Figure S4. Compound

Table S9. Crystal data and structure refinement for R102.

Identification code		R102
Empirical formula		C72 H84 N8 Sn2, 4(C4 H8 O)
Formula weight		1587.31
Temperature		193(2) K
Wavelength		0.71073 A
Crystal system, space group		Triclinic, P -1
Unit cell dimensions	a = 11.2468(7) A	alpha = 86.510(2) deg.
	b = 12.6512(7) A	beta = 78.588(2) deg.
	c = 14.5050(9) A	gamma = 79.579(2) deg.
Volume		1989.0(2) A^3
Z, Calculated density		1, 1.325 Mg/m^3
Absorption coefficient		0.683 mm^-1

F(000)	832
Crystal size	0.080 x 0.060 x 0.040 mm
Theta range for data collection	3.327 to 29.622 deg.
Limiting indices	-15<=h<=15, -17<=k<=17, -20<=l<=20
Reflections collected / unique	75976 / 11128 [R(int) = 0.0936]
Completeness to theta = 25.242	99.4 %
Refinement method	Full-matrix least-squares on F^2
Data / restraints / parameters	11128 / 277 / 564
Goodness-of-fit on F^2	1.015
Final R indices [I>2sigma(I)]	R1 = 0.0461, wR2 = 0.1054
R indices (all data)	R1 = 0.0747, wR2 = 0.1209
Extinction coefficient	0.0126(10)
Largest diff. peak and hole	1.132 and -0.909 e.A^-3

Table S10. Atomic coordinates (x 10^4) and equivalent isotropic displacement parameters (A^2 x 10^3) for R102.
U(eq) is defined as one third of the trace of the orthogonalized Uij tensor.

	x	y	z	U(eq)
C(1)	7596(3)	5785(2)	4640(2)	25(1)
C(2)	7495(3)	5731(3)	3707(2)	31(1)
C(3)	6786(3)	6554(3)	3259(2)	35(1)
C(4)	6215(3)	7465(3)	3729(2)	34(1)
C(5)	6292(3)	7571(3)	4678(2)	28(1)
C(6)	5712(3)	8527(3)	5142(3)	36(1)
C(7)	5787(3)	8659(3)	6059(3)	39(1)
C(8)	6402(3)	7829(3)	6555(2)	34(1)
C(9)	6931(3)	6843(2)	6151(2)	27(1)
C(10)	6958(3)	6705(2)	5169(2)	25(1)
C(11)	8587(3)	3925(2)	4711(2)	24(1)
C(12)	7481(3)	3306(3)	4982(2)	32(1)
C(13)	7679(4)	2623(5)	5846(4)	80(2)
C(14)	7341(4)	2573(5)	4212(4)	79(2)
C(15)	6253(3)	4048(3)	5221(4)	65(1)
C(16)	10183(3)	2413(2)	4079(2)	30(1)
C(17)	10704(3)	1681(3)	4708(3)	35(1)
C(18)	11205(3)	642(3)	4407(3)	48(1)
C(19)	11215(4)	349(3)	3503(3)	56(1)
C(20)	10720(4)	1087(3)	2882(3)	47(1)
C(21)	10205(3)	2130(3)	3149(2)	35(1)
C(22)	10750(3)	1953(3)	5701(3)	42(1)
C(23)	9669(4)	2923(3)	2451(2)	42(1)
C(24)	7082(3)	5931(3)	7667(2)	29(1)
C(25)	5716(3)	5845(3)	8102(2)	35(1)
C(26)	5707(4)	4703(3)	8496(3)	53(1)
C(27)	5164(4)	6631(4)	8912(3)	49(1)
C(28)	4837(3)	6045(4)	7398(3)	45(1)
C(29)	8024(3)	5543(3)	9082(2)	36(1)
C(30)	7876(3)	6404(3)	9679(2)	40(1)
C(31)	8031(4)	6178(4)	10607(3)	51(1)
C(32)	8340(4)	5144(4)	10931(3)	61(1)
C(33)	8536(4)	4304(4)	10327(3)	55(1)
C(34)	8402(3)	4493(3)	9383(2)	42(1)
C(35)	7613(4)	7554(3)	9352(3)	49(1)
C(36)	8753(4)	3560(3)	8711(3)	54(1)
O(1)	4578(11)	9100(6)	2166(8)	109(2)
C(37)	4034(16)	9949(9)	1700(12)	105(3)
C(38)	3512(15)	9554(9)	922(11)	110(3)
C(39)	3615(14)	8390(9)	1182(10)	109(3)
C(40)	4680(20)	8175(9)	1660(20)	112(3)
O(1')	5176(17)	8998(13)	1611(15)	113(3)
C(37')	4250(20)	9830(16)	1450(20)	111(3)
C(38')	3190(20)	9323(18)	1210(20)	111(3)

C(39')	4020(20)	8359(17)	810(18)	109(3)
C(40')	4720(40)	8043(15)	1570(40)	110(3)
C(43)	7733(10)	41(8)	2503(7)	117(3)
C(42)	7580(11)	919(8)	1851(10)	121(4)
O(2)	8651(14)	719(8)	1069(7)	203(5)
C(41)	9022(13)	-427(9)	1098(8)	146(4)
C(44)	8468(11)	-823(8)	1925(8)	135(4)
C(43')	7380(60)	-270(30)	2190(30)	140(5)
C(42')	7010(60)	830(30)	2100(40)	140(6)
O(2')	7840(50)	1200(30)	1290(30)	142(6)
C(41')	8420(60)	220(40)	780(30)	143(5)
C(44')	7930(60)	-620(30)	1250(40)	141(5)
N(1)	8441(2)	4967(2)	4998(2)	24(1)
N(2)	9719(2)	3514(2)	4329(2)	25(1)
N(3)	7461(2)	6003(2)	6691(2)	27(1)
N(4)	7988(3)	5748(2)	8112(2)	31(1)
Sn(1)	9263(1)	5074(1)	6270(1)	25(1)

Table S11. Bond lengths [A] and angles [deg] for R102.

C(1)-C(2)	1.387(4)
C(1)-N(1)	1.421(4)
C(1)-C(10)	1.431(4)
C(2)-C(3)	1.401(4)
C(2)-H(2)	0.9500
C(3)-C(4)	1.364(5)
C(3)-H(3)	0.9500
C(4)-C(5)	1.412(4)
C(4)-H(4)	0.9500
C(5)-C(6)	1.408(5)
C(5)-C(10)	1.444(4)
C(6)-C(7)	1.371(5)
C(6)-H(6)	0.9500
C(7)-C(8)	1.394(5)
C(7)-H(7)	0.9500
C(8)-C(9)	1.391(5)
C(8)-H(8)	0.9500
C(9)-N(3)	1.397(4)
C(9)-C(10)	1.440(4)
C(11)-N(2)	1.309(4)
C(11)-N(1)	1.378(4)
C(11)-C(12)	1.561(4)
C(12)-C(13)	1.508(5)
C(12)-C(15)	1.513(5)
C(12)-C(14)	1.543(6)
C(13)-H(13A)	0.9800
C(13)-H(13B)	0.9800
C(13)-H(13C)	0.9800
C(14)-H(14A)	0.9800
C(14)-H(14B)	0.9800
C(14)-H(14C)	0.9800
C(15)-H(15A)	0.9800
C(15)-H(15B)	0.9800
C(15)-H(15C)	0.9800
C(16)-C(17)	1.395(4)
C(16)-C(21)	1.410(5)
C(16)-N(2)	1.438(4)
C(17)-C(18)	1.393(5)
C(17)-C(22)	1.515(5)
C(18)-C(19)	1.381(6)
C(18)-H(18)	0.9500
C(19)-C(20)	1.379(6)
C(19)-H(19)	0.9500
C(20)-C(21)	1.386(5)
C(20)-H(20)	0.9500
C(21)-C(23)	1.515(5)
C(22)-H(22A)	0.9800
C(22)-H(22B)	0.9800
C(22)-H(22C)	0.9800
C(23)-H(23A)	0.9800
C(23)-H(23B)	0.9800

C(23)-H(23C)	0.9800
C(24)-N(4)	1.291(4)
C(24)-N(3)	1.398(4)
C(24)-C(25)	1.562(4)
C(25)-C(26)	1.522(5)
C(25)-C(28)	1.538(5)
C(25)-C(27)	1.544(5)
C(26)-H(26A)	0.9800
C(26)-H(26B)	0.9800
C(26)-H(26C)	0.9800
C(27)-H(27A)	0.9800
C(27)-H(27B)	0.9800
C(27)-H(27C)	0.9800
C(28)-H(28A)	0.9800
C(28)-H(28B)	0.9800
C(28)-H(28C)	0.9800
C(29)-C(34)	1.391(5)
C(29)-C(30)	1.398(5)
C(29)-N(4)	1.422(4)
C(30)-C(31)	1.397(5)
C(30)-C(35)	1.498(5)
C(31)-C(32)	1.370(6)
C(31)-H(31)	0.9500
C(32)-C(33)	1.377(6)
C(32)-H(32)	0.9500
C(33)-C(34)	1.408(5)
C(33)-H(33)	0.9500
C(34)-C(36)	1.523(6)
C(35)-H(35A)	0.9800
C(35)-H(35B)	0.9800
C(35)-H(35C)	0.9800
C(36)-H(36A)	0.9800
C(36)-H(36B)	0.9800
C(36)-H(36C)	0.9800
O(1)-C(37)	1.349(11)
O(1)-C(40)	1.395(14)
C(37)-C(38)	1.519(13)
C(37)-H(37A)	0.9900
C(37)-H(37B)	0.9900
C(38)-C(39)	1.486(14)
C(38)-H(38A)	0.9900
C(38)-H(38B)	0.9900
C(39)-C(40)	1.470(13)
C(39)-H(39A)	0.9900
C(39)-H(39B)	0.9900
C(40)-H(40A)	0.9900
C(40)-H(40B)	0.9900
O(1')-C(37')	1.385(15)
O(1')-C(40')	1.41(2)
C(37')-C(38')	1.557(18)
C(37')-H(37C)	0.9900
C(37')-H(37D)	0.9900
C(38')-C(39')	1.469(18)
C(38')-H(38C)	0.9900

C(38')-H(38D)	0.9900
C(39')-C(40')	1.466(18)
C(39')-H(39C)	0.9900
C(39')-H(39D)	0.9900
C(40')-H(40C)	0.9900
C(40')-H(40D)	0.9900
C(43)-C(42)	1.423(12)
C(43)-C(44)	1.446(13)
C(43)-H(43A)	0.9900
C(43)-H(43B)	0.9900
C(42)-O(2)	1.478(12)
C(42)-H(42A)	0.9900
C(42)-H(42B)	0.9900
O(2)-C(41)	1.435(12)
C(41)-C(44)	1.350(12)
C(41)-H(41A)	0.9900
C(41)-H(41B)	0.9900
C(44)-H(44A)	0.9900
C(44)-H(44B)	0.9900
C(43')-C(42')	1.389(18)
C(43')-C(44')	1.44(2)
C(43')-H(43C)	0.9900
C(43')-H(43D)	0.9900
C(42')-O(2')	1.454(19)
C(42')-H(42C)	0.9900
C(42')-H(42D)	0.9900
O(2')-C(41')	1.464(18)
C(41')-C(44')	1.374(18)
C(41')-H(41C)	0.9900
C(41')-H(41D)	0.9900
C(44')-H(44C)	0.9900
C(44')-H(44D)	0.9900
N(1)-Sn(1)	2.243(2)
N(2)-Sn(1)#1	2.327(2)
N(3)-Sn(1)	2.144(3)
C(2)-C(1)-N(1)	117.6(3)
C(2)-C(1)-C(10)	119.5(3)
N(1)-C(1)-C(10)	122.6(3)
C(1)-C(2)-C(3)	122.1(3)
C(1)-C(2)-H(2)	119.0
C(3)-C(2)-H(2)	119.0
C(4)-C(3)-C(2)	119.9(3)
C(4)-C(3)-H(3)	120.0
C(2)-C(3)-H(3)	120.0
C(3)-C(4)-C(5)	120.5(3)
C(3)-C(4)-H(4)	119.7
C(5)-C(4)-H(4)	119.7
C(6)-C(5)-C(4)	119.4(3)
C(6)-C(5)-C(10)	120.3(3)
C(4)-C(5)-C(10)	120.3(3)
C(7)-C(6)-C(5)	120.7(3)
C(7)-C(6)-H(6)	119.6
C(5)-C(6)-H(6)	119.6

C(6)-C(7)-C(8)	120.2(3)
C(6)-C(7)-H(7)	119.9
C(8)-C(7)-H(7)	119.9
C(9)-C(8)-C(7)	121.7(3)
C(9)-C(8)-H(8)	119.2
C(7)-C(8)-H(8)	119.2
C(8)-C(9)-N(3)	120.0(3)
C(8)-C(9)-C(10)	119.6(3)
N(3)-C(9)-C(10)	120.4(3)
C(1)-C(10)-C(9)	125.3(3)
C(1)-C(10)-C(5)	117.5(3)
C(9)-C(10)-C(5)	117.2(3)
N(2)-C(11)-N(1)	114.2(3)
N(2)-C(11)-C(12)	126.3(3)
N(1)-C(11)-C(12)	119.0(3)
C(13)-C(12)-C(15)	108.0(4)
C(13)-C(12)-C(14)	108.7(4)
C(15)-C(12)-C(14)	105.5(4)
C(13)-C(12)-C(11)	106.9(3)
C(15)-C(12)-C(11)	112.8(3)
C(14)-C(12)-C(11)	114.7(3)
C(12)-C(13)-H(13A)	109.5
C(12)-C(13)-H(13B)	109.5
H(13A)-C(13)-H(13B)	109.5
C(12)-C(13)-H(13C)	109.5
H(13A)-C(13)-H(13C)	109.5
H(13B)-C(13)-H(13C)	109.5
C(12)-C(14)-H(14A)	109.5
C(12)-C(14)-H(14B)	109.5
H(14A)-C(14)-H(14B)	109.5
C(12)-C(14)-H(14C)	109.5
H(14A)-C(14)-H(14C)	109.5
H(14B)-C(14)-H(14C)	109.5
C(12)-C(15)-H(15A)	109.5
C(12)-C(15)-H(15B)	109.5
H(15A)-C(15)-H(15B)	109.5
C(12)-C(15)-H(15C)	109.5
H(15A)-C(15)-H(15C)	109.5
H(15B)-C(15)-H(15C)	109.5
C(17)-C(16)-C(21)	121.3(3)
C(17)-C(16)-N(2)	120.7(3)
C(21)-C(16)-N(2)	117.7(3)
C(18)-C(17)-C(16)	118.2(3)
C(18)-C(17)-C(22)	118.2(3)
C(16)-C(17)-C(22)	123.6(3)
C(19)-C(18)-C(17)	121.1(4)
C(19)-C(18)-H(18)	119.4
C(17)-C(18)-H(18)	119.4
C(20)-C(19)-C(18)	120.1(4)
C(20)-C(19)-H(19)	120.0
C(18)-C(19)-H(19)	120.0
C(19)-C(20)-C(21)	121.0(4)
C(19)-C(20)-H(20)	119.5
C(21)-C(20)-H(20)	119.5

C(20)-C(21)-C(16)	118.3(3)
C(20)-C(21)-C(23)	119.8(3)
C(16)-C(21)-C(23)	121.9(3)
C(17)-C(22)-H(22A)	109.5
C(17)-C(22)-H(22B)	109.5
H(22A)-C(22)-H(22B)	109.5
C(17)-C(22)-H(22C)	109.5
H(22A)-C(22)-H(22C)	109.5
H(22B)-C(22)-H(22C)	109.5
C(21)-C(23)-H(23A)	109.5
C(21)-C(23)-H(23B)	109.5
H(23A)-C(23)-H(23B)	109.5
C(21)-C(23)-H(23C)	109.5
H(23A)-C(23)-H(23C)	109.5
H(23B)-C(23)-H(23C)	109.5
N(4)-C(24)-N(3)	113.1(3)
N(4)-C(24)-C(25)	125.5(3)
N(3)-C(24)-C(25)	120.2(3)
C(26)-C(25)-C(28)	107.2(3)
C(26)-C(25)-C(27)	108.4(3)
C(28)-C(25)-C(27)	106.9(3)
C(26)-C(25)-C(24)	107.2(3)
C(28)-C(25)-C(24)	114.6(3)
C(27)-C(25)-C(24)	112.4(3)
C(25)-C(26)-H(26A)	109.5
C(25)-C(26)-H(26B)	109.5
H(26A)-C(26)-H(26B)	109.5
C(25)-C(26)-H(26C)	109.5
H(26A)-C(26)-H(26C)	109.5
H(26B)-C(26)-H(26C)	109.5
C(25)-C(27)-H(27A)	109.5
C(25)-C(27)-H(27B)	109.5
H(27A)-C(27)-H(27B)	109.5
C(25)-C(27)-H(27C)	109.5
H(27A)-C(27)-H(27C)	109.5
H(27B)-C(27)-H(27C)	109.5
C(25)-C(28)-H(28A)	109.5
C(25)-C(28)-H(28B)	109.5
H(28A)-C(28)-H(28B)	109.5
C(25)-C(28)-H(28C)	109.5
H(28A)-C(28)-H(28C)	109.5
H(28B)-C(28)-H(28C)	109.5
C(34)-C(29)-C(30)	120.8(3)
C(34)-C(29)-N(4)	118.6(3)
C(30)-C(29)-N(4)	119.7(3)
C(31)-C(30)-C(29)	118.4(4)
C(31)-C(30)-C(35)	118.9(4)
C(29)-C(30)-C(35)	122.6(3)
C(32)-C(31)-C(30)	121.6(4)
C(32)-C(31)-H(31)	119.2
C(30)-C(31)-H(31)	119.2
C(31)-C(32)-C(33)	119.7(4)
C(31)-C(32)-H(32)	120.2
C(33)-C(32)-H(32)	120.2

C(32)-C(33)-C(34)	120.8(4)
C(32)-C(33)-H(33)	119.6
C(34)-C(33)-H(33)	119.6
C(29)-C(34)-C(33)	118.6(4)
C(29)-C(34)-C(36)	122.0(3)
C(33)-C(34)-C(36)	119.3(4)
C(30)-C(35)-H(35A)	109.5
C(30)-C(35)-H(35B)	109.5
H(35A)-C(35)-H(35B)	109.5
C(30)-C(35)-H(35C)	109.5
H(35A)-C(35)-H(35C)	109.5
H(35B)-C(35)-H(35C)	109.5
C(34)-C(36)-H(36A)	109.5
C(34)-C(36)-H(36B)	109.5
H(36A)-C(36)-H(36B)	109.5
C(34)-C(36)-H(36C)	109.5
H(36A)-C(36)-H(36C)	109.5
H(36B)-C(36)-H(36C)	109.5
C(37)-O(1)-C(40)	108.7(9)
O(1)-C(37)-C(38)	109.6(8)
O(1)-C(37)-H(37A)	109.7
C(38)-C(37)-H(37A)	109.7
O(1)-C(37)-H(37B)	109.7
C(38)-C(37)-H(37B)	109.7
H(37A)-C(37)-H(37B)	108.2
C(39)-C(38)-C(37)	100.4(10)
C(39)-C(38)-H(38A)	111.7
C(37)-C(38)-H(38A)	111.7
C(39)-C(38)-H(38B)	111.7
C(37)-C(38)-H(38B)	111.7
H(38A)-C(38)-H(38B)	109.5
C(40)-C(39)-C(38)	103.6(10)
C(40)-C(39)-H(39A)	111.0
C(38)-C(39)-H(39A)	111.0
C(40)-C(39)-H(39B)	111.0
C(38)-C(39)-H(39B)	111.0
H(39A)-C(39)-H(39B)	109.0
O(1)-C(40)-C(39)	104.5(11)
O(1)-C(40)-H(40A)	110.9
C(39)-C(40)-H(40A)	110.9
O(1)-C(40)-H(40B)	110.9
C(39)-C(40)-H(40B)	110.9
H(40A)-C(40)-H(40B)	108.9
C(37')-O(1')-C(40')	106.0(16)
O(1')-C(37')-C(38')	107.7(13)
O(1')-C(37')-H(37C)	110.2
C(38')-C(37')-H(37C)	110.2
O(1')-C(37')-H(37D)	110.2
C(38')-C(37')-H(37D)	110.2
H(37C)-C(37')-H(37D)	108.5
C(39')-C(38')-C(37')	93.6(15)
C(39')-C(38')-H(38C)	113.0
C(37')-C(38')-H(38C)	113.0
C(39')-C(38')-H(38D)	113.0

C(37')-C(38')-H(38D)	113.0
H(38C)-C(38')-H(38D)	110.4
C(40')-C(39')-C(38')	100.3(18)
C(40')-C(39')-H(39C)	111.7
C(38')-C(39')-H(39C)	111.7
C(40')-C(39')-H(39D)	111.7
C(38')-C(39')-H(39D)	111.7
H(39C)-C(39')-H(39D)	109.5
O(1')-C(40')-C(39')	99.1(18)
O(1')-C(40')-H(40C)	111.9
C(39')-C(40')-H(40C)	111.9
O(1')-C(40')-H(40D)	111.9
C(39')-C(40')-H(40D)	111.9
H(40C)-C(40')-H(40D)	109.6
C(42)-C(43)-C(44)	103.6(9)
C(42)-C(43)-H(43A)	111.0
C(44)-C(43)-H(43A)	111.0
C(42)-C(43)-H(43B)	111.0
C(44)-C(43)-H(43B)	111.0
H(43A)-C(43)-H(43B)	109.0
C(43)-C(42)-O(2)	106.2(8)
C(43)-C(42)-H(42A)	110.5
O(2)-C(42)-H(42A)	110.5
C(43)-C(42)-H(42B)	110.5
O(2)-C(42)-H(42B)	110.5
H(42A)-C(42)-H(42B)	108.7
C(41)-O(2)-C(42)	103.7(9)
C(44)-C(41)-O(2)	108.9(8)
C(44)-C(41)-H(41A)	109.9
O(2)-C(41)-H(41A)	109.9
C(44)-C(41)-H(41B)	109.9
O(2)-C(41)-H(41B)	109.9
H(41A)-C(41)-H(41B)	108.3
C(41)-C(44)-C(43)	110.4(8)
C(41)-C(44)-H(44A)	109.6
C(43)-C(44)-H(44A)	109.6
C(41)-C(44)-H(44B)	109.6
C(43)-C(44)-H(44B)	109.6
H(44A)-C(44)-H(44B)	108.1
C(42')-C(43')-C(44')	105(2)
C(42')-C(43')-H(43C)	110.7
C(44')-C(43')-H(43C)	110.7
C(42')-C(43')-H(43D)	110.7
C(44')-C(43')-H(43D)	110.7
H(43C)-C(43')-H(43D)	108.8
C(43')-C(42')-O(2')	107(2)
C(43')-C(42')-H(42C)	110.4
O(2')-C(42')-H(42C)	110.4
C(43')-C(42')-H(42D)	110.4
O(2')-C(42')-H(42D)	110.4
H(42C)-C(42')-H(42D)	108.6
C(42')-O(2')-C(41')	104(2)
C(44')-C(41')-O(2')	107.8(18)
C(44')-C(41')-H(41C)	110.1

O(2')-C(41')-H(41C)	110.2
C(44')-C(41')-H(41D)	110.2
O(2')-C(41')-H(41D)	110.2
H(41C)-C(41')-H(41D)	108.5
C(41')-C(44')-C(43')	106.7(19)
C(41')-C(44')-H(44C)	110.4
C(43')-C(44')-H(44C)	110.4
C(41')-C(44')-H(44D)	110.4
C(43')-C(44')-H(44D)	110.4
H(44C)-C(44')-H(44D)	108.6
C(11)-N(1)-C(1)	119.7(2)
C(11)-N(1)-Sn(1)	112.43(18)
C(1)-N(1)-Sn(1)	126.11(19)
C(11)-N(2)-C(16)	126.8(3)
C(11)-N(2)-Sn(1)#1	107.94(19)
C(16)-N(2)-Sn(1)#1	123.20(19)
C(9)-N(3)-C(24)	121.8(3)
C(9)-N(3)-Sn(1)	124.34(19)
C(24)-N(3)-Sn(1)	110.35(19)
C(24)-N(4)-C(29)	131.6(3)
N(3)-Sn(1)-N(1)	78.80(9)
N(3)-Sn(1)-N(2)#1	97.73(9)
N(1)-Sn(1)-N(2)#1	95.21(9)

Symmetry transformations used to generate equivalent atoms:
#1 -x+2,-y+1,-z+1

Table S12. Anisotropic displacement parameters (A^2 x 10^3) for R102.
The anisotropic displacement factor exponent takes the form:
-2 pi^2 [h^2 a*^2 U11 + ... + 2 h k a* b* U12]

	U11	U22	U33	U23	U13	U12
C(1)	22(1)	29(2)	24(1)	3(1)	-6(1)	-3(1)
C(2)	30(2)	38(2)	24(2)	-1(1)	-6(1)	-2(1)
C(3)	33(2)	47(2)	24(2)	6(1)	-9(1)	-3(2)
C(4)	34(2)	40(2)	30(2)	10(1)	-12(1)	-3(1)
C(5)	25(2)	31(2)	29(2)	5(1)	-8(1)	-5(1)
C(6)	36(2)	31(2)	42(2)	4(1)	-13(2)	0(1)
C(7)	41(2)	32(2)	41(2)	-2(2)	-9(2)	0(2)
C(8)	35(2)	37(2)	29(2)	-1(1)	-6(1)	-5(1)
C(9)	25(1)	30(2)	26(1)	2(1)	-6(1)	-6(1)
C(10)	23(1)	28(2)	24(1)	6(1)	-4(1)	-5(1)
C(11)	24(1)	27(2)	24(1)	6(1)	-10(1)	-5(1)
C(12)	25(2)	33(2)	38(2)	0(1)	-5(1)	-7(1)
C(13)	51(3)	112(4)	85(4)	63(3)	-27(3)	-44(3)
C(14)	52(3)	103(4)	89(4)	-41(3)	8(3)	-46(3)
C(15)	26(2)	44(2)	117(4)	6(2)	6(2)	-10(2)
C(16)	22(1)	26(2)	39(2)	2(1)	-3(1)	-4(1)
C(17)	25(2)	30(2)	49(2)	9(1)	-5(1)	-7(1)
C(18)	38(2)	32(2)	65(3)	13(2)	0(2)	-3(2)
C(19)	58(3)	26(2)	75(3)	-4(2)	6(2)	-5(2)
C(20)	54(2)	36(2)	50(2)	-9(2)	3(2)	-12(2)
C(21)	30(2)	34(2)	39(2)	-3(1)	-1(1)	-10(1)
C(22)	35(2)	44(2)	50(2)	15(2)	-15(2)	-9(2)
C(23)	51(2)	43(2)	34(2)	-3(2)	-10(2)	-14(2)
C(24)	33(2)	30(2)	22(1)	1(1)	-2(1)	-6(1)
C(25)	30(2)	50(2)	26(2)	2(1)	-3(1)	-8(2)
C(26)	45(2)	58(3)	56(2)	13(2)	-5(2)	-20(2)
C(27)	37(2)	73(3)	33(2)	-10(2)	2(2)	-3(2)
C(28)	32(2)	69(3)	34(2)	1(2)	-4(2)	-13(2)
C(29)	33(2)	48(2)	25(2)	6(1)	-6(1)	-5(2)
C(30)	41(2)	50(2)	27(2)	-1(2)	-5(1)	-5(2)
C(31)	55(2)	75(3)	25(2)	-4(2)	-12(2)	-8(2)
C(32)	69(3)	86(3)	25(2)	7(2)	-17(2)	1(3)
C(33)	60(3)	66(3)	36(2)	18(2)	-16(2)	1(2)
C(34)	43(2)	52(2)	29(2)	7(2)	-6(2)	-5(2)
C(35)	56(2)	50(2)	40(2)	-6(2)	-7(2)	-8(2)
C(36)	66(3)	44(2)	48(2)	8(2)	-15(2)	1(2)
O(1)	152(5)	77(3)	112(5)	-14(4)	-65(4)	-7(4)
C(37)	145(6)	65(4)	119(6)	-22(4)	-58(5)	-9(4)
C(38)	148(6)	75(4)	117(6)	-9(4)	-63(5)	-2(4)
C(39)	151(6)	71(4)	121(6)	-20(4)	-64(5)	-15(4)
C(40)	149(5)	71(4)	122(6)	-8(4)	-56(5)	-3(4)
O(1')	145(6)	78(4)	128(6)	-15(5)	-57(5)	-14(4)
C(37')	151(6)	71(4)	123(7)	-16(5)	-59(5)	-10(4)
C(38')	147(6)	75(5)	119(7)	-19(5)	-60(5)	-1(5)
C(39')	149(6)	70(4)	118(7)	-21(5)	-56(5)	-4(5)

C(40')	148(6)	70(4)	122(6)	-13(5)	-56(5)	-7(5)
C(43)	113(8)	98(7)	134(9)	-3(6)	-9(6)	-15(6)
C(42)	111(9)	99(7)	150(11)	-4(8)	-26(8)	-8(6)
O(2)	331(16)	133(8)	126(7)	-4(6)	5(9)	-46(10)
C(41)	175(9)	118(7)	114(6)	-25(6)	27(6)	10(6)
C(44)	170(9)	106(6)	102(6)	-17(5)	13(6)	10(6)
C(43')	172(11)	115(9)	106(8)	-18(8)	16(8)	9(8)
C(42')	171(13)	116(10)	103(10)	-14(9)	17(10)	14(10)
O(2')	170(12)	118(9)	104(10)	-12(9)	17(10)	17(10)
C(41')	173(11)	117(9)	106(8)	-18(8)	17(8)	15(8)
C(44')	172(10)	116(8)	107(8)	-22(7)	18(8)	10(8)
N(1)	25(1)	27(1)	22(1)	0(1)	-7(1)	-5(1)
N(2)	22(1)	26(1)	26(1)	2(1)	-7(1)	-5(1)
N(3)	28(1)	33(1)	19(1)	3(1)	-5(1)	-3(1)
N(4)	35(2)	37(2)	21(1)	3(1)	-8(1)	-5(1)
Sn(1)	25(1)	28(1)	21(1)	4(1)	-7(1)	-5(1)

Crystal data and structure refinement of compound $(L_1Ge)_2$

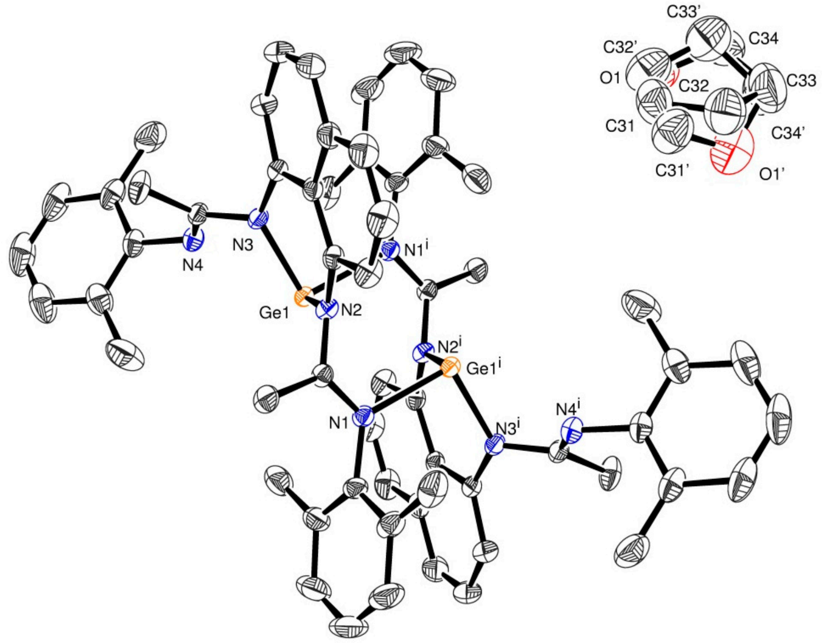

Figure S5. Asymmetric Unit [Symmetry code : (i) 1-x, 1-y, 1-z]

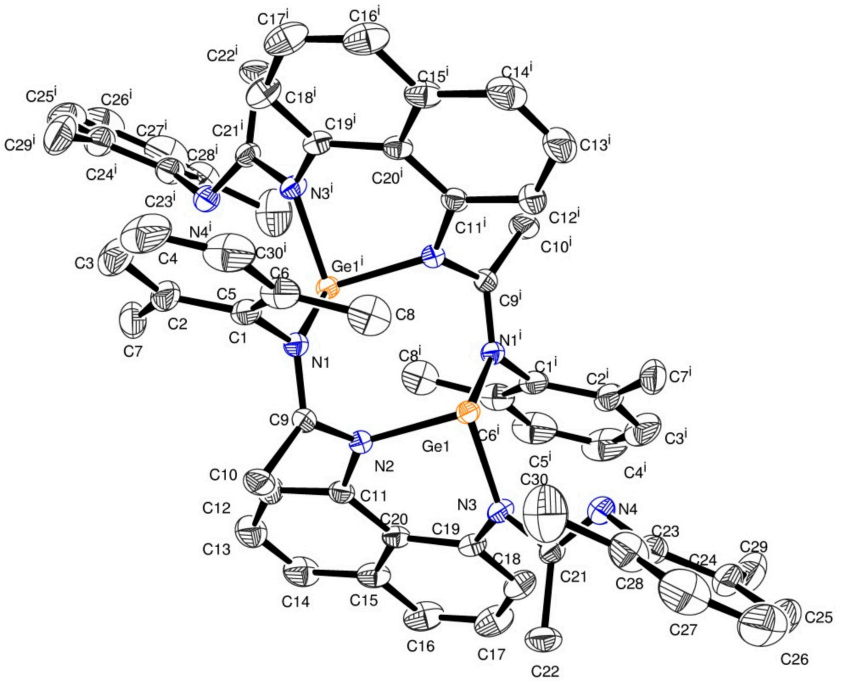

Figure S6. Compound [Symmetry code : (i) 1-x, 1-y, 1-z]

Table S13. Crystal data and structure refinement for AA-R34.

Identification code	AA-R34
Empirical formula	C60 H60 Ge2 N8, 2(C4 H8 O)
Formula weight	1182.59
Temperature	193(2) K
Wavelength	0.71073 A
Crystal system, space group	Triclinic, P -1
Unit cell dimensions	a = 9.1577(4) A alpha = 65.967(2) deg.<br>b = 12.9975(5) A beta = 73.850(2) deg.<br>c = 14.4975(5) A gamma = 83.201(2) deg.
Volume	1513.79(11) A^3
Z, Calculated density	1, 1.297 Mg/m^3
Absorption coefficient	1.044 mm^-1
F(000)	620
Crystal size	0.200 x 0.080 x 0.060 mm
Theta range for data collection	2.874 to 34.433 deg.
Limiting indices	-14<=h<=14, -20<=k<=20, -23<=l<=21
Reflections collected / unique	62134 / 12689 [R(int) = 0.0398]
Completeness to theta = 25.242	99.5 %
Refinement method	Full-matrix least-squares on F^2
Data / restraints / parameters	12689 / 186 / 414
Goodness-of-fit on F^2	1.051
Final R indices [I>2sigma(I)]	R1 = 0.0381, wR2 = 0.0898
R indices (all data)	R1 = 0.0527, wR2 = 0.0964
Largest diff. peak and hole	0.674 and -0.468 e.A^-3

Table S14. Atomic coordinates (x 10^4) and equivalent isotropic displacement parameters (A^2 x 10^3) for AA-R34.
U(eq) is defined as one third of the trace of the orthogonalized Uij tensor.

	x	y	z	U(eq)
C(1)	5002(2)	4429(1)	7510(1)	26(1)
C(2)	5066(2)	3390(2)	8339(1)	35(1)
C(3)	4578(2)	3367(2)	9350(1)	51(1)
C(4)	4064(2)	4323(2)	9526(2)	59(1)
C(5)	4052(2)	5347(2)	8696(2)	50(1)
C(6)	4523(2)	5419(2)	7669(1)	33(1)
C(7)	5668(2)	2348(2)	8151(1)	42(1)
C(8)	4540(2)	6536(2)	6771(2)	40(1)
C(9)	6772(1)	4662(1)	5873(1)	20(1)
C(10)	8088(2)	4774(1)	6255(1)	30(1)
C(11)	8077(1)	4261(1)	4369(1)	21(1)
C(12)	8404(2)	3167(1)	4984(1)	30(1)
C(13)	9418(2)	2482(1)	4552(2)	39(1)
C(14)	10050(2)	2866(1)	3507(2)	38(1)
C(15)	9725(2)	3965(1)	2840(1)	29(1)
C(16)	10386(2)	4347(2)	1752(1)	39(1)
C(17)	10089(2)	5406(2)	1096(1)	40(1)
C(18)	9133(2)	6137(1)	1500(1)	31(1)
C(19)	8510(1)	5817(1)	2560(1)	22(1)
C(20)	8744(1)	4698(1)	3267(1)	21(1)
C(21)	8118(1)	7709(1)	2521(1)	21(1)
C(22)	9799(2)	7948(1)	2184(1)	35(1)
C(23)	7531(2)	9586(1)	2280(1)	28(1)
C(24)	7771(2)	10359(1)	1239(1)	36(1)
C(25)	8141(2)	11472(2)	1003(2)	52(1)
C(26)	8267(3)	11801(2)	1762(2)	60(1)
C(27)	7999(2)	11041(2)	2785(2)	55(1)
C(28)	7608(2)	9924(2)	3071(2)	39(1)
C(29)	7619(3)	9999(2)	413(1)	50(1)
C(30)	7269(3)	9095(2)	4187(2)	59(1)
O(1)	2910(4)	1766(2)	1930(2)	76(1)
C(31)	3761(5)	1192(4)	2702(4)	74(1)
C(32)	3318(5)	-47(4)	3259(4)	81(1)
C(33)	2147(10)	-147(6)	2799(5)	93(2)
C(34)	2378(5)	886(4)	1778(4)	79(1)
O(1')	2125(11)	254(9)	3602(7)	97(2)
C(31')	3548(13)	772(13)	3286(8)	78(2)
C(32')	3854(18)	1449(13)	2118(10)	81(2)
C(33')	3102(19)	812(15)	1777(10)	90(2)
C(34')	1980(30)	50(20)	2741(11)	88(3)
Ge(1)	5879(1)	6276(1)	4108(1)	18(1)
N(1)	5383(1)	4465(1)	6463(1)	20(1)
N(2)	6962(1)	4871(1)	4839(1)	20(1)
N(3)	7640(1)	6605(1)	2931(1)	21(1)

N(4)	7093(1)	8469(1)	2558(1)	25(1)

Table S15. Bond lengths [A] and angles [deg] for AA-R34.

C(1)-C(6)	1.397(2)
C(1)-C(2)	1.403(2)
C(1)-N(1)	1.4419(16)
C(2)-C(3)	1.397(2)
C(2)-C(7)	1.502(3)
C(3)-C(4)	1.372(4)
C(3)-H(3)	0.9500
C(4)-C(5)	1.386(3)
C(4)-H(4)	0.9500
C(5)-C(6)	1.397(2)
C(5)-H(5)	0.9500
C(6)-C(8)	1.502(3)
C(7)-H(7A)	0.9800
C(7)-H(7B)	0.9800
C(7)-H(7C)	0.9800
C(8)-H(8A)	0.9800
C(8)-H(8B)	0.9800
C(8)-H(8C)	0.9800
C(9)-N(1)	1.3074(16)
C(9)-N(2)	1.3740(16)
C(9)-C(10)	1.5027(18)
C(10)-H(10A)	0.9800
C(10)-H(10B)	0.9800
C(10)-H(10C)	0.9800
C(11)-C(12)	1.3867(18)
C(11)-N(2)	1.4125(16)
C(11)-C(20)	1.4317(18)
C(12)-C(13)	1.408(2)
C(12)-H(12)	0.9500
C(13)-C(14)	1.358(3)
C(13)-H(13)	0.9500
C(14)-C(15)	1.414(2)
C(14)-H(14)	0.9500
C(15)-C(16)	1.416(2)
C(15)-C(20)	1.4373(18)
C(16)-C(17)	1.364(3)
C(16)-H(16)	0.9500
C(17)-C(18)	1.408(2)
C(17)-H(17)	0.9500
C(18)-C(19)	1.3815(18)
C(18)-H(18)	0.9500
C(19)-N(3)	1.4102(16)
C(19)-C(20)	1.4305(18)
C(21)-N(4)	1.2869(17)
C(21)-N(3)	1.3831(16)
C(21)-C(22)	1.5089(19)
C(22)-H(22A)	0.9800
C(22)-H(22B)	0.9800
C(22)-H(22C)	0.9800
C(23)-C(24)	1.402(2)
C(23)-C(28)	1.404(2)

C(23)-N(4)	1.4153(18)
C(24)-C(25)	1.406(2)
C(24)-C(29)	1.496(3)
C(25)-C(26)	1.368(4)
C(25)-H(25)	0.9500
C(26)-C(27)	1.377(4)
C(26)-H(26)	0.9500
C(27)-C(28)	1.400(3)
C(27)-H(27)	0.9500
C(28)-C(30)	1.502(3)
C(29)-H(29A)	0.9800
C(29)-H(29B)	0.9800
C(29)-H(29C)	0.9800
C(30)-H(30A)	0.9800
C(30)-H(30B)	0.9800
C(30)-H(30C)	0.9800
O(1)-C(34)	1.410(5)
O(1)-C(31)	1.443(5)
C(31)-C(32)	1.524(6)
C(31)-H(31A)	0.9900
C(31)-H(31B)	0.9900
C(32)-C(33)	1.454(8)
C(32)-H(32A)	0.9900
C(32)-H(32B)	0.9900
C(33)-C(34)	1.521(7)
C(33)-H(33A)	0.9900
C(33)-H(33B)	0.9900
C(34)-H(34A)	0.9900
C(34)-H(34B)	0.9900
O(1')-C(31')	1.410(9)
O(1')-C(34')	1.421(11)
C(31')-C(32')	1.517(10)
C(31')-H(31C)	0.9900
C(31')-H(31D)	0.9900
C(32')-C(33')	1.436(11)
C(32')-H(32C)	0.9900
C(32')-H(32D)	0.9900
C(33')-C(34')	1.517(10)
C(33')-H(33C)	0.9900
C(33')-H(33D)	0.9900
C(34')-H(34C)	0.9900
C(34')-H(34D)	0.9900
Ge(1)-N(3)	1.9332(10)
Ge(1)-N(2)	2.0048(10)
Ge(1)-N(1)#1	2.0961(11)

C(6)-C(1)-C(2)	122.28(14)
C(6)-C(1)-N(1)	118.95(13)
C(2)-C(1)-N(1)	118.71(14)
C(3)-C(2)-C(1)	117.35(18)
C(3)-C(2)-C(7)	121.06(16)
C(1)-C(2)-C(7)	121.57(14)
C(4)-C(3)-C(2)	121.36(19)
C(4)-C(3)-H(3)	119.3

C(2)-C(3)-H(3)	119.3
C(3)-C(4)-C(5)	120.39(17)
C(3)-C(4)-H(4)	119.8
C(5)-C(4)-H(4)	119.8
C(4)-C(5)-C(6)	120.7(2)
C(4)-C(5)-H(5)	119.7
C(6)-C(5)-H(5)	119.7
C(5)-C(6)-C(1)	117.91(17)
C(5)-C(6)-C(8)	120.38(17)
C(1)-C(6)-C(8)	121.70(13)
C(2)-C(7)-H(7A)	109.5
C(2)-C(7)-H(7B)	109.5
H(7A)-C(7)-H(7B)	109.5
C(2)-C(7)-H(7C)	109.5
H(7A)-C(7)-H(7C)	109.5
H(7B)-C(7)-H(7C)	109.5
C(6)-C(8)-H(8A)	109.5
C(6)-C(8)-H(8B)	109.5
H(8A)-C(8)-H(8B)	109.5
C(6)-C(8)-H(8C)	109.5
H(8A)-C(8)-H(8C)	109.5
H(8B)-C(8)-H(8C)	109.5
N(1)-C(9)-N(2)	116.31(11)
N(1)-C(9)-C(10)	123.05(12)
N(2)-C(9)-C(10)	120.36(11)
C(9)-C(10)-H(10A)	109.5
C(9)-C(10)-H(10B)	109.5
H(10A)-C(10)-H(10B)	109.5
C(9)-C(10)-H(10C)	109.5
H(10A)-C(10)-H(10C)	109.5
H(10B)-C(10)-H(10C)	109.5
C(12)-C(11)-N(2)	118.31(12)
C(12)-C(11)-C(20)	119.64(12)
N(2)-C(11)-C(20)	121.69(11)
C(11)-C(12)-C(13)	121.36(14)
C(11)-C(12)-H(12)	119.3
C(13)-C(12)-H(12)	119.3
C(14)-C(13)-C(12)	120.31(15)
C(14)-C(13)-H(13)	119.8
C(12)-C(13)-H(13)	119.8
C(13)-C(14)-C(15)	120.67(14)
C(13)-C(14)-H(14)	119.7
C(15)-C(14)-H(14)	119.7
C(14)-C(15)-C(16)	119.79(14)
C(14)-C(15)-C(20)	120.10(14)
C(16)-C(15)-C(20)	120.10(14)
C(17)-C(16)-C(15)	120.61(14)
C(17)-C(16)-H(16)	119.7
C(15)-C(16)-H(16)	119.7
C(16)-C(17)-C(18)	120.10(15)
C(16)-C(17)-H(17)	119.9
C(18)-C(17)-H(17)	119.9
C(19)-C(18)-C(17)	121.36(15)
C(19)-C(18)-H(18)	119.3

C(17)-C(18)-H(18)	119.3
C(18)-C(19)-N(3)	119.18(12)
C(18)-C(19)-C(20)	120.04(12)
N(3)-C(19)-C(20)	120.77(11)
C(19)-C(20)-C(11)	124.53(11)
C(19)-C(20)-C(15)	117.63(12)
C(11)-C(20)-C(15)	117.85(12)
N(4)-C(21)-N(3)	117.45(11)
N(4)-C(21)-C(22)	123.03(12)
N(3)-C(21)-C(22)	119.03(11)
C(21)-C(22)-H(22A)	109.5
C(21)-C(22)-H(22B)	109.5
H(22A)-C(22)-H(22B)	109.5
C(21)-C(22)-H(22C)	109.5
H(22A)-C(22)-H(22C)	109.5
H(22B)-C(22)-H(22C)	109.5
C(24)-C(23)-C(28)	121.00(14)
C(24)-C(23)-N(4)	119.86(14)
C(28)-C(23)-N(4)	118.97(14)
C(23)-C(24)-C(25)	118.11(18)
C(23)-C(24)-C(29)	120.42(14)
C(25)-C(24)-C(29)	121.47(17)
C(26)-C(25)-C(24)	121.3(2)
C(26)-C(25)-H(25)	119.3
C(24)-C(25)-H(25)	119.3
C(25)-C(26)-C(27)	120.03(17)
C(25)-C(26)-H(26)	120.0
C(27)-C(26)-H(26)	120.0
C(26)-C(27)-C(28)	121.3(2)
C(26)-C(27)-H(27)	119.4
C(28)-C(27)-H(27)	119.4
C(27)-C(28)-C(23)	118.17(19)
C(27)-C(28)-C(30)	121.82(19)
C(23)-C(28)-C(30)	120.01(16)
C(24)-C(29)-H(29A)	109.5
C(24)-C(29)-H(29B)	109.5
H(29A)-C(29)-H(29B)	109.5
C(24)-C(29)-H(29C)	109.5
H(29A)-C(29)-H(29C)	109.5
H(29B)-C(29)-H(29C)	109.5
C(28)-C(30)-H(30A)	109.5
C(28)-C(30)-H(30B)	109.5
H(30A)-C(30)-H(30B)	109.5
C(28)-C(30)-H(30C)	109.5
H(30A)-C(30)-H(30C)	109.5
H(30B)-C(30)-H(30C)	109.5
C(34)-O(1)-C(31)	103.9(3)
O(1)-C(31)-C(32)	109.8(3)
O(1)-C(31)-H(31A)	109.7
C(32)-C(31)-H(31A)	109.7
O(1)-C(31)-H(31B)	109.7
C(32)-C(31)-H(31B)	109.7
H(31A)-C(31)-H(31B)	108.2
C(33)-C(32)-C(31)	104.2(4)

C(33)-C(32)-H(32A)	110.9
C(31)-C(32)-H(32A)	110.9
C(33)-C(32)-H(32B)	110.9
C(31)-C(32)-H(32B)	110.9
H(32A)-C(32)-H(32B)	108.9
C(32)-C(33)-C(34)	103.8(4)
C(32)-C(33)-H(33A)	111.0
C(34)-C(33)-H(33A)	111.0
C(32)-C(33)-H(33B)	111.0
C(34)-C(33)-H(33B)	111.0
H(33A)-C(33)-H(33B)	109.0
O(1)-C(34)-C(33)	108.1(4)
O(1)-C(34)-H(34A)	110.1
C(33)-C(34)-H(34A)	110.1
O(1)-C(34)-H(34B)	110.1
C(33)-C(34)-H(34B)	110.1
H(34A)-C(34)-H(34B)	108.4
C(31')-O(1')-C(34')	106.1(9)
O(1')-C(31')-C(32')	106.7(8)
O(1')-C(31')-H(31C)	110.4
C(32')-C(31')-H(31C)	110.4
O(1')-C(31')-H(31D)	110.4
C(32')-C(31')-H(31D)	110.4
H(31C)-C(31')-H(31D)	108.6
C(33')-C(32')-C(31')	103.2(9)
C(33')-C(32')-H(32C)	111.1
C(31')-C(32')-H(32C)	111.1
C(33')-C(32')-H(32D)	111.1
C(31')-C(32')-H(32D)	111.1
H(32C)-C(32')-H(32D)	109.1
C(32')-C(33')-C(34')	106.5(9)
C(32')-C(33')-H(33C)	110.4
C(34')-C(33')-H(33C)	110.4
C(32')-C(33')-H(33D)	110.4
C(34')-C(33')-H(33D)	110.4
H(33C)-C(33')-H(33D)	108.6
O(1')-C(34')-C(33')	107.3(10)
O(1')-C(34')-H(34C)	110.3
C(33')-C(34')-H(34C)	110.2
O(1')-C(34')-H(34D)	110.2
C(33')-C(34')-H(34D)	110.2
H(34C)-C(34')-H(34D)	108.5
N(3)-Ge(1)-N(2)	87.48(4)
N(3)-Ge(1)-N(1)#1	95.69(4)
N(2)-Ge(1)-N(1)#1	96.79(4)
C(9)-N(1)-C(1)	121.22(11)
C(9)-N(1)-Ge(1)#1	110.44(8)
C(1)-N(1)-Ge(1)#1	125.38(8)
C(9)-N(2)-C(11)	119.28(10)
C(9)-N(2)-Ge(1)	112.38(8)
C(11)-N(2)-Ge(1)	126.95(8)
C(21)-N(3)-C(19)	120.24(10)
C(21)-N(3)-Ge(1)	113.24(8)
C(19)-N(3)-Ge(1)	126.15(8)

C(21)-N(4)-C(23)	119.68(12)

Symmetry transformations used to generate equivalent atoms:
#1 -x+1,-y+1,-z+1

Table S16. Anisotropic displacement parameters (A^2 x 10^3) for AA-R34.
The anisotropic displacement factor exponent takes the form:
-2 pi^2 [h^2 a*^2 U11 + ... + 2 h k a* b* U12]

	U11	U22	U33	U23	U13	U12
C(1)	20(1)	39(1)	19(1)	-11(1)	-4(1)	-6(1)
C(2)	29(1)	47(1)	22(1)	-3(1)	-9(1)	-11(1)
C(3)	46(1)	77(1)	20(1)	-5(1)	-9(1)	-20(1)
C(4)	48(1)	107(2)	26(1)	-32(1)	0(1)	-20(1)
C(5)	40(1)	84(2)	42(1)	-44(1)	-4(1)	-6(1)
C(6)	27(1)	49(1)	31(1)	-23(1)	-5(1)	-4(1)
C(7)	40(1)	36(1)	38(1)	4(1)	-20(1)	-7(1)
C(8)	44(1)	39(1)	48(1)	-28(1)	-12(1)	1(1)
C(9)	18(1)	22(1)	20(1)	-6(1)	-7(1)	-1(1)
C(10)	21(1)	42(1)	28(1)	-10(1)	-9(1)	-7(1)
C(11)	16(1)	21(1)	25(1)	-9(1)	-5(1)	-1(1)
C(12)	27(1)	23(1)	33(1)	-6(1)	-7(1)	3(1)
C(13)	34(1)	25(1)	52(1)	-12(1)	-11(1)	7(1)
C(14)	30(1)	29(1)	56(1)	-23(1)	-5(1)	6(1)
C(15)	23(1)	29(1)	38(1)	-20(1)	-1(1)	-2(1)
C(16)	35(1)	42(1)	43(1)	-29(1)	6(1)	-4(1)
C(17)	42(1)	46(1)	30(1)	-23(1)	8(1)	-9(1)
C(18)	34(1)	34(1)	23(1)	-12(1)	1(1)	-7(1)
C(19)	19(1)	24(1)	22(1)	-10(1)	-1(1)	-5(1)
C(20)	17(1)	22(1)	26(1)	-12(1)	-3(1)	-3(1)
C(21)	21(1)	20(1)	20(1)	-5(1)	-4(1)	-4(1)
C(22)	21(1)	28(1)	52(1)	-13(1)	-3(1)	-5(1)
C(23)	24(1)	21(1)	37(1)	-10(1)	-6(1)	-1(1)
C(24)	32(1)	23(1)	41(1)	-3(1)	-4(1)	-3(1)
C(25)	42(1)	23(1)	68(1)	-3(1)	-1(1)	-5(1)
C(26)	48(1)	26(1)	102(2)	-25(1)	-10(1)	-5(1)
C(27)	50(1)	41(1)	92(2)	-42(1)	-22(1)	4(1)
C(28)	39(1)	34(1)	54(1)	-23(1)	-17(1)	4(1)
C(29)	58(1)	43(1)	33(1)	1(1)	-8(1)	-10(1)
C(30)	85(2)	59(1)	49(1)	-29(1)	-31(1)	5(1)
O(1)	84(2)	59(1)	68(2)	-22(1)	6(1)	-3(1)
C(31)	57(2)	81(2)	99(3)	-45(2)	-21(2)	-9(2)
C(32)	65(2)	91(2)	92(2)	-37(2)	-24(2)	-8(2)
C(33)	82(3)	94(3)	124(3)	-60(2)	-23(2)	-19(3)
C(34)	67(2)	108(3)	78(2)	-51(2)	-21(2)	0(2)
O(1')	89(4)	88(4)	100(4)	-26(3)	-15(4)	-15(3)
C(31')	66(4)	89(5)	72(4)	-19(4)	-21(4)	-14(4)
C(32')	59(4)	93(4)	77(4)	-22(4)	-13(4)	-2(3)
C(33')	79(4)	96(4)	92(4)	-36(4)	-13(4)	-7(4)
C(34')	72(4)	84(5)	113(4)	-42(4)	-20(4)	-15(4)
Ge(1)	17(1)	17(1)	17(1)	-6(1)	-3(1)	-1(1)
N(1)	18(1)	22(1)	18(1)	-6(1)	-5(1)	-2(1)
N(2)	17(1)	21(1)	18(1)	-5(1)	-4(1)	0(1)
N(3)	20(1)	19(1)	20(1)	-6(1)	0(1)	-4(1)
N(4)	24(1)	20(1)	26(1)	-6(1)	-5(1)	-2(1)

Crystal data and structure refinement of compound L_2Ge

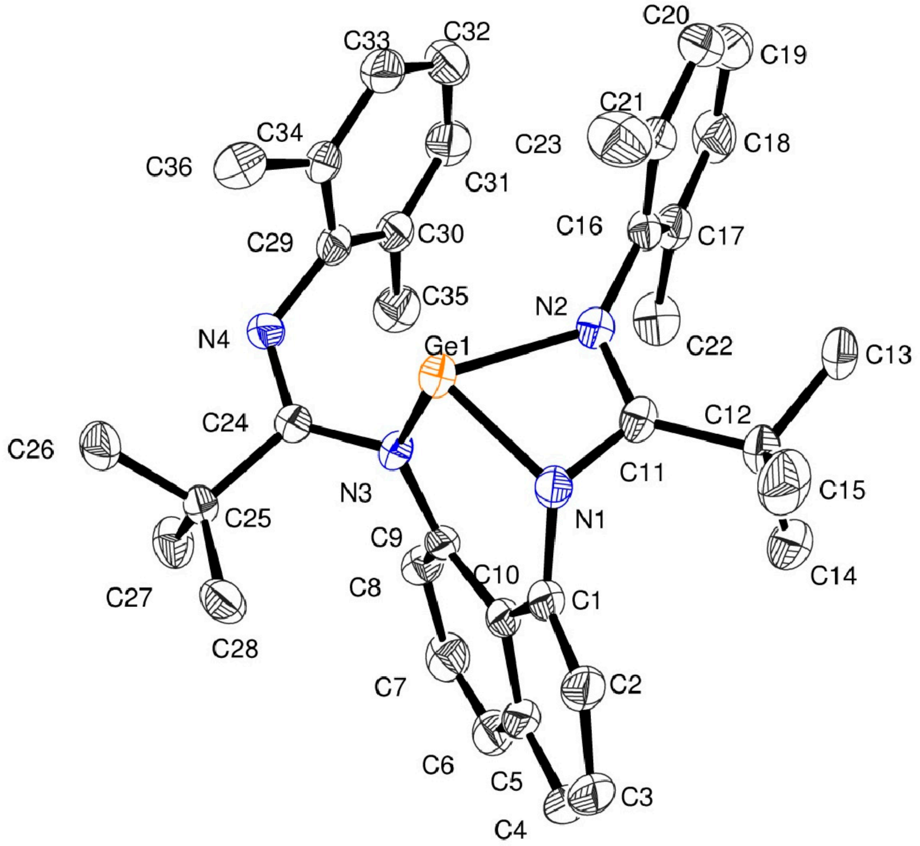

Figure S7. Asymmetric Unit

Table S17. Crystal data and structure refinement for R109.

Identification code	R109
Empirical formula	C36 H42 Ge N4
Formula weight	603.35
Temperature	193(2) K
Wavelength	0.71073 A
Crystal system, space group	Monoclinic, P 21/n
Unit cell dimensions	a = 10.879(2) A alpha = 90 deg.<br>b = 14.307(2) A beta = 97.332(6) deg.<br>c = 20.235(4) A gamma = 90 deg.
Volume	3123.7(10) A^3

Z, Calculated density	4, 1.283 Mg/m^3
Absorption coefficient	1.011 mm^-1
F(000)	1272
Crystal size	0.180 x 0.040 x 0.010 mm
Theta range for data collection	1.748 to 24.129 deg.
Limiting indices	-12<=h<=12, -14<=k<=16, -23<=l<=23
Reflections collected / unique	56977 / 4948 [R(int) = 0.1415]
Completeness to theta = 24.129	99.2 %
Refinement method	Full-matrix least-squares on F^2
Data / restraints / parameters	4948 / 0 / 380
Goodness-of-fit on F^2	1.051
Final R indices [I>2sigma(I)]	R1 = 0.0485, wR2 = 0.1040
R indices (all data)	R1 = 0.0920, wR2 = 0.1230
Largest diff. peak and hole	0.527 and -0.577 e.A^-3

Table S18. Atomic coordinates (x 10^4) and equivalent isotropic displacement parameters (A^2 x 10^3) for R109.
U(eq) is defined as one third of the trace of the orthogonalized Uij tensor.

	x	y	z	U(eq)
C(1)	8505(4)	5352(3)	1746(2)	28(1)
C(2)	9492(4)	5021(3)	1465(2)	35(1)
C(3)	9754(4)	5372(3)	855(2)	44(1)
C(4)	9044(4)	6073(3)	543(2)	44(1)
C(5)	7952(4)	6386(3)	786(2)	34(1)
C(6)	7179(4)	7072(3)	447(2)	38(1)
C(7)	6102(5)	7309(3)	666(2)	39(1)
C(8)	5718(4)	6860(3)	1216(2)	30(1)
C(9)	6409(4)	6167(3)	1556(2)	24(1)
C(10)	7623(4)	5967(3)	1382(2)	28(1)
C(11)	8690(4)	5728(3)	2941(2)	25(1)
C(12)	10019(4)	6107(3)	3089(2)	31(1)
C(13)	10277(4)	6598(3)	3770(2)	40(1)
C(14)	10290(4)	6810(3)	2554(2)	44(1)
C(15)	10897(4)	5256(3)	3106(3)	49(1)
C(16)	7704(4)	6242(3)	3925(2)	25(1)
C(17)	7389(4)	7194(3)	3923(2)	31(1)
C(18)	7273(4)	7612(3)	4527(2)	40(1)
C(19)	7448(4)	7109(3)	5113(2)	45(1)
C(20)	7739(4)	6171(3)	5105(2)	41(1)
C(21)	7852(4)	5718(3)	4507(2)	31(1)
C(22)	7182(4)	7739(3)	3283(2)	40(1)
C(23)	8152(5)	4689(3)	4498(2)	48(1)
C(24)	4593(4)	5390(2)	1884(2)	21(1)
C(25)	4093(4)	4904(3)	1212(2)	26(1)
C(26)	3245(4)	4092(3)	1381(2)	38(1)
C(27)	3282(4)	5564(3)	745(2)	38(1)
C(28)	5124(4)	4488(3)	856(2)	37(1)
C(29)	4105(4)	5776(3)	2966(2)	28(1)
C(30)	4070(4)	6748(3)	3095(2)	31(1)
C(31)	4200(4)	7034(3)	3752(2)	40(1)
C(32)	4357(4)	6419(4)	4276(2)	43(1)
C(33)	4339(4)	5459(3)	4141(2)	38(1)
C(34)	4187(4)	5125(3)	3489(2)	29(1)
C(35)	3853(4)	7445(3)	2541(2)	42(1)
C(36)	4033(4)	4092(3)	3363(2)	41(1)
N(1)	8322(3)	5144(2)	2415(2)	26(1)
N(2)	7773(3)	5801(2)	3298(2)	24(1)
N(3)	5867(3)	5612(2)	2030(2)	24(1)
N(4)	3822(3)	5429(2)	2308(2)	26(1)
Ge(1)	6714(1)	4836(1)	2710(1)	25(1)

Table S19. Bond lengths [A] and angles [deg] for R109.

C(1)-C(2)	1.362(6)
C(1)-N(1)	1.424(5)
C(1)-C(10)	1.435(6)
C(2)-C(3)	1.396(6)
C(2)-H(2)	0.9500
C(3)-C(4)	1.370(6)
C(3)-H(3)	0.9500
C(4)-C(5)	1.416(6)
C(4)-H(4)	0.9500
C(5)-C(6)	1.411(6)
C(5)-C(10)	1.431(6)
C(6)-C(7)	1.349(6)
C(6)-H(6)	0.9500
C(7)-C(8)	1.395(6)
C(7)-H(7)	0.9500
C(8)-C(9)	1.375(5)
C(8)-H(8)	0.9500
C(9)-N(3)	1.428(5)
C(9)-C(10)	1.439(5)
C(11)-N(2)	1.308(5)
C(11)-N(1)	1.374(5)
C(11)-C(12)	1.537(5)
C(12)-C(14)	1.534(6)
C(12)-C(13)	1.541(5)
C(12)-C(15)	1.545(6)
C(13)-H(13A)	0.9800
C(13)-H(13B)	0.9800
C(13)-H(13C)	0.9800
C(14)-H(14A)	0.9800
C(14)-H(14B)	0.9800
C(14)-H(14C)	0.9800
C(15)-H(15A)	0.9800
C(15)-H(15B)	0.9800
C(15)-H(15C)	0.9800
C(16)-C(21)	1.388(5)
C(16)-C(17)	1.404(5)
C(16)-N(2)	1.427(5)
C(17)-C(18)	1.381(6)
C(17)-C(22)	1.503(6)
C(18)-C(19)	1.379(6)
C(18)-H(18)	0.9500
C(19)-C(20)	1.380(6)
C(19)-H(19)	0.9500
C(20)-C(21)	1.392(6)
C(20)-H(20)	0.9500
C(21)-C(23)	1.508(6)
C(22)-H(22A)	0.9800
C(22)-H(22B)	0.9800
C(22)-H(22C)	0.9800
C(23)-H(23A)	0.9800
C(23)-H(23B)	0.9800

C(23)-H(23C)	0.9800
C(24)-N(4)	1.275(5)
C(24)-N(3)	1.417(5)
C(24)-C(25)	1.561(5)
C(25)-C(28)	1.530(6)
C(25)-C(27)	1.532(6)
C(25)-C(26)	1.548(5)
C(26)-H(26A)	0.9800
C(26)-H(26B)	0.9800
C(26)-H(26C)	0.9800
C(27)-H(27A)	0.9800
C(27)-H(27B)	0.9800
C(27)-H(27C)	0.9800
C(28)-H(28A)	0.9800
C(28)-H(28B)	0.9800
C(28)-H(28C)	0.9800
C(29)-C(34)	1.403(6)
C(29)-C(30)	1.417(6)
C(29)-N(4)	1.417(5)
C(30)-C(31)	1.380(6)
C(30)-C(35)	1.497(6)
C(31)-C(32)	1.372(6)
C(31)-H(31)	0.9500
C(32)-C(33)	1.400(6)
C(32)-H(32)	0.9500
C(33)-C(34)	1.392(6)
C(33)-H(33)	0.9500
C(34)-C(36)	1.506(6)
C(35)-H(35A)	0.9800
C(35)-H(35B)	0.9800
C(35)-H(35C)	0.9800
C(36)-H(36A)	0.9800
C(36)-H(36B)	0.9800
C(36)-H(36C)	0.9800
N(1)-Ge(1)	1.969(3)
N(2)-Ge(1)	2.073(3)
N(3)-Ge(1)	1.912(3)

C(2)-C(1)-N(1)	122.5(4)
C(2)-C(1)-C(10)	120.6(4)
N(1)-C(1)-C(10)	116.9(4)
C(1)-C(2)-C(3)	120.4(4)
C(1)-C(2)-H(2)	119.8
C(3)-C(2)-H(2)	119.8
C(4)-C(3)-C(2)	120.3(4)
C(4)-C(3)-H(3)	119.9
C(2)-C(3)-H(3)	119.9
C(3)-C(4)-C(5)	121.4(4)
C(3)-C(4)-H(4)	119.3
C(5)-C(4)-H(4)	119.3
C(6)-C(5)-C(4)	121.6(4)
C(6)-C(5)-C(10)	120.2(4)
C(4)-C(5)-C(10)	118.1(4)
C(7)-C(6)-C(5)	120.2(4)

C(7)-C(6)-H(6)	119.9
C(5)-C(6)-H(6)	119.9
C(6)-C(7)-C(8)	120.5(4)
C(6)-C(7)-H(7)	119.7
C(8)-C(7)-H(7)	119.7
C(9)-C(8)-C(7)	122.1(4)
C(9)-C(8)-H(8)	118.9
C(7)-C(8)-H(8)	118.9
C(8)-C(9)-N(3)	119.4(4)
C(8)-C(9)-C(10)	118.7(4)
N(3)-C(9)-C(10)	121.7(3)
C(5)-C(10)-C(1)	117.9(4)
C(5)-C(10)-C(9)	117.4(4)
C(1)-C(10)-C(9)	124.7(4)
N(2)-C(11)-N(1)	108.0(3)
N(2)-C(11)-C(12)	129.0(4)
N(1)-C(11)-C(12)	122.6(4)
C(14)-C(12)-C(11)	110.7(3)
C(14)-C(12)-C(13)	107.6(3)
C(11)-C(12)-C(13)	113.2(3)
C(14)-C(12)-C(15)	111.0(4)
C(11)-C(12)-C(15)	106.9(3)
C(13)-C(12)-C(15)	107.4(4)
C(12)-C(13)-H(13A)	109.5
C(12)-C(13)-H(13B)	109.5
H(13A)-C(13)-H(13B)	109.5
C(12)-C(13)-H(13C)	109.5
H(13A)-C(13)-H(13C)	109.5
H(13B)-C(13)-H(13C)	109.5
C(12)-C(14)-H(14A)	109.5
C(12)-C(14)-H(14B)	109.5
H(14A)-C(14)-H(14B)	109.5
C(12)-C(14)-H(14C)	109.5
H(14A)-C(14)-H(14C)	109.5
H(14B)-C(14)-H(14C)	109.5
C(12)-C(15)-H(15A)	109.5
C(12)-C(15)-H(15B)	109.5
H(15A)-C(15)-H(15B)	109.5
C(12)-C(15)-H(15C)	109.5
H(15A)-C(15)-H(15C)	109.5
H(15B)-C(15)-H(15C)	109.5
C(21)-C(16)-C(17)	121.9(4)
C(21)-C(16)-N(2)	120.1(3)
C(17)-C(16)-N(2)	117.8(3)
C(18)-C(17)-C(16)	117.9(4)
C(18)-C(17)-C(22)	121.1(4)
C(16)-C(17)-C(22)	121.0(4)
C(19)-C(18)-C(17)	121.1(4)
C(19)-C(18)-H(18)	119.5
C(17)-C(18)-H(18)	119.5
C(18)-C(19)-C(20)	120.3(4)
C(18)-C(19)-H(19)	119.8
C(20)-C(19)-H(19)	119.8
C(19)-C(20)-C(21)	120.6(4)

C(19)-C(20)-H(20)	119.7
C(21)-C(20)-H(20)	119.7
C(16)-C(21)-C(20)	118.2(4)
C(16)-C(21)-C(23)	121.3(4)
C(20)-C(21)-C(23)	120.5(4)
C(17)-C(22)-H(22A)	109.5
C(17)-C(22)-H(22B)	109.5
H(22A)-C(22)-H(22B)	109.5
C(17)-C(22)-H(22C)	109.5
H(22A)-C(22)-H(22C)	109.5
H(22B)-C(22)-H(22C)	109.5
C(21)-C(23)-H(23A)	109.5
C(21)-C(23)-H(23B)	109.5
H(23A)-C(23)-H(23B)	109.5
C(21)-C(23)-H(23C)	109.5
H(23A)-C(23)-H(23C)	109.5
H(23B)-C(23)-H(23C)	109.5
N(4)-C(24)-N(3)	124.0(3)
N(4)-C(24)-C(25)	114.8(3)
N(3)-C(24)-C(25)	120.3(3)
C(28)-C(25)-C(27)	110.4(3)
C(28)-C(25)-C(26)	107.8(3)
C(27)-C(25)-C(26)	106.6(3)
C(28)-C(25)-C(24)	112.8(3)
C(27)-C(25)-C(24)	111.8(3)
C(26)-C(25)-C(24)	107.0(3)
C(25)-C(26)-H(26A)	109.5
C(25)-C(26)-H(26B)	109.5
H(26A)-C(26)-H(26B)	109.5
C(25)-C(26)-H(26C)	109.5
H(26A)-C(26)-H(26C)	109.5
H(26B)-C(26)-H(26C)	109.5
C(25)-C(27)-H(27A)	109.5
C(25)-C(27)-H(27B)	109.5
H(27A)-C(27)-H(27B)	109.5
C(25)-C(27)-H(27C)	109.5
H(27A)-C(27)-H(27C)	109.5
H(27B)-C(27)-H(27C)	109.5
C(25)-C(28)-H(28A)	109.5
C(25)-C(28)-H(28B)	109.5
H(28A)-C(28)-H(28B)	109.5
C(25)-C(28)-H(28C)	109.5
H(28A)-C(28)-H(28C)	109.5
H(28B)-C(28)-H(28C)	109.5
C(34)-C(29)-C(30)	120.8(4)
C(34)-C(29)-N(4)	117.5(4)
C(30)-C(29)-N(4)	120.6(4)
C(31)-C(30)-C(29)	117.8(4)
C(31)-C(30)-C(35)	120.7(4)
C(29)-C(30)-C(35)	121.4(4)
C(32)-C(31)-C(30)	122.8(4)
C(32)-C(31)-H(31)	118.6
C(30)-C(31)-H(31)	118.6
C(31)-C(32)-C(33)	118.7(4)

C(31)-C(32)-H(32)	120.6
C(33)-C(32)-H(32)	120.6
C(34)-C(33)-C(32)	121.2(4)
C(34)-C(33)-H(33)	119.4
C(32)-C(33)-H(33)	119.4
C(33)-C(34)-C(29)	118.4(4)
C(33)-C(34)-C(36)	119.6(4)
C(29)-C(34)-C(36)	121.9(4)
C(30)-C(35)-H(35A)	109.5
C(30)-C(35)-H(35B)	109.5
H(35A)-C(35)-H(35B)	109.5
C(30)-C(35)-H(35C)	109.5
H(35A)-C(35)-H(35C)	109.5
H(35B)-C(35)-H(35C)	109.5
C(34)-C(36)-H(36A)	109.5
C(34)-C(36)-H(36B)	109.5
H(36A)-C(36)-H(36B)	109.5
C(34)-C(36)-H(36C)	109.5
H(36A)-C(36)-H(36C)	109.5
H(36B)-C(36)-H(36C)	109.5
C(11)-N(1)-C(1)	123.2(3)
C(11)-N(1)-Ge(1)	94.7(2)
C(1)-N(1)-Ge(1)	125.0(3)
C(11)-N(2)-C(16)	131.0(3)
C(11)-N(2)-Ge(1)	92.1(2)
C(16)-N(2)-Ge(1)	135.7(3)
C(24)-N(3)-C(9)	117.5(3)
C(24)-N(3)-Ge(1)	112.9(2)
C(9)-N(3)-Ge(1)	127.1(3)
C(24)-N(4)-C(29)	124.4(3)
N(3)-Ge(1)-N(1)	90.95(13)
N(3)-Ge(1)-N(2)	102.00(13)
N(1)-Ge(1)-N(2)	64.89(13)

Symmetry transformations used to generate equivalent atoms:

Table S20. Anisotropic displacement parameters (A^2 x 10^3) for R109.
The anisotropic displacement factor exponent takes the form:
-2 pi^2 [h^2 a*^2 U11 + ... + 2 h k a* b* U12]

	U11	U22	U33	U23	U13	U12
C(1)	27(3)	29(3)	28(2)	-7(2)	5(2)	-6(2)
C(2)	28(3)	37(3)	42(3)	-16(2)	13(2)	-4(2)
C(3)	31(3)	60(4)	44(3)	-21(3)	19(2)	-10(3)
C(4)	40(3)	58(3)	38(3)	-12(3)	19(2)	-18(3)
C(5)	36(3)	37(3)	32(3)	-8(2)	12(2)	-15(2)
C(6)	44(3)	37(3)	33(3)	5(2)	4(2)	-14(2)
C(7)	48(3)	27(3)	42(3)	3(2)	4(3)	-8(2)
C(8)	33(3)	22(2)	35(3)	1(2)	10(2)	-1(2)
C(9)	27(3)	23(2)	21(2)	-4(2)	6(2)	-6(2)
C(10)	28(3)	28(2)	30(2)	-12(2)	8(2)	-8(2)
C(11)	20(2)	22(2)	32(2)	5(2)	0(2)	1(2)
C(12)	21(3)	35(3)	35(3)	-2(2)	-1(2)	-3(2)
C(13)	26(3)	51(3)	40(3)	0(2)	-3(2)	-12(2)
C(14)	43(3)	45(3)	46(3)	-3(2)	11(2)	-20(2)
C(15)	26(3)	57(3)	64(3)	-4(3)	2(2)	8(2)
C(16)	23(3)	27(2)	26(2)	-5(2)	2(2)	-6(2)
C(17)	28(3)	27(2)	38(3)	-3(2)	5(2)	-4(2)
C(18)	44(3)	28(3)	48(3)	-13(2)	7(2)	-8(2)
C(19)	52(3)	45(3)	39(3)	-17(3)	14(2)	-15(2)
C(20)	46(3)	49(3)	27(3)	0(2)	4(2)	-15(2)
C(21)	30(3)	34(3)	29(3)	1(2)	4(2)	-6(2)
C(22)	40(3)	31(3)	47(3)	3(2)	-1(2)	4(2)
C(23)	64(4)	39(3)	40(3)	11(2)	2(3)	4(3)
C(24)	26(2)	14(2)	24(2)	2(2)	3(2)	1(2)
C(25)	26(2)	29(2)	23(2)	-2(2)	0(2)	-6(2)
C(26)	46(3)	31(3)	36(3)	-4(2)	3(2)	-12(2)
C(27)	35(3)	37(3)	37(3)	4(2)	-9(2)	-3(2)
C(28)	35(3)	44(3)	30(3)	-12(2)	2(2)	1(2)
C(29)	17(2)	35(3)	31(2)	-3(2)	4(2)	-3(2)
C(30)	19(3)	40(3)	33(3)	-4(2)	5(2)	0(2)
C(31)	35(3)	42(3)	45(3)	-13(2)	14(2)	0(2)
C(32)	35(3)	61(4)	34(3)	-11(3)	4(2)	-5(2)
C(33)	29(3)	53(3)	33(3)	4(2)	2(2)	-4(2)
C(34)	18(2)	38(3)	31(2)	-1(2)	3(2)	-3(2)
C(35)	40(3)	38(3)	49(3)	-3(2)	11(2)	1(2)
C(36)	43(3)	43(3)	39(3)	5(2)	11(2)	-4(2)
N(1)	21(2)	26(2)	29(2)	-5(2)	3(2)	-2(2)
N(2)	22(2)	22(2)	27(2)	2(2)	2(2)	1(2)
N(3)	18(2)	25(2)	28(2)	0(2)	4(2)	-2(2)
N(4)	23(2)	31(2)	23(2)	-2(2)	4(2)	-1(2)
Ge(1)	22(1)	23(1)	31(1)	0(1)	2(1)	-2(1)

Crystal data and structure refinement of compound 1a

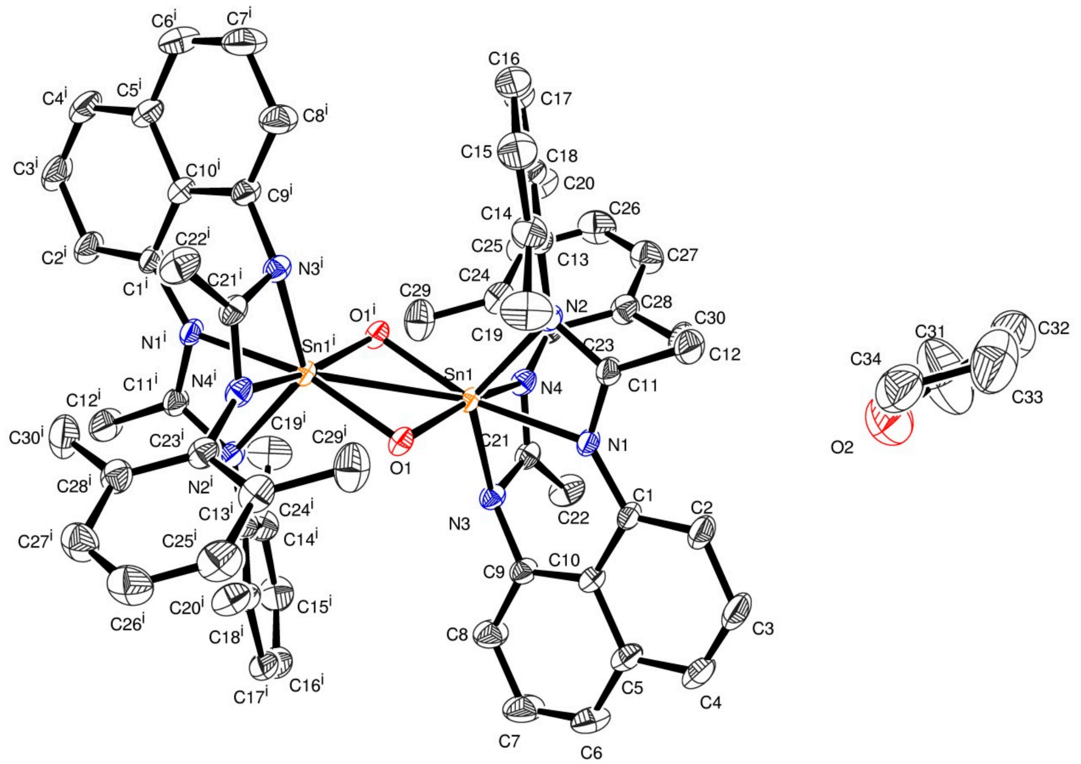

Figure S8. Asymmetric Unit

Table S21. Crystal data and structure refinement for R126.

Identification code	R126
Empirical formula	C60 H60 N8 O2 Sn2, 2(C4 H8 O)
Formula weight	1306.79
Temperature	193(2) K
Wavelength	0.71073 A
Crystal system, space group	Triclinic, P -1
Unit cell dimensions	a = 11.0951(5) A alpha = 88.337(2) deg.<br>b = 11.8481(6) A beta = 88.326(2) deg.<br>c = 12.8106(6) A gamma = 65.647(2) deg.
Volume	1533.27(13) A^3
Z, Calculated density	1, 1.415 Mg/m^3
Absorption coefficient	0.870 mm^-1

F(000)	672
Crystal size	0.120 x 0.120 x 0.080 mm
Theta range for data collection	3.182 to 36.438 deg.
Limiting indices	-18<=h<=18, -19<=k<=19, -21<=l<=21
Reflections collected / unique	122943 / 14935 [R(int) = 0.0430]
Completeness to theta = 25.242	99.7 %
Refinement method	Full-matrix least-squares on F^2
Data / restraints / parameters	14935 / 0 / 376
Goodness-of-fit on F^2	1.061
Final R indices [I>2sigma(I)]	R1 = 0.0272, wR2 = 0.0652
R indices (all data)	R1 = 0.0361, wR2 = 0.0703
Largest diff. peak and hole	1.016 and -0.944 e.A^-3

Table S22. Atomic coordinates (x 10^4) and equivalent isotropic displacement parameters (A^2 x 10^3) for R126.
U(eq) is defined as one third of the trace of the orthogonalized Uij tensor.

	x	y	z	U(eq)
C(1)	1225(1)	5332(1)	8431(1)	22(1)
C(2)	581(1)	4715(2)	7943(1)	33(1)
C(3)	-758(2)	4974(2)	8162(2)	38(1)
C(4)	-1470(1)	5842(2)	8865(1)	34(1)
C(5)	-884(1)	6531(1)	9367(1)	28(1)
C(6)	-1641(2)	7429(2)	10105(2)	38(1)
C(7)	-1111(2)	8092(2)	10630(2)	43(1)
C(8)	205(2)	7926(2)	10406(1)	34(1)
C(9)	961(1)	7097(1)	9665(1)	23(1)
C(10)	470(1)	6319(1)	9142(1)	22(1)
C(11)	3519(1)	4161(1)	7727(1)	24(1)
C(12)	3330(2)	3412(2)	6877(2)	46(1)
C(13)	5954(1)	3202(1)	7614(1)	24(1)
C(14)	6330(2)	1970(1)	7966(1)	31(1)
C(15)	7575(2)	1088(2)	7666(1)	37(1)
C(16)	8441(2)	1419(2)	7069(1)	36(1)
C(17)	8087(1)	2646(2)	6775(1)	31(1)
C(18)	6842(1)	3554(1)	7034(1)	25(1)
C(19)	5448(2)	1609(2)	8691(2)	47(1)
C(20)	6454(2)	4881(2)	6702(2)	35(1)
C(21)	2328(1)	7708(1)	8523(1)	22(1)
C(22)	1220(2)	8870(1)	8143(1)	34(1)
C(23)	3877(1)	7715(1)	7170(1)	23(1)
C(24)	4677(2)	8352(1)	7317(1)	29(1)
C(25)	5098(2)	8821(2)	6435(2)	39(1)
C(26)	4741(2)	8659(2)	5440(2)	41(1)
C(27)	3956(2)	8027(2)	5305(1)	36(1)
C(28)	3512(1)	7541(1)	6167(1)	28(1)
C(29)	5065(2)	8512(2)	8401(2)	41(1)
C(30)	2698(2)	6815(2)	6002(1)	41(1)
C(31)	853(6)	3423(4)	4475(3)	112(2)
C(32)	1106(3)	2388(3)	3786(2)	74(1)
C(33)	1235(3)	1337(3)	4558(3)	86(1)
C(34)	1539(3)	1780(3)	5565(2)	65(1)
N(1)	2600(1)	4968(1)	8340(1)	21(1)
N(2)	4712(1)	4140(1)	7914(1)	23(1)
N(3)	2221(1)	7087(1)	9390(1)	20(1)
N(4)	3484(1)	7180(1)	8050(1)	22(1)
O(1)	4297(1)	4403(1)	10413(1)	20(1)
O(2)	883(2)	3071(2)	5526(2)	72(1)
Sn(1)	4040(1)	5497(1)	9137(1)	16(1)

Table S23. Bond lengths [A] and angles [deg] for R126

C(1)-C(2)	1.3849(18)
C(1)-N(1)	1.4063(16)
C(1)-C(10)	1.4515(18)
C(2)-C(3)	1.409(2)
C(2)-H(2)	0.9500
C(3)-C(4)	1.354(3)
C(3)-H(3)	0.9500
C(4)-C(5)	1.414(2)
C(4)-H(4)	0.9500
C(5)-C(6)	1.417(2)
C(5)-C(10)	1.4388(18)
C(6)-C(7)	1.361(3)
C(6)-H(6)	0.9500
C(7)-C(8)	1.412(2)
C(7)-H(7)	0.9500
C(8)-C(9)	1.3772(19)
C(8)-H(8)	0.9500
C(9)-N(3)	1.4267(16)
C(9)-C(10)	1.4371(18)
C(11)-N(1)	1.3285(16)
C(11)-N(2)	1.3415(16)
C(11)-C(12)	1.497(2)
C(11)-Sn(1)	2.6612(12)
C(12)-H(12A)	0.9800
C(12)-H(12B)	0.9800
C(12)-H(12C)	0.9800
C(13)-C(18)	1.405(2)
C(13)-C(14)	1.407(2)
C(13)-N(2)	1.4186(16)
C(14)-C(15)	1.397(2)
C(14)-C(19)	1.508(3)
C(15)-C(16)	1.383(3)
C(15)-H(15)	0.9500
C(16)-C(17)	1.383(2)
C(16)-H(16)	0.9500
C(17)-C(18)	1.3949(19)
C(17)-H(17)	0.9500
C(18)-C(20)	1.501(2)
C(19)-H(19A)	0.9800
C(19)-H(19B)	0.9800
C(19)-H(19C)	0.9800
C(20)-H(20A)	0.9800
C(20)-H(20B)	0.9800
C(20)-H(20C)	0.9800
C(21)-N(4)	1.3113(17)
C(21)-N(3)	1.3439(17)
C(21)-C(22)	1.4978(18)
C(21)-Sn(1)	2.6374(12)
C(22)-H(22A)	0.9800
C(22)-H(22B)	0.9800
C(22)-H(22C)	0.9800

C(23)-C(24)	1.401(2)
C(23)-C(28)	1.404(2)
C(23)-N(4)	1.4213(17)
C(24)-C(25)	1.397(2)
C(24)-C(29)	1.505(2)
C(25)-C(26)	1.387(3)
C(25)-H(25)	0.9500
C(26)-C(27)	1.380(3)
C(26)-H(26)	0.9500
C(27)-C(28)	1.400(2)
C(27)-H(27)	0.9500
C(28)-C(30)	1.504(2)
C(29)-H(29A)	0.9800
C(29)-H(29B)	0.9800
C(29)-H(29C)	0.9800
C(30)-H(30A)	0.9800
C(30)-H(30B)	0.9800
C(30)-H(30C)	0.9800
C(31)-O(2)	1.395(4)
C(31)-C(32)	1.458(5)
C(31)-H(31A)	0.9900
C(31)-H(31B)	0.9900
C(32)-C(33)	1.530(5)
C(32)-H(32A)	0.9900
C(32)-H(32B)	0.9900
C(33)-C(34)	1.504(4)
C(33)-H(33A)	0.9900
C(33)-H(33B)	0.9900
C(34)-O(2)	1.397(3)
C(34)-H(34A)	0.9900
C(34)-H(34B)	0.9900
N(1)-Sn(1)	2.2236(10)
N(2)-Sn(1)	2.1644(11)
N(3)-Sn(1)	2.1423(10)
N(4)-Sn(1)	2.2742(10)
O(1)-Sn(1)#1	1.9996(8)
O(1)-Sn(1)	2.0079(9)
Sn(1)-Sn(1)#1	2.97787(19)
C(2)-C(1)-N(1)	122.61(12)
C(2)-C(1)-C(10)	118.62(12)
N(1)-C(1)-C(10)	118.51(10)
C(1)-C(2)-C(3)	121.84(15)
C(1)-C(2)-H(2)	119.1
C(3)-C(2)-H(2)	119.1
C(4)-C(3)-C(2)	120.95(14)
C(4)-C(3)-H(3)	119.5
C(2)-C(3)-H(3)	119.5
C(3)-C(4)-C(5)	120.05(13)
C(3)-C(4)-H(4)	120.0
C(5)-C(4)-H(4)	120.0
C(4)-C(5)-C(6)	119.19(13)
C(4)-C(5)-C(10)	120.64(14)
C(6)-C(5)-C(10)	120.16(14)

C(7)-C(6)-C(5)	121.41(14)
C(7)-C(6)-H(6)	119.3
C(5)-C(6)-H(6)	119.3
C(6)-C(7)-C(8)	119.61(15)
C(6)-C(7)-H(7)	120.2
C(8)-C(7)-H(7)	120.2
C(9)-C(8)-C(7)	120.89(15)
C(9)-C(8)-H(8)	119.6
C(7)-C(8)-H(8)	119.6
C(8)-C(9)-N(3)	117.66(12)
C(8)-C(9)-C(10)	121.30(12)
N(3)-C(9)-C(10)	120.95(11)
C(9)-C(10)-C(5)	116.42(12)
C(9)-C(10)-C(1)	125.86(11)
C(5)-C(10)-C(1)	117.71(12)
N(1)-C(11)-N(2)	110.50(11)
N(1)-C(11)-C(12)	127.95(12)
N(2)-C(11)-C(12)	121.42(12)
N(1)-C(11)-Sn(1)	56.50(6)
N(2)-C(11)-Sn(1)	54.00(6)
C(12)-C(11)-Sn(1)	174.81(11)
C(11)-C(12)-H(12A)	109.5
C(11)-C(12)-H(12B)	109.5
H(12A)-C(12)-H(12B)	109.5
C(11)-C(12)-H(12C)	109.5
H(12A)-C(12)-H(12C)	109.5
H(12B)-C(12)-H(12C)	109.5
C(18)-C(13)-C(14)	120.86(12)
C(18)-C(13)-N(2)	118.26(12)
C(14)-C(13)-N(2)	120.64(13)
C(15)-C(14)-C(13)	118.33(15)
C(15)-C(14)-C(19)	120.20(15)
C(13)-C(14)-C(19)	121.40(14)
C(16)-C(15)-C(14)	121.13(15)
C(16)-C(15)-H(15)	119.4
C(14)-C(15)-H(15)	119.4
C(15)-C(16)-C(17)	119.94(14)
C(15)-C(16)-H(16)	120.0
C(17)-C(16)-H(16)	120.0
C(16)-C(17)-C(18)	120.96(15)
C(16)-C(17)-H(17)	119.5
C(18)-C(17)-H(17)	119.5
C(17)-C(18)-C(13)	118.63(13)
C(17)-C(18)-C(20)	120.86(14)
C(13)-C(18)-C(20)	120.51(12)
C(14)-C(19)-H(19A)	109.5
C(14)-C(19)-H(19B)	109.5
H(19A)-C(19)-H(19B)	109.5
C(14)-C(19)-H(19C)	109.5
H(19A)-C(19)-H(19C)	109.5
H(19B)-C(19)-H(19C)	109.5
C(18)-C(20)-H(20A)	109.5
C(18)-C(20)-H(20B)	109.5
H(20A)-C(20)-H(20B)	109.5

C(18)-C(20)-H(20C)	109.5
H(20A)-C(20)-H(20C)	109.5
H(20B)-C(20)-H(20C)	109.5
N(4)-C(21)-N(3)	112.95(11)
N(4)-C(21)-C(22)	124.42(13)
N(3)-C(21)-C(22)	122.62(12)
N(4)-C(21)-Sn(1)	59.57(6)
N(3)-C(21)-Sn(1)	53.94(6)
C(22)-C(21)-Sn(1)	171.30(10)
C(21)-C(22)-H(22A)	109.5
C(21)-C(22)-H(22B)	109.5
H(22A)-C(22)-H(22B)	109.5
C(21)-C(22)-H(22C)	109.5
H(22A)-C(22)-H(22C)	109.5
H(22B)-C(22)-H(22C)	109.5
C(24)-C(23)-C(28)	121.39(13)
C(24)-C(23)-N(4)	119.17(12)
C(28)-C(23)-N(4)	119.36(12)
C(25)-C(24)-C(23)	118.07(15)
C(25)-C(24)-C(29)	121.70(15)
C(23)-C(24)-C(29)	120.23(14)
C(26)-C(25)-C(24)	121.11(16)
C(26)-C(25)-H(25)	119.4
C(24)-C(25)-H(25)	119.4
C(27)-C(26)-C(25)	120.25(15)
C(27)-C(26)-H(26)	119.9
C(25)-C(26)-H(26)	119.9
C(26)-C(27)-C(28)	120.55(16)
C(26)-C(27)-H(27)	119.7
C(28)-C(27)-H(27)	119.7
C(27)-C(28)-C(23)	118.63(14)
C(27)-C(28)-C(30)	119.79(14)
C(23)-C(28)-C(30)	121.55(13)
C(24)-C(29)-H(29A)	109.5
C(24)-C(29)-H(29B)	109.5
H(29A)-C(29)-H(29B)	109.5
C(24)-C(29)-H(29C)	109.5
H(29A)-C(29)-H(29C)	109.5
H(29B)-C(29)-H(29C)	109.5
C(28)-C(30)-H(30A)	109.5
C(28)-C(30)-H(30B)	109.5
H(30A)-C(30)-H(30B)	109.5
C(28)-C(30)-H(30C)	109.5
H(30A)-C(30)-H(30C)	109.5
H(30B)-C(30)-H(30C)	109.5
O(2)-C(31)-C(32)	112.0(3)
O(2)-C(31)-H(31A)	109.2
C(32)-C(31)-H(31A)	109.2
O(2)-C(31)-H(31B)	109.2
C(32)-C(31)-H(31B)	109.2
H(31A)-C(31)-H(31B)	107.9
C(31)-C(32)-C(33)	102.4(3)
C(31)-C(32)-H(32A)	111.3
C(33)-C(32)-H(32A)	111.3

C(31)-C(32)-H(32B)	111.3
C(33)-C(32)-H(32B)	111.3
H(32A)-C(32)-H(32B)	109.2
C(34)-C(33)-C(32)	102.9(2)
C(34)-C(33)-H(33A)	111.2
C(32)-C(33)-H(33A)	111.2
C(34)-C(33)-H(33B)	111.2
C(32)-C(33)-H(33B)	111.2
H(33A)-C(33)-H(33B)	109.1
O(2)-C(34)-C(33)	106.0(2)
O(2)-C(34)-H(34A)	110.5
C(33)-C(34)-H(34A)	110.5
O(2)-C(34)-H(34B)	110.5
C(33)-C(34)-H(34B)	110.5
H(34A)-C(34)-H(34B)	108.7
C(11)-N(1)-C(1)	132.08(11)
C(11)-N(1)-Sn(1)	93.61(7)
C(1)-N(1)-Sn(1)	134.30(8)
C(11)-N(2)-C(13)	126.28(11)
C(11)-N(2)-Sn(1)	95.91(8)
C(13)-N(2)-Sn(1)	134.66(8)
C(21)-N(3)-C(9)	118.61(10)
C(21)-N(3)-Sn(1)	95.58(8)
C(9)-N(3)-Sn(1)	127.19(8)
C(21)-N(4)-C(23)	123.45(11)
C(21)-N(4)-Sn(1)	90.62(8)
C(23)-N(4)-Sn(1)	145.85(9)
Sn(1)#1-O(1)-Sn(1)	95.98(4)
C(31)-O(2)-C(34)	106.4(3)
O(1)#1-Sn(1)-O(1)	84.01(4)
O(1)#1-Sn(1)-N(3)	117.25(4)
O(1)-Sn(1)-N(3)	106.26(4)
O(1)#1-Sn(1)-N(2)	103.51(4)
O(1)-Sn(1)-N(2)	101.24(4)
N(3)-Sn(1)-N(2)	132.38(4)
O(1)#1-Sn(1)-N(1)	163.46(4)
O(1)-Sn(1)-N(1)	97.84(4)
N(3)-Sn(1)-N(1)	78.15(4)
N(2)-Sn(1)-N(1)	59.97(4)
O(1)#1-Sn(1)-N(4)	93.43(4)
O(1)-Sn(1)-N(4)	162.99(4)
N(3)-Sn(1)-N(4)	60.07(4)
N(2)-Sn(1)-N(4)	95.71(4)
N(1)-Sn(1)-N(4)	89.39(4)
O(1)#1-Sn(1)-C(21)	109.70(4)
O(1)-Sn(1)-C(21)	136.53(4)
N(3)-Sn(1)-C(21)	30.47(4)
N(2)-Sn(1)-C(21)	114.25(4)
N(1)-Sn(1)-C(21)	80.27(4)
N(4)-Sn(1)-C(21)	29.81(4)
O(1)#1-Sn(1)-C(11)	133.59(4)
O(1)-Sn(1)-C(11)	100.72(4)
N(3)-Sn(1)-C(11)	105.72(4)
N(2)-Sn(1)-C(11)	30.09(4)

N(1)-Sn(1)-C(11)	29.88(4)
N(4)-Sn(1)-C(11)	93.23(4)
C(21)-Sn(1)-C(11)	98.20(4)
O(1)#1-Sn(1)-Sn(1)#1	42.11(3)
O(1)-Sn(1)-Sn(1)#1	41.90(2)
N(3)-Sn(1)-Sn(1)#1	119.75(3)
N(2)-Sn(1)-Sn(1)#1	106.75(3)
N(1)-Sn(1)-Sn(1)#1	137.37(3)
N(4)-Sn(1)-Sn(1)#1	133.24(3)
C(21)-Sn(1)-Sn(1)#1	135.70(3)
C(11)-Sn(1)-Sn(1)#1	126.04(3)

Symmetry transformations used to generate equivalent atoms:
#1 -x+1,-y+1,-z+2

Table S24. Anisotropic displacement parameters (A^2 x 10^3) for R126.
The anisotropic displacement factor exponent takes the form:
-2 pi^2 [h^2 a*^2 U11 + ... + 2 h k a* b* U12]

	U11	U22	U33	U23	U13	U12
C(1)	16(1)	24(1)	25(1)	-1(1)	-5(1)	-9(1)
C(2)	23(1)	40(1)	39(1)	-10(1)	-5(1)	-16(1)
C(3)	26(1)	48(1)	46(1)	-4(1)	-9(1)	-22(1)
C(4)	19(1)	43(1)	43(1)	5(1)	-6(1)	-16(1)
C(5)	16(1)	31(1)	35(1)	3(1)	-2(1)	-9(1)
C(6)	20(1)	42(1)	48(1)	-4(1)	8(1)	-10(1)
C(7)	28(1)	42(1)	55(1)	-17(1)	15(1)	-10(1)
C(8)	26(1)	31(1)	42(1)	-13(1)	7(1)	-10(1)
C(9)	18(1)	21(1)	28(1)	-2(1)	0(1)	-6(1)
C(10)	15(1)	23(1)	26(1)	1(1)	-2(1)	-7(1)
C(11)	20(1)	26(1)	27(1)	-8(1)	-2(1)	-9(1)
C(12)	30(1)	60(1)	51(1)	-36(1)	2(1)	-19(1)
C(13)	19(1)	23(1)	26(1)	-7(1)	-2(1)	-5(1)
C(14)	31(1)	24(1)	34(1)	-5(1)	0(1)	-8(1)
C(15)	35(1)	24(1)	42(1)	-4(1)	-3(1)	-1(1)
C(16)	24(1)	33(1)	37(1)	-7(1)	-2(1)	3(1)
C(17)	19(1)	36(1)	30(1)	-5(1)	-2(1)	-5(1)
C(18)	18(1)	26(1)	27(1)	-4(1)	-3(1)	-5(1)
C(19)	48(1)	32(1)	58(1)	0(1)	12(1)	-15(1)
C(20)	25(1)	31(1)	46(1)	2(1)	0(1)	-10(1)
C(21)	19(1)	16(1)	28(1)	0(1)	-5(1)	-6(1)
C(22)	25(1)	22(1)	46(1)	9(1)	-5(1)	0(1)
C(23)	20(1)	21(1)	27(1)	6(1)	-3(1)	-7(1)
C(24)	29(1)	24(1)	37(1)	6(1)	-5(1)	-13(1)
C(25)	40(1)	35(1)	48(1)	11(1)	-1(1)	-22(1)
C(26)	47(1)	38(1)	39(1)	11(1)	5(1)	-20(1)
C(27)	41(1)	36(1)	29(1)	8(1)	-1(1)	-14(1)
C(28)	26(1)	29(1)	28(1)	7(1)	-4(1)	-10(1)
C(29)	53(1)	38(1)	43(1)	6(1)	-14(1)	-29(1)
C(30)	45(1)	54(1)	33(1)	8(1)	-13(1)	-30(1)
C(31)	195(5)	94(3)	72(2)	-11(2)	-7(3)	-84(3)
C(32)	53(1)	90(2)	66(2)	-21(2)	-8(1)	-14(1)
C(33)	64(2)	57(2)	122(3)	-37(2)	-28(2)	-6(1)
C(34)	43(1)	68(2)	77(2)	-8(1)	-9(1)	-15(1)
N(1)	16(1)	22(1)	24(1)	-4(1)	-4(1)	-7(1)
N(2)	17(1)	24(1)	26(1)	-8(1)	-1(1)	-6(1)
N(3)	17(1)	18(1)	25(1)	-2(1)	-1(1)	-6(1)
N(4)	18(1)	20(1)	25(1)	5(1)	-5(1)	-6(1)
O(1)	17(1)	22(1)	24(1)	3(1)	-5(1)	-10(1)
O(2)	74(1)	70(1)	71(1)	-24(1)	2(1)	-27(1)
Sn(1)	13(1)	16(1)	20(1)	0(1)	-4(1)	-5(1)

Crystal data and structure refinement of compound 1b

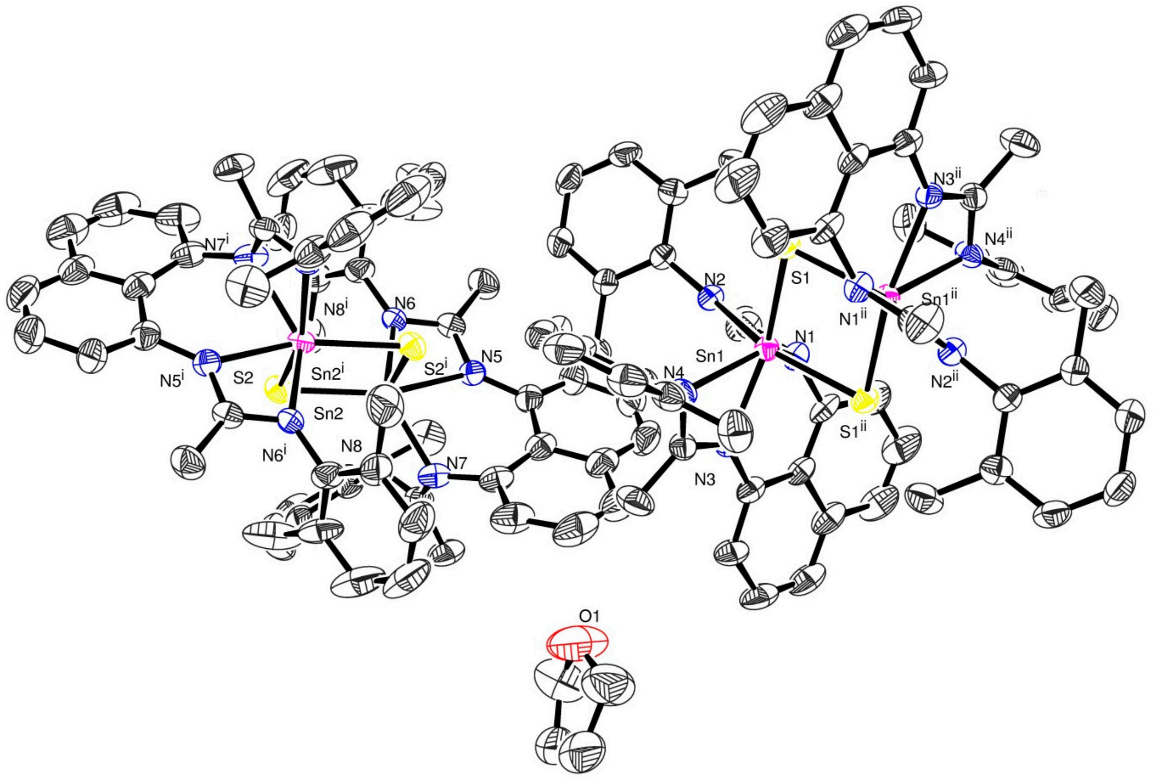

Figure S9. Asymmetric unit (Symmetry code : i = -x, -y, 1-z ; ii =-x, 1-y, 2-z)

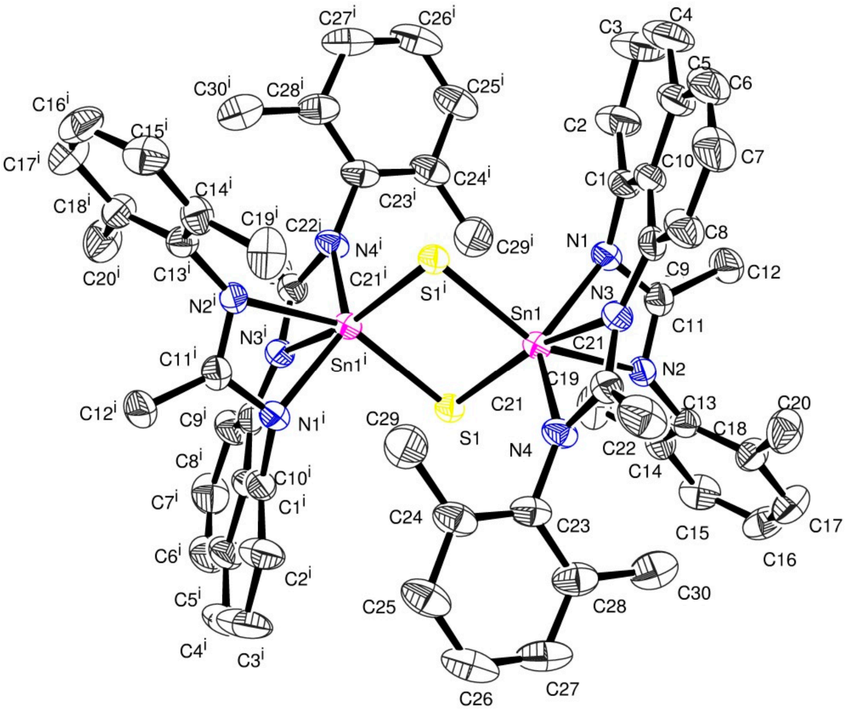

Figure S10. Compound (Symmetry code : i= -x, 1-y, 2-z)

Table S25. Crystal data and structure refinement for R200.

Identification code	R200
Empirical formula	C60 H60 N8 S2 Sn2, C4 H8 O
Formula weight	1266.80
Temperature	193(2) K
Wavelength	0.71073 A
Crystal system, space group	Triclinic, P -1
Unit cell dimensions	a = 12.269(5) A alpha = 95.205(15) deg.<br>b = 12.952(7) A beta = 94.892(14) deg.<br>c = 19.923(6) A gamma = 110.382(19) deg.
Volume	2932(2) A^3
Z, Calculated density	2, 1.435 Mg/m^3

Absorption coefficient	0.972 mm^-1
F(000)	1296
Crystal size	0.100 x 0.080 x 0.060 mm
Theta range for data collection	2.837 to 28.722 deg.
Limiting indices	-16<=h<=16, -17<=k<=17, -26<=l<=26
Reflections collected / unique	75605 / 15114 [R(int) = 0.1174]
Completeness to theta = 25.242	99.9 %
Refinement method	Full-matrix least-squares on F^2
Data / restraints / parameters	15114 / 0 / 706
Goodness-of-fit on F^2	1.008
Final R indices [I>2sigma(I)]	R1 = 0.0515, wR2 = 0.0893
R indices (all data)	R1 = 0.1157, wR2 = 0.1092
Largest diff. peak and hole	0.687 and -0.593 e.A^-3

Table S26. Atomic coordinates (x 10^4) and equivalent isotropic displacement parameters (A^2 x 10^3) for R200.
U(eq) is defined as one third of the trace of the orthogonalized Uij tensor.

	x	y	z	U(eq)
C(1)	2592(4)	6749(4)	8920(2)	37(1)
C(2)	2884(5)	7875(4)	8972(3)	52(1)
C(3)	4065(5)	8594(5)	9071(3)	71(2)
C(4)	4919(5)	8150(5)	9100(3)	65(2)
C(5)	4674(4)	6991(5)	9059(3)	48(1)
C(6)	5589(4)	6578(5)	9095(3)	56(2)
C(7)	5369(4)	5471(5)	9085(3)	55(2)
C(8)	4211(4)	4720(4)	9051(3)	44(1)
C(9)	3262(4)	5068(4)	9008(2)	34(1)
C(10)	3473(4)	6245(4)	8993(2)	34(1)
C(11)	837(4)	5695(4)	8142(2)	33(1)
C(12)	1231(4)	6306(4)	7553(2)	47(1)
C(13)	-836(4)	4290(4)	7492(2)	31(1)
C(14)	-1886(4)	4487(4)	7392(2)	36(1)
C(15)	-2644(4)	3987(4)	6802(3)	45(1)
C(16)	-2363(5)	3323(4)	6313(3)	49(1)
C(17)	-1319(5)	3151(4)	6410(3)	48(1)
C(18)	-544(4)	3612(4)	7000(2)	36(1)
C(19)	-2172(5)	5265(5)	7905(3)	50(1)
C(20)	609(4)	3436(5)	7086(3)	53(1)
C(21)	1632(4)	3272(4)	9070(2)	33(1)
C(22)	2207(4)	2417(4)	9001(3)	54(2)
C(23)	-212(4)	1913(4)	9313(3)	35(1)
C(24)	-244(4)	1663(4)	9986(3)	41(1)
C(25)	-1004(4)	626(4)	10091(3)	50(1)
C(26)	-1696(4)	-134(4)	9560(4)	56(2)
C(27)	-1649(4)	105(4)	8906(3)	54(2)
C(28)	-914(4)	1137(4)	8765(3)	44(1)
C(29)	512(5)	2461(4)	10574(3)	56(2)
C(30)	-856(5)	1396(5)	8045(3)	59(2)
C(31)	2199(4)	3911(4)	5415(3)	41(1)
C(32)	2329(5)	4999(4)	5368(3)	54(1)
C(33)	2942(5)	5857(5)	5898(4)	69(2)
C(34)	3394(5)	5607(5)	6482(4)	66(2)
C(35)	3345(4)	4525(5)	6546(3)	54(2)
C(36)	3794(5)	4291(7)	7156(3)	72(2)
C(37)	3753(5)	3248(7)	7234(3)	77(2)
C(38)	3336(5)	2384(6)	6677(3)	66(2)
C(39)	2929(4)	2608(5)	6054(3)	43(1)
C(40)	2817(4)	3657(4)	5991(3)	43(1)
C(41)	648(4)	2940(4)	4426(2)	35(1)
C(42)	309(5)	3879(4)	4194(3)	54(1)
C(43)	-829(4)	1495(4)	3612(3)	44(1)
C(44)	-1937(5)	1388(5)	3776(3)	59(2)

C(45)	-2875(6)	970(6)	3285(4)	88(2)
C(46)	-2746(7)	594(6)	2632(4)	90(3)
C(47)	-1646(6)	666(5)	2467(3)	72(2)
C(48)	-672(5)	1126(4)	2961(3)	52(1)
C(49)	-2124(6)	1727(7)	4493(4)	92(3)
C(50)	537(6)	1232(6)	2779(3)	71(2)
C(51)	3373(4)	1988(4)	4987(3)	38(1)
C(52)	4675(4)	2597(5)	5127(3)	57(2)
C(53)	3345(4)	1555(4)	3769(3)	41(1)
C(54)	3623(4)	610(5)	3577(3)	56(2)
C(55)	4058(5)	543(6)	2963(4)	80(2)
C(56)	4220(5)	1373(8)	2554(4)	90(3)
C(57)	3967(5)	2293(6)	2752(3)	71(2)
C(58)	3542(4)	2424(5)	3366(3)	51(1)
C(59)	3487(5)	-271(5)	4043(4)	88(3)
C(60)	3339(5)	3469(5)	3601(3)	65(2)
C(61)	5681(8)	2215(8)	8124(5)	115(3)
C(62)	6549(9)	1870(10)	8452(5)	145(5)
C(63)	6224(7)	1656(6)	9147(6)	108(3)
C(64)	5219(8)	2057(8)	9169(4)	116(3)
N(1)	1386(3)	6079(3)	8778(2)	32(1)
N(2)	-56(3)	4756(3)	8109(2)	29(1)
N(3)	2106(3)	4357(3)	9033(2)	30(1)
N(4)	523(3)	2992(3)	9188(2)	32(1)
N(5)	1442(3)	3001(3)	4947(2)	35(1)
N(6)	167(3)	1905(3)	4119(2)	33(1)
N(7)	2683(3)	1793(3)	5474(2)	39(1)
N(8)	2808(3)	1605(3)	4370(2)	37(1)
S(1)	-1432(1)	4460(1)	9574(1)	30(1)
S(2)	268(1)	-759(1)	4290(1)	36(1)
Sn(1)	496(1)	4687(1)	9270(1)	26(1)
Sn(2)	1041(1)	1155(1)	4825(1)	30(1)
O(1)	4754(5)	2050(6)	8513(3)	126(2)

Table S27. Bond lengths [A] and angles [deg] for R200.

C(1)-C(2)	1.367(7)
C(1)-N(1)	1.419(5)
C(1)-C(10)	1.447(7)
C(2)-C(3)	1.409(7)
C(2)-H(2)	0.9500
C(3)-C(4)	1.359(9)
C(3)-H(3)	0.9500
C(4)-C(5)	1.417(8)
C(4)-H(4)	0.9500
C(5)-C(6)	1.401(8)
C(5)-C(10)	1.441(6)
C(6)-C(7)	1.361(8)
C(6)-H(6)	0.9500
C(7)-C(8)	1.406(7)
C(7)-H(7)	0.9500
C(8)-C(9)	1.387(6)
C(8)-H(8)	0.9500
C(9)-N(3)	1.404(5)
C(9)-C(10)	1.459(7)
C(11)-N(2)	1.315(5)
C(11)-N(1)	1.345(6)
C(11)-C(12)	1.496(6)
C(12)-H(12A)	0.9800
C(12)-H(12B)	0.9800
C(12)-H(12C)	0.9800
C(13)-C(14)	1.399(6)
C(13)-C(18)	1.408(6)
C(13)-N(2)	1.433(5)
C(14)-C(15)	1.385(6)
C(14)-C(19)	1.519(6)
C(15)-C(16)	1.385(7)
C(15)-H(15)	0.9500
C(16)-C(17)	1.376(7)
C(16)-H(16)	0.9500
C(17)-C(18)	1.390(6)
C(17)-H(17)	0.9500
C(18)-C(20)	1.507(6)
C(19)-H(19A)	0.9800
C(19)-H(19B)	0.9800
C(19)-H(19C)	0.9800
C(20)-H(20A)	0.9800
C(20)-H(20B)	0.9800
C(20)-H(20C)	0.9800
C(21)-N(4)	1.328(5)
C(21)-N(3)	1.332(5)
C(21)-C(22)	1.508(6)
C(22)-H(22A)	0.9800
C(22)-H(22B)	0.9800
C(22)-H(22C)	0.9800
C(23)-C(28)	1.408(7)
C(23)-C(24)	1.409(7)

C(23)-N(4)	1.433(5)
C(24)-C(25)	1.390(6)
C(24)-C(29)	1.492(7)
C(25)-C(26)	1.372(8)
C(25)-H(25)	0.9500
C(26)-C(27)	1.368(8)
C(26)-H(26)	0.9500
C(27)-C(28)	1.397(7)
C(27)-H(27)	0.9500
C(28)-C(30)	1.504(8)
C(29)-H(29A)	0.9800
C(29)-H(29B)	0.9800
C(29)-H(29C)	0.9800
C(30)-H(30A)	0.9800
C(30)-H(30B)	0.9800
C(30)-H(30C)	0.9800
C(31)-C(32)	1.376(7)
C(31)-N(5)	1.418(6)
C(31)-C(40)	1.446(7)
C(32)-C(33)	1.409(8)
C(32)-H(32)	0.9500
C(33)-C(34)	1.361(9)
C(33)-H(33)	0.9500
C(34)-C(35)	1.399(9)
C(34)-H(34)	0.9500
C(35)-C(36)	1.393(9)
C(35)-C(40)	1.433(7)
C(36)-C(37)	1.358(10)
C(36)-H(36)	0.9500
C(37)-C(38)	1.423(9)
C(37)-H(37)	0.9500
C(38)-C(39)	1.391(7)
C(38)-H(38)	0.9500
C(39)-C(40)	1.427(7)
C(39)-N(7)	1.427(6)
C(41)-N(6)	1.326(6)
C(41)-N(5)	1.339(6)
C(41)-C(42)	1.513(7)
C(42)-H(42A)	0.9800
C(42)-H(42B)	0.9800
C(42)-H(42C)	0.9800
C(43)-C(44)	1.388(7)
C(43)-C(48)	1.395(7)
C(43)-N(6)	1.426(6)
C(44)-C(45)	1.362(8)
C(44)-C(49)	1.516(9)
C(45)-C(46)	1.389(11)
C(45)-H(45)	0.9500
C(46)-C(47)	1.390(10)
C(46)-H(46)	0.9500
C(47)-C(48)	1.396(8)
C(47)-H(47)	0.9500
C(48)-C(50)	1.519(8)
C(49)-H(49A)	0.9800

C(49)-H(49B)	0.9800
C(49)-H(49C)	0.9800
C(50)-H(50A)	0.9800
C(50)-H(50B)	0.9800
C(50)-H(50C)	0.9800
C(51)-N(8)	1.319(6)
C(51)-N(7)	1.326(6)
C(51)-C(52)	1.503(6)
C(51)-Sn(2)	2.662(5)
C(52)-H(52A)	0.9800
C(52)-H(52B)	0.9800
C(52)-H(52C)	0.9800
C(53)-C(58)	1.405(7)
C(53)-C(54)	1.411(7)
C(53)-N(8)	1.422(6)
C(54)-C(55)	1.384(9)
C(54)-C(59)	1.509(9)
C(55)-C(56)	1.377(11)
C(55)-H(55)	0.9500
C(56)-C(57)	1.365(10)
C(56)-H(56)	0.9500
C(57)-C(58)	1.389(8)
C(57)-H(57)	0.9500
C(58)-C(60)	1.502(8)
C(59)-H(59A)	0.9800
C(59)-H(59B)	0.9800
C(59)-H(59C)	0.9800
C(60)-H(60A)	0.9800
C(60)-H(60B)	0.9800
C(60)-H(60C)	0.9800
C(61)-O(1)	1.396(9)
C(61)-C(62)	1.423(11)
C(61)-H(61A)	0.9900
C(61)-H(61B)	0.9900
C(62)-C(63)	1.498(13)
C(62)-H(62A)	0.9900
C(62)-H(62B)	0.9900
C(63)-C(64)	1.498(11)
C(63)-H(63A)	0.9900
C(63)-H(63B)	0.9900
C(64)-O(1)	1.378(9)
C(64)-H(64A)	0.9900
C(64)-H(64B)	0.9900
N(1)-Sn(1)	2.130(4)
N(2)-Sn(1)	2.374(3)
N(3)-Sn(1)	2.240(3)
N(4)-Sn(1)	2.198(4)
N(5)-Sn(2)	2.252(4)
N(6)-Sn(2)	2.189(4)
N(7)-Sn(2)	2.148(4)
N(8)-Sn(2)	2.327(4)
S(1)-Sn(1)	2.4172(14)
S(1)-Sn(1)#1	2.4585(13)
S(2)-Sn(2)	2.4271(17)

S(2)-Sn(2)#2	2.4546(13)
C(2)-C(1)-N(1)	117.6(5)
C(2)-C(1)-C(10)	121.9(4)
N(1)-C(1)-C(10)	120.5(4)
C(1)-C(2)-C(3)	121.0(6)
C(1)-C(2)-H(2)	119.5
C(3)-C(2)-H(2)	119.5
C(4)-C(3)-C(2)	118.9(6)
C(4)-C(3)-H(3)	120.6
C(2)-C(3)-H(3)	120.6
C(3)-C(4)-C(5)	122.8(5)
C(3)-C(4)-H(4)	118.6
C(5)-C(4)-H(4)	118.6
C(6)-C(5)-C(4)	120.5(5)
C(6)-C(5)-C(10)	120.4(5)
C(4)-C(5)-C(10)	119.1(5)
C(7)-C(6)-C(5)	121.1(5)
C(7)-C(6)-H(6)	119.4
C(5)-C(6)-H(6)	119.4
C(6)-C(7)-C(8)	120.2(5)
C(6)-C(7)-H(7)	119.9
C(8)-C(7)-H(7)	119.9
C(9)-C(8)-C(7)	121.8(5)
C(9)-C(8)-H(8)	119.1
C(7)-C(8)-H(8)	119.1
C(8)-C(9)-N(3)	123.2(4)
C(8)-C(9)-C(10)	118.9(4)
N(3)-C(9)-C(10)	117.7(4)
C(5)-C(10)-C(1)	116.1(4)
C(5)-C(10)-C(9)	117.4(4)
C(1)-C(10)-C(9)	126.5(4)
N(2)-C(11)-N(1)	112.8(4)
N(2)-C(11)-C(12)	125.4(4)
N(1)-C(11)-C(12)	121.8(4)
C(11)-C(12)-H(12A)	109.5
C(11)-C(12)-H(12B)	109.5
H(12A)-C(12)-H(12B)	109.5
C(11)-C(12)-H(12C)	109.5
H(12A)-C(12)-H(12C)	109.5
H(12B)-C(12)-H(12C)	109.5
C(14)-C(13)-C(18)	120.9(4)
C(14)-C(13)-N(2)	120.1(4)
C(18)-C(13)-N(2)	119.0(4)
C(15)-C(14)-C(13)	118.7(4)
C(15)-C(14)-C(19)	120.4(4)
C(13)-C(14)-C(19)	120.9(4)
C(16)-C(15)-C(14)	121.0(5)
C(16)-C(15)-H(15)	119.5
C(14)-C(15)-H(15)	119.5
C(17)-C(16)-C(15)	120.0(5)
C(17)-C(16)-H(16)	120.0
C(15)-C(16)-H(16)	120.0
C(16)-C(17)-C(18)	121.1(5)

C(16)-C(17)-H(17)	119.5
C(18)-C(17)-H(17)	119.5
C(17)-C(18)-C(13)	118.4(4)
C(17)-C(18)-C(20)	120.1(4)
C(13)-C(18)-C(20)	121.5(4)
C(14)-C(19)-H(19A)	109.5
C(14)-C(19)-H(19B)	109.5
H(19A)-C(19)-H(19B)	109.5
C(14)-C(19)-H(19C)	109.5
H(19A)-C(19)-H(19C)	109.5
H(19B)-C(19)-H(19C)	109.5
C(18)-C(20)-H(20A)	109.5
C(18)-C(20)-H(20B)	109.5
H(20A)-C(20)-H(20B)	109.5
C(18)-C(20)-H(20C)	109.5
H(20A)-C(20)-H(20C)	109.5
H(20B)-C(20)-H(20C)	109.5
N(4)-C(21)-N(3)	110.9(4)
N(4)-C(21)-C(22)	121.1(4)
N(3)-C(21)-C(22)	128.0(4)
C(21)-C(22)-H(22A)	109.5
C(21)-C(22)-H(22B)	109.5
H(22A)-C(22)-H(22B)	109.5
C(21)-C(22)-H(22C)	109.5
H(22A)-C(22)-H(22C)	109.5
H(22B)-C(22)-H(22C)	109.5
C(28)-C(23)-C(24)	121.4(4)
C(28)-C(23)-N(4)	119.5(4)
C(24)-C(23)-N(4)	119.1(4)
C(25)-C(24)-C(23)	117.5(5)
C(25)-C(24)-C(29)	120.1(5)
C(23)-C(24)-C(29)	122.4(4)
C(26)-C(25)-C(24)	121.6(6)
C(26)-C(25)-H(25)	119.2
C(24)-C(25)-H(25)	119.2
C(27)-C(26)-C(25)	120.8(5)
C(27)-C(26)-H(26)	119.6
C(25)-C(26)-H(26)	119.6
C(26)-C(27)-C(28)	120.7(5)
C(26)-C(27)-H(27)	119.6
C(28)-C(27)-H(27)	119.6
C(27)-C(28)-C(23)	118.1(5)
C(27)-C(28)-C(30)	120.7(5)
C(23)-C(28)-C(30)	121.2(5)
C(24)-C(29)-H(29A)	109.5
C(24)-C(29)-H(29B)	109.5
H(29A)-C(29)-H(29B)	109.5
C(24)-C(29)-H(29C)	109.5
H(29A)-C(29)-H(29C)	109.5
H(29B)-C(29)-H(29C)	109.5
C(28)-C(30)-H(30A)	109.5
C(28)-C(30)-H(30B)	109.5
H(30A)-C(30)-H(30B)	109.5
C(28)-C(30)-H(30C)	109.5

H(30A)-C(30)-H(30C)	109.5
H(30B)-C(30)-H(30C)	109.5
C(32)-C(31)-N(5)	123.8(5)
C(32)-C(31)-C(40)	119.0(5)
N(5)-C(31)-C(40)	117.1(4)
C(31)-C(32)-C(33)	121.8(6)
C(31)-C(32)-H(32)	119.1
C(33)-C(32)-H(32)	119.1
C(34)-C(33)-C(32)	119.6(6)
C(34)-C(33)-H(33)	120.2
C(32)-C(33)-H(33)	120.2
C(33)-C(34)-C(35)	121.1(6)
C(33)-C(34)-H(34)	119.4
C(35)-C(34)-H(34)	119.4
C(36)-C(35)-C(34)	119.8(6)
C(36)-C(35)-C(40)	120.0(6)
C(34)-C(35)-C(40)	120.2(6)
C(37)-C(36)-C(35)	121.1(6)
C(37)-C(36)-H(36)	119.4
C(35)-C(36)-H(36)	119.4
C(36)-C(37)-C(38)	120.6(6)
C(36)-C(37)-H(37)	119.7
C(38)-C(37)-H(37)	119.7
C(39)-C(38)-C(37)	119.3(6)
C(39)-C(38)-H(38)	120.4
C(37)-C(38)-H(38)	120.4
C(38)-C(39)-C(40)	120.4(5)
C(38)-C(39)-N(7)	118.8(5)
C(40)-C(39)-N(7)	120.8(4)
C(39)-C(40)-C(35)	117.5(5)
C(39)-C(40)-C(31)	125.3(5)
C(35)-C(40)-C(31)	117.2(5)
N(6)-C(41)-N(5)	110.2(4)
N(6)-C(41)-C(42)	122.6(4)
N(5)-C(41)-C(42)	127.3(4)
C(41)-C(42)-H(42A)	109.5
C(41)-C(42)-H(42B)	109.5
H(42A)-C(42)-H(42B)	109.5
C(41)-C(42)-H(42C)	109.5
H(42A)-C(42)-H(42C)	109.5
H(42B)-C(42)-H(42C)	109.5
C(44)-C(43)-C(48)	120.9(5)
C(44)-C(43)-N(6)	120.8(5)
C(48)-C(43)-N(6)	118.1(4)
C(45)-C(44)-C(43)	119.5(6)
C(45)-C(44)-C(49)	119.2(6)
C(43)-C(44)-C(49)	121.3(5)
C(44)-C(45)-C(46)	120.7(7)
C(44)-C(45)-H(45)	119.6
C(46)-C(45)-H(45)	119.6
C(45)-C(46)-C(47)	120.2(6)
C(45)-C(46)-H(46)	119.9
C(47)-C(46)-H(46)	119.9
C(46)-C(47)-C(48)	119.5(7)

C(46)-C(47)-H(47)	120.3
C(48)-C(47)-H(47)	120.3
C(43)-C(48)-C(47)	119.0(6)
C(43)-C(48)-C(50)	121.2(5)
C(47)-C(48)-C(50)	119.8(6)
C(44)-C(49)-H(49A)	109.5
C(44)-C(49)-H(49B)	109.5
H(49A)-C(49)-H(49B)	109.5
C(44)-C(49)-H(49C)	109.5
H(49A)-C(49)-H(49C)	109.5
H(49B)-C(49)-H(49C)	109.5
C(48)-C(50)-H(50A)	109.5
C(48)-C(50)-H(50B)	109.5
H(50A)-C(50)-H(50B)	109.5
C(48)-C(50)-H(50C)	109.5
H(50A)-C(50)-H(50C)	109.5
H(50B)-C(50)-H(50C)	109.5
N(8)-C(51)-N(7)	113.7(4)
N(8)-C(51)-C(52)	123.4(4)
N(7)-C(51)-C(52)	122.9(5)
N(8)-C(51)-Sn(2)	60.9(2)
N(7)-C(51)-Sn(2)	53.2(2)
C(52)-C(51)-Sn(2)	172.2(4)
C(51)-C(52)-H(52A)	109.5
C(51)-C(52)-H(52B)	109.5
H(52A)-C(52)-H(52B)	109.5
C(51)-C(52)-H(52C)	109.5
H(52A)-C(52)-H(52C)	109.5
H(52B)-C(52)-H(52C)	109.5
C(58)-C(53)-C(54)	121.6(5)
C(58)-C(53)-N(8)	119.5(5)
C(54)-C(53)-N(8)	118.9(5)
C(55)-C(54)-C(53)	117.7(6)
C(55)-C(54)-C(59)	121.7(6)
C(53)-C(54)-C(59)	120.5(5)
C(56)-C(55)-C(54)	121.2(7)
C(56)-C(55)-H(55)	119.4
C(54)-C(55)-H(55)	119.4
C(57)-C(56)-C(55)	120.5(6)
C(57)-C(56)-H(56)	119.8
C(55)-C(56)-H(56)	119.8
C(56)-C(57)-C(58)	121.5(7)
C(56)-C(57)-H(57)	119.2
C(58)-C(57)-H(57)	119.2
C(57)-C(58)-C(53)	117.5(6)
C(57)-C(58)-C(60)	121.3(6)
C(53)-C(58)-C(60)	121.2(5)
C(54)-C(59)-H(59A)	109.5
C(54)-C(59)-H(59B)	109.5
H(59A)-C(59)-H(59B)	109.5
C(54)-C(59)-H(59C)	109.5
H(59A)-C(59)-H(59C)	109.5
H(59B)-C(59)-H(59C)	109.5
C(58)-C(60)-H(60A)	109.5

C(58)-C(60)-H(60B)	109.5
H(60A)-C(60)-H(60B)	109.5
C(58)-C(60)-H(60C)	109.5
H(60A)-C(60)-H(60C)	109.5
H(60B)-C(60)-H(60C)	109.5
O(1)-C(61)-C(62)	109.7(9)
O(1)-C(61)-H(61A)	109.7
C(62)-C(61)-H(61A)	109.7
O(1)-C(61)-H(61B)	109.7
C(62)-C(61)-H(61B)	109.7
H(61A)-C(61)-H(61B)	108.2
C(61)-C(62)-C(63)	106.7(8)
C(61)-C(62)-H(62A)	110.4
C(63)-C(62)-H(62A)	110.4
C(61)-C(62)-H(62B)	110.4
C(63)-C(62)-H(62B)	110.4
H(62A)-C(62)-H(62B)	108.6
C(62)-C(63)-C(64)	101.4(7)
C(62)-C(63)-H(63A)	111.5
C(64)-C(63)-H(63A)	111.5
C(62)-C(63)-H(63B)	111.5
C(64)-C(63)-H(63B)	111.5
H(63A)-C(63)-H(63B)	109.3
O(1)-C(64)-C(63)	108.8(8)
O(1)-C(64)-H(64A)	109.9
C(63)-C(64)-H(64A)	109.9
O(1)-C(64)-H(64B)	109.9
C(63)-C(64)-H(64B)	109.9
H(64A)-C(64)-H(64B)	108.3
C(11)-N(1)-C(1)	123.0(4)
C(11)-N(1)-Sn(1)	99.0(3)
C(1)-N(1)-Sn(1)	125.9(3)
C(11)-N(2)-C(13)	120.9(4)
C(11)-N(2)-Sn(1)	88.9(3)
C(13)-N(2)-Sn(1)	150.1(3)
C(21)-N(3)-C(9)	132.6(4)
C(21)-N(3)-Sn(1)	93.9(3)
C(9)-N(3)-Sn(1)	132.2(3)
C(21)-N(4)-C(23)	125.3(4)
C(21)-N(4)-Sn(1)	95.9(3)
C(23)-N(4)-Sn(1)	138.0(3)
C(41)-N(5)-C(31)	131.7(4)
C(41)-N(5)-Sn(2)	93.7(3)
C(31)-N(5)-Sn(2)	134.5(3)
C(41)-N(6)-C(43)	125.7(4)
C(41)-N(6)-Sn(2)	97.0(3)
C(43)-N(6)-Sn(2)	135.1(3)
C(51)-N(7)-C(39)	119.4(4)
C(51)-N(7)-Sn(2)	97.2(3)
C(39)-N(7)-Sn(2)	123.8(3)
C(51)-N(8)-C(53)	125.2(4)
C(51)-N(8)-Sn(2)	89.4(3)
C(53)-N(8)-Sn(2)	145.3(3)
Sn(1)-S(1)-Sn(1)#1	87.92(4)

Sn(2)-S(2)-Sn(2)#2	87.14(4)
N(1)-Sn(1)-N(4)	131.38(14)
N(1)-Sn(1)-N(3)	77.63(14)
N(4)-Sn(1)-N(3)	59.16(13)
N(1)-Sn(1)-N(2)	58.65(13)
N(4)-Sn(1)-N(2)	99.06(13)
N(3)-Sn(1)-N(2)	91.81(12)
N(1)-Sn(1)-S(1)	118.91(10)
N(4)-Sn(1)-S(1)	103.69(10)
N(3)-Sn(1)-S(1)	162.73(10)
N(2)-Sn(1)-S(1)	93.17(9)
N(1)-Sn(1)-S(1)#1	96.05(10)
N(4)-Sn(1)-S(1)#1	105.19(10)
N(3)-Sn(1)-S(1)#1	90.90(10)
N(2)-Sn(1)-S(1)#1	153.19(9)
S(1)-Sn(1)-S(1)#1	92.08(4)
N(7)-Sn(2)-N(6)	132.85(15)
N(7)-Sn(2)-N(5)	77.24(15)
N(6)-Sn(2)-N(5)	58.93(14)
N(7)-Sn(2)-N(8)	59.18(14)
N(6)-Sn(2)-N(8)	97.54(14)
N(5)-Sn(2)-N(8)	84.40(13)
N(7)-Sn(2)-S(2)	120.77(12)
N(6)-Sn(2)-S(2)	100.09(10)
N(5)-Sn(2)-S(2)	158.57(10)
N(8)-Sn(2)-S(2)	95.04(10)
N(7)-Sn(2)-S(2)#2	98.24(11)
N(6)-Sn(2)-S(2)#2	102.43(10)
N(5)-Sn(2)-S(2)#2	95.94(10)
N(8)-Sn(2)-S(2)#2	156.87(11)
S(2)-Sn(2)-S(2)#2	92.86(4)
N(7)-Sn(2)-C(51)	29.63(14)
N(6)-Sn(2)-C(51)	116.03(15)
N(5)-Sn(2)-C(51)	77.16(14)
N(8)-Sn(2)-C(51)	29.71(14)
S(2)-Sn(2)-C(51)	112.22(11)
S(2)#2-Sn(2)-C(51)	127.86(12)
C(64)-O(1)-C(61)	105.8(6)

Symmetry transformations used to generate equivalent atoms:
#1 -x,-y+1,-z+2   #2 -x,-y,-z+1

Table S28. Anisotropic displacement parameters (A^2 x 10^3) for R200. The anisotropic displacement factor exponent takes the form: -2 pi^2 [h^2 a*^2 U11 + ... + 2 h k a* b* U12]

	U11	U22	U33	U23	U13	U12
C(1)	28(2)	45(3)	24(2)	7(2)	-3(2)	-4(2)
C(2)	48(3)	34(3)	64(4)	13(3)	3(3)	0(2)
C(3)	54(4)	40(3)	94(5)	16(3)	-3(4)	-13(3)
C(4)	38(3)	57(4)	74(4)	18(3)	0(3)	-15(3)
C(5)	33(3)	55(3)	38(3)	11(2)	3(2)	-5(2)
C(6)	30(3)	73(4)	48(3)	9(3)	1(2)	-4(3)
C(7)	25(3)	83(5)	52(3)	7(3)	2(2)	14(3)
C(8)	25(2)	55(3)	48(3)	10(3)	7(2)	10(2)
C(9)	26(2)	49(3)	22(2)	3(2)	4(2)	7(2)
C(10)	28(2)	38(3)	27(2)	7(2)	2(2)	-1(2)
C(11)	38(3)	36(3)	29(2)	7(2)	7(2)	15(2)
C(12)	52(3)	48(3)	32(3)	14(2)	6(2)	5(3)
C(13)	30(2)	31(2)	30(2)	5(2)	2(2)	8(2)
C(14)	38(3)	41(3)	31(2)	11(2)	6(2)	17(2)
C(15)	35(3)	47(3)	52(3)	11(3)	-8(2)	16(2)
C(16)	52(3)	43(3)	41(3)	-4(2)	-16(2)	12(3)
C(17)	51(3)	50(3)	39(3)	-9(2)	-6(2)	22(3)
C(18)	35(2)	38(3)	34(3)	-2(2)	2(2)	13(2)
C(19)	55(3)	67(4)	43(3)	13(3)	9(3)	39(3)
C(20)	43(3)	68(4)	50(3)	-4(3)	9(3)	27(3)
C(21)	31(2)	33(3)	34(2)	4(2)	8(2)	10(2)
C(22)	38(3)	48(3)	86(4)	14(3)	21(3)	22(3)
C(23)	22(2)	26(2)	56(3)	4(2)	4(2)	10(2)
C(24)	35(3)	29(3)	64(3)	12(2)	10(2)	13(2)
C(25)	42(3)	37(3)	76(4)	24(3)	13(3)	15(2)
C(26)	33(3)	34(3)	99(5)	24(3)	6(3)	6(2)
C(27)	32(3)	30(3)	89(5)	-2(3)	-11(3)	6(2)
C(28)	31(3)	36(3)	62(4)	1(3)	-3(2)	11(2)
C(29)	77(4)	42(3)	52(3)	13(3)	11(3)	22(3)
C(30)	54(3)	50(3)	61(4)	-6(3)	-7(3)	11(3)
C(31)	34(3)	39(3)	45(3)	5(2)	10(2)	7(2)
C(32)	48(3)	37(3)	68(4)	4(3)	6(3)	8(3)
C(33)	54(4)	44(4)	97(5)	-10(4)	10(4)	6(3)
C(34)	41(3)	65(4)	71(4)	-18(4)	7(3)	0(3)
C(35)	31(3)	76(4)	47(3)	-3(3)	11(2)	10(3)
C(36)	57(4)	109(6)	42(4)	-5(4)	6(3)	24(4)
C(37)	60(4)	137(7)	30(3)	11(4)	-1(3)	31(4)
C(38)	63(4)	106(5)	38(3)	25(3)	1(3)	39(4)
C(39)	30(2)	60(3)	39(3)	16(3)	8(2)	13(2)
C(40)	31(3)	55(3)	39(3)	5(2)	10(2)	8(2)
C(41)	37(3)	38(3)	36(3)	16(2)	10(2)	17(2)
C(42)	66(4)	47(3)	57(4)	13(3)	2(3)	30(3)
C(43)	43(3)	44(3)	45(3)	13(2)	-7(2)	19(2)
C(44)	38(3)	81(4)	63(4)	36(3)	-4(3)	23(3)
C(45)	46(4)	106(6)	103(6)	51(5)	-16(4)	15(4)

C(46)	67(5)	72(5)	100(6)	27(4)	-44(5)	-5(4)
C(47)	87(5)	56(4)	60(4)	5(3)	-28(4)	19(4)
C(48)	62(4)	49(3)	44(3)	4(3)	-12(3)	25(3)
C(49)	61(4)	164(8)	86(5)	65(5)	29(4)	69(5)
C(50)	92(5)	85(5)	42(3)	-6(3)	5(3)	42(4)
C(51)	30(2)	41(3)	47(3)	8(2)	5(2)	17(2)
C(52)	27(3)	78(4)	59(4)	9(3)	5(2)	11(3)
C(53)	26(2)	49(3)	41(3)	-3(2)	7(2)	6(2)
C(54)	23(2)	48(3)	84(4)	-19(3)	2(3)	3(2)
C(55)	31(3)	91(5)	91(5)	-50(4)	14(3)	2(3)
C(56)	41(4)	147(8)	52(4)	-37(5)	10(3)	7(4)
C(57)	45(3)	104(6)	43(4)	7(4)	5(3)	1(4)
C(58)	35(3)	69(4)	41(3)	5(3)	8(2)	9(3)
C(59)	49(4)	41(4)	177(8)	10(4)	40(4)	14(3)
C(60)	69(4)	65(4)	64(4)	32(3)	24(3)	20(3)
C(61)	107(7)	126(8)	137(8)	25(6)	25(6)	68(6)
C(62)	118(8)	194(11)	127(9)	-94(8)	-55(7)	106(8)
C(63)	81(6)	44(4)	176(10)	15(5)	-34(6)	5(4)
C(64)	130(8)	159(9)	88(6)	13(6)	-8(6)	93(7)
N(1)	32(2)	29(2)	30(2)	7(2)	6(2)	5(2)
N(2)	27(2)	33(2)	25(2)	5(2)	5(2)	8(2)
N(3)	23(2)	33(2)	32(2)	1(2)	6(2)	7(2)
N(4)	28(2)	24(2)	45(2)	6(2)	10(2)	10(2)
N(5)	32(2)	32(2)	39(2)	10(2)	4(2)	10(2)
N(6)	34(2)	30(2)	36(2)	6(2)	2(2)	16(2)
N(7)	32(2)	55(3)	35(2)	16(2)	8(2)	17(2)
N(8)	34(2)	35(2)	44(2)	7(2)	13(2)	11(2)
S(1)	24(1)	32(1)	29(1)	3(1)	3(1)	6(1)
S(2)	37(1)	34(1)	40(1)	7(1)	13(1)	14(1)
Sn(1)	25(1)	24(1)	27(1)	4(1)	5(1)	6(1)
Sn(2)	26(1)	32(1)	35(1)	10(1)	6(1)	12(1)
O(1)	88(4)	234(8)	82(4)	11(4)	0(3)	92(5)

Crystal data and structure refinement of compound 2a

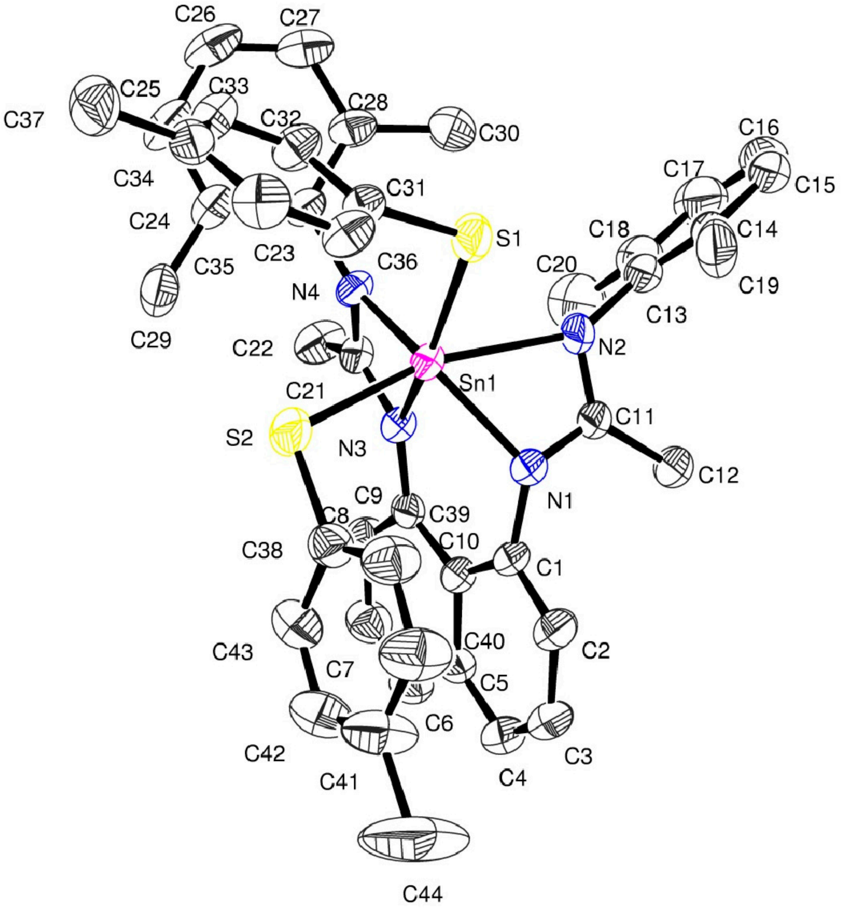

Figure S11. Asymmetric unit

Table S29. Crystal data and structure refinement for R202.

Identification code	R202
Empirical formula	C44 H44 N4 S2 Sn
Formula weight	811.64
Temperature	193(2) K
Wavelength	0.71073 A
Crystal system, space group	Monoclinic, P 21/c
Unit cell dimensions	a = 19.655(17) A alpha = 90 deg.
	b = 11.760(8) A beta = 104.67(4) deg.
	c = 20.040(17) A gamma = 90 deg.

Volume	4481(6) A^3
Z, Calculated density	4, 1.203 Mg/m^3
Absorption coefficient	0.695 mm^-1
F(000)	1672
Crystal size	0.120 x 0.060 x 0.060 mm
Theta range for data collection	2.723 to 29.596 deg.
Limiting indices	-26<=h<=27, -16<=k<=16, -27<=l<=27
Reflections collected / unique	124683 / 12518 [R(int) = 0.1184]
Completeness to theta = 25.242	99.5 %
Refinement method	Full-matrix least-squares on F^2
Data / restraints / parameters	12518 / 0 / 468
Goodness-of-fit on F^2	0.996
Final R indices [I>2sigma(I)]	R1 = 0.0473, wR2 = 0.1191
R indices (all data)	R1 = 0.0947, wR2 = 0.1422
Largest diff. peak and hole	0.537 and -0.874 e.A^-3

Table S30. Atomic coordinates (x 10^4) and equivalent isotropic displacement parameters (A^2 x 10^3) for R202.
U(eq) is defined as one third of the trace of the orthogonalized Uij tensor.

	x	y	z	U(eq)
C(1)	1960(2)	7174(3)	5651(2)	40(1)
C(2)	1523(2)	7951(3)	5862(2)	49(1)
C(3)	1459(2)	9075(3)	5605(2)	55(1)
C(4)	1823(2)	9403(3)	5142(2)	52(1)
C(5)	2296(2)	8642(3)	4937(2)	41(1)
C(6)	2675(2)	9027(3)	4466(2)	47(1)
C(7)	3132(2)	8320(3)	4261(2)	51(1)
C(8)	3244(2)	7209(3)	4523(2)	44(1)
C(9)	2893(2)	6784(3)	4991(2)	35(1)
C(10)	2384(2)	7498(3)	5205(2)	36(1)
C(11)	1506(2)	5294(3)	5435(2)	41(1)
C(12)	849(2)	5699(3)	4941(2)	63(1)
C(13)	1325(2)	3333(3)	5118(2)	53(1)
C(14)	865(2)	2741(3)	5436(3)	63(1)
C(15)	478(2)	1848(4)	5068(4)	84(2)
C(16)	538(3)	1553(4)	4422(4)	90(2)
C(17)	989(3)	2136(4)	4125(3)	89(2)
C(18)	1396(2)	3043(4)	4464(2)	66(1)
C(19)	777(2)	3073(4)	6129(3)	80(2)
C(20)	1857(3)	3691(4)	4121(2)	84(2)
C(21)	3498(2)	4900(3)	5253(2)	37(1)
C(22)	3888(2)	4762(3)	4700(2)	55(1)
C(23)	4033(2)	3193(3)	5890(2)	37(1)
C(24)	4737(2)	3402(3)	6228(2)	43(1)
C(25)	5187(2)	2466(4)	6404(2)	54(1)
C(26)	4948(2)	1369(4)	6262(2)	61(1)
C(27)	4251(2)	1182(3)	5937(2)	54(1)
C(28)	3778(2)	2082(3)	5743(2)	43(1)
C(29)	5011(2)	4575(3)	6449(2)	54(1)
C(30)	3014(2)	1866(3)	5398(2)	58(1)
C(31)	3336(2)	3378(3)	7696(2)	39(1)
C(32)	3925(2)	2785(3)	7626(2)	45(1)
C(33)	4500(2)	2663(3)	8181(2)	46(1)
C(34)	4512(2)	3111(3)	8825(2)	42(1)
C(35)	3921(2)	3687(3)	8889(2)	52(1)
C(36)	3335(2)	3834(3)	8335(2)	47(1)
C(37)	5143(2)	2959(4)	9431(2)	62(1)
C(38)	3226(2)	7381(3)	7131(2)	43(1)
C(39)	2722(2)	7433(3)	7504(2)	57(1)
C(40)	2420(3)	8455(4)	7605(2)	73(1)
C(41)	2608(3)	9444(4)	7331(3)	79(2)
C(42)	3103(3)	9390(4)	6958(2)	67(1)
C(43)	3412(2)	8391(3)	6857(2)	53(1)
C(44)	2222(4)	10570(5)	7417(4)	153(3)

N(1)	1928(1)	6031(2)	5877(1)	38(1)
N(2)	1743(1)	4257(2)	5488(2)	44(1)
N(3)	3053(1)	5725(2)	5316(1)	35(1)
N(4)	3544(1)	4119(2)	5747(1)	35(1)
S(1)	2568(1)	3477(1)	7001(1)	48(1)
S(2)	3685(1)	6111(1)	7058(1)	57(1)
Sn(1)	2816(1)	4955(1)	6249(1)	34(1)

Table S31. Bond lengths [A] and angles [deg] for R202.

C(1)-C(2)	1.391(5)
C(1)-C(10)	1.420(5)
C(1)-N(1)	1.425(4)
C(2)-C(3)	1.412(5)
C(2)-H(2)	0.9500
C(3)-C(4)	1.362(5)
C(3)-H(3)	0.9500
C(4)-C(5)	1.424(5)
C(4)-H(4)	0.9500
C(5)-C(6)	1.418(5)
C(5)-C(10)	1.442(4)
C(6)-C(7)	1.362(5)
C(6)-H(6)	0.9500
C(7)-C(8)	1.404(5)
C(7)-H(7)	0.9500
C(8)-C(9)	1.391(5)
C(8)-H(8)	0.9500
C(9)-N(3)	1.403(4)
C(9)-C(10)	1.451(5)
C(11)-N(2)	1.301(4)
C(11)-N(1)	1.361(4)
C(11)-C(12)	1.493(5)
C(12)-H(12A)	0.9800
C(12)-H(12B)	0.9800
C(12)-H(12C)	0.9800
C(13)-C(18)	1.394(6)
C(13)-C(14)	1.415(6)
C(13)-N(2)	1.448(5)
C(14)-C(15)	1.392(6)
C(14)-C(19)	1.494(7)
C(15)-C(16)	1.375(8)
C(15)-H(15)	0.9500
C(16)-C(17)	1.369(8)
C(16)-H(16)	0.9500
C(17)-C(18)	1.401(6)
C(17)-H(17)	0.9500
C(18)-C(20)	1.481(6)
C(19)-H(19A)	0.9800
C(19)-H(19B)	0.9800
C(19)-H(19C)	0.9800
C(20)-H(20A)	0.9800
C(20)-H(20B)	0.9800
C(20)-H(20C)	0.9800
C(21)-N(3)	1.334(4)
C(21)-N(4)	1.337(4)
C(21)-C(22)	1.506(5)
C(22)-H(22A)	0.9800
C(22)-H(22B)	0.9800
C(22)-H(22C)	0.9800
C(23)-C(24)	1.400(5)
C(23)-C(28)	1.405(5)

C(23)-N(4)	1.432(4)
C(24)-C(25)	1.400(5)
C(24)-C(29)	1.507(5)
C(25)-C(26)	1.378(6)
C(25)-H(25)	0.9500
C(26)-C(27)	1.377(6)
C(26)-H(26)	0.9500
C(27)-C(28)	1.398(5)
C(27)-H(27)	0.9500
C(28)-C(30)	1.506(5)
C(29)-H(29A)	0.9800
C(29)-H(29B)	0.9800
C(29)-H(29C)	0.9800
C(30)-H(30A)	0.9800
C(30)-H(30B)	0.9800
C(30)-H(30C)	0.9800
C(31)-C(36)	1.389(5)
C(31)-C(32)	1.389(5)
C(31)-S(1)	1.779(4)
C(32)-C(33)	1.378(5)
C(32)-H(32)	0.9500
C(33)-C(34)	1.389(5)
C(33)-H(33)	0.9500
C(34)-C(35)	1.380(5)
C(34)-C(37)	1.510(5)
C(35)-C(36)	1.393(5)
C(35)-H(35)	0.9500
C(36)-H(36)	0.9500
C(37)-H(37A)	0.9800
C(37)-H(37B)	0.9800
C(37)-H(37C)	0.9800
C(38)-C(39)	1.387(5)
C(38)-C(43)	1.395(5)
C(38)-S(2)	1.771(4)
C(39)-C(40)	1.378(5)
C(39)-H(39)	0.9500
C(40)-C(41)	1.377(6)
C(40)-H(40)	0.9500
C(41)-C(42)	1.369(6)
C(41)-C(44)	1.558(7)
C(42)-C(43)	1.362(6)
C(42)-H(42)	0.9500
C(43)-H(43)	0.9500
C(44)-H(44A)	0.9800
C(44)-H(44B)	0.9800
C(44)-H(44C)	0.9800
N(1)-Sn(1)	2.132(3)
N(2)-Sn(1)	2.411(3)
N(3)-Sn(1)	2.230(3)
N(4)-Sn(1)	2.180(3)
S(1)-Sn(1)	2.4297(15)
S(2)-Sn(1)	2.4483(16)
C(2)-C(1)-C(10)	121.4(3)

C(2)-C(1)-N(1)	116.2(3)
C(10)-C(1)-N(1)	122.3(3)
C(1)-C(2)-C(3)	120.6(4)
C(1)-C(2)-H(2)	119.7
C(3)-C(2)-H(2)	119.7
C(4)-C(3)-C(2)	120.0(3)
C(4)-C(3)-H(3)	120.0
C(2)-C(3)-H(3)	120.0
C(3)-C(4)-C(5)	120.7(3)
C(3)-C(4)-H(4)	119.6
C(5)-C(4)-H(4)	119.6
C(6)-C(5)-C(4)	118.9(3)
C(6)-C(5)-C(10)	120.7(3)
C(4)-C(5)-C(10)	120.4(3)
C(7)-C(6)-C(5)	120.3(3)
C(7)-C(6)-H(6)	119.9
C(5)-C(6)-H(6)	119.9
C(6)-C(7)-C(8)	120.6(3)
C(6)-C(7)-H(7)	119.7
C(8)-C(7)-H(7)	119.7
C(9)-C(8)-C(7)	122.0(3)
C(9)-C(8)-H(8)	119.0
C(7)-C(8)-H(8)	119.0
C(8)-C(9)-N(3)	122.8(3)
C(8)-C(9)-C(10)	119.2(3)
N(3)-C(9)-C(10)	117.8(3)
C(1)-C(10)-C(5)	116.7(3)
C(1)-C(10)-C(9)	126.1(3)
C(5)-C(10)-C(9)	117.2(3)
N(2)-C(11)-N(1)	113.5(3)
N(2)-C(11)-C(12)	125.8(3)
N(1)-C(11)-C(12)	120.7(3)
C(11)-C(12)-H(12A)	109.5
C(11)-C(12)-H(12B)	109.5
H(12A)-C(12)-H(12B)	109.5
C(11)-C(12)-H(12C)	109.5
H(12A)-C(12)-H(12C)	109.5
H(12B)-C(12)-H(12C)	109.5
C(18)-C(13)-C(14)	122.0(4)
C(18)-C(13)-N(2)	119.0(4)
C(14)-C(13)-N(2)	118.9(4)
C(15)-C(14)-C(13)	117.3(5)
C(15)-C(14)-C(19)	120.8(5)
C(13)-C(14)-C(19)	121.9(4)
C(16)-C(15)-C(14)	121.6(5)
C(16)-C(15)-H(15)	119.2
C(14)-C(15)-H(15)	119.2
C(17)-C(16)-C(15)	120.1(5)
C(17)-C(16)-H(16)	120.0
C(15)-C(16)-H(16)	120.0
C(16)-C(17)-C(18)	121.6(6)
C(16)-C(17)-H(17)	119.2
C(18)-C(17)-H(17)	119.2
C(13)-C(18)-C(17)	117.5(5)

C(13)-C(18)-C(20)	122.0(4)
C(17)-C(18)-C(20)	120.5(5)
C(14)-C(19)-H(19A)	109.5
C(14)-C(19)-H(19B)	109.5
H(19A)-C(19)-H(19B)	109.5
C(14)-C(19)-H(19C)	109.5
H(19A)-C(19)-H(19C)	109.5
H(19B)-C(19)-H(19C)	109.5
C(18)-C(20)-H(20A)	109.5
C(18)-C(20)-H(20B)	109.5
H(20A)-C(20)-H(20B)	109.5
C(18)-C(20)-H(20C)	109.5
H(20A)-C(20)-H(20C)	109.5
H(20B)-C(20)-H(20C)	109.5
N(3)-C(21)-N(4)	110.6(3)
N(3)-C(21)-C(22)	128.0(3)
N(4)-C(21)-C(22)	121.3(3)
C(21)-C(22)-H(22A)	109.5
C(21)-C(22)-H(22B)	109.5
H(22A)-C(22)-H(22B)	109.5
C(21)-C(22)-H(22C)	109.5
H(22A)-C(22)-H(22C)	109.5
H(22B)-C(22)-H(22C)	109.5
C(24)-C(23)-C(28)	121.4(3)
C(24)-C(23)-N(4)	119.7(3)
C(28)-C(23)-N(4)	118.6(3)
C(25)-C(24)-C(23)	118.0(3)
C(25)-C(24)-C(29)	119.4(3)
C(23)-C(24)-C(29)	122.5(3)
C(26)-C(25)-C(24)	121.5(4)
C(26)-C(25)-H(25)	119.3
C(24)-C(25)-H(25)	119.3
C(27)-C(26)-C(25)	119.6(3)
C(27)-C(26)-H(26)	120.2
C(25)-C(26)-H(26)	120.2
C(26)-C(27)-C(28)	121.5(4)
C(26)-C(27)-H(27)	119.2
C(28)-C(27)-H(27)	119.2
C(27)-C(28)-C(23)	118.0(4)
C(27)-C(28)-C(30)	121.1(3)
C(23)-C(28)-C(30)	121.0(3)
C(24)-C(29)-H(29A)	109.5
C(24)-C(29)-H(29B)	109.5
H(29A)-C(29)-H(29B)	109.5
C(24)-C(29)-H(29C)	109.5
H(29A)-C(29)-H(29C)	109.5
H(29B)-C(29)-H(29C)	109.5
C(28)-C(30)-H(30A)	109.5
C(28)-C(30)-H(30B)	109.5
H(30A)-C(30)-H(30B)	109.5
C(28)-C(30)-H(30C)	109.5
H(30A)-C(30)-H(30C)	109.5
H(30B)-C(30)-H(30C)	109.5
C(36)-C(31)-C(32)	118.8(3)

C(36)-C(31)-S(1)	119.8(3)
C(32)-C(31)-S(1)	121.2(3)
C(33)-C(32)-C(31)	120.3(3)
C(33)-C(32)-H(32)	119.9
C(31)-C(32)-H(32)	119.9
C(32)-C(33)-C(34)	122.0(3)
C(32)-C(33)-H(33)	119.0
C(34)-C(33)-H(33)	119.0
C(35)-C(34)-C(33)	117.1(3)
C(35)-C(34)-C(37)	121.6(4)
C(33)-C(34)-C(37)	121.3(4)
C(34)-C(35)-C(36)	122.2(3)
C(34)-C(35)-H(35)	118.9
C(36)-C(35)-H(35)	118.9
C(31)-C(36)-C(35)	119.6(3)
C(31)-C(36)-H(36)	120.2
C(35)-C(36)-H(36)	120.2
C(34)-C(37)-H(37A)	109.5
C(34)-C(37)-H(37B)	109.5
H(37A)-C(37)-H(37B)	109.5
C(34)-C(37)-H(37C)	109.5
H(37A)-C(37)-H(37C)	109.5
H(37B)-C(37)-H(37C)	109.5
C(39)-C(38)-C(43)	118.0(3)
C(39)-C(38)-S(2)	122.0(3)
C(43)-C(38)-S(2)	119.7(3)
C(40)-C(39)-C(38)	120.7(4)
C(40)-C(39)-H(39)	119.7
C(38)-C(39)-H(39)	119.7
C(41)-C(40)-C(39)	120.6(4)
C(41)-C(40)-H(40)	119.7
C(39)-C(40)-H(40)	119.7
C(42)-C(41)-C(40)	118.6(4)
C(42)-C(41)-C(44)	122.2(5)
C(40)-C(41)-C(44)	119.1(5)
C(43)-C(42)-C(41)	121.7(4)
C(43)-C(42)-H(42)	119.1
C(41)-C(42)-H(42)	119.1
C(42)-C(43)-C(38)	120.3(4)
C(42)-C(43)-H(43)	119.8
C(38)-C(43)-H(43)	119.8
C(41)-C(44)-H(44A)	109.5
C(41)-C(44)-H(44B)	109.5
H(44A)-C(44)-H(44B)	109.5
C(41)-C(44)-H(44C)	109.5
H(44A)-C(44)-H(44C)	109.5
H(44B)-C(44)-H(44C)	109.5
C(11)-N(1)-C(1)	117.5(3)
C(11)-N(1)-Sn(1)	99.1(2)
C(1)-N(1)-Sn(1)	124.9(2)
C(11)-N(2)-C(13)	121.1(3)
C(11)-N(2)-Sn(1)	88.3(2)
C(13)-N(2)-Sn(1)	150.6(2)
C(21)-N(3)-C(9)	132.6(3)

C(21)-N(3)-Sn(1)	93.8(2)
C(9)-N(3)-Sn(1)	132.8(2)
C(21)-N(4)-C(23)	125.9(3)
C(21)-N(4)-Sn(1)	95.9(2)
C(23)-N(4)-Sn(1)	137.4(2)
C(31)-S(1)-Sn(1)	105.59(12)
C(38)-S(2)-Sn(1)	103.78(13)
N(1)-Sn(1)-N(4)	132.96(11)
N(1)-Sn(1)-N(3)	78.01(11)
N(4)-Sn(1)-N(3)	59.72(10)
N(1)-Sn(1)-N(2)	58.27(11)
N(4)-Sn(1)-N(2)	97.20(12)
N(3)-Sn(1)-N(2)	86.71(11)
N(1)-Sn(1)-S(1)	111.48(9)
N(4)-Sn(1)-S(1)	102.83(8)
N(3)-Sn(1)-S(1)	158.14(7)
N(2)-Sn(1)-S(1)	82.44(9)
N(1)-Sn(1)-S(2)	105.90(9)
N(4)-Sn(1)-S(2)	97.26(9)
N(3)-Sn(1)-S(2)	94.06(9)
N(2)-Sn(1)-S(2)	163.67(8)
S(1)-Sn(1)-S(2)	101.67(7)

Symmetry transformations used to generate equivalent atoms:

Table S32. Anisotropic displacement parameters (A^2 x 10^3) for R202.
The anisotropic displacement factor exponent takes the form:
-2 pi^2 [h^2 a*^2 U11 + ... + 2 h k a* b* U12]

	U11	U22	U33	U23	U13	U12
C(1)	29(2)	30(2)	57(2)	10(2)	2(2)	6(1)
C(2)	40(2)	42(2)	66(3)	9(2)	13(2)	8(2)
C(3)	43(2)	38(2)	86(3)	7(2)	17(2)	11(2)
C(4)	44(2)	35(2)	72(3)	11(2)	8(2)	7(2)
C(5)	37(2)	32(2)	49(2)	5(1)	-1(2)	-2(1)
C(6)	52(2)	36(2)	50(2)	10(2)	4(2)	-1(2)
C(7)	57(2)	46(2)	50(2)	8(2)	14(2)	-6(2)
C(8)	47(2)	39(2)	44(2)	4(2)	7(2)	0(2)
C(9)	30(2)	32(2)	37(2)	3(1)	-5(1)	-3(1)
C(10)	30(2)	32(2)	41(2)	5(1)	-1(1)	2(1)
C(11)	27(2)	34(2)	55(2)	11(2)	-1(2)	0(1)
C(12)	37(2)	42(2)	91(3)	18(2)	-17(2)	1(2)
C(13)	34(2)	36(2)	74(3)	6(2)	-13(2)	1(2)
C(14)	30(2)	36(2)	107(4)	20(2)	-10(2)	0(2)
C(15)	39(2)	42(2)	151(5)	13(3)	-16(3)	-1(2)
C(16)	58(3)	46(3)	139(5)	-4(3)	-29(3)	-3(2)
C(17)	71(3)	59(3)	107(4)	-11(3)	-34(3)	4(3)
C(18)	55(3)	52(2)	70(3)	4(2)	-20(2)	2(2)
C(19)	37(2)	78(3)	118(5)	32(3)	9(3)	-8(2)
C(20)	91(4)	89(4)	57(3)	-1(3)	-8(3)	-2(3)
C(21)	29(2)	37(2)	42(2)	-1(1)	2(1)	1(1)
C(22)	61(3)	55(2)	54(2)	9(2)	22(2)	16(2)
C(23)	36(2)	37(2)	36(2)	0(1)	6(1)	10(1)
C(24)	37(2)	50(2)	39(2)	0(2)	7(2)	12(2)
C(25)	44(2)	70(3)	44(2)	1(2)	6(2)	27(2)
C(26)	69(3)	55(2)	57(3)	4(2)	14(2)	34(2)
C(27)	76(3)	37(2)	53(2)	3(2)	21(2)	15(2)
C(28)	54(2)	36(2)	40(2)	-2(2)	12(2)	10(2)
C(29)	33(2)	65(2)	60(2)	-9(2)	5(2)	3(2)
C(30)	56(3)	42(2)	73(3)	-14(2)	9(2)	-2(2)
C(31)	37(2)	37(2)	41(2)	7(1)	6(2)	2(1)
C(32)	48(2)	49(2)	36(2)	1(2)	8(2)	11(2)
C(33)	39(2)	54(2)	44(2)	9(2)	12(2)	12(2)
C(34)	41(2)	41(2)	41(2)	10(2)	3(2)	-4(2)
C(35)	64(3)	55(2)	35(2)	-4(2)	13(2)	1(2)
C(36)	48(2)	46(2)	48(2)	1(2)	14(2)	14(2)
C(37)	55(3)	70(3)	49(2)	12(2)	-5(2)	-8(2)
C(38)	45(2)	40(2)	42(2)	-5(2)	7(2)	-2(2)
C(39)	69(3)	48(2)	58(3)	12(2)	27(2)	2(2)
C(40)	99(4)	57(3)	81(3)	0(2)	55(3)	8(2)
C(41)	117(4)	39(2)	91(4)	-6(2)	45(3)	5(3)
C(42)	87(3)	42(2)	75(3)	8(2)	24(3)	-10(2)
C(43)	58(2)	48(2)	54(2)	-1(2)	16(2)	-11(2)
C(44)	228(9)	47(3)	225(9)	-10(4)	133(7)	29(4)
N(1)	30(1)	32(1)	49(2)	8(1)	3(1)	2(1)

N(2)	28(1)	39(2)	56(2)	10(1)	-4(1)	-3(1)
N(3)	30(1)	33(1)	39(2)	4(1)	3(1)	3(1)
N(4)	31(1)	32(1)	39(2)	2(1)	4(1)	7(1)
S(1)	35(1)	54(1)	51(1)	14(1)	0(1)	-2(1)
S(2)	41(1)	51(1)	66(1)	-16(1)	-10(1)	4(1)
Sn(1)	26(1)	33(1)	38(1)	2(1)	1(1)	4(1)

Crystal data and structure refinement of compound 3b

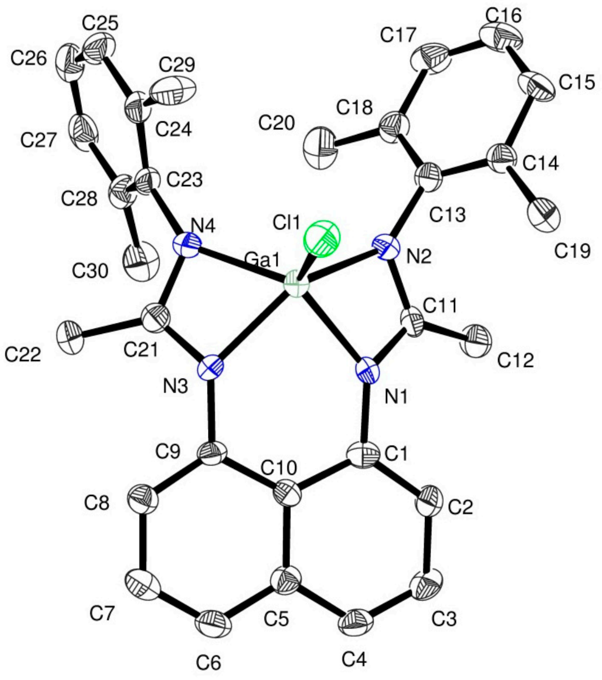

Figure S12. Asymmetric Unit

Table S33. Crystal data and structure refinement for R229.

Identification code	R229
Empirical formula	C30 H30 Cl Ga N4
Formula weight	551.75
Temperature	193(2) K
Wavelength	0.71073 A
Crystal system, space group	Monoclinic, P 21/c
Unit cell dimensions	a = 10.208(2) A alpha = 90 deg.<br>b = 19.494(4) A beta = 90.929(7) deg.<br>c = 13.220(3) A gamma = 90 deg.
Volume	2630.4(10) A^3
Z, Calculated density	4, 1.393 Mg/m^3
Absorption coefficient	1.174 mm^-1

F(000)	1144
Crystal size	0.100 x 0.040 x 0.040 mm
Theta range for data collection	3.255 to 24.147 deg.
Limiting indices	-11<=h<=11, -22<=k<=21, -15<=l<=15
Reflections collected / unique	26145 / 4150 [R(int) = 0.1436]
Completeness to theta = 24.147	98.7 %
Refinement method	Full-matrix least-squares on F^2
Data / restraints / parameters	4150 / 0 / 331
Goodness-of-fit on F^2	1.010
Final R indices [I>2sigma(I)]	R1 = 0.0502, wR2 = 0.0863
R indices (all data)	R1 = 0.1078, wR2 = 0.1079
Largest diff. peak and hole	0.447 and -0.523 e.A^-3

Table S34. Atomic coordinates (x 10^4) and equivalent isotropic displacement parameters (A^2 x 10^3) for R229.
U(eq) is defined as one third of the trace of the orthogonalized Uij tensor.

	x	y	z	U(eq)
C(1)	794(5)	4090(2)	5502(3)	24(1)
C(2)	-495(5)	4091(3)	5177(4)	29(1)
C(3)	-864(5)	4177(2)	4165(4)	30(1)
C(4)	81(5)	4279(2)	3470(4)	31(1)
C(5)	1417(5)	4316(2)	3747(4)	26(1)
C(6)	2352(5)	4442(3)	2991(4)	33(1)
C(7)	3651(5)	4491(3)	3232(4)	34(1)
C(8)	4072(5)	4419(2)	4234(4)	27(1)
C(9)	3212(5)	4270(2)	5002(3)	23(1)
C(10)	1825(5)	4219(2)	4782(3)	21(1)
C(11)	580(5)	3659(2)	7257(4)	24(1)
C(12)	-697(5)	3284(3)	7223(4)	34(1)
C(13)	1038(5)	3471(2)	9078(4)	26(1)
C(14)	121(5)	3868(2)	9593(4)	26(1)
C(15)	-103(5)	3716(3)	10601(4)	36(1)
C(16)	541(6)	3193(3)	11090(4)	43(2)
C(17)	1426(6)	2799(3)	10565(4)	40(2)
C(18)	1695(5)	2930(3)	9559(4)	31(1)
C(19)	-607(5)	4450(3)	9077(4)	35(1)
C(20)	2662(5)	2501(3)	9001(4)	46(2)
C(21)	4693(5)	3837(2)	6356(4)	26(1)
C(22)	5883(5)	3640(3)	5775(4)	33(1)
C(23)	5439(5)	3287(3)	7938(4)	28(1)
C(24)	6011(5)	3565(3)	8807(4)	32(1)
C(25)	6882(5)	3166(3)	9366(4)	40(2)
C(26)	7205(5)	2516(3)	9069(4)	43(2)
C(27)	6589(5)	2231(3)	8240(4)	40(2)
C(28)	5671(5)	2600(3)	7668(4)	32(1)
C(29)	5666(6)	4275(3)	9151(4)	46(2)
C(30)	4939(6)	2248(3)	6824(4)	45(2)
N(1)	1147(4)	4005(2)	6526(3)	24(1)
N(2)	1397(4)	3646(2)	8065(3)	25(1)
N(3)	3659(4)	4180(2)	6006(3)	24(1)
N(4)	4550(4)	3696(2)	7342(3)	25(1)
Cl(1)	2899(1)	5202(1)	8016(1)	35(1)
Ga(1)	2771(1)	4185(1)	7328(1)	24(1)

Table S35. Bond lengths [A] and angles [deg] for R229.

C(1)-C(2)	1.378(6)
C(1)-N(1)	1.406(6)
C(1)-C(10)	1.452(6)
C(2)-C(3)	1.394(6)
C(2)-H(2)	0.9500
C(3)-C(4)	1.359(6)
C(3)-H(3)	0.9500
C(4)-C(5)	1.408(7)
C(4)-H(4)	0.9500
C(5)-C(6)	1.414(6)
C(5)-C(10)	1.437(6)
C(6)-C(7)	1.362(7)
C(6)-H(6)	0.9500
C(7)-C(8)	1.393(7)
C(7)-H(7)	0.9500
C(8)-C(9)	1.384(6)
C(8)-H(8)	0.9500
C(9)-N(3)	1.408(6)
C(9)-C(10)	1.445(6)
C(11)-N(1)	1.320(5)
C(11)-N(2)	1.345(6)
C(11)-C(12)	1.494(6)
C(11)-Ga(1)	2.462(5)
C(12)-H(12A)	0.9800
C(12)-H(12B)	0.9800
C(12)-H(12C)	0.9800
C(13)-C(18)	1.397(7)
C(13)-C(14)	1.399(6)
C(13)-N(2)	1.435(6)
C(14)-C(15)	1.388(6)
C(14)-C(19)	1.513(7)
C(15)-C(16)	1.370(7)
C(15)-H(15)	0.9500
C(16)-C(17)	1.382(7)
C(16)-H(16)	0.9500
C(17)-C(18)	1.386(7)
C(17)-H(17)	0.9500
C(18)-C(20)	1.498(7)
C(19)-H(19A)	0.9800
C(19)-H(19B)	0.9800
C(19)-H(19C)	0.9800
C(20)-H(20A)	0.9800
C(20)-H(20B)	0.9800
C(20)-H(20C)	0.9800
C(21)-N(3)	1.327(6)
C(21)-N(4)	1.342(6)
C(21)-C(22)	1.498(6)
C(21)-Ga(1)	2.458(5)
C(22)-H(22A)	0.9800
C(22)-H(22B)	0.9800
C(22)-H(22C)	0.9800

C(23)-C(24)	1.391(7)
C(23)-C(28)	1.407(7)
C(23)-N(4)	1.433(6)
C(24)-C(25)	1.385(7)
C(24)-C(29)	1.501(7)
C(25)-C(26)	1.368(8)
C(25)-H(25)	0.9500
C(26)-C(27)	1.373(8)
C(26)-H(26)	0.9500
C(27)-C(28)	1.395(7)
C(27)-H(27)	0.9500
C(28)-C(30)	1.499(7)
C(29)-H(29A)	0.9800
C(29)-H(29B)	0.9800
C(29)-H(29C)	0.9800
C(30)-H(30A)	0.9800
C(30)-H(30B)	0.9800
C(30)-H(30C)	0.9800
N(1)-Ga(1)	1.984(4)
N(2)-Ga(1)	2.017(4)
N(3)-Ga(1)	1.981(4)
N(4)-Ga(1)	2.051(4)
Cl(1)-Ga(1)	2.1828(14)

C(2)-C(1)-N(1)	121.9(4)
C(2)-C(1)-C(10)	119.6(4)
N(1)-C(1)-C(10)	118.3(4)
C(1)-C(2)-C(3)	122.7(5)
C(1)-C(2)-H(2)	118.6
C(3)-C(2)-H(2)	118.6
C(4)-C(3)-C(2)	118.9(5)
C(4)-C(3)-H(3)	120.5
C(2)-C(3)-H(3)	120.5
C(3)-C(4)-C(5)	121.8(5)
C(3)-C(4)-H(4)	119.1
C(5)-C(4)-H(4)	119.1
C(4)-C(5)-C(6)	119.1(5)
C(4)-C(5)-C(10)	120.3(4)
C(6)-C(5)-C(10)	120.5(4)
C(7)-C(6)-C(5)	120.8(5)
C(7)-C(6)-H(6)	119.6
C(5)-C(6)-H(6)	119.6
C(6)-C(7)-C(8)	119.9(5)
C(6)-C(7)-H(7)	120.0
C(8)-C(7)-H(7)	120.0
C(9)-C(8)-C(7)	122.0(5)
C(9)-C(8)-H(8)	119.0
C(7)-C(8)-H(8)	119.0
C(8)-C(9)-N(3)	121.3(4)
C(8)-C(9)-C(10)	119.9(4)
N(3)-C(9)-C(10)	118.8(4)
C(5)-C(10)-C(9)	116.7(4)
C(5)-C(10)-C(1)	116.5(4)
C(9)-C(10)-C(1)	126.8(4)

N(1)-C(11)-N(2)	108.5(4)
N(1)-C(11)-C(12)	128.4(5)
N(2)-C(11)-C(12)	122.9(4)
N(1)-C(11)-Ga(1)	53.5(2)
N(2)-C(11)-Ga(1)	55.0(2)
C(12)-C(11)-Ga(1)	175.4(4)
C(11)-C(12)-H(12A)	109.5
C(11)-C(12)-H(12B)	109.5
H(12A)-C(12)-H(12B)	109.5
C(11)-C(12)-H(12C)	109.5
H(12A)-C(12)-H(12C)	109.5
H(12B)-C(12)-H(12C)	109.5
C(18)-C(13)-C(14)	121.1(5)
C(18)-C(13)-N(2)	118.4(4)
C(14)-C(13)-N(2)	120.3(4)
C(15)-C(14)-C(13)	118.1(5)
C(15)-C(14)-C(19)	120.4(5)
C(13)-C(14)-C(19)	121.6(4)
C(16)-C(15)-C(14)	121.8(5)
C(16)-C(15)-H(15)	119.1
C(14)-C(15)-H(15)	119.1
C(15)-C(16)-C(17)	119.3(5)
C(15)-C(16)-H(16)	120.4
C(17)-C(16)-H(16)	120.4
C(16)-C(17)-C(18)	121.4(5)
C(16)-C(17)-H(17)	119.3
C(18)-C(17)-H(17)	119.3
C(17)-C(18)-C(13)	118.4(5)
C(17)-C(18)-C(20)	120.7(5)
C(13)-C(18)-C(20)	120.9(5)
C(14)-C(19)-H(19A)	109.5
C(14)-C(19)-H(19B)	109.5
H(19A)-C(19)-H(19B)	109.5
C(14)-C(19)-H(19C)	109.5
H(19A)-C(19)-H(19C)	109.5
H(19B)-C(19)-H(19C)	109.5
C(18)-C(20)-H(20A)	109.5
C(18)-C(20)-H(20B)	109.5
H(20A)-C(20)-H(20B)	109.5
C(18)-C(20)-H(20C)	109.5
H(20A)-C(20)-H(20C)	109.5
H(20B)-C(20)-H(20C)	109.5
N(3)-C(21)-N(4)	110.1(4)
N(3)-C(21)-C(22)	126.7(4)
N(4)-C(21)-C(22)	123.2(5)
N(3)-C(21)-Ga(1)	53.5(2)
N(4)-C(21)-Ga(1)	56.6(2)
C(22)-C(21)-Ga(1)	178.5(4)
C(21)-C(22)-H(22A)	109.5
C(21)-C(22)-H(22B)	109.5
H(22A)-C(22)-H(22B)	109.5
C(21)-C(22)-H(22C)	109.5
H(22A)-C(22)-H(22C)	109.5
H(22B)-C(22)-H(22C)	109.5

C(24)-C(23)-C(28)	120.7(5)
C(24)-C(23)-N(4)	119.4(5)
C(28)-C(23)-N(4)	119.9(5)
C(25)-C(24)-C(23)	118.7(5)
C(25)-C(24)-C(29)	120.5(5)
C(23)-C(24)-C(29)	120.8(5)
C(26)-C(25)-C(24)	121.5(6)
C(26)-C(25)-H(25)	119.3
C(24)-C(25)-H(25)	119.3
C(25)-C(26)-C(27)	119.7(6)
C(25)-C(26)-H(26)	120.2
C(27)-C(26)-H(26)	120.2
C(26)-C(27)-C(28)	121.3(5)
C(26)-C(27)-H(27)	119.4
C(28)-C(27)-H(27)	119.4
C(27)-C(28)-C(23)	117.9(5)
C(27)-C(28)-C(30)	119.3(5)
C(23)-C(28)-C(30)	122.7(5)
C(24)-C(29)-H(29A)	109.5
C(24)-C(29)-H(29B)	109.5
H(29A)-C(29)-H(29B)	109.5
C(24)-C(29)-H(29C)	109.5
H(29A)-C(29)-H(29C)	109.5
H(29B)-C(29)-H(29C)	109.5
C(28)-C(30)-H(30A)	109.5
C(28)-C(30)-H(30B)	109.5
H(30A)-C(30)-H(30B)	109.5
C(28)-C(30)-H(30C)	109.5
H(30A)-C(30)-H(30C)	109.5
H(30B)-C(30)-H(30C)	109.5
C(11)-N(1)-C(1)	131.0(4)
C(11)-N(1)-Ga(1)	94.2(3)
C(1)-N(1)-Ga(1)	133.8(3)
C(11)-N(2)-C(13)	125.6(4)
C(11)-N(2)-Ga(1)	91.9(3)
C(13)-N(2)-Ga(1)	139.9(3)
C(21)-N(3)-C(9)	129.4(4)
C(21)-N(3)-Ga(1)	93.9(3)
C(9)-N(3)-Ga(1)	133.3(3)
C(21)-N(4)-C(23)	124.7(4)
C(21)-N(4)-Ga(1)	90.3(3)
C(23)-N(4)-Ga(1)	144.7(3)
N(3)-Ga(1)-N(1)	85.32(15)
N(3)-Ga(1)-N(2)	139.06(16)
N(1)-Ga(1)-N(2)	65.43(16)
N(3)-Ga(1)-N(4)	65.69(15)
N(1)-Ga(1)-N(4)	130.88(15)
N(2)-Ga(1)-N(4)	112.06(16)
N(3)-Ga(1)-Cl(1)	110.32(12)
N(1)-Ga(1)-Cl(1)	115.30(11)
N(2)-Ga(1)-Cl(1)	107.98(12)
N(4)-Ga(1)-Cl(1)	111.79(12)
N(3)-Ga(1)-C(21)	32.59(15)
N(1)-Ga(1)-C(21)	109.82(16)

N(2)-Ga(1)-C(21)	132.52(16)
N(4)-Ga(1)-C(21)	33.10(15)
Cl(1)-Ga(1)-C(21)	115.20(12)
N(3)-Ga(1)-C(11)	113.13(16)
N(1)-Ga(1)-C(11)	32.32(15)
N(2)-Ga(1)-C(11)	33.11(15)
N(4)-Ga(1)-C(11)	127.61(16)
Cl(1)-Ga(1)-C(11)	116.26(11)
C(21)-Ga(1)-C(11)	126.69(16)

Symmetry transformations used to generate equivalent atoms:

Table S36. Anisotropic displacement parameters (A^2 x 10^3) for R229. The anisotropic displacement factor exponent takes the form: -2 pi^2 [h^2 a*^2 U11 + ... + 2 h k a* b* U12]

	U11	U22	U33	U23	U13	U12
C(1)	30(3)	21(3)	22(3)	-4(2)	-4(2)	-4(2)
C(2)	20(3)	38(3)	29(3)	-1(3)	1(2)	-4(3)
C(3)	25(3)	32(3)	31(3)	-10(3)	-10(3)	-4(3)
C(4)	34(3)	34(3)	23(3)	-3(3)	-7(3)	5(3)
C(5)	26(3)	26(3)	24(3)	-4(2)	-3(2)	-1(2)
C(6)	40(4)	38(3)	20(3)	2(2)	-1(3)	2(3)
C(7)	41(4)	37(3)	23(3)	3(3)	6(3)	0(3)
C(8)	27(3)	30(3)	24(3)	-1(2)	1(2)	-2(2)
C(9)	23(3)	28(3)	18(3)	0(2)	-4(2)	-2(2)
C(10)	25(3)	18(3)	19(3)	-3(2)	2(2)	0(2)
C(11)	19(3)	27(3)	25(3)	-6(3)	6(3)	-2(2)
C(12)	29(3)	41(3)	33(3)	2(3)	2(3)	-10(3)
C(13)	21(3)	30(3)	26(3)	1(3)	1(2)	-7(3)
C(14)	26(3)	28(3)	25(3)	1(2)	1(3)	-4(3)
C(15)	44(4)	45(4)	20(3)	-1(3)	8(3)	-12(3)
C(16)	57(4)	46(4)	25(3)	7(3)	1(3)	-13(3)
C(17)	47(4)	38(4)	35(3)	16(3)	-7(3)	-6(3)
C(18)	28(3)	30(3)	33(3)	6(3)	0(3)	1(3)
C(19)	35(3)	42(3)	29(3)	-3(3)	7(3)	2(3)
C(20)	42(4)	40(4)	58(4)	15(3)	9(3)	4(3)
C(21)	24(3)	29(3)	25(3)	-3(2)	0(3)	-6(3)
C(22)	25(3)	45(3)	30(3)	-2(3)	7(3)	3(3)
C(23)	19(3)	39(4)	25(3)	11(3)	1(3)	3(3)
C(24)	21(3)	44(4)	29(3)	8(3)	4(3)	1(3)
C(25)	28(3)	57(4)	34(3)	19(3)	-5(3)	-2(3)
C(26)	29(3)	55(4)	46(4)	29(3)	9(3)	8(3)
C(27)	35(4)	42(4)	42(4)	16(3)	14(3)	5(3)
C(28)	25(3)	33(3)	39(3)	4(3)	1(3)	-5(3)
C(29)	54(4)	55(4)	30(3)	0(3)	-15(3)	4(3)
C(30)	47(4)	39(4)	51(4)	1(3)	6(3)	2(3)
N(1)	19(2)	33(3)	20(2)	4(2)	5(2)	1(2)
N(2)	24(2)	32(3)	20(2)	4(2)	1(2)	-1(2)
N(3)	18(2)	33(2)	21(2)	1(2)	-2(2)	4(2)
N(4)	25(3)	30(3)	19(2)	-1(2)	0(2)	3(2)
Cl(1)	33(1)	35(1)	36(1)	-7(1)	-3(1)	3(1)
Ga(1)	19(1)	30(1)	21(1)	0(1)	1(1)	0(1)

Calculations

Calculations were performed with the Gaussian 16 suite of programs [1] using the density functional method B3LYP with dispersion (D3). [2, 3] Tin atom was treated with a Stuttgart-Dresden pseudopotential in combination with its adapted basis set.[4] All other atoms have been described with a 6-31G(d,p) basis set. Geometry optimisations were carried out without any symmetry restrictions. Frequency calculations were undertaken to confirm the nature of the stationary points, yielding one imaginary frequency for transition states (TS) and all of them positive for *minima*. The connectivity of the transition states and their adjacent *minima* was confirmed by intrinsic reaction coordinate (IRC) calculations.[5,6]

1. **Gaussian 16, Revision B.01**, M. J. Frisch, G. W. Trucks, H. B. Schlegel, G. E. Scuseria, M. A. Robb, J. R. Cheeseman, G. Scalmani, V. Barone, G. A. Petersson, H. Nakatsuji, X. Li, M. Caricato, A. V. Marenich, J. Bloino, B. G. Janesko, R. Gomperts, B. Mennucci, H. P. Hratchian, J. V. Ortiz, A. F. Izmaylov, J. L. Sonnenberg, D. Williams-Young,F. Ding, F. Lipparini, F. Egidi, J. Goings, B. Peng, A. Petrone, T. Henderson, D. Ranasinghe, V. G. Zakrzewski, J. Gao, N. Rega, G. Zheng, W. Liang, M. Hada, M. Ehara, K. Toyota, R. Fukuda, J. Hasegawa, M. Ishida, T. Nakajima, Y. Honda, O. Kitao, H. Nakai, T. Vreven, K. Throssell, J. A. Montgomery, Jr., J. E. Peralta,F. Ogliaro, M. J. Bearpark, J. J. Heyd, E. N. Brothers, K. N. Kudin, V. N. Staroverov, T. A. Keith, R. Kobayashi, J. Normand, K. Raghavachari, A. P. Rendell, J. C. Burant, S. S. Iyengar, J. Tomasi, M. Cossi, J. M. Millam, M. Klene, C. Adamo, R. Cammi, J. W. Ochterski, R. L. Martin, K. Morokuma, O. Farkas, J. B. Foresman, and D. J. Fox, Gaussian, Inc., Wallingford CT, 2016.

2. a) Becke, A. D. Density-functional exchange-energy approximation with correct asymptotic behavior. *Phys. Rev. A* **1988**, 38, 3098-3100. https://doi.org/10.1103/PhysRevA.38.3098 ; b) Becke, A. D. Density-functional thermochemistry. III. The role of exact exchange. *J. Chem. Phys.* **1993**, *98*, 5648-5652. https://doi.org/10.1063/1.464913 ; c) Lee, C.; Yang, W.; Parr, R. G. Development of the Colle-Salvetti correlation-energy formula into a functional of the electron density. *Phys. Rev. B* **1988**, *37*, 785-789. https://doi.org/10.1103/PhysRevB.37.785

3. Grimme, S. Semiempirical GGA-type density functional constructed with a long-range dispersion correction. *J. Comp. Chem.*, **2006**, *27*, 1787-1799. https://doi.org/10.1002/jcc.20495

4. Andrae, D.; Häussermann, U.; Dolg, M.; Stoll, H.; Preuss, H. Energy-adjustedab initio pseudopotentials for the second and third row transition elements. *Theor. Chim. Acta*. **1990**, *77*, 123–141. https://doi.org/10.1007/BF01114537

5. Fukui, K. The path of chemical reactions - the IRC approach. *Acc. Chem. Res.* **1981**, *14*, 363–368. https://doi.org/10.1021/ar00072a001

6. Hratchian, H. P.; Schlegel, H. B. In Theory and Applications of Computational Chemistry: The First 40 Years, Eds D. C. E. Dykstra, G. Frenking, K. S. Kim, G. Scuseria. Elsevier., Amsterdam, 2005.

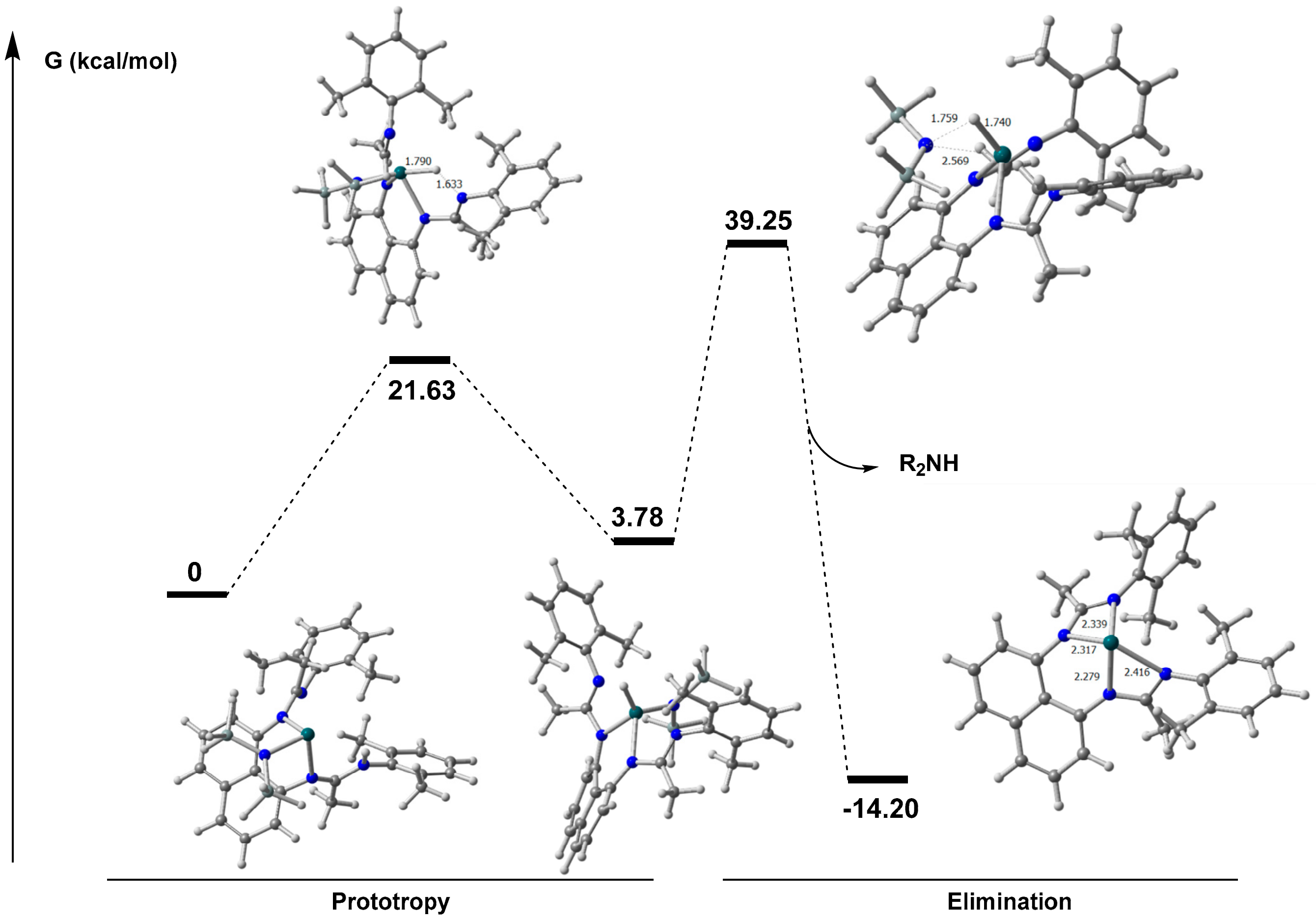

DFT calculations for the formation of **L1Sn**.

Coordinates, energies

L1

Sum of electronic and thermal Free Energies= -2021.440506 au

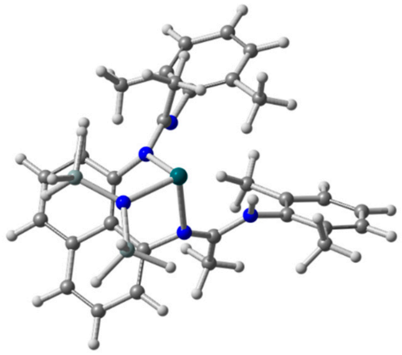

Sn	-1.331745000000	0.665129000000	-1.771438000000
N	1.342588000000	-1.965339000000	-0.026149000000
C	-3.098596000000	-2.529756000000	2.590868000000
H	-3.776410000000	-2.909169000000	3.349073000000
C	2.395962000000	2.673886000000	0.475996000000
C	3.336214000000	1.868689000000	1.145782000000
C	-3.027583000000	-1.134078000000	2.373977000000
N	-1.036840000000	1.400586000000	0.462289000000
C	0.342767000000	1.823397000000	2.467140000000
C	4.611217000000	2.409666000000	1.364445000000
H	5.348785000000	1.801756000000	1.880219000000
C	-2.319885000000	-3.382585000000	1.841651000000
H	-2.367337000000	-4.455123000000	2.003962000000
C	0.720171000000	-1.934788000000	-2.434087000000
N	-0.738039000000	-1.130530000000	-0.641957000000
C	-3.846485000000	-0.252274000000	3.131048000000
H	-4.533974000000	-0.684026000000	3.852302000000
C	4.949178000000	3.682003000000	0.919935000000
H	5.946881000000	4.072001000000	1.095384000000
C	-2.122670000000	-0.603394000000	1.388612000000
C	4.233185000000	-4.230868000000	0.206254000000
H	4.465287000000	-5.193420000000	0.654534000000
C	2.721194000000	3.965717000000	0.013208000000
C	2.938069000000	-3.728882000000	0.316338000000
C	-1.401673000000	-1.520094000000	0.543460000000
C	0.495175000000	-1.682876000000	-0.955231000000
C	3.048587000000	0.470642000000	1.632050000000
H	2.893655000000	0.460279000000	2.716951000000
H	3.899555000000	-0.181669000000	1.423458000000
H	2.174510000000	0.014159000000	1.167035000000
C	1.859548000000	-4.493053000000	1.037188000000
H	1.136130000000	-4.922210000000	0.332501000000
H	1.289909000000	-3.830189000000	1.695362000000
H	2.280695000000	-5.314658000000	1.622911000000
C	0.094771000000	1.795014000000	0.983498000000
C	4.007063000000	4.454057000000	0.241740000000
H	4.266382000000	5.448135000000	-0.109852000000
C	-1.504572000000	-2.881575000000	0.816436000000
H	-0.964282000000	-3.572187000000	0.180866000000
C	-3.785761000000	1.106467000000	2.951647000000
H	-4.432051000000	1.770088000000	3.516775000000
C	3.626431000000	-1.742087000000	-0.941820000000
C	5.228012000000	-3.511948000000	-0.457475000000
H	6.236411000000	-3.908027000000	-0.527736000000
C	-2.854022000000	1.646819000000	2.039692000000
H	-2.766126000000	2.720451000000	1.909343000000
C	2.622679000000	-2.480038000000	-0.270957000000
C	-2.021821000000	0.823184000000	1.311924000000
C	4.918757000000	-2.277581000000	-1.017496000000
H	5.690851000000	-1.705104000000	-1.525934000000
C	1.696976000000	4.796850000000	-0.717824000000
H	0.756846000000	4.858842000000	-0.160478000000
H	1.455275000000	4.377129000000	-1.703051000000

H	2.067318000000	5.811141000000	-0.880700000000
C	3.358680000000	-0.390728000000	-1.561671000000
H	2.475071000000	0.084583000000	-1.134235000000
H	3.197277000000	-0.468430000000	-2.643579000000
H	4.207948000000	0.281372000000	-1.408631000000
N	1.090804000000	2.192498000000	0.153217000000
H	0.834329000000	2.234783000000	-0.827035000000
N	-3.415153000000	0.352186000000	-1.456907000000
Si	-4.104156000000	-1.241852000000	-1.528045000000
H	-4.410959000000	-1.836196000000	-0.200785000000
H	-5.388116000000	-1.207430000000	-2.290899000000
H	-3.173443000000	-2.156366000000	-2.244989000000
Si	-4.433477000000	1.725096000000	-1.185908000000
H	-5.303955000000	1.586441000000	0.013989000000
H	-3.562134000000	2.923103000000	-0.989887000000
H	-5.337622000000	2.027294000000	-2.334849000000
H	-0.493411000000	2.308850000000	2.968553000000
H	0.396371000000	0.798409000000	2.842745000000
H	1.266692000000	2.345789000000	2.705392000000
H	0.813202000000	-0.997923000000	-2.994882000000
H	-0.138389000000	-2.475512000000	-2.841196000000
H	1.629973000000	-2.513317000000	-2.596844000000

TS1 (Prototropy from NH → SnH)

Sum of electronic and thermal Free Energies= -2021.406023 au

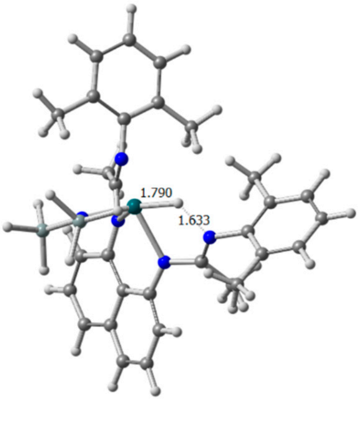

Sn	0.279203000000	-0.193096000000	0.752522000000
N	2.165799000000	-1.074855000000	-0.301388000000
C	1.299949000000	5.034334000000	-1.795186000000
H	1.260993000000	6.088254000000	-2.052031000000
C	-3.762050000000	-1.526349000000	-0.008655000000
C	-3.575846000000	-2.633616000000	-0.861065000000
C	0.113422000000	4.409373000000	-1.330439000000
N	-1.301745000000	1.108401000000	-0.142472000000
C	-3.336414000000	0.798933000000	-1.582044000000
C	-4.649364000000	-3.497779000000	-1.088806000000
H	-4.510628000000	-4.348264000000	-1.750957000000
C	2.473010000000	4.331561000000	-1.876653000000
H	3.391138000000	4.817371000000	-2.192133000000
C	3.364074000000	0.090488000000	-2.132933000000
N	1.420588000000	0.949698000000	-0.740795000000
C	-1.053530000000	5.201980000000	-1.190384000000
H	-1.002527000000	6.250629000000	-1.465755000000
C	-5.883569000000	-3.284760000000	-0.477532000000
H	-6.707830000000	-3.967128000000	-0.660368000000
C	0.103968000000	3.007635000000	-0.989702000000
C	3.195440000000	-4.425961000000	-1.516374000000
H	2.932095000000	-5.136973000000	-2.294472000000
C	-5.001522000000	-1.308670000000	0.626837000000
C	2.513054000000	-3.207023000000	-1.451805000000
C	1.354299000000	2.292755000000	-1.120974000000
C	2.323982000000	0.003850000000	-1.053815000000
C	-2.233105000000	-2.874427000000	-1.502439000000
H	-1.884795000000	-1.998131000000	-2.061616000000

H	-2.272669000000	-3.720327000000	-2.193223000000
H	-1.468145000000	-3.091352000000	-0.749301000000
C	1.413951000000	-2.883016000000	-2.431505000000
H	1.722241000000	-2.133027000000	-3.169647000000
H	0.537156000000	-2.477832000000	-1.920169000000
H	1.107664000000	-3.776312000000	-2.980978000000
C	-2.440414000000	0.417432000000	-0.417359000000
C	-6.050444000000	-2.197457000000	0.377537000000
H	-7.007400000000	-2.032413000000	0.865345000000
C	-2.205275000000	4.657977000000	-0.681275000000
H	-3.090961000000	5.268526000000	-0.534488000000
C	3.853928000000	-2.605954000000	0.514543000000
C	4.189613000000	-4.740067000000	-0.593182000000
H	4.706034000000	-5.693036000000	-0.653731000000
C	-2.233702000000	3.301340000000	-0.327750000000
H	-3.130560000000	2.887097000000	0.118383000000
C	2.865423000000	-2.292902000000	-0.438810000000
C	-1.142939000000	2.452932000000	-0.509794000000
C	4.509283000000	-3.835585000000	0.418036000000
H	5.275449000000	-4.084079000000	1.146952000000
C	-5.173837000000	-0.137546000000	1.559169000000
H	-5.166779000000	0.817994000000	1.020358000000
H	-4.354662000000	-0.091094000000	2.283124000000
H	-6.121028000000	-0.204092000000	2.100423000000
C	4.185183000000	-1.623546000000	1.607760000000
H	4.903465000000	-2.050092000000	2.311969000000
H	3.289643000000	-1.324204000000	2.161816000000
H	4.613347000000	-0.700539000000	1.202051000000
N	-2.685288000000	-0.666836000000	0.286026000000
H	-1.215445000000	-1.178115000000	0.779199000000
N	1.025681000000	0.772738000000	2.427710000000
Si	2.279509000000	1.975387000000	2.286246000000
H	2.843064000000	2.212169000000	3.646099000000
H	3.381440000000	1.512353000000	1.404320000000
H	1.793337000000	3.275552000000	1.758540000000
Si	0.232076000000	0.512661000000	3.953370000000
H	-0.418082000000	1.743281000000	4.485856000000
H	-0.822373000000	-0.518724000000	3.752924000000
H	1.172753000000	0.027964000000	5.001369000000
H	3.583157000000	-0.907413000000	-2.513545000000
H	4.296329000000	0.494372000000	-1.725512000000
H	3.034920000000	0.743062000000	-2.942957000000
H	-4.311657000000	1.149519000000	-1.233413000000
H	-3.520829000000	-0.094929000000	-2.183165000000
H	-2.889143000000	1.576032000000	-2.199057000000
C	2.500480000000	2.973953000000	-1.516224000000
H	3.451420000000	2.462692000000	-1.501545000000

SnH derivative

Sum of electronic and thermal Free Energies= -2021.434479 au

Sn	0.006770000000	-0.355066000000	-0.371221000000
N	2.394203000000	-0.471119000000	-0.595504000000

C	0.704582000000	5.573690000000	-0.721809000000
H	0.643253000000	6.651064000000	-0.603780000000
C	-3.515866000000	-1.515846000000	-0.470199000000
C	-4.040295000000	-1.548692000000	-1.778071000000
C	-0.142870000000	4.754843000000	0.070380000000
N	-1.352905000000	1.227589000000	0.246820000000
C	-3.892946000000	1.287281000000	0.324097000000
C	-5.063416000000	-2.455685000000	-2.067913000000
H	-5.473823000000	-2.484280000000	-3.073601000000
C	1.565216000000	5.009568000000	-1.629953000000
H	2.180646000000	5.634357000000	-2.269932000000
C	3.777662000000	1.562959000000	-0.917666000000
N	1.245065000000	1.402286000000	-0.791822000000
C	-1.026985000000	5.351482000000	1.007662000000
H	-1.021850000000	6.432153000000	1.110286000000
C	-5.550791000000	-3.321317000000	-1.091078000000
H	-6.341527000000	-4.025001000000	-1.332431000000
C	-0.105321000000	3.326333000000	-0.076426000000
C	4.834991000000	-3.195629000000	-1.101811000000
H	5.091453000000	-3.926775000000	-1.863395000000
C	-3.992634000000	-2.391142000000	0.523923000000
C	3.807077000000	-2.285913000000	-1.353487000000
C	0.945759000000	2.766009000000	-0.890969000000
C	2.474305000000	0.827238000000	-0.778477000000
C	-3.498553000000	-0.621833000000	-2.837216000000
H	-3.708403000000	0.429789000000	-2.608976000000
H	-3.939854000000	-0.844037000000	-3.811881000000
H	-2.411342000000	-0.713285000000	-2.922556000000
C	3.038298000000	-2.311369000000	-2.648575000000
H	3.078047000000	-1.343406000000	-3.159602000000
H	1.979063000000	-2.526229000000	-2.472745000000
H	3.434335000000	-3.073180000000	-3.324586000000
C	-2.563196000000	0.606772000000	0.150573000000
C	-5.013023000000	-3.287213000000	0.193715000000
H	-5.385019000000	-3.965540000000	0.956645000000
C	-1.850071000000	4.571124000000	1.780940000000
H	-2.491070000000	5.020370000000	2.533208000000
C	4.158143000000	-1.319479000000	0.874107000000
C	5.525194000000	-3.178409000000	0.109606000000
H	6.319035000000	-3.895488000000	0.294489000000
C	-1.898031000000	3.179677000000	1.575691000000
H	-2.572479000000	2.575095000000	2.167771000000
C	3.482966000000	-1.336565000000	-0.364141000000
C	-1.119816000000	2.560402000000	0.604274000000
C	5.182436000000	-2.248775000000	1.086780000000
H	5.703826000000	-2.245471000000	2.039916000000
C	-3.407607000000	-2.347619000000	1.910879000000
H	-3.668061000000	-1.416862000000	2.427776000000
H	-2.316006000000	-2.386027000000	1.877201000000
H	-3.772006000000	-3.181108000000	2.516407000000
C	3.768364000000	-0.344877000000	1.956605000000
H	4.152684000000	-0.672361000000	2.925466000000
H	2.682108000000	-0.258546000000	2.024371000000
H	4.161275000000	0.662001000000	1.771931000000
N	-2.439463000000	-0.652344000000	-0.177416000000
H	-0.170068000000	-1.189565000000	-1.835965000000
N	0.242128000000	-1.221606000000	1.492141000000
Si	-0.173104000000	-0.276804000000	2.888139000000
H	0.250599000000	-1.040511000000	4.095876000000

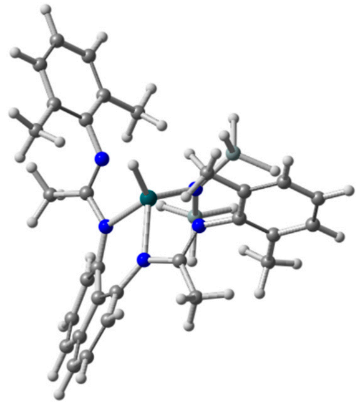

H	0.529329000000	1.033822000000	2.885078000000
H	-1.626314000000	0.008029000000	3.025234000000
Si	0.823333000000	-2.854311000000	1.603919000000
H	-0.186475000000	-3.766785000000	2.214205000000
H	1.096498000000	-3.337366000000	0.224413000000
H	2.062864000000	-2.972427000000	2.414801000000
H	4.585241000000	0.960050000000	-0.503530000000
H	3.733384000000	2.532915000000	-0.418993000000
H	4.003900000000	1.740965000000	-1.972927000000
H	-4.220464000000	1.225748000000	1.366307000000
H	-4.639147000000	0.774407000000	-0.284664000000
H	-3.838479000000	2.341485000000	0.050340000000
C	1.688558000000	3.609440000000	-1.709056000000
H	2.397165000000	3.178545000000	-2.403734000000

Amine elimination

Sum of electronic and thermal Free Energies= -2021.377957 au

Sn	-0.015271000000	0.027585000000	0.923622000000
N	-1.014386000000	-1.889642000000	0.061604000000
C	5.090374000000	-1.390345000000	-1.602219000000
H	6.111352000000	-1.349545000000	-1.968873000000
C	-2.296066000000	2.497909000000	-0.227936000000
C	-3.528207000000	2.091286000000	-0.773984000000
C	4.320354000000	-0.198096000000	-1.622063000000
N	0.935411000000	1.136177000000	-0.706496000000
C	-4.633805000000	2.936189000000	-0.643036000000
H	-5.584896000000	2.628597000000	-1.068380000000
C	4.565834000000	-2.559232000000	-1.113070000000
H	5.165455000000	-3.463156000000	-1.076527000000
N	1.139115000000	-1.506609000000	-0.146031000000
C	4.911818000000	0.994699000000	-2.112459000000
H	5.939741000000	0.957166000000	-2.459176000000
C	-4.528099000000	4.155548000000	0.021134000000
H	-5.395481000000	4.801552000000	0.115816000000
C	2.960861000000	-0.200595000000	-1.151145000000
C	-4.160778000000	-3.203588000000	-1.409624000000
H	-4.643762000000	-3.290906000000	-2.378996000000
C	-2.180825000000	3.719514000000	0.470017000000
C	-2.887758000000	-2.626525000000	-1.338479000000
C	2.441193000000	-1.454269000000	-0.656761000000
C	0.145998000000	-2.350497000000	-0.408887000000
C	-3.639974000000	0.769887000000	-1.481370000000
H	-2.959698000000	0.715207000000	-2.336969000000
H	-4.657246000000	0.600589000000	-1.842609000000
H	-3.375234000000	-0.059324000000	-0.819645000000
C	-2.220146000000	-2.119967000000	-2.593591000000
H	-1.609065000000	-2.890859000000	-3.077137000000
H	-1.564456000000	-1.273100000000	-2.380638000000
H	-2.969850000000	-1.800922000000	-3.321994000000
C	-0.101110000000	1.896268000000	-1.064366000000
C	-3.308725000000	4.537911000000	0.575794000000
H	-3.227255000000	5.479910000000	1.110716000000
C	3.246477000000	-2.581770000000	-0.627436000000

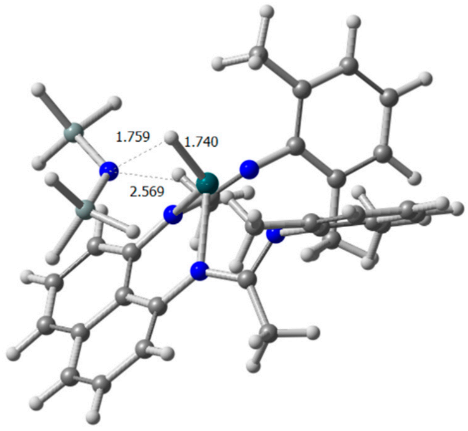

H	2.856659000000	-3.486567000000	-0.178739000000
C	4.207331000000	2.171013000000	-2.122761000000
H	4.669390000000	3.090405000000	-2.468100000000
C	-2.931811000000	-2.944832000000	1.096359000000
C	-4.815403000000	-3.646232000000	-0.264777000000
H	-5.804738000000	-4.086764000000	-0.339171000000
C	2.882223000000	2.198022000000	-1.655625000000
H	2.364261000000	3.145814000000	-1.596482000000
C	-2.270069000000	-2.514520000000	-0.073367000000
C	2.238283000000	1.052440000000	-1.206330000000
C	-4.202059000000	-3.510755000000	0.980046000000
H	-4.712958000000	-3.849323000000	1.876843000000
C	-0.880801000000	4.120057000000	1.119635000000
H	-0.492068000000	3.325002000000	1.762655000000
H	-1.013880000000	5.015938000000	1.730312000000
H	-0.092058000000	4.335093000000	0.390418000000
C	-2.270960000000	-2.787382000000	2.441124000000
H	-2.176732000000	-1.731375000000	2.720012000000
H	-1.256746000000	-3.198525000000	2.445027000000
H	-2.850590000000	-3.286317000000	3.221395000000
N	-1.175384000000	1.638373000000	-0.317753000000
H	0.354394000000	0.416615000000	2.578398000000
N	2.096925000000	0.367399000000	2.345447000000
Si	2.925147000000	1.860822000000	2.242793000000
H	2.000090000000	2.882337000000	1.657557000000
H	3.372848000000	2.413396000000	3.560426000000
H	4.134194000000	1.845844000000	1.364677000000
Si	2.605448000000	-1.109567000000	3.036842000000
H	3.000114000000	-1.031817000000	4.477826000000
H	1.439087000000	-2.057297000000	2.971166000000
H	3.729352000000	-1.794710000000	2.332061000000
C	0.307075000000	-3.670636000000	-1.102976000000
H	-0.655407000000	-4.073803000000	-1.411349000000
H	0.971996000000	-3.568317000000	-1.963952000000
H	0.768671000000	-4.383695000000	-0.413671000000
C	-0.124662000000	2.873098000000	-2.203715000000
H	0.201448000000	3.863870000000	-1.872963000000
H	0.549802000000	2.546326000000	-2.997412000000
H	-1.142625000000	2.968339000000	-2.584678000000

L1Sn

Sum of electronic and thermal Free Energies= -1383.498096 au

Sn	-0.073500000000	0.048241000000	-1.607961000000
N	0.305976000000	-1.891444000000	-0.357900000000
C	-5.752003000000	-0.343197000000	0.362869000000
H	-6.774774000000	-0.100632000000	0.635195000000
N	1.150883000000	1.381768000000	-0.008333000000
C	2.350574000000	2.126564000000	0.062408000000
C	2.408389000000	3.414621000000	-0.518380000000
C	-4.753632000000	0.650330000000	0.544536000000
N	-1.063143000000	1.224820000000	0.073674000000

C	0.076486000000	2.637833000000	1.839911000000
H	1.030864000000	2.493445000000	2.348991000000
H	-0.748045000000	2.385605000000	2.509355000000
H	-0.007114000000	3.699698000000	1.590735000000
C	3.611867000000	4.123447000000	-0.472613000000
H	3.656838000000	5.113636000000	-0.917992000000
C	-5.436551000000	-1.573351000000	-0.157378000000
H	-6.206993000000	-2.323493000000	-0.307818000000
C	-1.492382000000	-3.296251000000	0.682735000000
H	-2.225014000000	-2.954481000000	1.418377000000
H	-0.677742000000	-3.819265000000	1.180854000000
H	-2.003497000000	-4.001511000000	0.021719000000
N	-1.776297000000	-1.237228000000	-0.705584000000
C	-5.123577000000	1.918636000000	1.061718000000
H	-6.165060000000	2.094616000000	1.312615000000
C	4.748186000000	3.572888000000	0.114956000000
H	5.676883000000	4.134746000000	0.139752000000
C	-3.383645000000	0.372308000000	0.203206000000
C	3.332037000000	-4.005586000000	-0.141848000000
H	3.994507000000	-4.550455000000	-0.808679000000
C	3.503917000000	1.550674000000	0.631598000000
C	2.235075000000	-3.328686000000	-0.679009000000
C	-3.089748000000	-0.935132000000	-0.331438000000
C	-0.985702000000	-2.130515000000	-0.124626000000
C	1.211345000000	4.004611000000	-1.223005000000
H	0.777112000000	3.290104000000	-1.929506000000
H	1.496760000000	4.901381000000	-1.778246000000
H	0.409088000000	4.290746000000	-0.533622000000
C	1.965612000000	-3.338880000000	-2.161363000000
H	2.080443000000	-2.339529000000	-2.597239000000
H	0.941187000000	-3.656882000000	-2.379479000000
H	2.654908000000	-4.010081000000	-2.679669000000
C	0.036053000000	1.769202000000	0.608338000000
C	4.689680000000	2.290236000000	0.653484000000
H	5.575500000000	1.847553000000	1.100639000000
C	-4.106095000000	-1.859979000000	-0.515111000000
H	-3.856985000000	-2.815010000000	-0.963856000000
C	-4.182814000000	2.905311000000	1.213705000000
H	-4.466927000000	3.887783000000	1.578589000000
C	1.631340000000	-2.582851000000	1.578057000000
C	3.580555000000	-3.992260000000	1.229467000000
H	4.437080000000	-4.523002000000	1.633478000000
C	-2.839870000000	2.659682000000	0.877856000000
H	-2.129702000000	3.472718000000	0.944623000000
C	1.374300000000	-2.627237000000	0.189756000000
C	-2.403554000000	1.419679000000	0.418370000000
C	2.734280000000	-3.282854000000	2.077121000000
H	2.939157000000	-3.249018000000	3.143698000000
C	3.450746000000	0.163986000000	1.207305000000
H	3.118086000000	-0.566336000000	0.465550000000
H	2.739696000000	0.103322000000	2.035674000000
H	4.430345000000	-0.149322000000	1.576229000000
C	0.775662000000	-1.752151000000	2.502015000000
H	-0.137703000000	-2.272846000000	2.810340000000
H	0.467785000000	-0.823808000000	2.014464000000
H	1.327202000000	-1.503603000000	3.412429000000

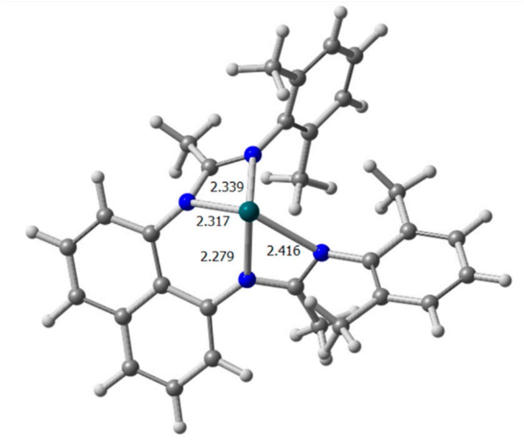

Dimethylamine

Sum of electronic and thermal Free Energies= -637.965039 au

N	0.000013000000	0.619231000000	0.013062000000
Si	-1.568800000000	-0.134296000000	-0.001379000000
Si	1.568801000000	-0.134298000000	-0.001374000000
H	-0.000056000000	1.627612000000	-0.100148000000
H	2.501052000000	0.609543000000	0.888029000000
H	2.199845000000	-0.185867000000	-1.350864000000
H	1.398947000000	-1.524682000000	0.486474000000
H	-1.399201000000	-1.524548000000	0.486909000000
H	-2.501148000000	0.609826000000	0.887694000000
H	-2.199551000000	-0.186184000000	-1.350986000000

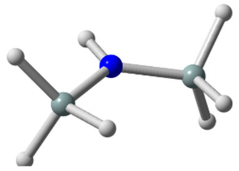

$SnCl_2$

Sum of electronic and thermal Free Energies= -923.922288 au

Sn	0.000000000000	0.000000000000	0.639572000000
Cl	0.000000000000	1.823507000000	-0.940547000000
Cl	0.000000000000	-1.823507000000	-0.940547000000

$GeCl_2$

Sum of electronic and thermal Free Energies= -2995.495709 au

Cl	0.000000000000	-1.702742000000	-0.688640000000
Cl	0.000000000000	1.702742000000	-0.688640000000
Ge	0.000000000000	0.000000000000	0.731680000000
